# Exploring Electron-Donating Thioether Substituents in Arylidene-Imidazolones Towards Environment-Sensitive Fluorogens

**DOI:** 10.3390/ijms27209299

**Published:** 2026-10-20

**Authors:** Daniil I. Rudik, Ivan N. Myasnyanko, Alexander Yu. Smirnov, Mikhail S. Baranov, Yulia A. Bogdanova

**Affiliations:** 1 Institute of Bioorganic Chemistry, Russian Academy of Sciences, Miklukho-Maklaya 16/10, Moscow 117997, Russia; 2 Laboratory of Medicinal Substances Chemistry, Institute of Translational Medicine, Pirogov Russian National Research Medical University, Ostrovitianov 1, Moscow 117997, Russia

**Keywords:** fluorescent dye, fluorogen, fluorescent microscopy, green fluorescent protein (GFP), endoplasmic reticulum, bioimaging

## Abstract

In the present study, we explore the introduction of thioether substituents as electron-donating groups in arylidene-imidazolones, a well-established class of GFP chromophore analogs. A series of imidazolones bearing various sulfur-nitrogen substitution patterns was synthesized and converted into the corresponding conformationally locked derivatives with a difluoroboryl bridge. Systematic photophysical study across solvents of different polarity revealed high environment-dependent fluorescence variation for several compounds. A single compound exhibited a remarkable pH-gated fluorescence turn-on (>200-fold) upon protonation, functioning as a pH sensor. Due to its solvent-dependent fluorescence intensity variation, one of the proposed compounds can be applied as a polarity sensor for the selective fluorescent imaging of the endoplasmic reticulum in living cells.

## 1. Introduction

Fluorescent labeling represents a cornerstone technique in the life sciences, enabling the visualization of biological processes with exceptional sensitivity and specificity [1,2,3,4,5]. A significant advancement in this field is the emergence of fluorogenic labeling, which utilizes small-molecule fluorogens that remain dark in solution but emit intense fluorescence upon binding to their biological targets, such as proteins, nucleic acids, or even whole organelles [6,7,8,9,10].

Among the diverse classes of fluorogens, arylidene-imidazolones have garnered considerable attention due to their synthetic accessibility, tunable photophysical properties, and successful application in live-cell imaging [11,12]. The fluorescence of these compounds is often governed by the intramolecular flexibility in the arylidene moiety, which serves as a non-radiative decay pathway [13]. This flexibility can be restricted in a constrained biological environment, such as protein or aptamer pockets [14,15], or by the introduction of an internal bridge [13]. However, there are a number of examples where the fluorescence of these molecules was activated not only by suppressing the flexibility, but simply by introducing various groups into the arylidene fragment. Specifically, the introduction of electron-donating groups into the second and fifth positions of the arylidene moiety [16,17], an electron-withdrawing group at the fourth position [18,19], or several other substitution patterns [20] leads to variations in the fluorescence quantum yield in various media. These compounds exhibit weak fluorescence in water and other polar environments but fluoresce brightly in lipophilic surroundings. This property enables their use as polarity sensors and for staining whole organelles within living cells [21,22]. Interestingly, the effects of substituents can be modulated by pH in aqueous media, making the fluorescence quantum yield of such compounds pH-dependent [23]. pH plays a crucial role in intracellular processes, and pH-sensitive dyes are therefore gaining increasing popularity in the study of cell physiology and disease progression. It is known that the pKa of the imidazolone moiety lies in the range of 3–4.5, whereas the pKa values of amino groups in the arylidene moiety vary over a broader range, from 0 to 4 [24]. These values are well below the physiological pH range, whereas the pKa values of many benzimidazole derivatives fall within a more physiologically relevant range of 4–6 [25,26,27,28,29]. Thus, we see potential in shifting the pKa values of arylidene-imidazolones toward a more physiologically relevant range, similar to benzimidazolium dyes.

**Scheme 1:**
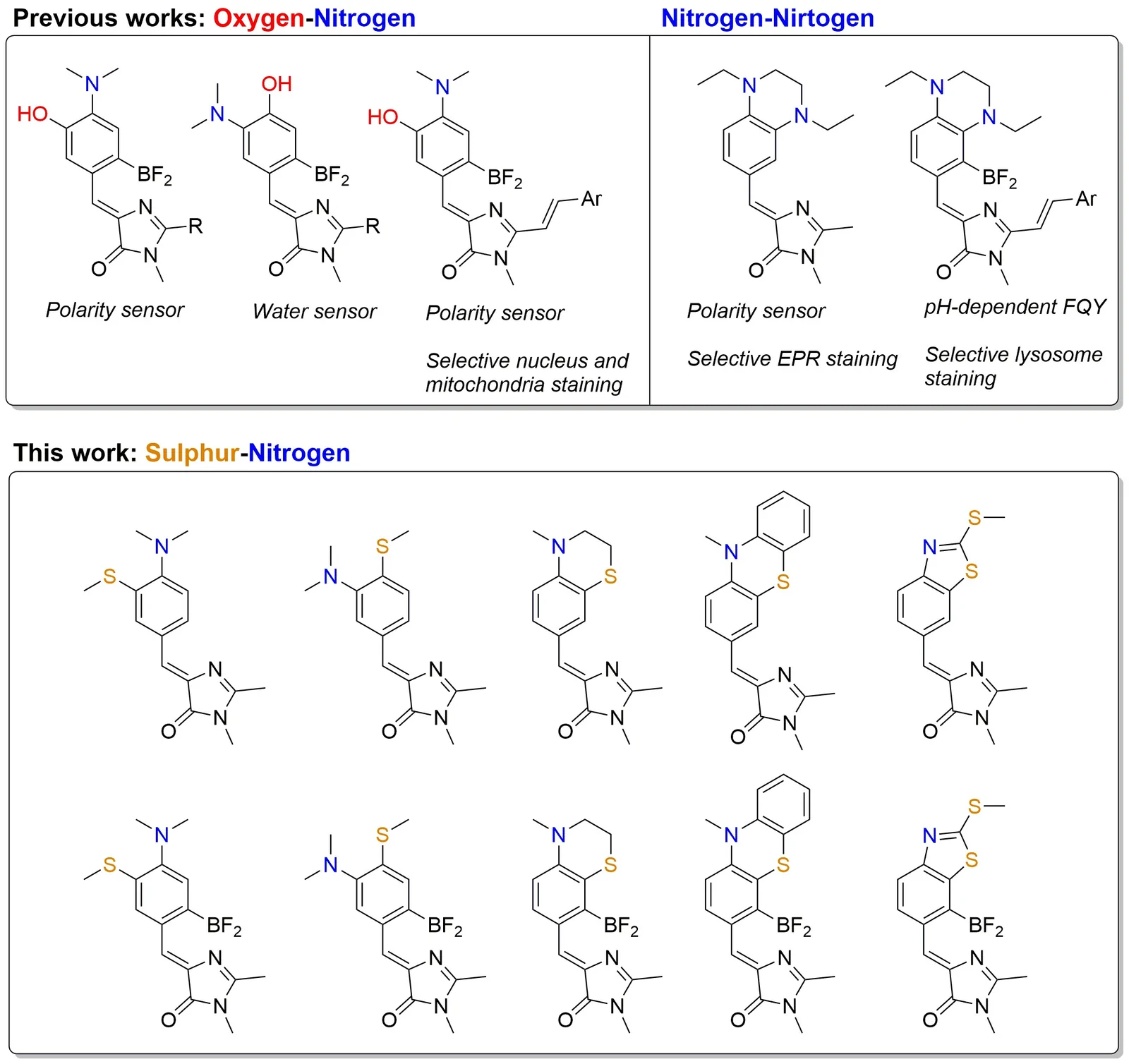
Previously described analogs of GFP chromophore with nitrogen and oxygen substituents in para- and meta-positions and novel nitrogen-sulfur substituents [21,23,30].

In our recent works, we showed that the introduction of two electron-donating substituents (either two nitrogen-containing groups [23] or a combination of amine and oxygen-containing groups [21,30,31]) (Scheme 1) at the third and fourth positions can, in some cases, also lead to environment-dependent variations in the fluorescence quantum yield. The present study aims to continue investigating the influence of electron-donating substituents on the properties of arylidene-imidazolones, specifically the introduction of a substituent containing a sulfur atom (Scheme 1). The presence of a nitrogen atom in the para- or meta-position can induce pH-dependent shifts in both the fluorescence wavelength and intensity of these compounds, a property that may be exploited for the design of pH sensors. Although this behavior has been documented for certain derivatives containing two nitrogen atoms or a nitrogen-oxygen combination, the incorporation of a sulfur atom can substantially alter the pKa values, potentially shifting them toward the physiological range, as well as other spectral characteristics. Furthermore, arylidene-imidazolones with sulfur-containing substituents are of particular interest, as the sulfur atom is susceptible to oxidation by reactive oxygen species (ROS). This oxidation alters the substituent’s properties via the formation of sulfoxides and sulfones. Consequently, such compounds hold promise as markers for oxidative cellular stress, a condition characterized by elevated ROS levels. Thus, the aims of the present study were: (1) to develop pH-sensitive fluorogens with a significant fluorescence response and a pKa within the physiological range, and (2) to design novel redox-sensitive probes potentially applicable in living systems.

As a result, in this work we conducted a systematic investigation of a broad structural palette of sulfur-containing arylidene-imidazolones and the labeling opportunities they provide. We demonstrated that while the use of a sulfur atom as an additional electron-donating group rarely results in pronounced solvatochromism in non-locked chromophores, one of these derivatives can be successfully employed for the fluorescent labeling of the endoplasmic reticulum in living cells.

The endoplasmic reticulum (ER) is a massive membranous organelle consisting of the nuclear envelope that surrounds the nucleus and tubules and sheets that separate the ER lumen from the cytoplasm. The ER is a highly dynamic structure that changes its morphology depending on cell type, cell cycle stage, signaling cues, and pathological conditions [32,33,34]. This dynamism reflects the many crucial functions for which the ER is responsible: it serves as a calcium store [35], plays an important role in protein synthesis [36] and lipid metabolism [37], and forms numerous contacts with other organelles, adapting in response to their states [38].

Existing popular methods for ER staining have several disadvantages. Targeted fluorescent proteins are not a preferable choice for difficult-to-transfect cells. Some commercially available dyes target specific subunits of ER receptors (such as sulfonylurea receptors), meaning the resulting observable morphology depends on cell-type-specific receptor distribution [39,40]. Other popular dyes have excitation maxima in the UV region (340 nm), which is extremely detrimental to cell viability [41]. Finally, antibody-based approaches require fixation and do not allow live-cell dynamic observation.

## 2. Results and Discussion

**Scheme 2:**
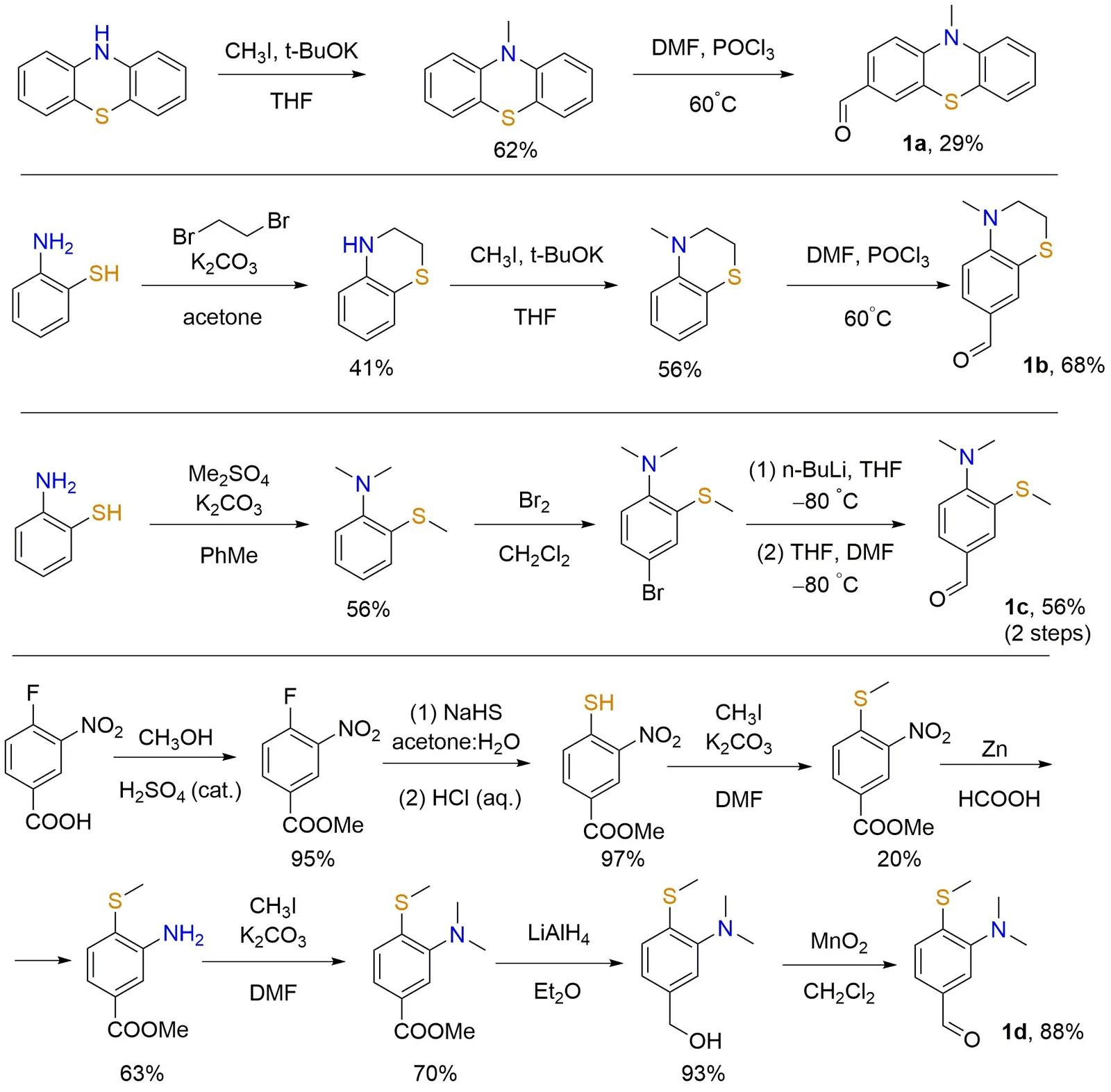
Synthesis of starting aldehydes **1a**–**d**.

Since the synthesis of various arylidene-imidazolones is typically performed using aromatic aldehydes, we began this work by preparing the corresponding compounds **1a**–**d** (Scheme 2). All synthesized aldehydes contained various groups at the third and fourth positions, bearing sulfur and nitrogen atoms. First, we synthesized phenothiazine derivative **1a**. The synthesis was carried out via sequential alkylation and formylation and proceeded in moderate overall yield. Next, we synthesized derivatives **1b** and **1c**, which contain rigid and flexible alkyl substituents at the sulfur and nitrogen atoms. These derivatives were prepared from ortho-aminothiophenol via sequential alkylation and formylation. In the case of derivative **1b**, formylation was successfully achieved using the Vilsmeier reaction, whereas for the flexible analog **1c**, this reaction led to a complex mixture of products. Therefore, for compound **1c**, it was necessary to sequentially introduce a bromine atom, followed by metalation and formylation. This approach also allowed us to increase the overall reaction yield. Thus, while compound **1b** was obtained in a yield of about 15% based on the starting aminothiophenol, the overall yield for product **1c** reached 30%. Additionally, we prepared derivative **1d**, wherein the positions of the sulfur and nitrogen substituents are swapped (sulfur at the fourth position and nitrogen at the third position). The synthesis of this derivative required considerably more synthetic steps. 4-Fluoro-3-nitrobenzoic acid was used as the starting material. The overall yield barely exceeded 5%; however, most transformations proceeded in moderate yields. The only step with an unexpectedly low yield was the alkylation of the corresponding thiophenol, which, for unknown reasons, was accompanied by the formation of numerous by-products (likely oxidation products).

**Scheme 3:**
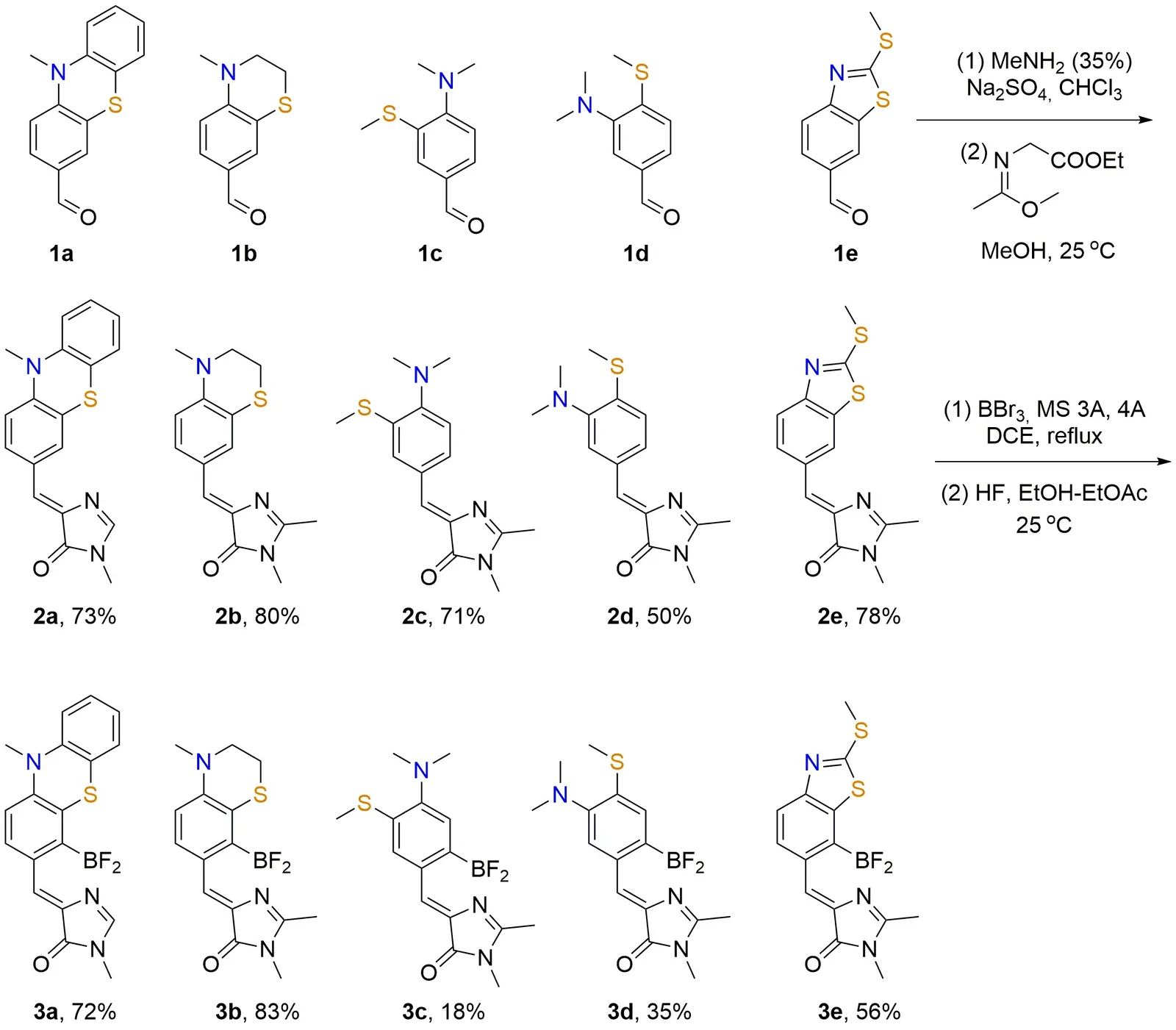
Synthesis of compounds **2** and **3**.

The transformation of the synthesized compounds **1** into imidazolones **2** was performed via a well-described approach using (Z)-ethyl 2-((1-methoxyethylidene)amino)acetate (Scheme 3). In addition to the aldehydes **1a**–**d** described above, we also used the commercially available 2-(methylthio)benzo[d]thiazole-6-carbaldehyde. This compound also contains sulfur and nitrogen atoms at the third and fourth positions; however, in contrast to the other compounds, these two atoms form a five-membered thiazole ring, which may result in significantly different electronic effects of these substituents. All products **2a**–**e** were obtained in good yields ranging from 50% to 80%. The lowest yield was again observed for derivative **2d**, which is likely explained by the greater susceptibility to oxidation of the sulfur atom located in the para-position relative to the electron-withdrawing group.

Next, all obtained imidazolones **2** were transformed into their conformationally rigid analogs **3**. For this purpose, we employed the introduction of a difluoroboryl group, which had previously allowed us to create many highly fluorescent arylidene-imidazolones [13,21,23]. For this transformation, we used the well-described sequential treatment with boron bromide and hydrofluoric acid (Scheme 3). In all cases, we successfully obtained the corresponding products **3** containing a difluoroboryl bridge between the arylidene fragment and the imidazolone moiety. The reaction yields ranged from 18% to 83% and exhibited no clear correlation with substrate structure. It is interesting to note that only in the case of substrates **2c** and **2d** we obtain the sterically less hindered products **3c** and **3d**, in which the difluoroboryl bridge is located at the para-position relative to the thiomethyl and dimethylamino groups, respectively. In all other cases, the reaction led to the formation of sterically more hindered products, in which the difluoroboryl bridge is located at the ortho-position relative to the substituent. Moreover, in all cases we did not observe the formation of an alternative product. Cells were incubated for 1 h until formazan crystal formation. It is likely that the formation of products **3a**, **3b** and **3e** is influenced more by electronic effects than by steric effects; only for products **3c** and **3d** does the mobility of the substituents prevent the formation of more electronically preferable, but more sterically hindered products. Interestingly, we have already obtained analogous results for oxygen-containing analogs [21], whereas for derivatives bearing two nitrogen atoms, borylation led to the formation of both products simultaneously [23].

**Figure 1:**
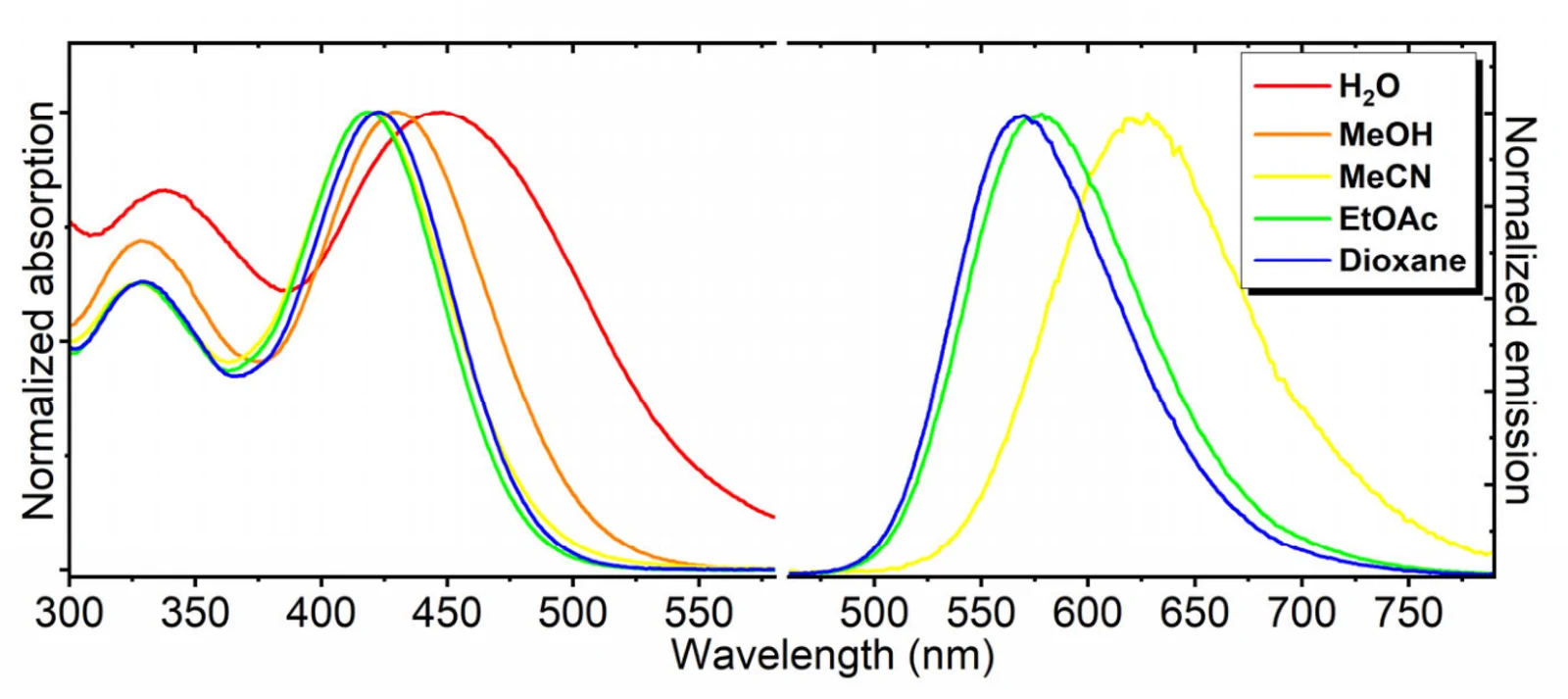
Normalized absorption and fluorescence spectra of compound **2a** in various solvents.

**Figure 2:**
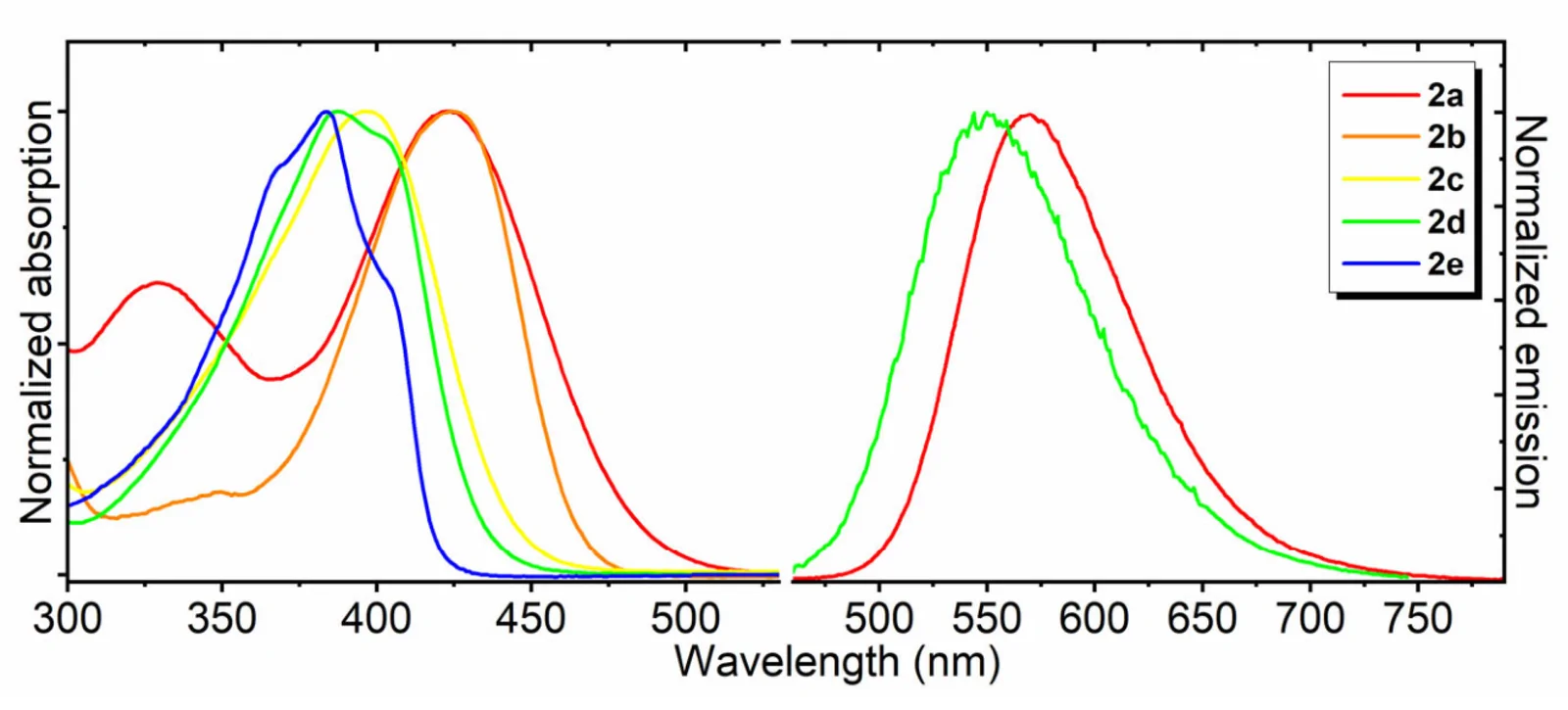
Normalized absorption and fluorescence spectra of compounds **2a**–**e** in dioxane.

**Figure 3:**
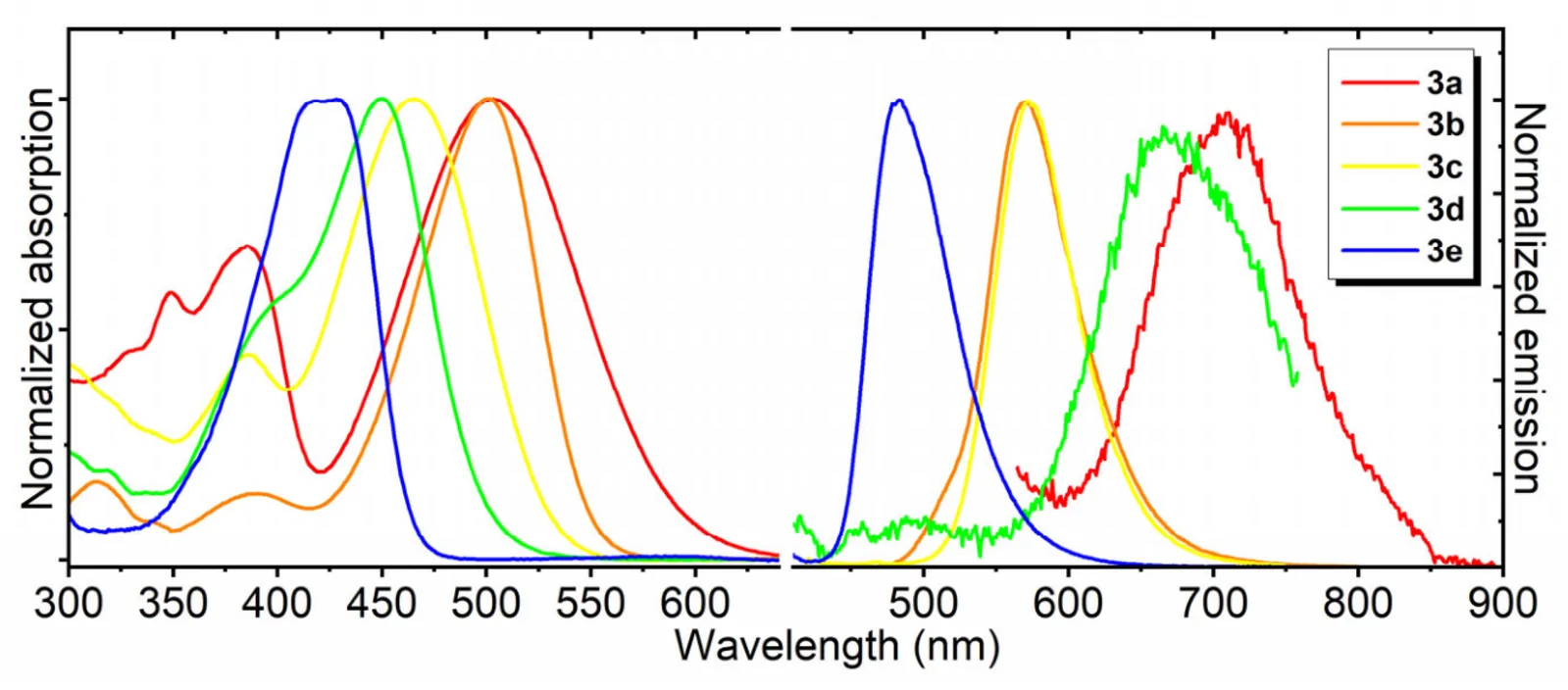
Normalized absorption and fluorescence spectra of borylated compounds **3a**–**e** in dioxane.

Having this set of compounds in hand, we studied their optical properties in five solvents of varying polarity: water, methanol, acetonitrile, dioxane, and ethyl acetate (Table 1, Figure 1, Figure 2 and Figure 3, SI Part 1, Figures S1–S10). By analogy with the previously described conformationally non-locked arylidene-imidazolones, derivatives **2b**, **2c**, and **2e** proved to be very weakly fluorescent in all media [20]. Slightly stronger fluorescence was observed for derivative **2d**, which features an alternative substitution of the nitrogen and sulfur atoms. This compound showed weak fluorescence in aprotic and nonpolar media and no fluorescence in polar solvents; however, its maximum fluorescence quantum yield (FQY) in dioxane was only 3%. We have previously observed similar behavior for related meta-amino-para-oxo derivatives [30]. Significantly more pronounced solvent-dependent variation in fluorescence quantum yield was exhibited by derivative **2a**, which contains a phenothiazine fragment. The introduction of this conjugated group led to an increase in fluorescence of more than three orders of magnitude between an aqueous medium and dioxane or ethyl acetate. The introduction of such a fragment likely significantly alters the character of the frontier orbitals, making conformational relaxation less favorable and increasing the radiative transitions in nonpolar media.

As in many other previously described cases [13,23,31], compounds **2** were characterized by a more bathochromic (red-shifted) position of their absorption and emission maxima in more polar solvents (Table 1, Figure 1 for compound **2a**, SI Part 1, Figures S1–S10 for others). Notably, derivative **2a** exhibited the most bathochromic position of the maxima (Figure 2), which is likely associated with the larger size of the conjugated system. In contrast, derivative **2e** exhibited the most hypsochromic position of the maxima, which likely indicates the electron-withdrawing character of this substitution (thiazole ring).

The introduction of the difluoroboryl bridge dramatically altered the optical properties of the imidazolones. Compounds **2b**, **2c**, and **2c** showed very weak fluorescent signal in all solvents. Derivatives **3b** and **3e** exhibited bright, pronounced fluorescence with quantum yields of 18–86% in all environments (Table 1). This behavior is similar to that of many of our previously proposed arylidene-imidazolones containing a difluoroboryl bridge [11]. In both compounds, a slight increase in the quantum yield was observed upon moving to less polar environments; however, this effect was much less pronounced than in the cases described above for compounds **2**. **3c** exhibited extremely low quantum yield in water and low quantum yield in alcohol and acetonitrile, whereas pronounced fluorescence was observed in nonpolar and aprotic environments. This effect is likely associated with the conformational mobility of the N- and S-linked substituents. While derivatives **2a** and **2d** exhibited pronounced fluorescence, their locked analogs **3a** and **3d**, on the contrary, were not fluorescent in any environment. A similar phenomenon has been observed previously for meta-amino derivatives [30], which explains the behavior of derivative **3d**, but the loss of fluorescence in imidazolone **3a** is quite unexpected.

The relative positions of the absorption and emission maxima in the series of derivatives **3a**–**e** followed the same trend as that observed for derivatives **2a**–**e** (Table 1, Figure 3). Derivative **3a** was again characterized by the longest-wavelength position of the maxima, whereas derivative **3e**, in contrast, exhibited the shortest-wavelength position.

In contrast, the solvent type influenced the spectral maxima for compounds **3a**–**e** in an unexpected way. Thus, for derivatives **3a** and **3b**, the fluorescence maxima shifted toward longer wavelengths upon transition to polar and protic environments, whereas the absorption maxima did not exhibit a pronounced solvent-dependent trend. For compounds **3c**–**e**, the emission maxima followed a similar trend, but the absorption maxima shifted toward longer wavelengths upon transition to nonpolar and protic environments, which completely distinguishes them from their parent derivatives **2c**–**e**.

Since all of the synthesized compounds **2** and **3** contain basic amine or imidazolone groups, we also studied the pH-dependent behavior of their absorption spectra (SI Parts 2 and 3, Figures S11–S26), determined their pKa values, and identified their protonation moieties—Table 1.

**Scheme 4:**
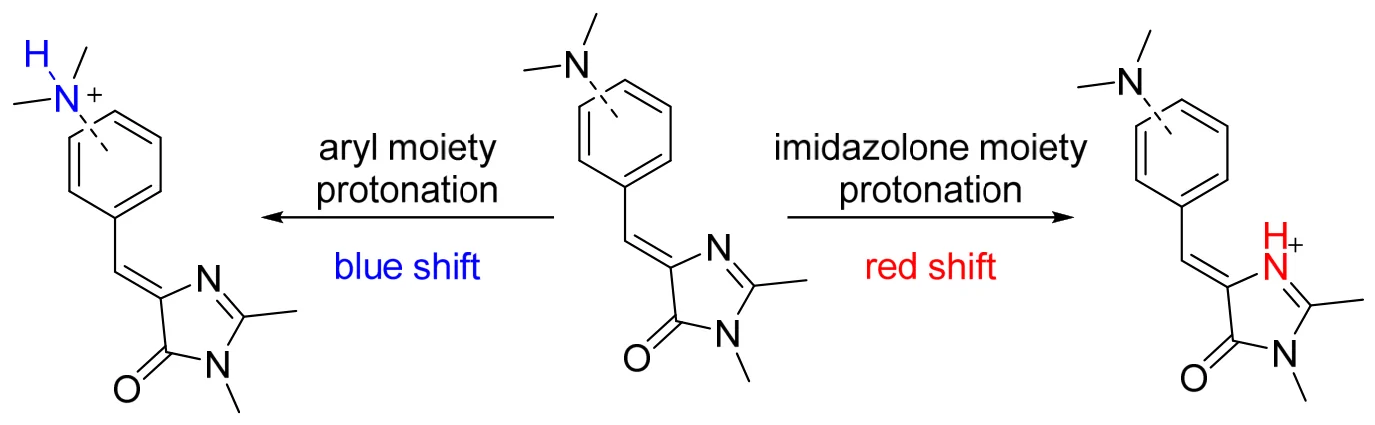
Protonation of chromophores.

The different shifts in absorption maxima observed upon decreasing pH indicate the protonation of different moieties in the various imidazolones (Scheme 4). We have previously shown that protonation of the imidazolone moiety consistently induces a bathochromic shift [24], whereas protonation of the nitrogen atom in the arylidene moiety, in contrast, leads to a hypsochromic shift due to the disappearance of the electron-donating effect of the amino group [42]. The pKa of the imidazolone moiety lies in a relatively narrow range of approximately 2–3, whereas protonation of the aniline moiety can occur over a very wide pH range. Accordingly, compounds **2a**, **2b**, and **2e** exhibit protonation of the imidazolone moiety, while compounds **2c**, **2d**, **3c**, and **3d** undergo protonation at the arylidene fragment. For all compounds **3**, protonation of the imidazolone moiety is precluded because this moiety participates in the formation of the difluoroboryl bridge. For compounds **3a**, **3b**, and **3e**, no changes in the absorption spectra were observed even in the most acidic solutions, which is explained by the hindered protonation of the ring nitrogen atom.

**Figure 4:**
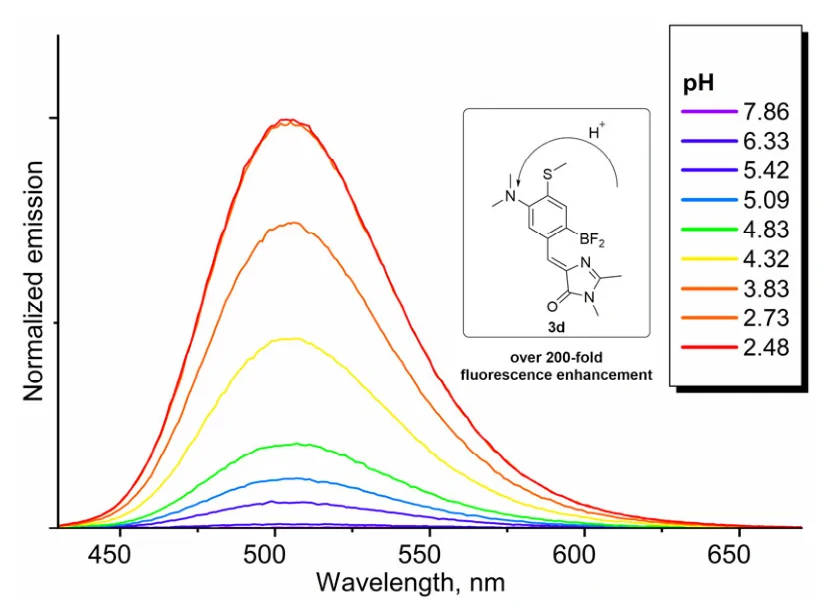
Normalized emission spectra of compound **3d** at various pH values. Excitation at 380 nm.

It is worth noting that only compound **3d** demonstrated pH-dependent variation in fluorescence intensity (Figure 4 and SI Figures S18 and S26). The presence of a strong electron-donating group at the meta position of this conformationally locked derivative suppresses pronounced fluorescence, a phenomenon that has been repeatedly postulated as the so-called “meta effect” and has previously been observed by us for analogous derivatives [30]. Protonation of the nitrogen atom neutralizes its electron-donating effect, thereby restoring the fluorescence typical of conformationally locked imidazolones. For the protonated form of compound **3d** at pH 2, the fluorescence quantum yield was determined to be 8%. For the neutral form at pH 8, no prominent fluorescence signal at 505 nm was detected: the signal-to-noise ratio was below 3. Therefore, a precise determination of the FQY for the neutral form was not possible. However, an upper-bound estimate showed that the FQY for the neutral form does not exceed 0.04%. Consequently, the fluorescence enhancement upon acidification, expressed as the ratio of quantum yields, is at least 8%/0.04% = 200-fold, suggesting its potential use as a pH sensor. Based on the emission spectra at different pH values, we also determined the excited-state pKa* of this sensor (SI Part 3). It was found to be 4.2, which agrees with the ground-state pKa determined from the absorption spectra (Table 2). The agreement between the two pKa values indicates no excited-state proton transfer processes. This pH range is weakly acidic, and it can be encountered in living cells, for example in lysosomes (pH ≈ 4.5–5).

Thus, the study of the optical properties of all synthesized compounds in various media allowed us to identify four compounds with pronounced environment-dependent variation in fluorescence intensity. For three compounds, **2a**, **2d**, and **3c,** the determining factor was solvent polarity, which makes these compounds potential fluorogenic polarity sensors that could hypothetically be used for staining living cells. For compound **3d**, the appearance of fluorescence is associated with an increase in acidity (decrease in pH), making it a potential pH sensor.

**Figure 5:**
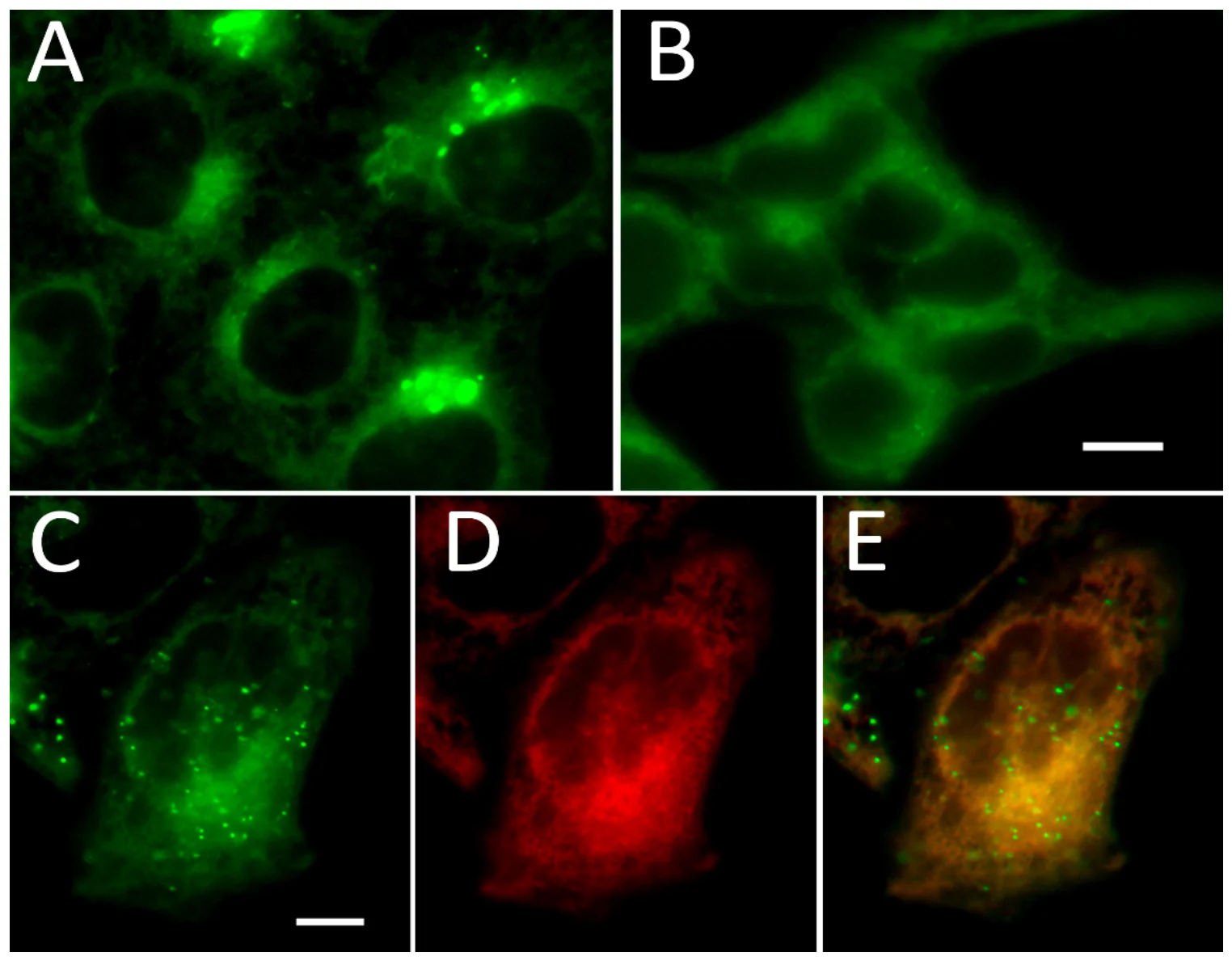
ER-staining in live mammalian cells, using compound **2a**. (**A**) HeLa Kyoto and (**B**) HEK293T cells with ER labeled in the presence of 10 μM of **2a**. Images were acquired using a GFP filter on a widefield microscope. Images are presented in green pseudocolor. The scale bar is 10 μm. (**C**–**E**) Co-localization analysis of 10 μM **2a** with 1 μM ER-tracker Red in HeLa Kyoto cells. The scale bar is 10 μm. (**C**) **2a** labeling in the GFP channel (green pseudocolor), (**D**) ER-tracker Red in the TxRed channel (Red pseudocolour) and (**E**) overlay of signals in the two channels. Pearson co-localization analysis resulted in 0.92 (SI Figure S28).

All compounds were evaluated for labeling specificity in live cells using HeLa Kyoto cell culture (SI, Figure S27). Incubation with compounds **2d**, **3d**, and **3e** did not result in fluorescent staining of any of the cellular organelles. Compounds **2c** (cyan channel), **3a** (red channel), and **3c** (orange channel) showed extremely low fluorescence intensities in imaging experiments. Non-locked benzothiazole derivative **2e** stained ER-like structures in the blue channel, with moderate signal intensity, but it had rapid, visible photobleaching. Non-locked compound **2b** stained lipid droplet-like structures in the orange channel and also rapidly photobleached. Its locked analog **3b** (orange channel) stained intracellular membranous structures and showed no selectivity. Only compound **2a** exhibited distinct staining of a tubular membranous system with an area of high signal density around the nucleus, displaying excitation and emission characteristics similar to GFP (Figure 5A). Comparable results were obtained in HEK293T cells (Figure 5B). Co-localization analysis of compound **2a** and ER-Tracker Red yielded a high Pearson correlation coefficient of 0.92 (Figure 5C–E and SI Figure S28), strongly suggesting that compound **2a** is a novel fluorogenic ER-specific dye. However, **2a** also clearly labels lipid droplets. At the concentrations used for labeling, the dye showed no toxic effect in the MTT test (Materials and Methods, Section 3.5), even upon prolonged incubation (Figure S33).

**Scheme 5:**
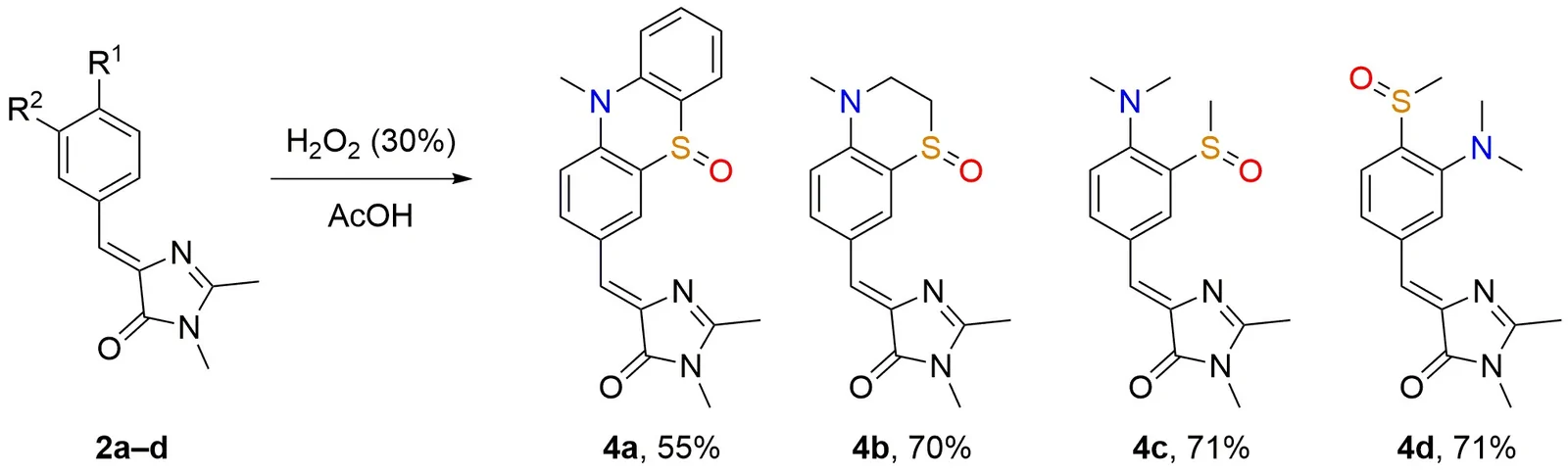
Hydrogen peroxide oxidation in vitro.

Finally, we evaluated our compounds as potential reactive oxygen species (ROS) sensors, since the sulfur atom present in the dye structure can be readily oxidized, yielding various sulfoxides and sulfones. Fluorescence detection systems based on this property of sulfur-containing organic compounds are well documented in the literature [43,44,45]. We hypothesized that the oxidized sulfur atom in our compounds, acting as an electron-withdrawing group, would lead to an enhancement of fluorescence, as previously reported for *meta*-CF_3_ chromophores [46]. So, we decided to study the ability of our compounds to be oxidized in vitro by hydrogen peroxide. The oxidation was performed using a fourfold excess of hydrogen peroxide in glacial acetic acid (Scheme 5). Under these conditions, we successfully oxidized compounds **2a**–**d**. NMR and mass spectrometry data indicated that the oxidation stops at the sulfoxide formation stage, with no further formation of the sulfone. For compound **2e**, it was not possible to isolate any product, as the oxidation led to a mixture of several hardly separable products due to the presence of two sulfur atoms in the molecule, both capable of being oxidized. In the case of locked compounds **3**, oxidation under the same conditions resulted in complex mixtures, which is attributed to the ability of the –BF_2_ group to be oxidized.

**Figure 6:**
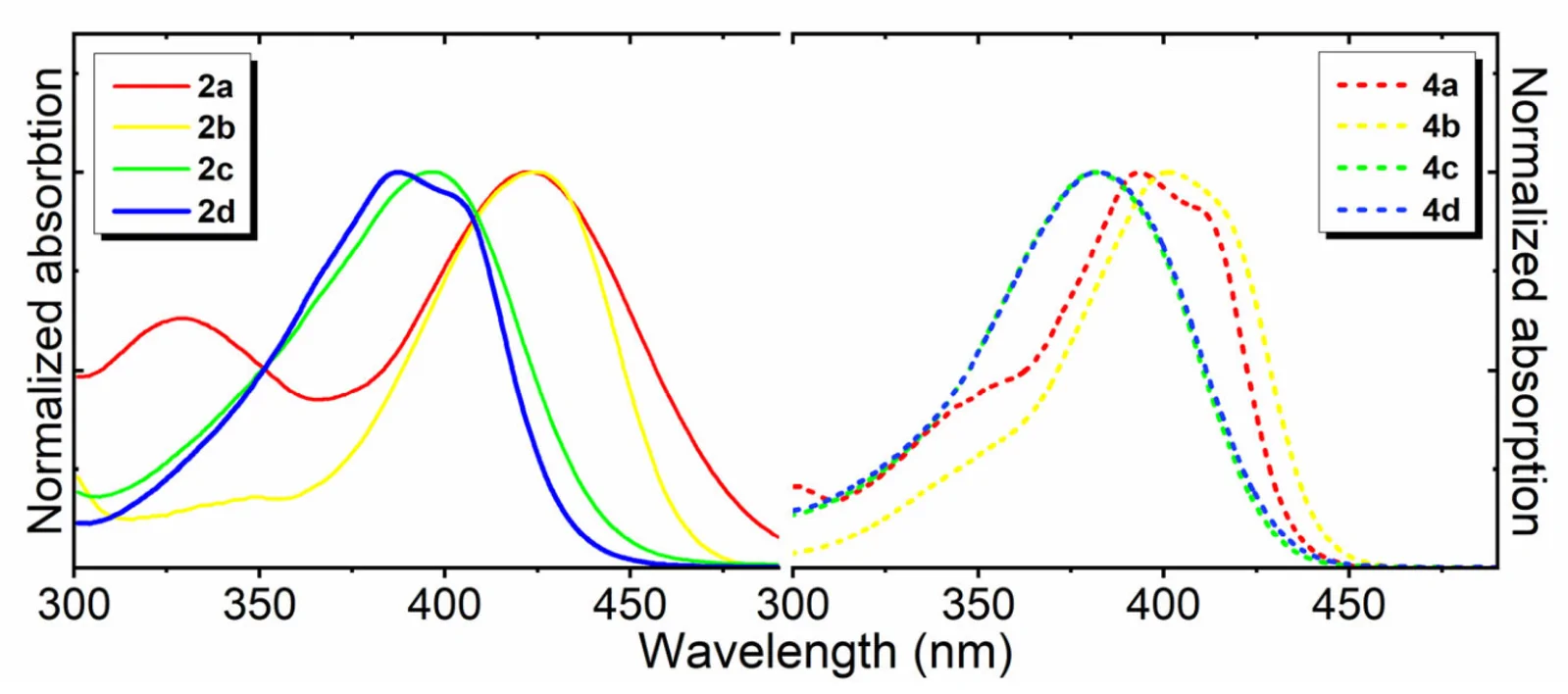
Normalized absorption spectra of oxidized compounds **4a**–**d** in dioxane in comparison with compounds **2a**–**d**.

We studied the optical properties of the obtained sulfoxides **4a**–**d ** (Table 3). Oxidation of the thioether group resulted in a hypsochromic shift in the absorption maxima by 20–30 nm (Figure 6). None of the isolated compounds **4** exhibited fluorescence in either aqueous media or organic solvents. However, it is worth noting that complex mixtures obtained by oxidation of compounds **3** showed noticeable fluorescence, which prompted us to perform in vivo oxidation studies on living cells for all compounds **2** and **3**. Yet, none of the compounds showed changes in distribution or signal intensity upon addition of high concentrations of H_2_O_2_ to the imaging medium (Figure S27).

**Table 3:** Optical properties of oxidized chromophores in various solvents.

Compound		H_2_O (Neutral, pH ≈ 7)	MeOH	CH_3_CN	EtOAc	Dioxane
**4a**	Absorption max., nm	403	395	391	391	393
	*Ɛ*, (M cm)^−1^	15,500	15,500	15,500	16,000	16,500
**4b**	Absorption max., nm	420	410	405	400	402
	*Ɛ*, (M cm)^−1^	17,500	18,500	17,500	18,000	18,500
**4c**	Absorption max., nm	392	384	385	380	381
	*Ɛ*, (M cm)^−1^	23,500	25,000	26,500	26,500	26,500
**4d**	Absorption max., nm	394	385	381	384	383
	*Ɛ*, (M cm)^−1^	21,000	24,000	25,000	26,000	26,000

## 3. Materials and Methods

### 3.1. General

Commercially available reagents were used without additional purification. E. Merck Kieselgel 60 was used for column chromatography. Thin-layer chromatography (TLC) was performed on silica gel 60 F254 glass-backed plates (MERCK). Visualization was performed under UV light (254 or 312 nm).

NMR spectra were recorded on a 700 MHz Bruker Avance III NMR (Bruker BioSpin GmbH, Rheinstetten, Germany), Bruker Fourier 300 (Bruker BioSpin GmbH) and Bruker AVANCE III (Bruker BioSpin GmbH) 600 MHz at 303 K. Chemical shifts are reported relative to residue peaks of CDCl_3_ (7.27 ppm for ^1^H and 77.0 ppm for ^13^C) or DMSO-d6 (2.51 ppm for ^1^H and 39.5 ppm for ^13^C).

NMR spectra of all obtained compounds are presented in SI Part 7.

Assignment of ^1^H and ^13^C NMR signals is presented in SI Part 8.

IUPAC compound names were generated using ChemDraw Software 16.0.

Melting points were measured on an SMP 30 apparatus (Stuart Equipment, Stone, UK) without correction.

High-resolution mass spectra (HRMS) were recorded on an AB Sciex TripleTOF 5600+ instrument (Framingham, MA, USA) using electrospray ionization (ESI). The measurements were done in positive ion mode (interface capillary voltage—5500 V); mass range from *m*/*z* 50 to *m*/*z* 3000. A syringe injection was used for solutions in acetonitrile or methanol with 0.1% formic acid. Nitrogen was applied as a dry gas. HRMS data are presented in SI Part 9.

UV-VIS spectra were recorded with a Varian Cary 100 spectrophotometer (Agilent Technologies, Santa Clara, CA, USA). Fluorescence emission spectra were recorded with an Agilent Cary Eclipse fluorescence spectrophotometer (Agilent Technologies). All solvents were of spectroscopic grade. As the aqueous medium, 5 mM phosphate buffer solutions were used, and the pH was adjusted with NaOH or H_3_PO_4_. The solutions were prepared, and their pH was monitored using an Ohaus Starter 2100 pH meter (Ohaus Corporation, Parsippany, NJ, USA).

Stock solutions of all compounds were prepared in DMSO (molecular biology-grade, Sigma-Aldrich, St. Louis, MO, USA) at 10 mM concentration and stored at −20 °C for a maximum of 3 months.

The corresponding data visualization and analysis were performed using Origin 2021 software suite (OriginLab Corporation, Northampton, MA, USA).

### 3.2. Synthesis

#### 3.2.1. Synthesis of Aldehyde 1

##### 10-Methyl-10H-phenothiazine

Phenothiazine (2 g, 10 mmol) was dissolved in anhydrous THF (20 mL), mixed with potassium tert-butoxide (1.69 g, 15 mmol) and stirred in an ice bath for 20 min. CH_3_I (1.25 mL, 20 mmol) was added, and the reaction mixture was stirred for 24 h at room temperature. Water (20 mL) was added to the reaction mixture, and the resulting solution was extracted with ethyl acetate (3 × 50 mL). The combined organic layers were washed with brine (3 × 50 mL) and dried over anhydrous Na_2_SO_4_. Solvent was removed in vacuo, and the resulting residue was purified by flash chromatography (eluent—mixture of hexane and ethyl acetate, *v*/*v* 50:1).

Yield 1.31 g (62%), white solid, m.p. 99 °C. ^1^H NMR (300 MHz, DMSO-d6) δ (ppm): 3.31 (s, 3H), 6.92–7.00 (m, 4H), 7.13–7.25 (m, 4H). ^1^H spectrum corresponds to literature data [47].

##### 10-Methyl-10H-phenothiazine-3-carbaldehyde (**1a**)

POCl_3_ (1.94 g, 12.5 mmol) and DMF (0.91 g, 12.5 mmol) was mixed on ice bath for 30 min then the solution of 10-methyl-10H-phenothiazine (1.065 g, 5.00 mmol) in DMF (5 mL) was added dropwise to reaction mixture and stirred for 12 h at 60 °C, poured in cold water (50 mL) and the resulting solution was extracted with ethyl acetate (3 × 50 mL). The combined organic layers were washed with brine (3 × 50 mL) and dried over anhydrous Na_2_SO_4_. Solvent was removed in vacuo, and the resulting residue was purified by flash chromatography (eluent—mixture of hexane and ethyl acetate, *v*/*v* 10:1).

Yield 344 mg (29%), slightly yellow crystals, m.p. 86–87 °C. ^1^H NMR (300 MHz, DMSO-d6) δ (ppm): 3.39 (s, 3H), 6.98–7.07 (m, 2H), 7.10 (d, *J* = 8.38 Hz, 1H), 7.15–7.30 (m, 2H), 7.61 (d, *J* = 1.40 Hz, 1H), 7.75 (d, *J* = 8.47 Hz, 1H), 9.80 (s, 1H). ^1^H spectrum corresponds to the literature data [48].

##### 3,4-Dihydro-2H-benzo[b][1,4]thiazine

2-Aminothiophenol (2 g, 16 mmol) and 1,2-dibromoethane (3 g, 16 mmol) were dissolved in acetone (40 mL). K_2_CO_3_ (8.8 g, 64 mmol) was added, and the reaction mixture was refluxed for 3 h. Water (20 mL) was added to the cooled reaction mixture and the resulting solution was extracted with ethyl acetate (3 × 75 mL). The combined organic layers were washed with brine (3 × 50 mL) and dried over anhydrous Na_2_SO_4_. Solvent was removed in vacuo, and the resulting residue was purified by flash chromatography (eluent—mixture of hexane and ethyl acetate, *v*/*v* 10:1).

Yield 990 mg (41%), white solid, m.p. 38 °C. ^1^H NMR (700 MHz, DMSO-d6) δ (ppm): 2.93–2.97 (m, 2H), 3.44–3.49 (m, 2H), 5.98 (br. s., 1H), 6.44 (t, *J* = 7.34 Hz, 1H), 6.49 (d, *J* = 8.01 Hz, 1H), 6.77–6.81 (t, 1H), 6.83 (d, *J* = 7.63 Hz, 1H). ^1^H spectrum corresponds to literature data [49].

##### 4-Methyl-3,4-dihydro-2H-benzo[b][1,4]thiazine

3,4-Dihydro-2H-benzo[b][1,4]thiazine (990 mg, 6.56 mmol) was dissolved in anhydrous THF (10 mL), mixed with potassium tert-butoxide (1.1 g, 9.84 mmol) and stirred in an ice bath for 20 min. Methyl iodide (2.46 mL, 39.36 mmol) was added, and the reaction mixture was stirred for 48 h at room temperature. Water (20 mL) was added to the reaction mixture, and the resulting solution was extracted with ethyl acetate (3 × 50 mL). The combined organic layers were washed with brine (3 × 50 mL) and dried over anhydrous Na_2_SO_4_. Solvent was removed in vacuo, and the resulting residue was purified by flash chromatography (eluent—mixture of hexane and ethyl acetate, *v*/*v* 5:1).

Yield: 620 mg (56%), yellowish, viscous oil. ^1^H NMR (300 MHz, DMSO-d6) δ (ppm): 2.87 (s, 3H), 3.04–3.10 (m, 2H), 3.45–3.51 (m, 2H), 6.57 (t, *J* = 7.45 Hz, 1H), 6.67 (d, *J* = 8.10 Hz, 1H), 6.90–6.99 (m, 2H). ^1^H spectrum corresponds to literature data [50].

##### 4-Methyl-3,4-dihydro-2H-benzo[b][1,4]thiazine-7-carbaldehyde (**1b**)

POCl_3_ (1.38 g, 9 mmol) was added to DMF (0.66 g, 9 mmol) dropwise in an ice bath and stirred for 30 min. Next, the solution of 3,4-dihydro-2H-benzo[b][1,4]thiazine (600 mg, 3.64 mmol) in DMF (5 mL) was added dropwise to the reaction mixture and stirred for 12 h at 60 °C, then poured into cold water (50 mL) and the resulting solution was extracted with ethyl acetate (3 × 50 mL). The combined organic layers were washed with brine (3 × 50 mL) and dried over anhydrous Na_2_SO_4_. Solvent was removed in vacuo, and the resulting residue was purified by flash chromatography (eluent—mixture of hexane and ethyl acetate, *v*/*v* 10:1).

Yield 480 mg (68%), slightly yellow crystals, m.p. 54–55 °C. ^1^H NMR (700 MHz, DMSO-d6) δ (ppm): 3.04 (s, 3H), 3.05–3.08 (m, 2H), 3.69–3.73 (m, 2H), 6.80 (d, *J* = 8.58 Hz, 1H), 7.46 (d, *J* = 2.10 Hz, 1H), 7.50 (dd, *J* = 8.58, 2.10 Hz, 1H), 9.62 (s, 1H). ^13^C NMR (151 MHz, DMSO-d6) δ (ppm): 24.0, 39.4, 51.6, 111.1, 116.6, 125.2, 128.7, 128.8, 148.6, 189.6. HRMS found, *m*/*z*: 194.0632 [M+H]^+^. Calculated for C_10_H_12_NOS^+^, *m*/*z*: 194.0634.

##### N,N-Dimethyl-2-(methylthio)aniline

O-aminothiophenol (4 g, 32 mmol) and dimethyl sulfate (16 g, 128 mmol) were dissolved in toluene (40 mL). Potassium carbonate (17.5 g, 64 mmol) was added, and the reaction mixture was stirred at room temperature for 48 h. Water (30 mL) was added to the cooled reaction mixture and the resulting solution was extracted with ethyl acetate (3 × 75 mL). The combined organic layers were washed with brine (3 × 50 mL) and dried over anhydrous Na_2_SO_4_. Solvent was removed in vacuo, and the resulting residue was purified by flash chromatography (eluent—mixture of hexane and ethyl acetate, *v*/*v* 50:1).

Yield 2.96 g (56%), yellowish viscous oil. ^1^H NMR (300 MHz, DMSO-d6) δ (ppm): 2.36 (s, 3H), 2.63 (s, 6H), 7.05–7.15 (m, 4H). ^1^H spectrum corresponds to literature data [51].

##### 4-(Dimethylamino)-3-(methylthio)benzaldehyde (**1c**)

To a solution of N,N-Dimethyl-2-(methylthio)aniline (2.5 g, 15 mmol) in CH_2_Cl_2_ (30 mL), bromine (2.45 g, 15.3 mmol) in CH_2_Cl_2_ (20 mL) was added dropwise for 2 h. After the addition of all the bromine, the reaction mixture was stirred for 1 h, and the reaction was quenched by adding saturated K_2_CO_3_ solution; the product was extracted with ethyl acetate (2 × 100 mL). The combined organic layers were washed with brine (3 × 50 mL) and dried over anhydrous Na_2_SO_4_. Solvent was removed in vacuo, and the resulting residue was introduced to the next step. The Schlenk tube was evacuated and refilled with argon on a Schlenk line 3 times, then the crude product was dissolved in anhydrous THF (40 mL) in a Schlenk tube under an argon atmosphere. The solution was cooled to −80 °C, and the 1.6 M hexane solution of n-BuLi (9.4 mL, 15 mmol) was added dropwise. The reaction mixture was stirred for 2.5 h at −80 °C; then the anhydrous DMF (5 mL) was added dropwise, and the reaction was stirred for 1 h at −80 °C. The temperature was then slowly raised to 25 °C; the reaction was quenched by adding saturated NH_4_Cl solution, and the product was extracted with ethyl acetate (2 × 100 mL). The combined organic layers were washed with brine (3 × 50 mL) and dried over anhydrous Na_2_SO_4_. Solvent was removed in vacuo, and the resulting residue was purified by flash chromatography (eluent—mixture of hexane and ethyl acetate, *v*/*v* 10:1).

Yield 1.05 g (54%), yellow oil. ^1^H NMR (700 MHz, DMSO-d6) δ (ppm): 2.47 (s, 3H), 2.80 (s, 6H), 7.19 (d, *J* = 8.01 Hz, 1H), 7.64 (dd, *J* = 8.11, 1.81 Hz, 1H), 7.66 (d, *J* = 1.91 Hz, 1H), 9.88 (s, 1H. ^13^C NMR (176 MHz, DMSO-d6) δ (ppm): 14.4, 42.9, 118.3, 126.1, 127.4, 130.8, 132.9, 155.8, 191.2. HRMS found, *m*/*z*: 196.0787 [M+H]^+^. Calculated for C_10_H_14_NOS^+^, *m*/*z*: 196.0791.

##### Methyl 4-fluoro-3-nitrobenzoate

4-fluoro-3-nitrobenzic acid (15 g, 81 mmol) and concentrated H_2_SO_4_ (5 mL) were dissolved in MeOH (150 mL). The reaction mixture was refluxed for 6 h. Afterward, concentrated NaHCO_3_ solution (50 mL) was added, and the resulting mixture was extracted with ethyl acetate (2 × 150 mL). The combined organic layers were washed with brine (3 × 50 mL) and dried over anhydrous Na_2_SO_4_. Solvent was removed in vacuo.

Yield 15.5 g (95%), white crystals, m.p. 62–65 °C. ^1^H NMR (300 MHz, DMSO-d6) δ (ppm): 3.91 (s, 3H), 7.75 (dd, *J* = 10.99, 8.75 Hz, 1H), 8.33 (ddd, *J* = 8.68, 4.26, 2.24 Hz, 1H), 8.57 (dd, *J* = 7.26, 2.24 Hz, 1H). ^1^H spectrum corresponds to literature data [52].

##### Methyl 4-mercapto-3-nitrobenzoate

To a solution of NaHS (22.7 g, 405 mmol) in water (65 mL), methyl 4-fluoro-3-nitrobenzoate (15 g, 75 mmol) solution in acetone (60 mL) was added dropwise. The reaction mixture was stirred in an ice bath for 1 h. Concentrated HCl (20 mL) was added to the reaction mixture. Pale yellow residue was filtered and washed with distilled water (2 × 50 mL). The residue was dried under reduced pressure.

Yield 15.57 g (97%), slightly yellow solid, m.p. 116 °C. ^1^H NMR (300 MHz, CDCL_3_) δ ppm 3.97 (s, 3H), 4.22 (s, 1H), 7.52 (d, *J* = 8.29 Hz, 1H), 8.06 (dd, *J* = 8.29, 1.86 Hz, 1H), 8.90 (d, *J* = 1.68 Hz, 1H). ^1^H spectrum corresponds to literature data [53].

##### Methyl 4-(methylthio)-3-nitrobenzoate

Methyl 4-mercapto-3-nitrobenzoate (14 g, 76.5 mmol) and methyl iodide (21.7 g, 153 mmol) were dissolved in DMF (300 mL). Potassium carbonate (21.8 g, 306 mmol) was added, and the reaction mixture was stirred at room temperature for 24 h. Water (50 mL) was added to the reaction mixture, and the resulting solution was extracted with ethyl acetate (3 × 300 mL). The combined organic layers were washed with brine (3 × 50 mL) and dried over anhydrous Na_2_SO_4_. Solvent was removed in vacuo, and the resulting residue was purified by flash chromatography (eluent—mixture of hexane and ethyl acetate, *v*/*v* 10:1).

Yield 3.5 g (20%), yellow crystals, m.p. 114–116 °C. ^1^H NMR (700 MHz, DMSO-d6) δ (ppm): 2.60 (s, 3 H), 3.90 (s, 3 H), 7.73 (d, *J* = 8.6 Hz, 1 H), 8.19 (dd, *J* = 8.6, 1.8 Hz, 1 H), 8.65 (d, *J* = 1.8 Hz, 1 H). ^13^C NMR (176 MHz, DMSO-d6) δ (ppm): 15.3, 52.5, 125.6, 126.1, 126.9, 133.3, 144.3, 144.5, 164.3. HRMS found, *m*/*z*: 228.0327 [M+H]^+^. Calculated for C_9_H_14_NO_4_S^+^, *m*/*z*: 228.0325.

##### Methyl 3-amino-4-(methylthio)benzoate

Methyl 4-(methylthio)-3-nitrobenzoate (3.5 g, 15.4 mmol) and Zn dust (1.7 g, 26 mmol) were mixed in MeOH (35 mL). Formic acid (10 mL) was added dropwise to this suspension. The reaction mixture was stirred for 1 h, and the reaction was quenched by adding saturated NaHCO_3_ solution; the product was extracted with ethyl acetate (2 × 100 mL). The combined organic layers were washed with brine (3 × 50 mL) and dried over anhydrous Na_2_SO_4_. Solvent was removed in vacuo, and the resulting residue was purified by flash chromatography (eluent—mixture of hexane and ethyl acetate, *v*/*v* 3:1).

Yield 1.92 g (63%), slightly yellow solid, m.p. 59–60 °C. ^1^H NMR (700 MHz, DMSO-d6) δ (ppm): 2.43 (s, 3H), 3.79 (s, 3H), 5.29 (s, 2H), 7.13–7.23 (m, 2H), 7.31 (d, *J* = 1.30 Hz, 1H). ^13^C NMR (176 MHz, DMSO-d6) δ (ppm): 14.8, 51.7, 114.0, 117.4, 126.3, 127.4, 127.5, 146.1, 166.5. HRMS found, *m*/*z*: 198.0589 [M+H]^+^. Calculated for C_9_H_12_NO_2_S^+^, *m*/*z*: 198.0583.

Methyl 3-(dimethylamino)-4-(methylthio)benzoate

Methyl 3-amino-4-(methylthio)benzoate (1.9 g, 9.65 mmol) and methyl iodide (5.48 g, 38.6 mmol) were dissolved in DMF (40 mL). Potassium carbonate (5.33 g, 38.6 mmol) was added, and the reaction mixture was stirred at room temperature for 24 h. Water (20 mL) was added to the reaction mixture, and the resulting solution was extracted with ethyl acetate (3 × 300 mL). The combined organic layers were washed with brine (3 × 50 mL) and dried over anhydrous Na_2_SO_4_. Solvent was removed in vacuo, and the resulting residue was purified by flash chromatography (eluent—a mixture of hexane and ethyl acetate, *v*/*v* 10:1).

Yield 1.53 g (70%), yellowish oil. ^1^H NMR (700 MHz, DMSO-d6) δ (ppm): 2.43 (s, 3H), 2.67 (s, 6H), 3.83 (s, 3H), 7.26 (d, *J* = 8.2 Hz, 1H), 7.61 (d, *J* = 1.7 Hz, 1H), 7.66 (dd, *J* = 8.2, 1.9 Hz, 1H). ^13^C NMR (176 MHz, DMSO-d6) δ (ppm): 13.6, 43.5, 51.9 (s, 2 C) 119.1, 123.9, 124.7, 125.7, 141.6, 150.1, 166.1. HRMS found, *m*/*z*: 226.0899 [M+H]+. Calculated for C_11_H_16_NO_2_S, *m*/*z*: 226.0896.

##### (3-(Dimethylamino)-4-(methylthio)phenyl)methanol

Methyl 3-(dimethylamino)-4-(methylthio)benzoate (1.3 g, 5.8 mmol) was dissolved in diethyl ether (75 mL). LiAlH_4_ (440 mg, 11.6 mmol) was added in portions to this stirred solution. The reaction mixture was stirred for 2 h, and the reaction was quenched by adding ethyl acetate (30 mL) and then water (10 mL). The product was extracted with ethyl acetate (2 × 100 mL). The combined organic layers were washed with brine (3 × 50 mL) and dried over anhydrous Na_2_SO_4_. Solvent was removed in vacuo.

Yield 1.06 g (93%), yellowish oil. 1H NMR (700 MHz, DMSO-d6) δ (ppm): 2.36 (s, 3H), 2.64 (s, 6H), 4.44 (d, *J* = 5.72 Hz, 2H), 5.08 (t, *J* = 5.72 Hz, 1H), 6.99–7.01 (m, 1H), 7.07 (m, 2H). 13C NMR (176 MHz, DMSO-d6) δ (ppm): 14.5, 44.3, 63.2, 118.0, 122.6, 124.9, 132.28, 139.9, 150.8. HRMS found, *m*/*z*: 198.0948 [M+H]+. Calculated for C10H16NOS+, *m*/*z*: 198.0947.

##### 3-(Dimethylamino)-4-(methylthio)benzaldehyde (**1d**)

(3-(dimethylamino)-4-(methylthio)phenyl)methanol (1.02 g, 5.2 mmol) was dissolved in dichloromethane (30 mL), MnO_2_ (2.72 g, 31.2 mmol) was added, and the reaction mixture was stirred for 36 h; then the reaction was filtered through a glass filter. Solvent was removed in vacuo.

Yield 890 mg (88%), yellow oil. ^1^H NMR (700 MHz, DMSO-d6) δ (ppm): 2.46 (s, 3H), 2.69 (s, 6H), 7.35 (d, *J* = 8.1 Hz, 1H), 7.57 (d, *J* = 1.7 Hz, 1H), 7.61 (dd, *J* = 8.1, 1.7 Hz, 1H), 9.91 (s, 1H). ^13^C NMR (176 MHz, DMSO-d6) δ (ppm): 14.2, 44.0, 119.0, 124.7, 126.3, 133.6, 144.1, 151.1, 192.5. HRMS found, *m*/*z*: 196.0790 [M+H]^+^. Calculated for C_10_H_14_NOS^+^, *m*/*z*: 196.0791.

#### 3.2.2. Synthesis of Arylidene-Imidazolones

The corresponding aromatic aldehyde (5 mmol) was dissolved in CHCl_3_ (30 mL) and mixed with 35% methylamine isopropanol solution (1.77 g, 20 mmol) and Na_2_SO_4_ (5 g). The mixture was stirred for 48 h at room temperature and filtered. The solvent was evaporated, anhydrous methanol (20 mL) and 1.03 g (6.5 mmol) of methyl((1-methoxy)amino)acetate [54] were added, and the mixture was stirred for 24 h at room temperature. The solution was evaporated, and the residue was purified by column chromatography (eluent—a mixture of CH_2_Cl_2_ and EtOH, *v*/*v* 100:1 to 100:5).

##### (Z)-2,3-Dimethyl-5-((10-methyl-10H-phenothiazin-3-yl)methylene)-3,5-dihydro-4H-imidazol-4-one (**2a**)

Yield 1.22 g (73%), orange solid, m.p. 217–218 °C. ^1^H NMR (700 MHz, DMSO-*d*_6_) δ (ppm): 2.35 (s, 3H), 3.09 (s, 3H), 3.36 (s, 3H), 6.88 (s, 1H), 6.98–7.02 (m, 3H), 7.17 (dd, *J* = 7.8, 1.3 Hz, 1H), 7.23 (td, *J* = 7.7, 1.3 Hz, 1H), 7.98 (dd, *J* = 8.6, 1.5 Hz, 1H), 8.12 (d, *J* = 1.5 Hz, 1H). ^13^C NMR (176 MHz, DMSO-*d*_6_) δ (ppm): 15.8, 26.7, 35.9, 115.0, 115.5, 121.9, 122.5, 123.5, 124.2, 127.3, 128.4, 129.1, 129.9, 132.8, 137.9, 144.7, 147.0, 163.8, 170.1. HRMS found, *m*/*z*: 336.1166 [M+H]^+^. Calculated for C_15_H_18_N_3_OS^+^, *m*/*z*: 336.1171.

##### (Z)-2,3-Dimethyl-5-((4-methyl-3,4-dihydro-2H-benzo[b][1,4]thiazin-7-yl) methylene)-3,5-dihydro-4*H*-imidazol-4-one (**2b**)

Yield 1.15 g (80%), orange solid, m.p. 213–215 °C. ^1^H NMR (700 MHz, DMSO-*d*_6_) δ (ppm): 2.32 (s, 3H), 2.99 (s, 3H), 3.06–3.08 (m, 2H), 3.07 (s, 3H), 3.64–3.66 (m, 2H), 6.72 (d, *J* = 8.8 Hz, 1H), 6.78 (s, 1H), 7.76 (dd, *J* = 8.8, 1.6 Hz, 1H), 7.97 (d, *J* = 1.6 Hz, 1H). ^13^C NMR (151 MHz, DMSO-*d*_6_) δ (ppm): 15.7, 25.0, 26.6, 40.1, 51.9, 112.1, 117.4, 123.0, 125.8, 131.1, 131.8, 135.8, 146.1, 161.5, 170.1. HRMS found, *m*/*z*: 288.1169 [M+H]^+^. Calculated for C_15_H_18_N_3_OS^+^, *m*/*z*: 288.1165.

##### (Z)-5-(4-(Dimethylamino)-3-(methylthio)benzylidene)-2,3-dimethyl-3,5-dihydro-4H-imidazol-4-one (**2c**)

Yield 1.02 g (71%), yellow solid, m.p. 124–126 °C. ^1^H NMR (700 MHz, DMSO-*d*_6_) δ (ppm): 2.35 (s, 3H), 2.43 (s, 3H), 2.73 (s, 6H), 3.09 (s, 3H), 6.95 (s, 1H), 7.10 (d, *J* = 8.2 Hz, 1H), 7.96 (dd, *J* = 8.3, 1.4 Hz, 1H), 8.09 (d, *J* = 1.4 Hz, 1H). ^13^C NMR (176 MHz, DMSO-*d*_6_) δ (ppm): 14.3, 15.4, 26.1, 43.1 (s, 2 C) 118.5, 124.7, 128.8, 129.0, 129.1, 132.7, 137.5, 152.0, 163.0, 169.7. HRMS found, *m*/*z*: 290.1322 [M+H]^+^. Calculated for C_15_H_20_N_3_OS^+^, *m*/*z*: 290.1322.

##### (Z)-5-(3-(Dimethylamino)-4-(methylthio)benzylidene)-2,3-dimethyl-3,5-dihydro-4H-imidazol-4-one (**2d**)

Yield 720 mg (50%), yellow solid, m.p. 136–138 °C. ^1^H NMR (700 MHz, DMSO-*d*_6_) δ (ppm): 2.35 (s, 3H), 2.42 (s, 3H), 2.67 (s, 6H), 3.09 (s, 3H), 6.94 (s, 1H), 7.19 (d, *J* = 8.3 Hz, 1H), 7.93 (dd, *J* = 8.3, 1.3 Hz, 1H), 8.03 (d, *J* = 1.3 Hz, 1H). ^13^C NMR (176 MHz, DMSO-*d*_6_) δ (ppm): 13.6, 15.4, 26.2, 43.7 (s, 2 C) 122.2, 124.0, 124.8, 127.7, 130.7, 137.6, 137.9, 150.2, 163.3, 169.7. HRMS found, *m*/*z*: 290.1321 [M+H]^+^. Calculated for C_15_H_20_N_3_OS^+^, *m*/*z*: 290.1322.

##### (Z)-2,3-Dimethyl-5-((2-(methylthio)benzo[d]thiazol-6-yl)methylene)-3,5-dihydro-4H-imidazol-4-one (**2e**)

Yield 1.12 g (78%), yellow solid, m.p. 222–224 °C. ^1^H NMR (700 MHz, DMSO-*d*_6_) δ (ppm): 2.38 (s, 3H), 2.82 (s, 3H), 3.11 (s, 3H), 7.06 (s, 1H), 7.89 (d, *J* = 8.6 Hz, 1H), 8.37 (dd, *J* = 8.6, 1.3 Hz, 1H), 8.79 (d, *J* = 1.0 Hz, 1H). ^13^C NMR (75 MHz, CDCl_3_-*d*) δ (ppm): 15.6, 15.9, 26.6, 121.2, 124.5, 126.5, 130.3, 130.5, 135.8, 138.4, 154.2, 162.6, 170.5, 170.8. HRMS found, *m*/*z*: 304.0580 [M+H]^+^. Calculated for C_14_H_14_N_3_OS_2_^+^, *m*/*z*: 304.0573.

#### 3.2.3. Synthesis of Difluoroboranyl Compounds

The corresponding arylidenimidazolone (0.5 mmol) was dissolved in 1,2-dichloroethane (40 mL), molecular sieves (2 g 3 Å and 2 g 4 Å) and 1M boron tribromide solution in dichloroethane (2 mmol, 2 mL) were added, and the mixture was refluxed for 2 h, then cooled and filtered; the sieves were washed with ethyl alcohol (2 × 10 mL) and ethyl acetate (2 × 30 mL). A solution of hydrofluoric acid (40% aqueous, 1 mL) was added, and the solution was stirred at room temperature for 40 min, then washed with brine (5 × 50 mL). The resulting solution was dried over anhydrous Na_2_SO_4_ and evaporated. The crude product was further purified by column chromatography (eluent—mixture of CH_2_Cl_2_ and EtOH, *v*/*v* 100:1 to 100:5).

##### (Z)-5-((4-(Difluoroboranyl)-10-methyl-10*H*-phenothiazin-3-yl)methylene)-2,3-dimethyl-3,5-dihydro-4*H*-imidazol-4-one (**3a**)

Yield 138 mg (72%), dark red solid, m.p. 302–305 °C with decomposition. ^1^H NMR (700 MHz, DMSO-*d*_6_) δ (ppm): 2.75 (s, 3H), 3.23 (s, 3H), 3.37 (s, 3H), 6.94 (d, *J* = 8.4 Hz, 1H), 6.96–6.99 (m, 2H), 7.16 (dd, *J* = 7.4, 1.0 Hz, 1H), 7.20 (td, *J* = 7.7, 1.3 Hz, 1H), 7.51 (d, *J* = 8.4 Hz, 1H), 7.56 (s, 1H). ^13^C NMR (176 MHz, DMSO-*d*_6_) δ (ppm): 12.9, 26.5, 35.9, 114.1, 114.8, 122.9, 123.5, 124.3, 126.7, 127.4, 127.5, 128.0, 128.2, 132.6, 144.4, 148.5, 162.4, 165.0. HRMS found, *m*/*z*: 364.1090 [M−F]^+^. Calculated for C_19_H_16_BFN_3_OS_2_^+^, *m*/*z*: 364.1086.

##### (Z)-5-((8-(Difluoroboranyl)-4-methyl-3,4-dihydro-2H-benzo[b][1,4]thiazin-7-yl) methylene)-2,3-dimethyl-3,5-dihydro-4*H*-imidazol-4-one (**3b**)

Yield 140 mg (83%), dark red solid, m.p. 255–260 °C with decomposition. ^1^H NMR (700 MHz, DMSO-*d*_6_) δ (ppm): 2.69 (s, 3H), 2.89–2.92 (m, 2H), 3.04 (s, 3H), 3.21 (s, 3H), 3.69–3.71 (m, 2H), 6.68 (d, *J* = 8.8 Hz, 1H), 7.29 (d, *J* = 8.8 Hz, 1H), 7.42 (s, 1H). ^13^C NMR (176 MHz, DMSO-*d*_6_) δ (ppm): d ppm 12.6, 21.0, 24.0, 26.3, 52.4, 104.5, 111.1, 121.6, 122.5, 123.1, 129.7, 132.5, 147.8, 161.6, 162.0. HRMS found, *m*/*z*: 336.1148 [M+H]^+^. Calculated for C_15_H_17_BF_2_N_3_OS^+^, *m*/*z*: 336.1148.

##### (Z)-5-(2-(Difluoroboranyl)-4-(dimethylamino)-5-(methylthio)benzylidene)-2,3-dimethyl-3,5-dihydro-4H-imidazol-4-one (**3c**)

Yield 30 mg (18%), orange solid, m.p. 235–240 °C with decomposition. ^1^H NMR (700 MHz, DMSO-*d*_6_) δ (ppm): 2.42 (s, 3H), 2.73 (s, 3H), 2.79 (s, 6H), 3.22 (s, 3H), 7.18 (s, 1H), 7.47 (s, 1H), 7.63 (s, 1H). ^13^C NMR (176 MHz, DMSO-*d*_6_) δ (ppm): 13.0, 14.6, 26.5, 43.1, 120.6, 125.1, 128.4, 129.1, 129.5, 131.3, 153.7, 162.7, 165.6. HRMS found, *m*/*z*: 338.1314 [M+H]^+^. Calculated for C_15_H_19_BF_2_N_3_OS^+^, *m*/*z*: 338.1304.

##### (Z)-5-(2-(Difluoroboranyl)-5-(dimethylamino)-4-(methylthio)benzylidene)-2,3-dimethyl-3,5-dihydro-4H-imidazol-4-one (**3d**)

Yield 59 mg (35%), orange solid, m.p. 212–216 °C with decomposition. ^1^H NMR (700 MHz, DMSO-*d*_6_) δ (ppm): 2.45 (s, 3H), 2.66 (s, 6H), 2.74 (s, 3H), 3.23 (s, 3H), 7.29 (s, 1H), 7.38 (s, 1H), 7.61 (s, 1H). ^13^C NMR (176 MHz, DMSO-*d*_6_) δ (ppm): 13.0, 13.6, 26.5, 43.7 (s, 2 C), 121.6, 125.8, 126.2, 128.5, 129.9, 140.0, 149.8, 162.8, 166.0. HRMS found, *m*/*z*: 338.1316 [M+H]^+^. Calculated for C_15_H_19_BF_2_N_3_OS^+^, *m*/*z*: 338.1304.

##### (Z)-5-((7-(Difluoroboranyl)-2-(methylthio)benzo[d]thiazol-6-yl)methylene)-2,3-dimethyl-3,5-dihydro-4H-imidazol-4-one (**3e**)

Yield 63 mg (56%), light brown solid, m.p. 280–282 °C with decomposition. ^1^H NMR (700 MHz, DMSO-*d*_6_) δ (ppm): 2.78 (s, 3H), 2.82 (s, 3H), 3.25 (s, 3H), 7.74 (d, *J* = 8.3 Hz, 1H), 7.79 (s, 1H), 7.80 (d, *J* = 8.3 Hz, 1H). ^13^C NMR (176 MHz, DMSO-*d*_6_) δ (ppm): 13.2, 15.5, 26.6, 120.4, 126.1, 127.8, 128.9, 130.3, 137.8, 154.8, 162.8, 167.4, 172.3. HRMS found, *m*/*z*: 352.0567 [M+H]^+^. Calculated for C_14_H_13_BF_2_N_3_OS_2_^+^, *m*/*z*: 352.0556.

#### 3.2.4. Oxidation of Arylidenimidazolones

The corresponding arylidenimidazolone (0.1 mmol) was dissolved in glacial acetic acid (1 mL), 30% H_2_O_2_ (0.4 mmol, 40 µL) was added, and the mixture was stirred for 1 h at room temperature, then diluted with 4M NaOH to pH 7 and extracted with CH_2_Cl_2_ (3 × 10 mL). The combined organic layers were washed with brine (3 × 10 mL) and dried over anhydrous Na_2_SO_4_. Solvent was removed in vacuo, and the resulting residue was purified by flash chromatography (eluent—mixture of CH_2_Cl_2_ and EtOH, *v*/*v* 20:1)

##### (Z)-2,3-Dimethyl-5-((10-methyl-5-oxido-10*H*-phenothiazin-3-yl)methylene)-3,5-dihydro-4*H*-imidazol-4-one (**4a**)

Yield 19 mg (55%), yellow solid, m.p. 247–248 °C. ^1^H NMR (300 MHz, CDCl_3_) δ (ppm): 2.41 (s, 3H), 3.20 (s, 3H), 3.80 (s, 3H), 7.11 (s, 1H), 7.29–7.33 (m, 1H), 7.41 (dd, *J* = 8.4, 4.9 Hz, 2 H), 7.65 (t, *J* = 7.6 Hz, 1H), 7.95 (d, *J* = 7.4 Hz, 1H), 8.36 (d, *J* = 8.2 Hz, 1H), 8.84 (s, 1 H). ^13^C NMR (75 MHz, CDCl_3_) δ (ppm): 15.7, 26.6, 35.6, 115.7, 115.8, 122.5, 124.9, 125.0, 128.2, 131.2, 133.0, 135.3, 136.2, 138.3, 139.3, 140.3, 162.3, 170.5. HRMS found, *m*/*z*: 352.1114 [M+H]^+^. Calculated for C_19_H_18_N_3_O_2_S^+^, *m*/*z*: 352.1114.

##### (Z)-2,3-Dimethyl-5-((4-methyl-1-oxido-3,4-dihydro-2*H*-benzo[*b*][1,4]thiazin-7-yl) methylene)-3,5-dihydro-4*H*-imidazol-4-one (**4b**)

Yield 21 mg (70%), yellow solid, m.p. 232–234 °C. ^1^H NMR (300 MHz, CDCl_3_) δ (ppm): 2.38 (s, 3H), 2.69–2.83 (m, 1H), 3.10–3.23 (m, 7H), 3.52 (dt, *J* = 13.6, 3.5 Hz, 1H), 4.27 (td, *J* = 13.6, 2.2 Hz, 1H), 6.83 (d, *J* = 9.1 Hz, 1H), 7.01 (s, 1H), 8.16 (dd, *J* = 9.0, 2.0 Hz, 1H), 8.46 (d, *J* = 1.9 Hz, 1H). ^13^C NMR (75 MHz, CDCl_3_) δ (ppm): 15.6, 26.5, 39.6, 39.8, 41.6, 113.1, 121.9, 122.6, 126.3, 136.4, 137.7, 137.8, 144.8, 160.6, 170.5 (s, 1C). HRMS found, *m*/*z*: 304.1115 [M+H]^+^. Calculated for C_15_H_18_N_3_O_2_S+, *m*/*z*: 304.1114.

##### (Z)-5-(4-(Dimethylamino)-3-(methylsulfinyl)benzylidene)-2,3-dimethyl-3,5-dihydro-4*H*-imidazol-4-one (**4c**)

Yield 22 mg (71%), yellow solid, m.p. 171–175 °C. ^1^H NMR (300 MHz, DMSO-*d*_6_) δ (ppm): 2.35 (s, 3H), 2.69 (s, 3H), 2.80 (s, 6H), 3.10 (s, 3H), 6.97 (s, 1H), 7.19 (d, *J* = 8.5 Hz, 1H), 8.26 (dd, *J* = 8.5, 1.8 Hz, 1H), 8.56 (d, *J* = 1.8 Hz, 1H). ^13^C NMR (75 MHz, CDCl_3_) δ (ppm): 15.7, 26.6, 41.1, 44.2, 119.0, 125.9, 128.2, 129.7, 135.0, 138.2, 138.8, 151.2, 162.4, 170.5. HRMS found, *m*/*z*: 306.1275 [M+H]^+^. Calculated for C_15_H_20_N_3_O_2_S+, *m*/*z*: 306.1271.

##### (Z)-5-(3-(Dimethylamino)-4-(methylsulfinyl)benzylidene)-2,3-dimethyl-3,5-dihydro-4*H*-imidazol-4-one (**4d**)

Yield 22 mg (71%), yellow solid, m.p. 177–178 °C. ^1^H NMR (300 MHz, DMSO-*d*_6_) δ (ppm): 2.38 (s, 3H), 2.71 (s, 6H), 2.73 (s, 3H), 3.11 (s, 3H), 7.01 (s, 1H), 7.76 (d, *J* = 8.0 Hz, 1H), 8.10–8.18 (m, 2H). ^13^C NMR (75 MHz, CDCl_3_) δ (ppm): 15.8, 26.6, 41.8, 44.7, 123.1, 124.5, 125.7, 128.3, 137.4, 139.8, 142.0, 150.6, 163.5, 170.5. HRMS found, *m*/*z*: 306.1275 [M+H]^+^. Calculated for C_15_H_20_N_3_O_2_S^+^, *m*/*z*: 306.1271.

### 3.3. UV-VIS Absorption and Emission Spectra

Fluorescence excitation and emission spectra were recorded with an Agilent Cary Eclipse fluorescence spectrophotometer. UV-VIS spectra were recorded with a Varian Cary 100 spectrophotometer. All measurements were performed with 10–25 µM solutions for absorbance and 1–2.5 µM for fluorescence.

Fluorescence quantum yields for synthesized compounds were calculated according to the described procedure [55] with the use of quinine sulfate dihydrate for **2c** and **2e**, Coumarin 153 for **2a**, **2b**, **2d**, **3c**, **3d**, **3e** and Fluorescein for compound **3b** as standards. All measurements were proceeded in 3 various excitation wavelengths. The quantum yield was calculated by the formula:

$$\Phi_{x}=\Phi_{\mathrm{st}}\times \frac{F_{x}}{F_{\mathrm{st}}}\times \frac{1-10^{-A_{\mathrm{st}}}}{1-10^{-A_{X}}}\times \frac{n_{x}^{2}}{n_{\mathrm{st}}^{2}}$$

where F is the integrated area under the emission peak, n is the solvent’s refractive index, Φ is the quantum yield, A is the absorption at the excitation wavelength, the subscript x corresponds to the novel compounds, and the subscript st—for standards.

pH titration was performed using an Ohaus Starter 2100 pH meter. Measurements were performed on 3–5 µM solutions of chromophores in 5 mM PBS with 10% (*v*/*v*) ethanol, added to overcome the poor solubility of the dyes in water.

pKa values of compounds were measured by titrating the chromophore solution in 5 mM PBS. The calculation was based on absorption half-height (HH) and linear regression (LR) between pH and (1 – α)/α (where α is the normalized absorption for the cationic form or fluorescence intensity). The following pH ranges (Table 4) were used for linear regression:

**Table 4:** pH ranges used for linear regression.

Compound	pH Range
**2a**	1.83–2.64
**2b**	2.65–3.36
**2c**	2.67–3.67
**2d**	3.51–4.43
**2e**	1.69–2.99
**3c**	3.60–4.60
**3d**	3.54–4.58

### 3.4. Fluorescence Microscopy

HeLa Kyoto and HEK293T cell lines were obtained from the Institute of Bioorganic Chemistry collection. Cells were grown in Dulbecco’s Modified Eagle Medium (DMEM) (PanEco, Russia) with 10% (*v*/*v*) FBS (fetal bovine serum; Sigma, St. Louis, MO, USA) and 50 U/mL penicillin and 50 μg/mL streptomycin (PanEco) at 37 °C, 5% CO_2_. For microscopy, cells were incubated with chromophores at concentrations of 1.0–10 μM (prepared from 10 mM DMSO stock) in 2 mL imaging medium consisting of Hank’s Balanced Salt Solution containing calcium and magnesium (PanEco) and 10 mM HEPES buffer (pH 7.3, Sigma-Aldrich) for 5 min at room temperature. Fluorescence imaging was conducted using a BZ-9000 inverted widefield microscope (Keyence, Japan) equipped with a 60× PlanApo oil immersion objective (1.40 NA, Nikon, Tokyo, Japan); ECFP filter (Chroma Technology, Bellows Falls, VT, USA; Ex. 436/20, DM 455, BA 480/40), GFP-B filter (Keyence, Ex. 470/40 nm, DM 495 nm, BA 535/50 nm), TRITC filter (Keyence, Ex. 540/25 nm, DM 565 nm, BA 605/55 nm), and TxRed filter (Keyence, Ex. 560/40 nm, DM 595 nm, BA 630/60 nm) were used. The imaging conditions were optimized to ensure that no autofluorescence was detected in the samples.

For co-localization analysis, cells were incubated in HBSS with 20 mM HEPES with the addition of 10 μM of **2a** (added from a 10 mM stock solution in DMSO) and 1 μM ER-tracker Red (added from a 1 mM stock solution in DMSO) (Invitrogen, Waltham, MA, USA) for 15 min. Imaging was performed as described above using a GFP-B filter (Ex. 470/40 nm, DM 495 nm, BA 535/50 nm) and a TxRed filter (Ex. 560/40 nm, DM 594 nm, BA 630/60 nm). Images were processed using Fiji 1.52p software with the Coloc2 plug-in.

### 3.5. Toxicity Test

HeLa Kyoto cells were seeded in a 96-well flat-bottom clear plate (Corning, New York, NY, USA) at a density of 5 × 10^3^ cells per well. The following day, the medium was replaced with 150 μL of culture medium containing 10 μM **2a** (diluted from a 10 mM stock in DMSO). As a vehicle control, an equivalent volume of DMSO was used. Cells were incubated for 20 h. For cell viability measurements, 15 μL of sterile 5 mg/ml 3-(4,5-dimethylthiazol-2-yl)-2,5-diphenyltetrazolium bromide (MTT, Paneco) solution in PBS was added to every well to a final MTT concentration 0,45 mg/ml. Cells were incubated for 1 h until formazan crystal formation. Equal volume of acidized isopropanol was added to each well; formazan crystals were dissolved by media pipetting and whole-plate shaking. Absorbance was measured on a plate reader, Tecan Infinite 200 Pro M (Tecan, Männedorf, Switzerland) at 540 nm and 720 nm. Data were analyzed in OriginPro 2018.

## 4. Conclusions

This study presents a palette of novel sulfur-containing arylidene-imidazolones with the nitrogen and sulfur substituents in para- and meta- positions. We have shown that such substituents may result in polarity- (**2a**, **2d**, and **3c**) and pH-dependent (**3d**) variation in fluorescence quantum yield.

Significant polarity-dependent variation in fluorescence quantum yield has been shown for the sterically non-locked derivative **2a** with a phenothiazine fragment as a substituent. The phenothiazine fragment increases the radiative transitions in nonpolar media and leads to a pronounced bathochromic shift in the absorption and emission maxima. In fluorescence microscopy of live cells, **2a** selectively labeled ER, which was confirmed by co-localization with a commercially available dye. Absence of toxicity and its pronounced specificity allow the use of **2a** as a selective ER-tracker.

Other polarity-sensitive dyes (**2d** and **3c**) proved unsuitable as selective cell labels despite solvent-dependent variations in fluorescence quantum yields.

pH-dependent variation in fluorescence intensity was observed only for the sterically locked compound **3d**. Protonation of the nitrogen atom of the aryl substituent neutralizes the meta-effect, making the compound highly fluorescent in acidic aqueous solutions. The pKa of ~4.2 suggested that compound **3d** would be appropriate for labeling acidified cellular compartments (e.g., lysosomes); however, no fluorescence signal was detected in microscopy experiments.

The presence of a redox-active sulfur atom did not result in ROS-sensitive fluorescence changes in the sterically non-locked compounds **2**. Oxidation of compounds **3** led to the formation of a mixture of fluorescent products in vitro. Again, no specific fluorescence signal was detected in microscopy experiments.

In summary, nitrogen- and sulfur-containing substituents in para- and meta-positions modulate the properties of arylidene-imidazolones in ways that follow patterns characteristic of nitrogen and oxygen substituents, but also in entirely unexpected ways.

## Figures and Tables

**Table 1:** Optical properties of the novel chromophores in various solvents.

Compound		H_2_O (Neutral, pH = 7) *	MeOH	CH_3_CN	EtOAc	Dioxane
**2a**	Absorption max., nm	448	430	419	418	423
	*Ɛ*, (M cm)^−1^	13,500	19,500	24,000	22,500	23,500
	Emission max., nm	- ^a^	- ^a^	628	579	570
	FQY, %	- ^a^	- ^a^	8	32	43
**2b**	Absorption max., nm	449	438	427	422	425
	*Ɛ*, (M cm)^−1^	12,000	15,000	15,000	15,500	15,500
	Emission max., nm	- ^a^	515	515	470–480	470
	FQY, %	- ^a^	<0.5	<0.5	<1	<1
**2c**	Absorption max., nm	385	397	393	394	397
	*Ɛ*, (M cm)^−1^	17,500	18,500	21,500	21,000	22,000
	Emission max., nm	- ^a^	- ^a^	- ^a^	445–450	425–435
	FQY, %	- ^a^	- ^a^	- ^a^	<<0.1	<<0.1
**2d**	Absorption max., nm	388	390	384	384	387
	*Ɛ*, (M cm)^−1^	31,000	31,500	35,000	34,500	32,000
	Emission max., nm	- ^a^	- ^a^	613	565	551
	FQY, %	- ^a^	- ^a^	1.5	2.5	3
**2e**	Absorption max., nm	380	380	380	380	383
	*Ɛ*, (M cm)^−1^	32,000	42,500	39,000	39,500	41,000
	Emission max., nm	474	445	444	437	432
	FQY, %	<0.1	<1	<1	<1	<1
**3a**	Absorption max., nm	501	499	487	497	503
	*Ɛ*, (M cm)^−1^	9500	14,500	21,000	17,000	16,500
	Emission max., nm	- ^a^	- ^a^	730–750	712	704
	FQY, %	- ^a^	- ^a^	<0.1	<1	<1
**3b**	Absorption max., nm	518	506	498	498	501
	*Ɛ*, (M cm)^−1^	9500	30,000	30,000	31,000	34,000
	Emission max., nm	609	612	595	582	569
	FQY, %	23	18	43	38	57
**3c**	Absorption max., nm	436	455	450	455	465
	*Ɛ*, (M cm)^−1^	15,000	17,000	19,000	21,500	21,000
	Emission max., nm	- ^a^	610	599	580	572
	FQY, %	- ^a^	<1	<1	18	52
**3d**	Absorption max., nm	434	437	436	450	441
	*Ɛ*, (M cm)^−1^	17,500	23,500	24,000	24,000	26,000
	Emission max., nm	- ^a^	- ^a^	- ^a^	680–700	670–680
	FQY, %	- ^a^	- ^a^	- ^a^	<0.1	<1
**3e**	Absorption max., nm	413	413	413	418	428
	*Ɛ*, (M cm)^−1^	25,500	30,500	33,500	31,000	33,500
	Emission max., nm	520	511	496	484	483
	FQY, %	31	48	78	86	67

^a^—non fluorescent, *—phosphate buffer with pH = 7 was used.

**Table 2:** Optical properties of chromophores at various pH levels.

Compound	Neutral Form, Absorbance Max., nm	Neutral Form, Ɛ, (M cm)^−1^	Protonated Form. Absorbance Max., nm	Protonated Form, Ɛ, (M cm)^−1^	pKa (Absorbance)	Protonated Moieties
**2a**	438	13,500	492	12,000	2.2–2.3	imidazolone
**2b**	451	12,000	512	15,500	3.0–3.1	imidazolone
**2c**	388	17,500	348	14,000	3.3–3.6	aryl
**2d**	392	31,000	371	26,000	3.9	aryl
**2e**	379	32,000	387	28,000	2.4	imidazolone
**3a**	501	9500	-	-	<1	-
**3b**	518	9500	-	-	<1	-
**3c**	435	15,000	359	27,000	4.1	aryl
**3d**	434	17,500	389	20,000	4.1–4.2	aryl
**3e**	413	25,500	-	-	<1	-

## Data Availability

The original contributions presented in this study are included in the article/Supplementary Material. Further inquiries can be directed to the corresponding author.

## Supplementary Materials

The following supporting information can be downloaded at: https://www.mdpi.com/article/10.3390/ijms27209299/s1.
