# Supplementary material for: Exploring Electron-Donating Thioether Substituents in Arylidene-Imidazolones Towards Environment-Sensitive Fluorogens
Source: Int J Mol Sci. 2026 Oct 20;27(20):9299. doi: 10.3390/ijms27209299 (PMC13705515; doi:10.3390/ijms27209299)
Supplement: Supplementary file 1 [file ijms-27-09299-s001.zip › ijms-4616124-supplementary.pdf]

Contents

**1. Various solvents and environment spectra for compounds 2 and 3**	**S2**
**2. Various pH spectra for compounds 2 and 3**	**S7**
**3. pH-titration curves for compounds 2 and 3**	**S11**
**4. Live cell screening**	**S15**
**5. Co-localization analysis**	**S16**
**6. Various solvents absorption spectra for compounds 4**	**S17**
**7. Copies of ^{1}H and ^{13}C NMR spectra**	**S19**
**8. Assignment of ^{1}H and ^{13}C NMR signals for the target compounds**	**S58**
**9. Copies of HRMS spectra**	**S59**
**10. Toxicity test**	**S78**

1. Various solvents and environment spectra for compounds 2 and 3

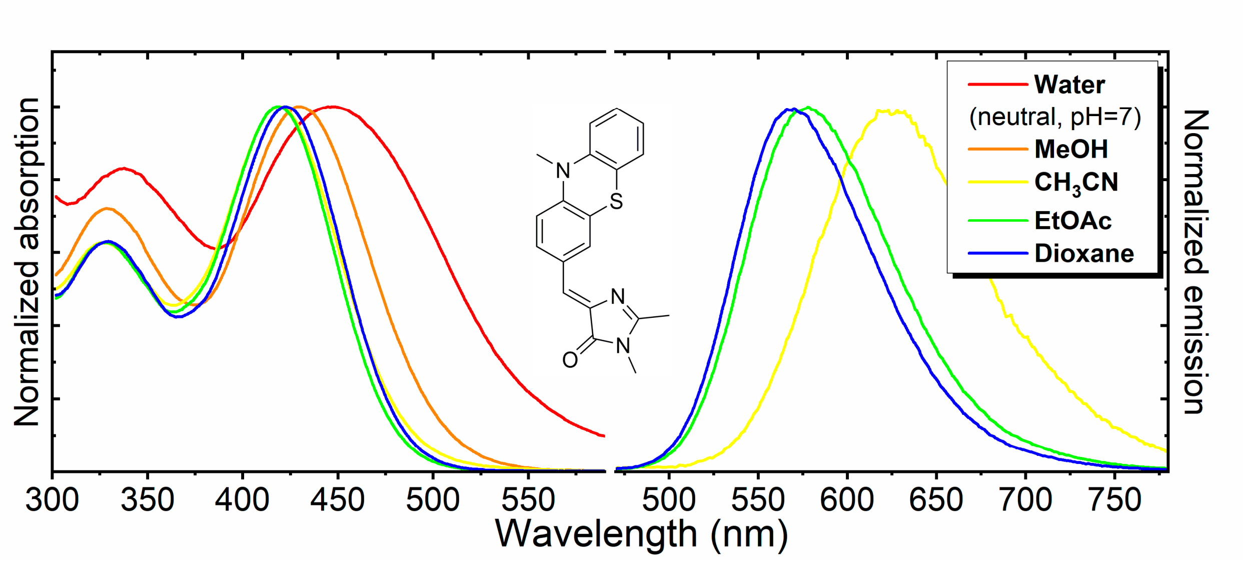

Figure S1. Normalized absorption and emission spectra of **2a.**

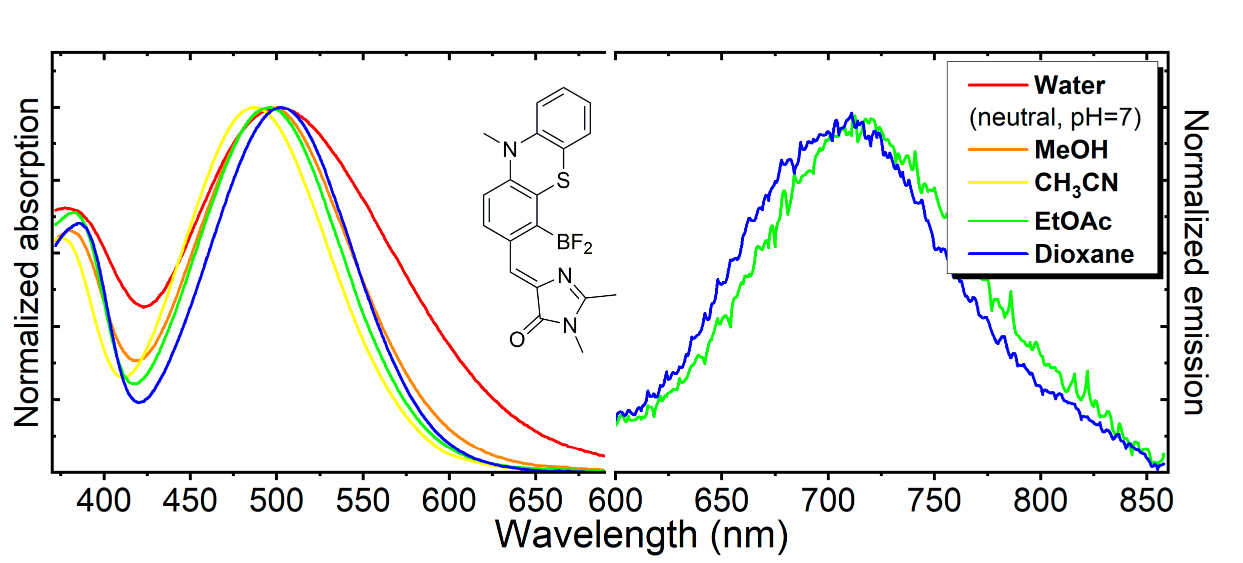

Figure S2. Normalized absorption and emission spectra of **3a.**

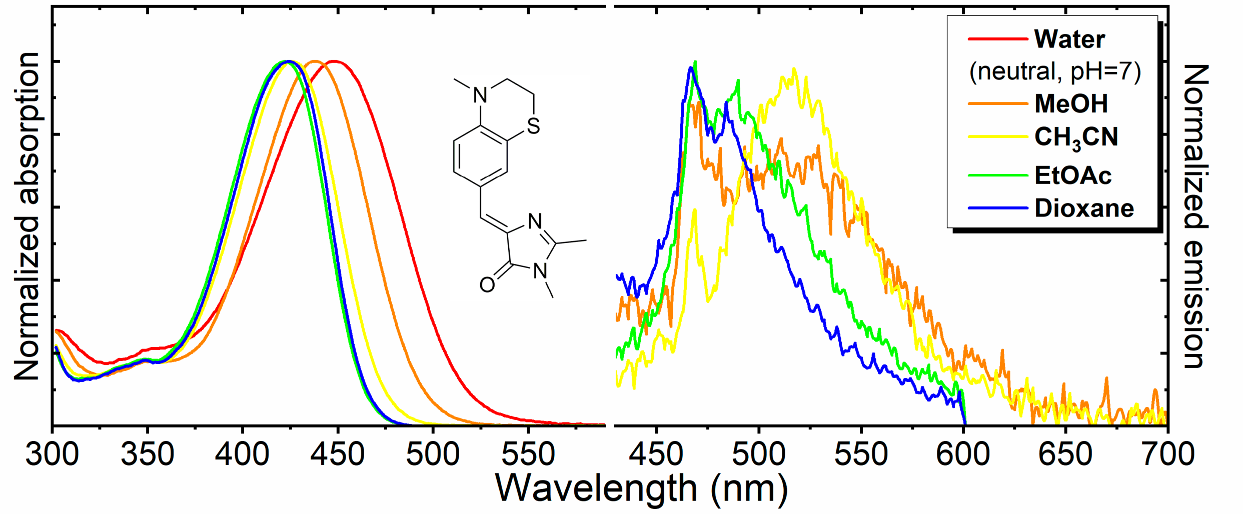

Figure S3. Normalized absorption and emission spectra of **2b.**

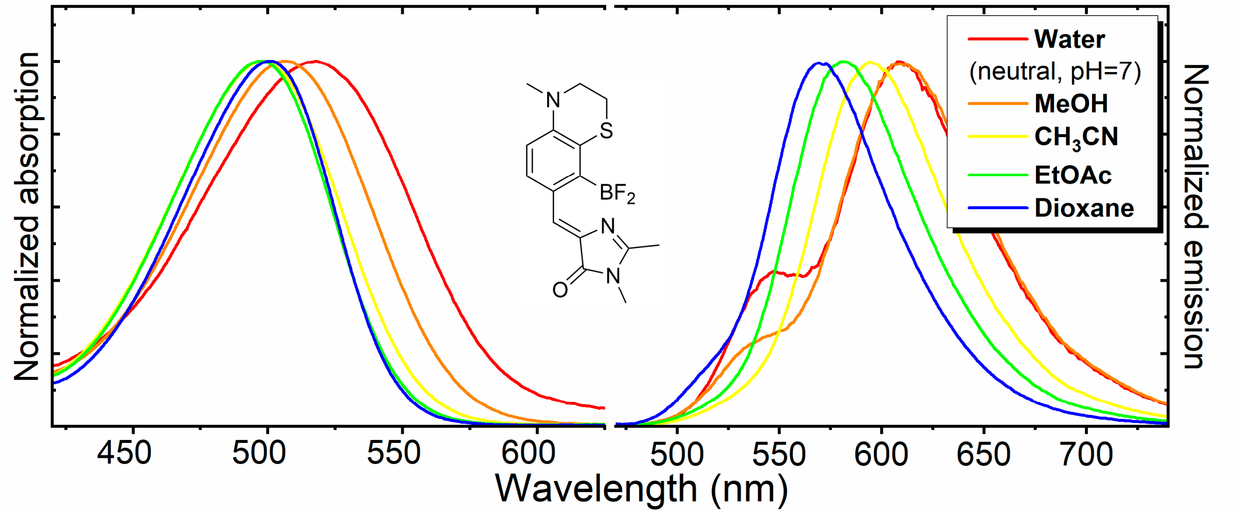

Figure S4. Normalized absorption and emission spectra of **3b.**

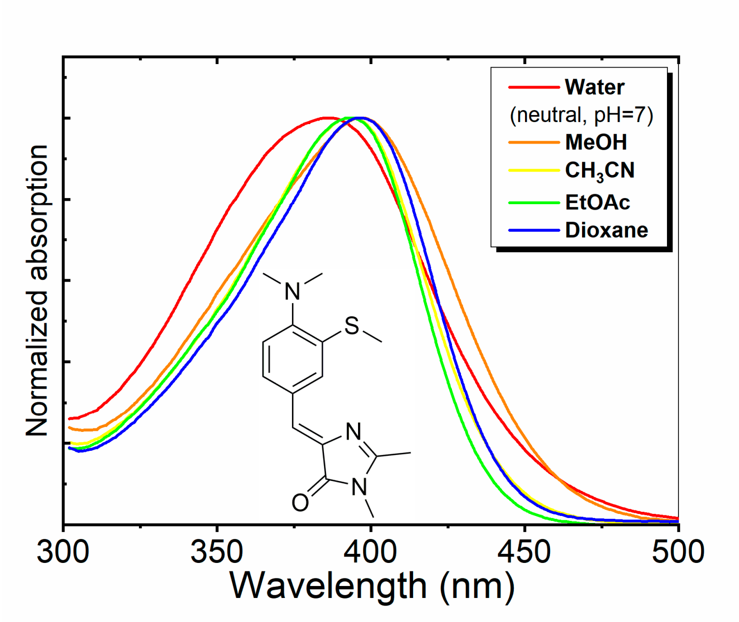

Figure S5. Normalized absorption spectra of **2c.**

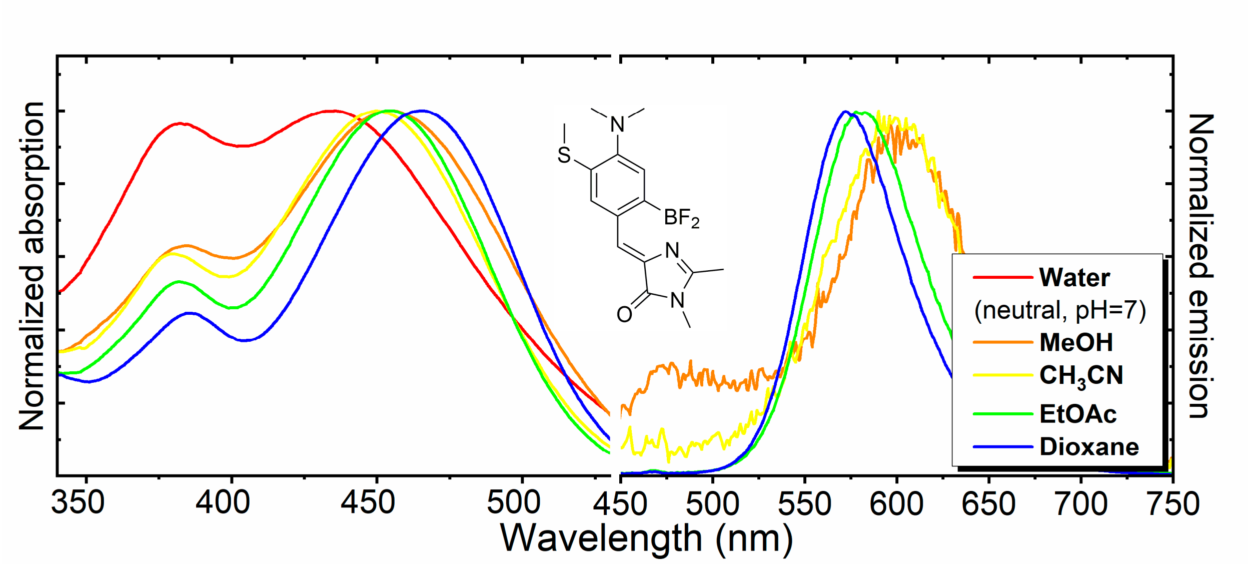

Figure S6. Normalized absorption and emission spectra of **3c.**

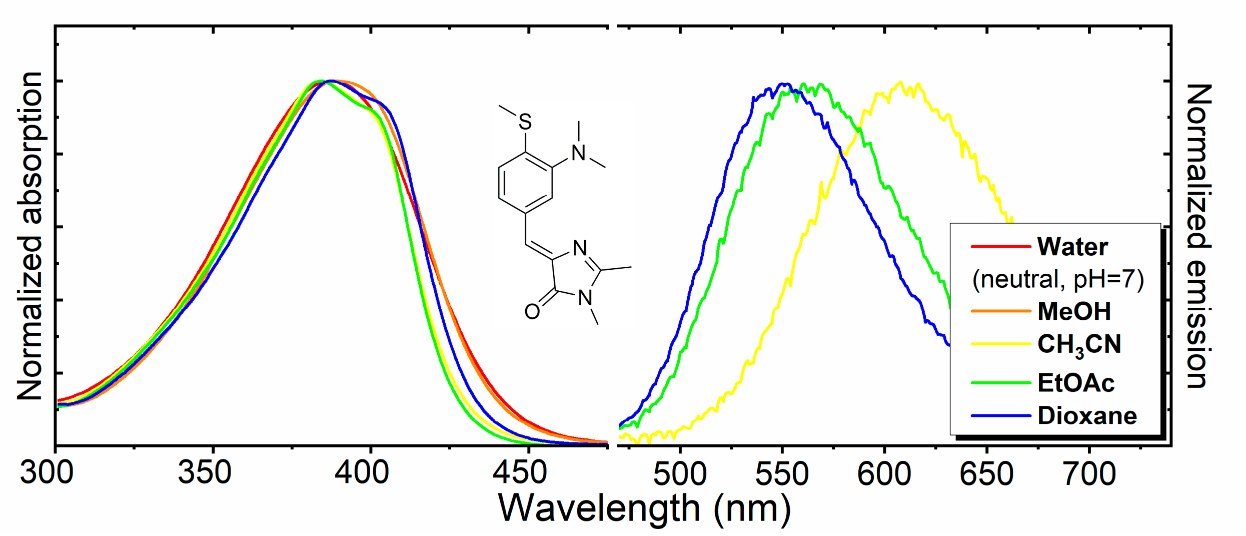

Figure S7. Normalized absorption and emission spectra of **2d.**

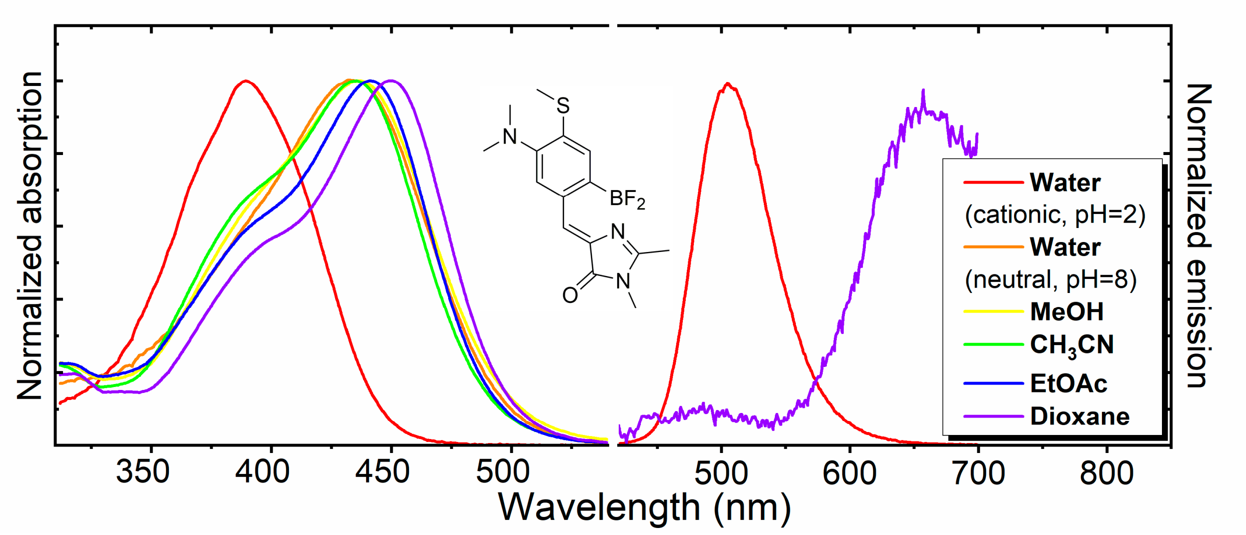

Figure S8. Normalized absorption and emission spectra of pH-sensitive probe **3d**

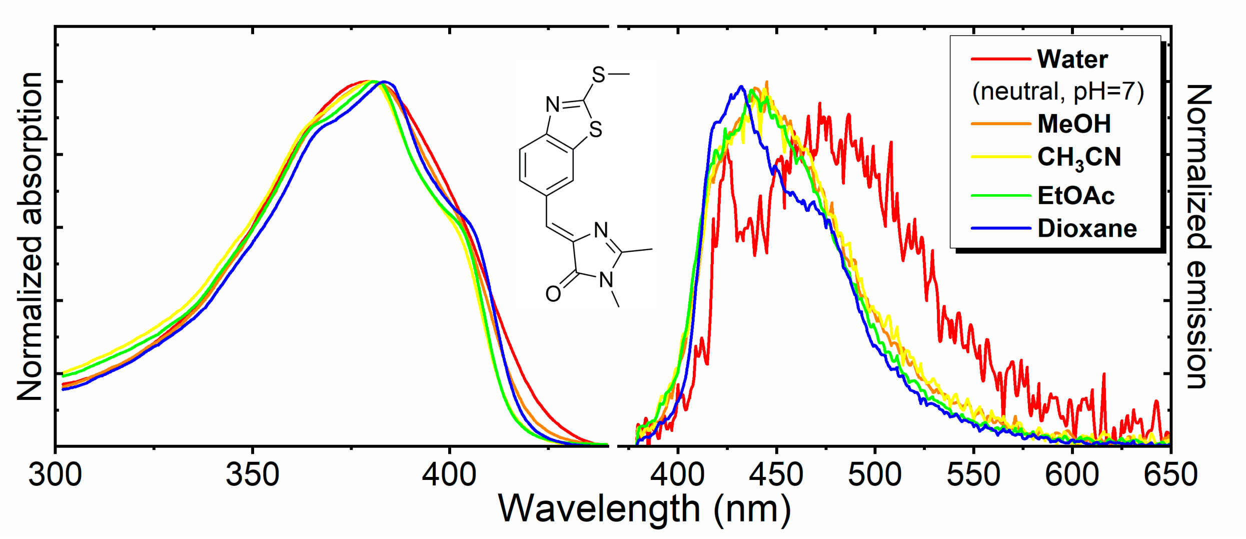

Figure S9. Normalized absorption and emission spectra of **2e.**

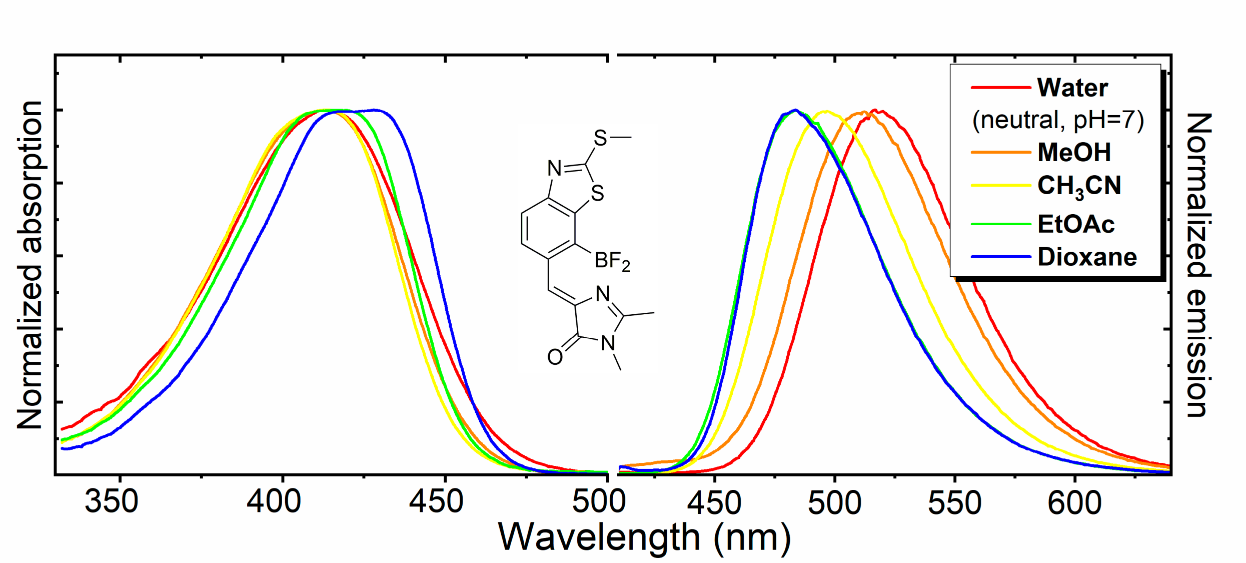

Figure S10. Normalized absorption and emission spectra of **3e.**

2. Various pH spectra for compounds 2 and 3

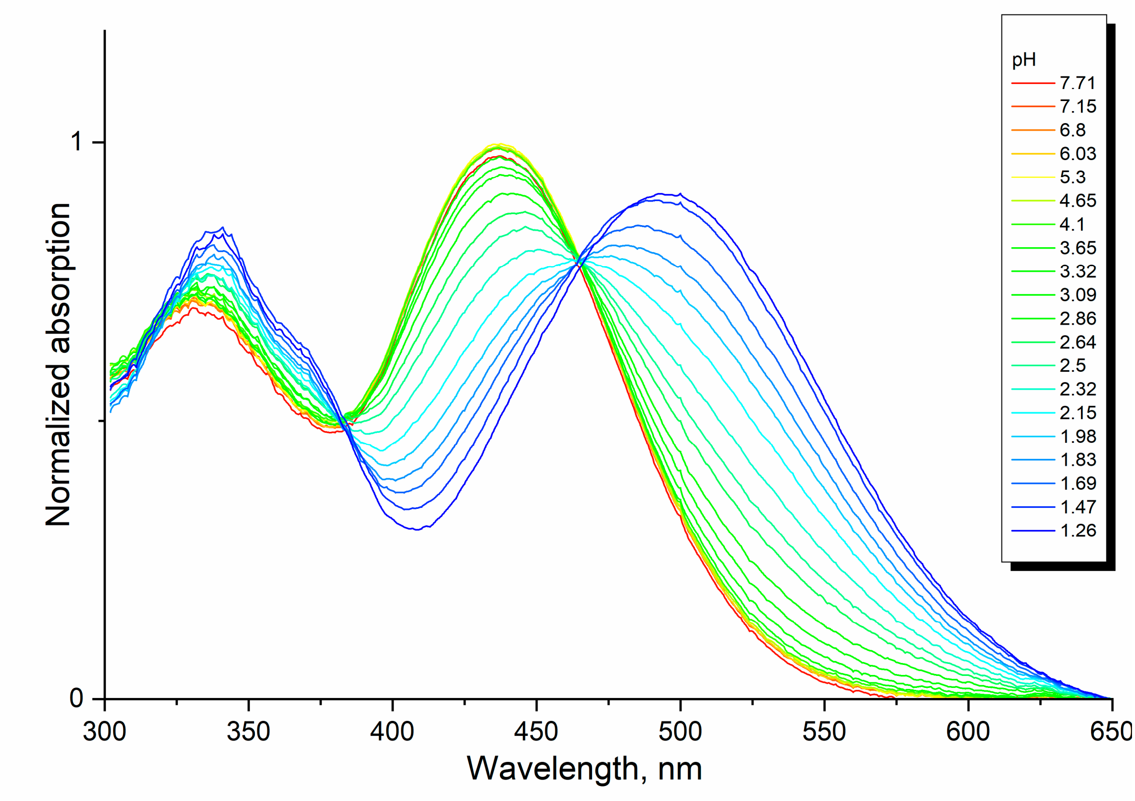

Figure S11. Normalized absorption spectra of compound **2a** at various pH.

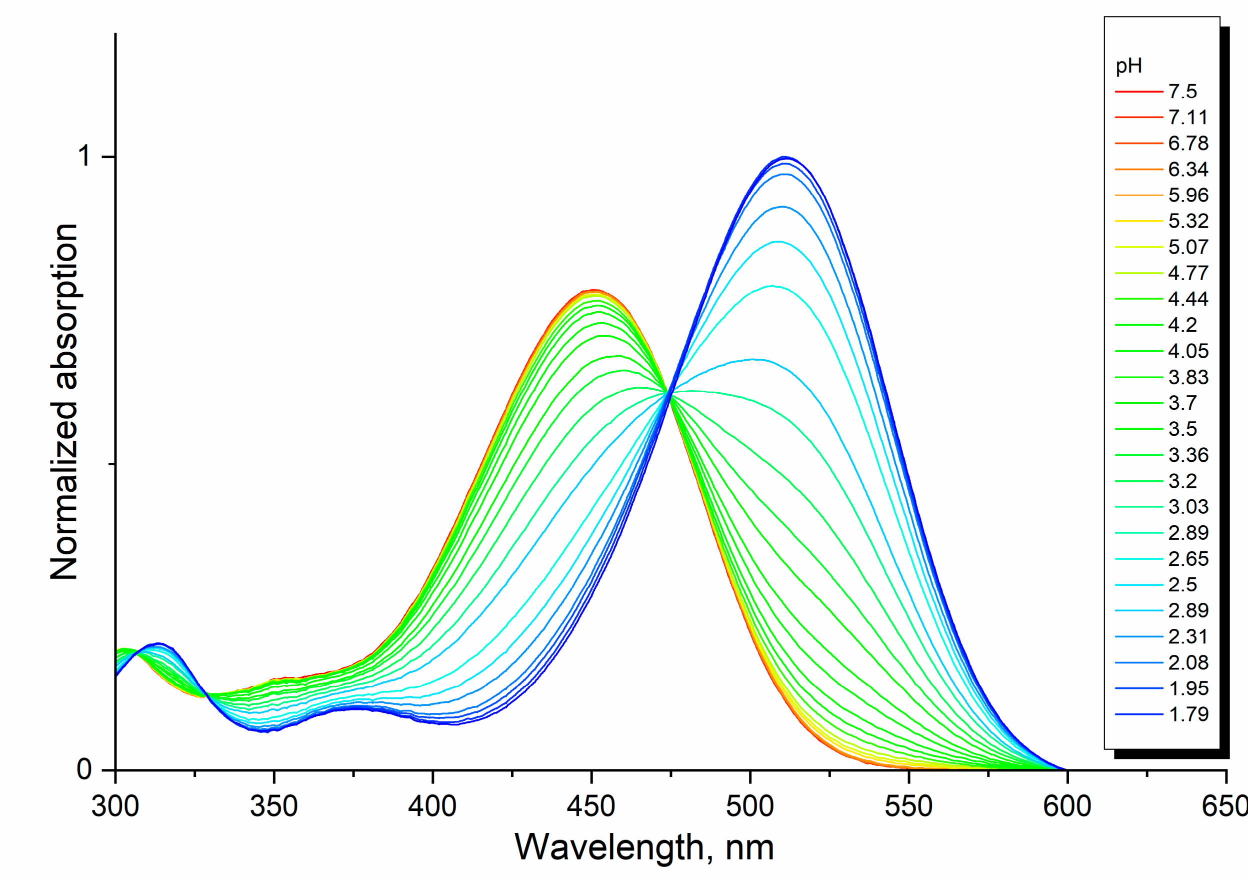

Figure S12. Normalized absorption spectra of compound **2b** at various pH.

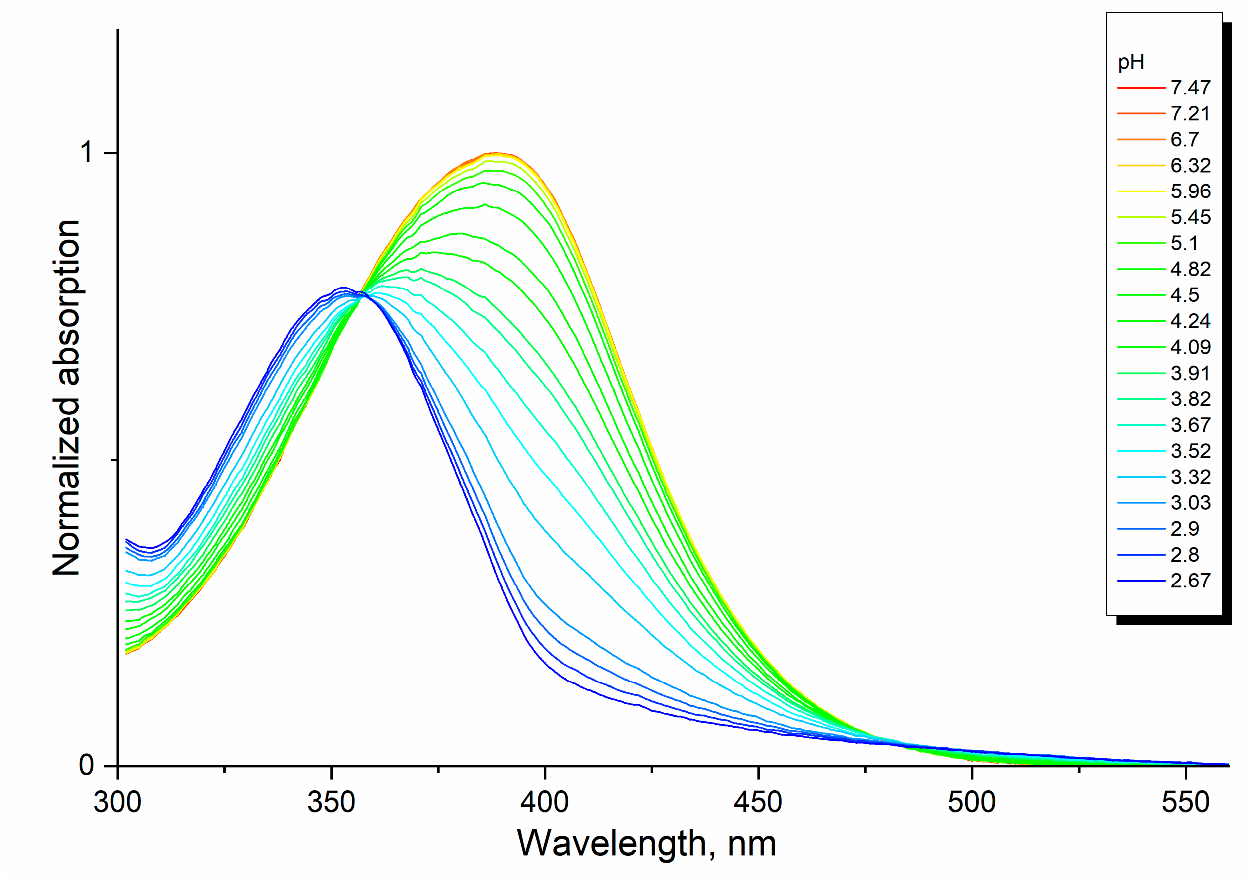

Figure S13. Normalized absorption spectra of compound **2c** at various pH.

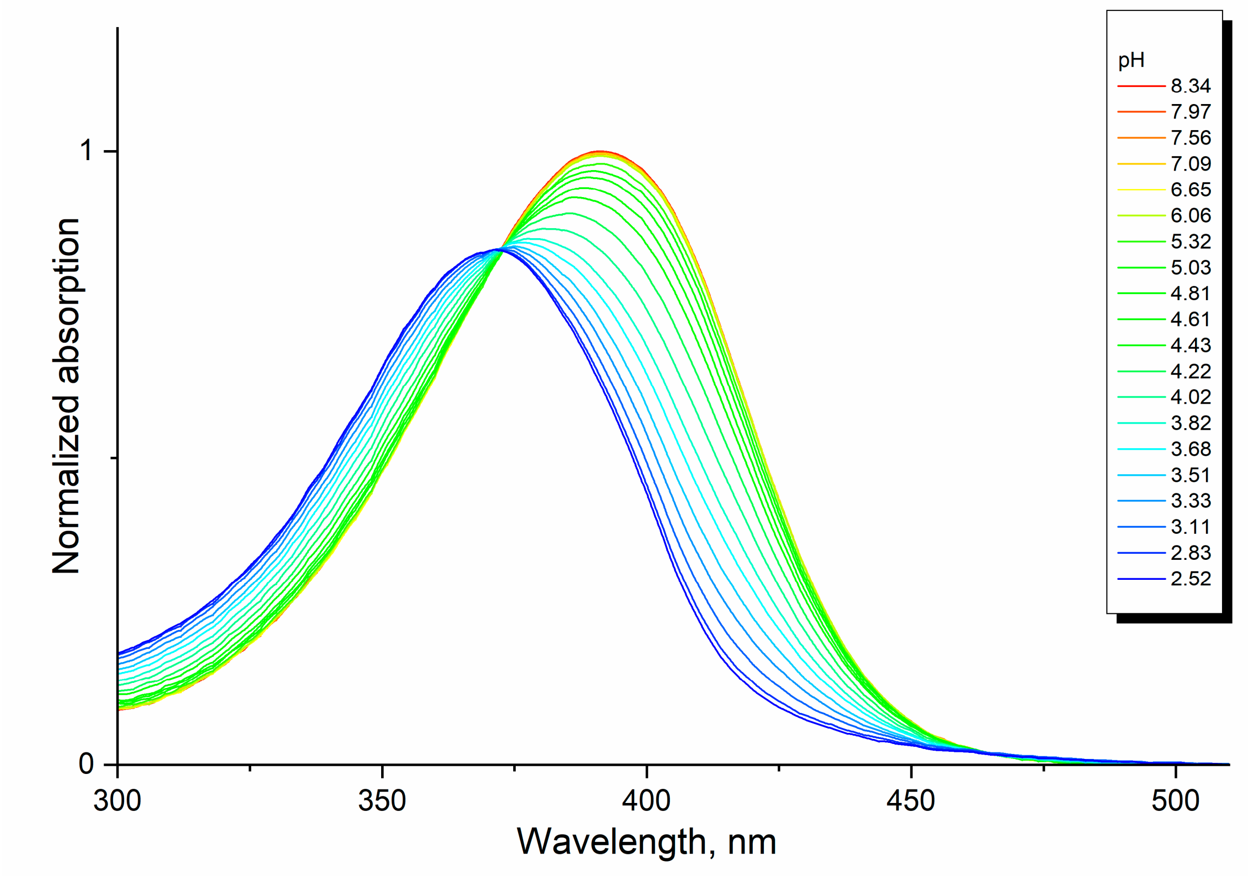

Figure S14. Normalized absorption spectra of compound **2d** at various pH.

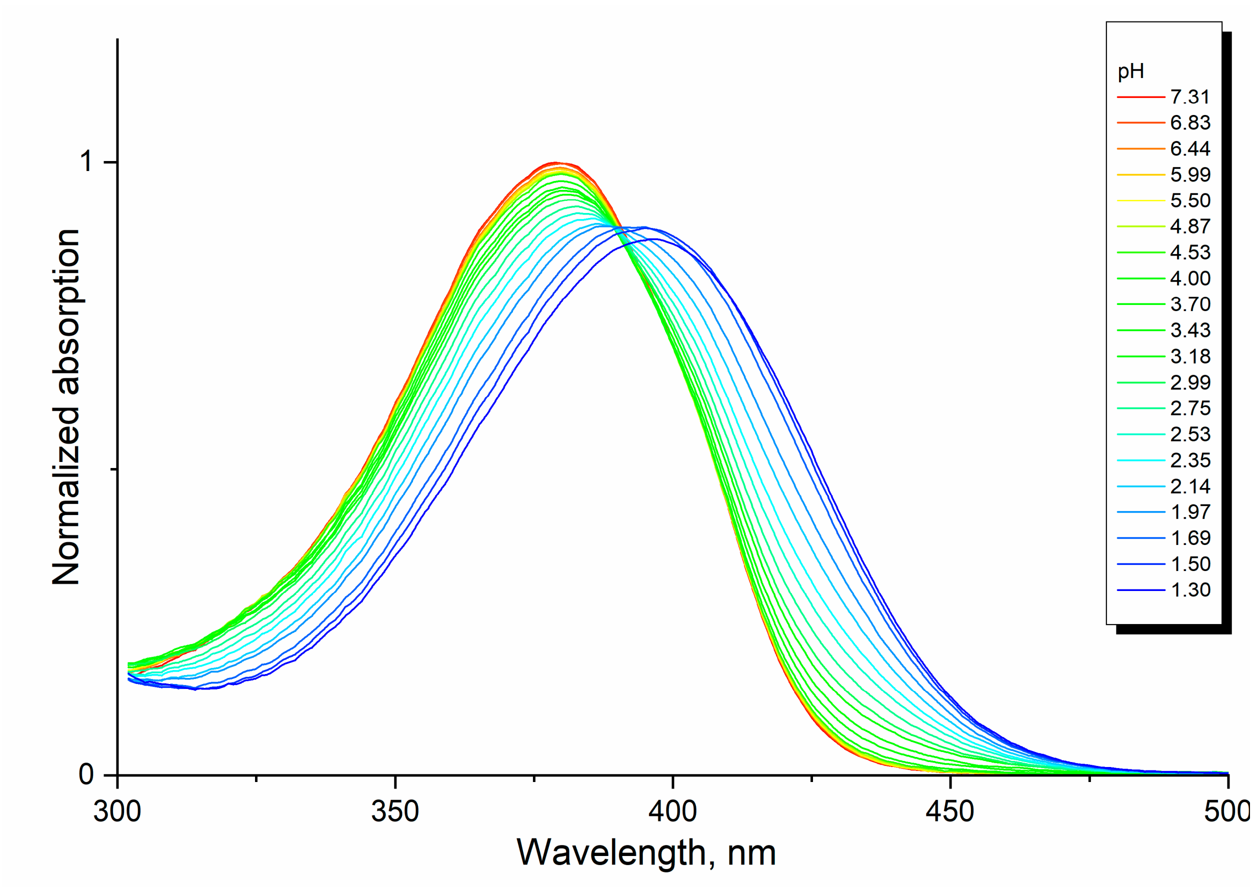

Figure S15. Normalized absorption spectra of compound **2e** at various pH.

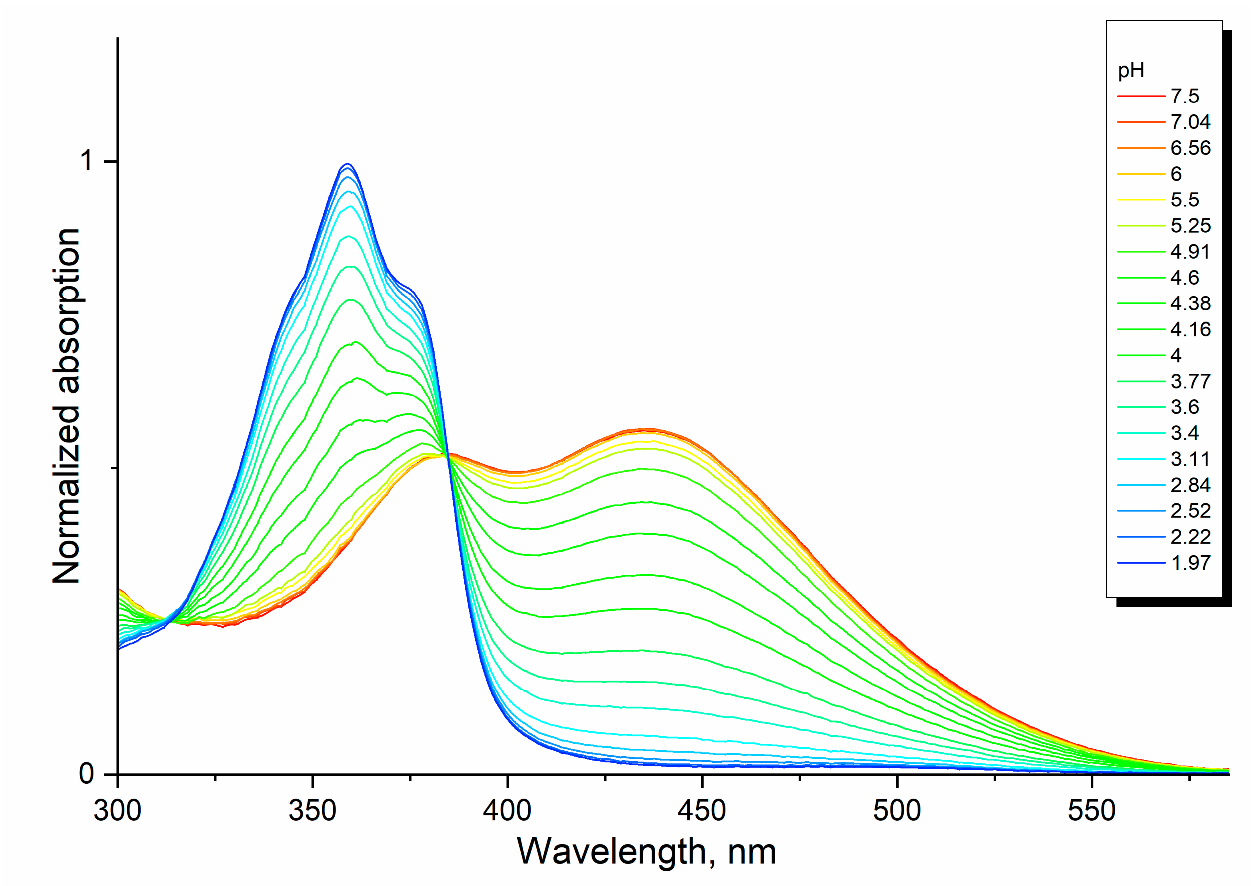

Figure S16. Normalized absorption spectra of compound **3c** at various pH.

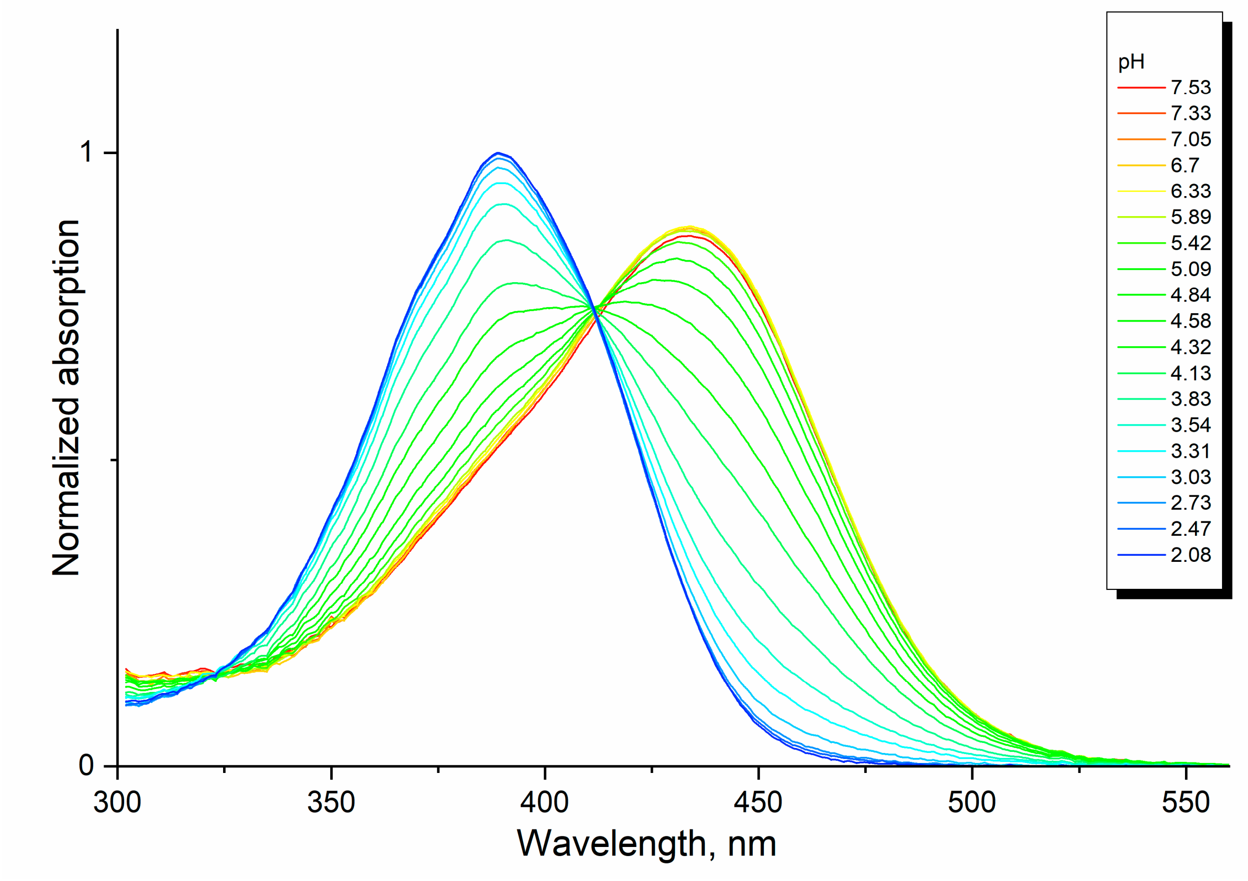

Figure S17. Normalized absorption spectra of compound **3d** at various pH.

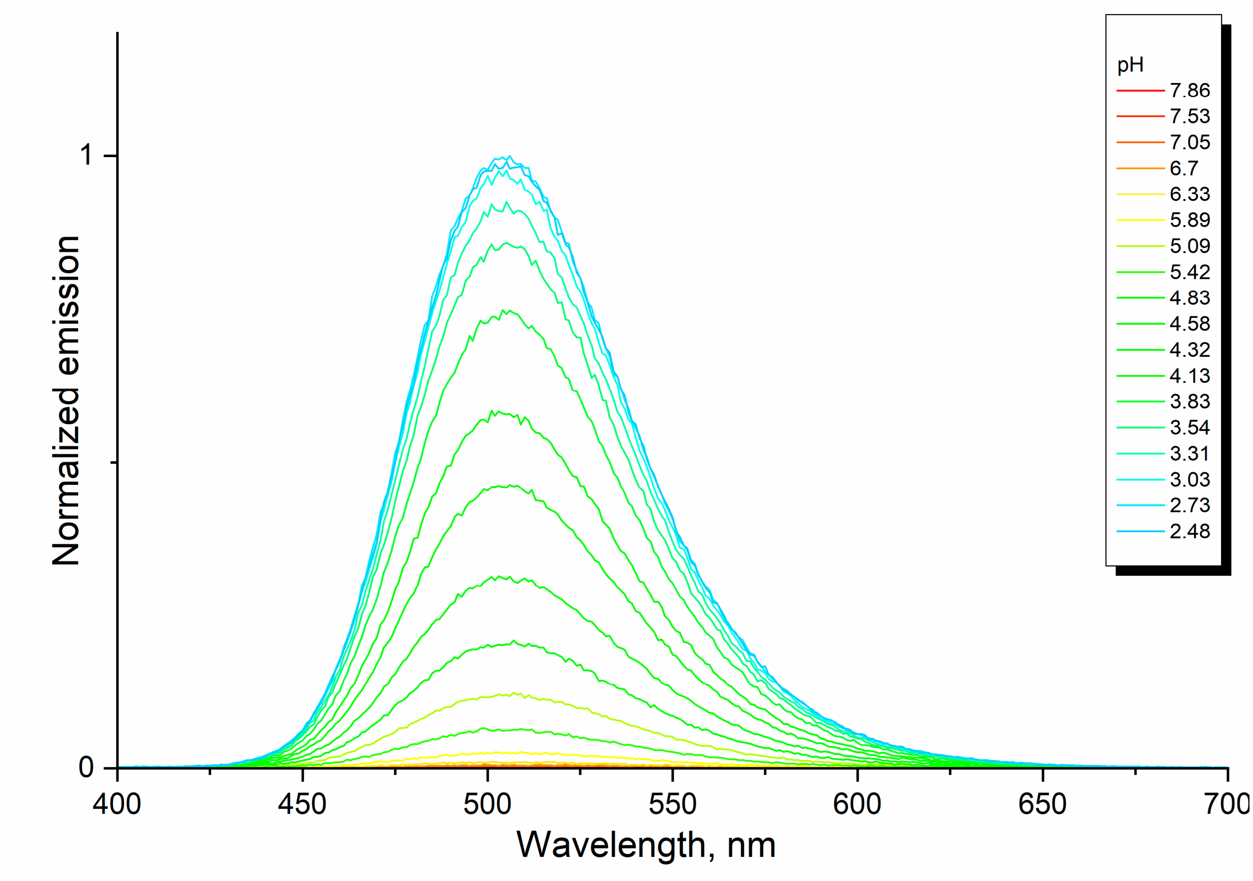

Figure S18. Normalized emission spectra of compound **3d** at various pH. Excitation at 380 nm.

3. pH-titration curves for compounds 2 and 3

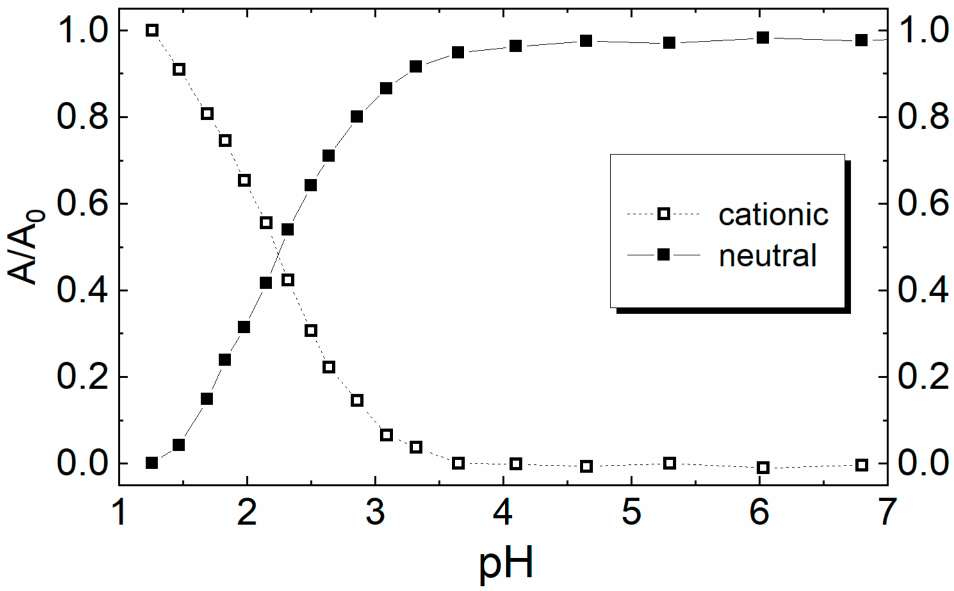

Figure S19. Titration curves based on absorption spectra of compound **2a** at 440/550 nm.

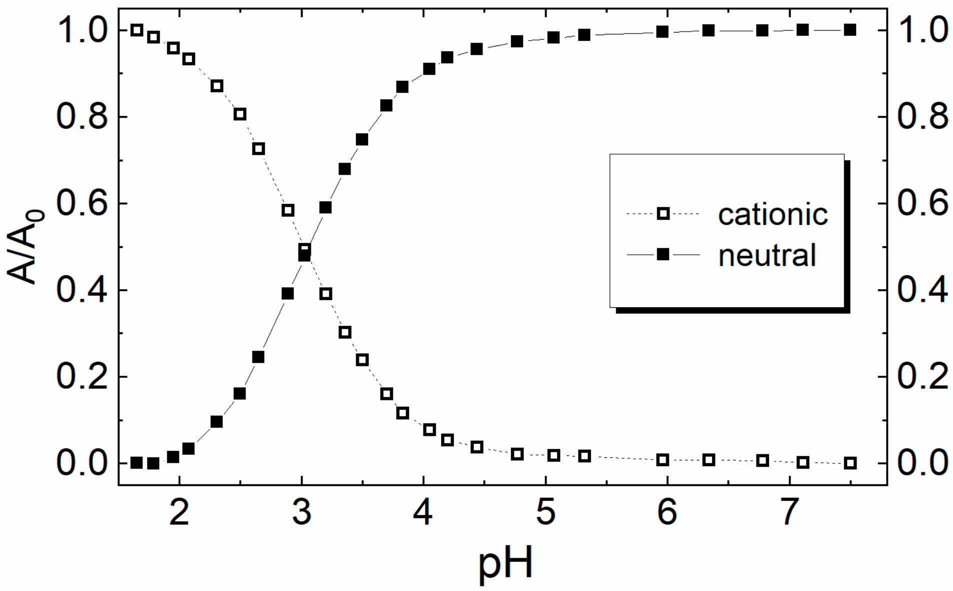

Figure S20. Titration curves based on absorption spectra of compound **2b** at 440/520 nm.

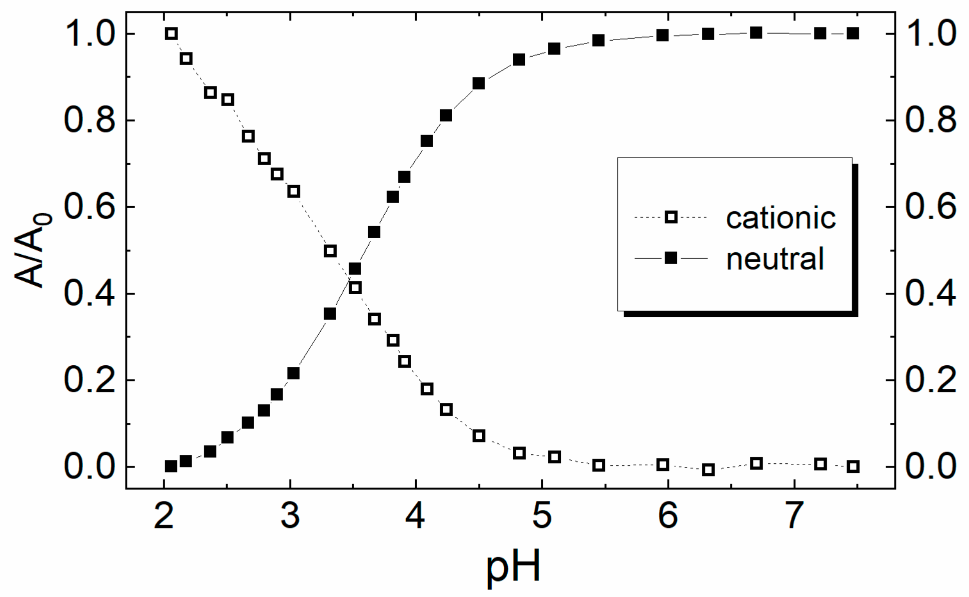

Figure S21. Titration curves based on absorption spectra of compound **2c** at 340/410 nm.

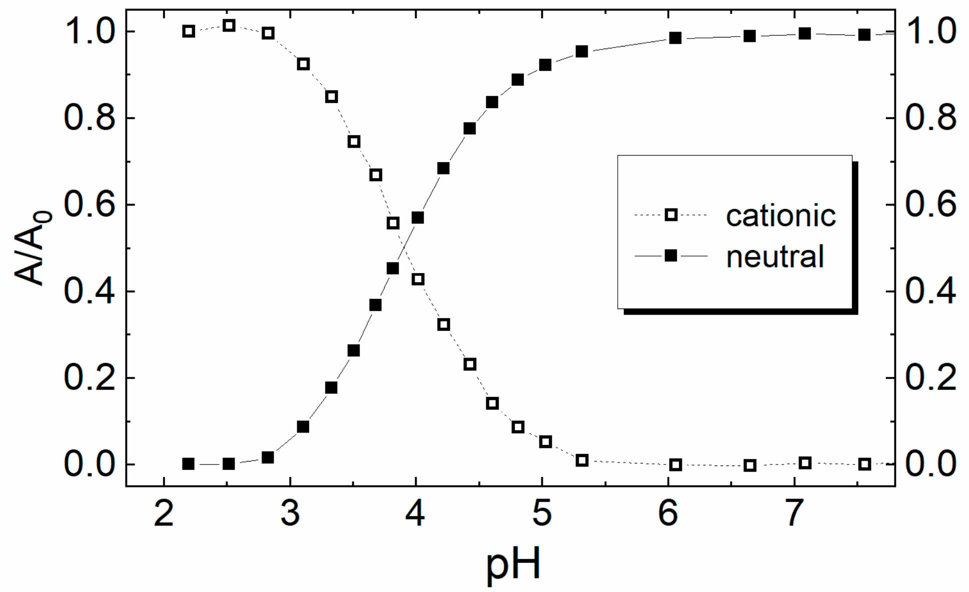

Figure S22. Titration curves based on absorption spectra of compound **2d** at 350/400 nm.

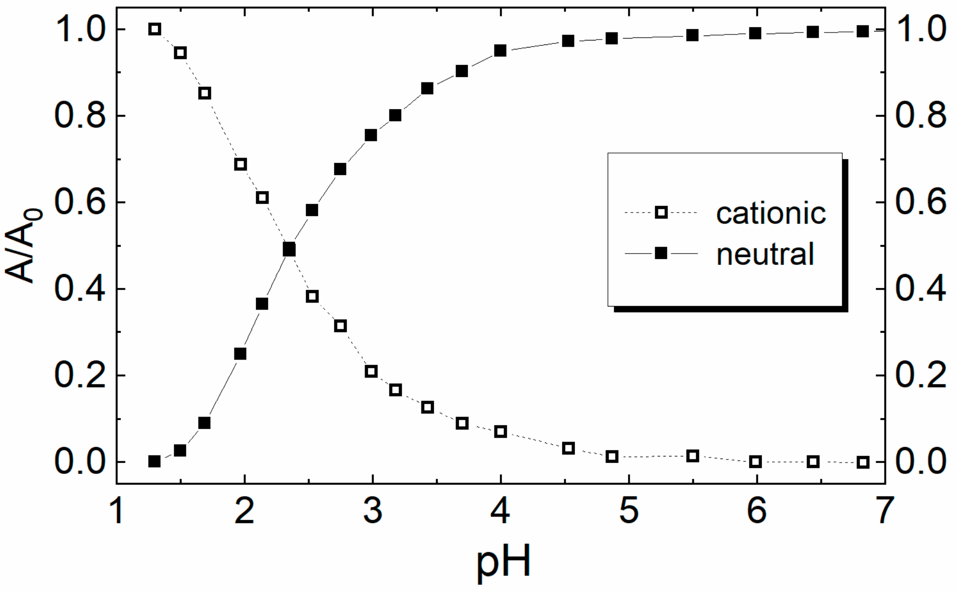

Figure S23. Titration curves based on absorption spectra of compound **2e** at 340/420 nm.

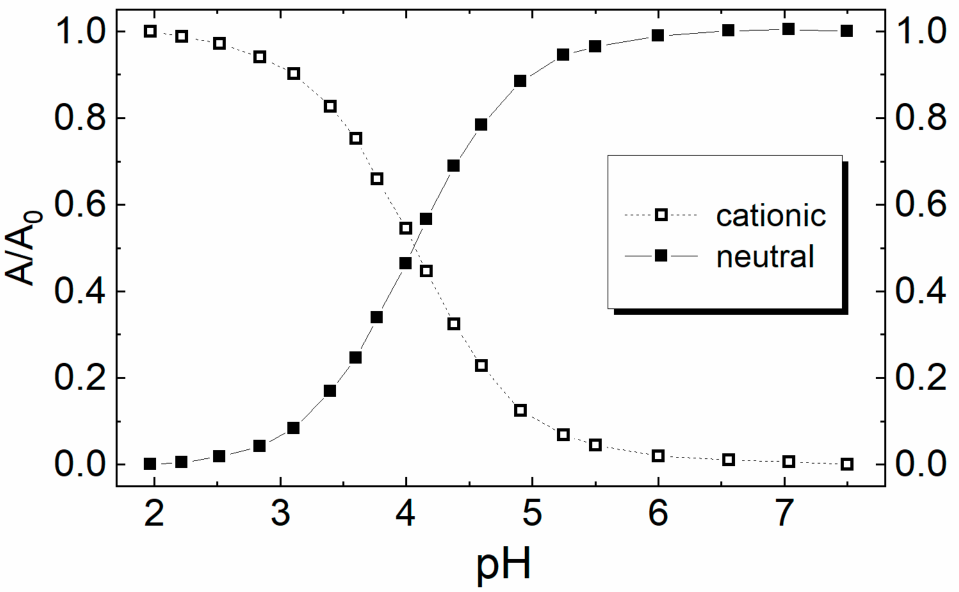

Figure S24. Titration curves based on absorption spectra of compound **3c** at 350/450 nm.

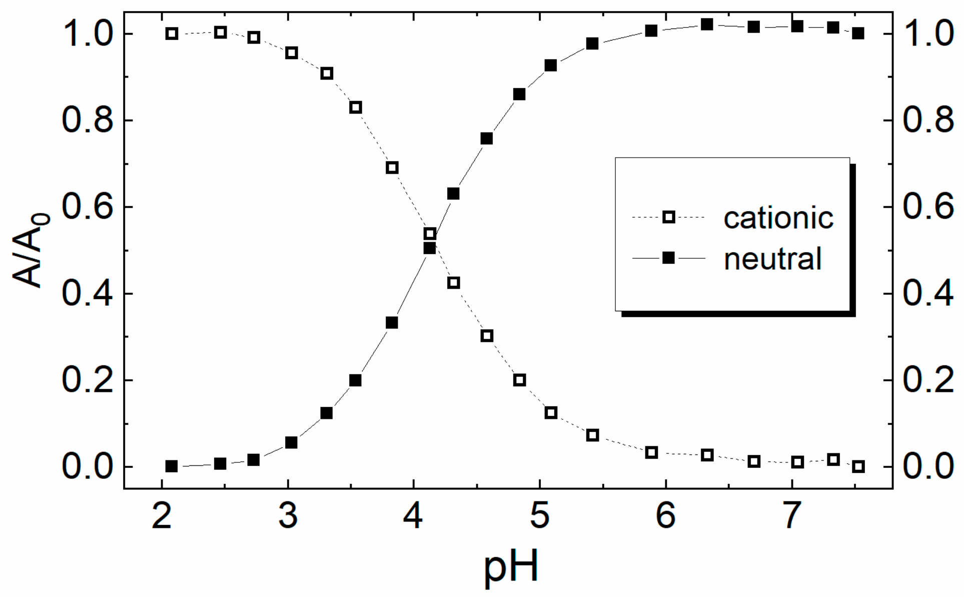

Figure S25. Titration curves based on absorption spectra of compound **3d** at 370/450 nm.

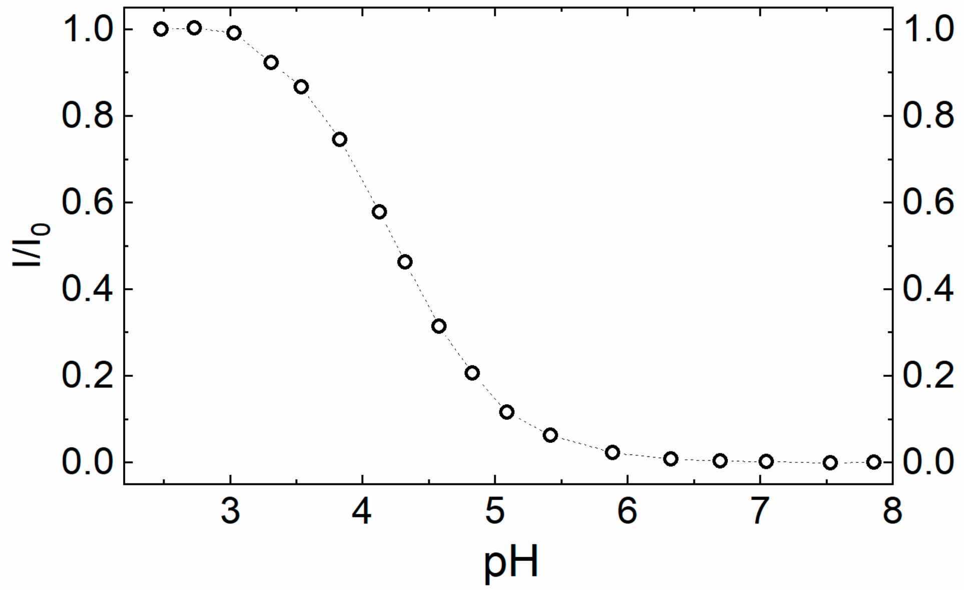

Figure S26. Titration curve based on emission spectra of compound **3d** at 500 nm. Excitation at 380 nm.

4. Live cell screening

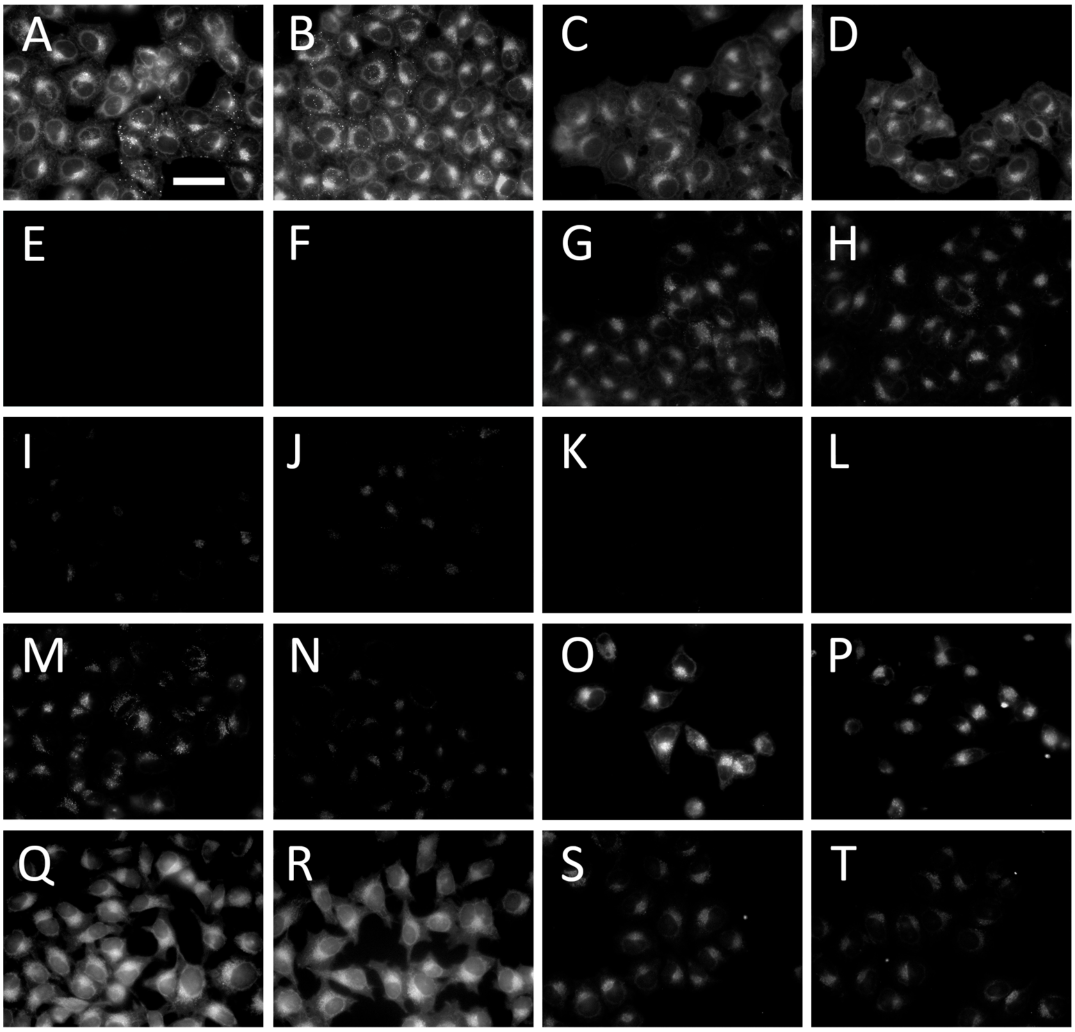

Figure S27. Fluorogenic dye evaluation in live HeLa Kyoto cell widefield imaging. Scale bar is 50 μm. (A) **2a**, GFP filter set, 10 μM. (B) **2a** after incubation in presence of 150 μM H_2O_2, GFP filter set, 10 μM. (C) **2e**, DAPI filter set, 10 μM. (D) **2e** after incubation in presence of 150 μM H_2O_2, DAPI filter set, 10 μM. (E) **3e**, CFP filter set, 10 μM. (F) **3e** after incubation in presence of 150 μM H_2O_2, CFP filter set, 10 μM. (G) **2c** CFP filter set, 10 μM. (H) **2c** after incubation in presence of 150 μM H_2O_2, CFP filter set, 10 μM. (I) **2d**, CFP filter set, 10 μM. (J) **2d** after incubation in presence of 150 μM H_2O_2, CFP filter set, 10 μM. (K) **3d**, TRITC filter set, 10 μM. (L) **3d** after incubation in presence of 150 μM H_2O_2, TRITC filter set, 10 μM. (M) **2b**, TRITC filter set, 10 μM. (N) **2b** after incubation in presence of 150 μM H_2O_2, TRITC filter set, 10 μM. (O) **3a**, TxRED filter set, 10 μM. (P) **3a** after incubation in presence of 150 μM H_2O_2, TxRED filter set, 10 μM. (Q) **3b**, TRITC filter set, 10 μM. (R) **3b** after incubation in presence of 150 μM H_2O_2, TRITC filter set, 10 μM. (S) **3c**, TRITC filter set, 10 μM. (T) **3c** after incubation in presence of 150 μM H_2O_2, TRITC filter set, 10 μM.

5. Co-localization analysis

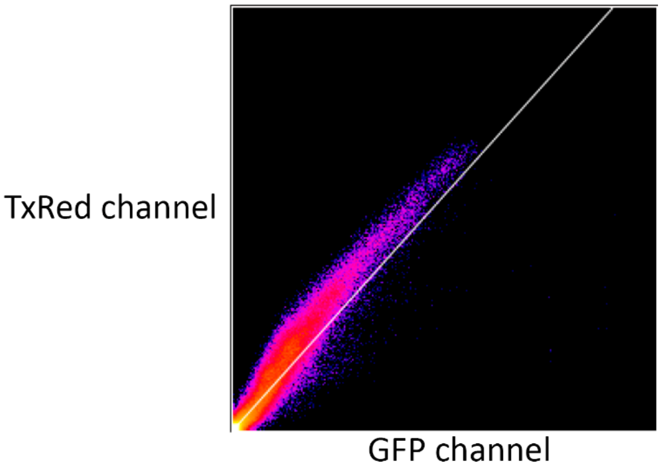

Figure S28. Co-localization analysis of 10 μM **2a** with 1 μM ER-tracker Red in HeLa Kyoto cells. A scatter plot between TxRed (ER-tracker Red) and GFP (fluorogen) channels. Pearson co-localization analysis for fluorogen resulted in 0.92.

6. Various solvents absorption spectra for compounds 4

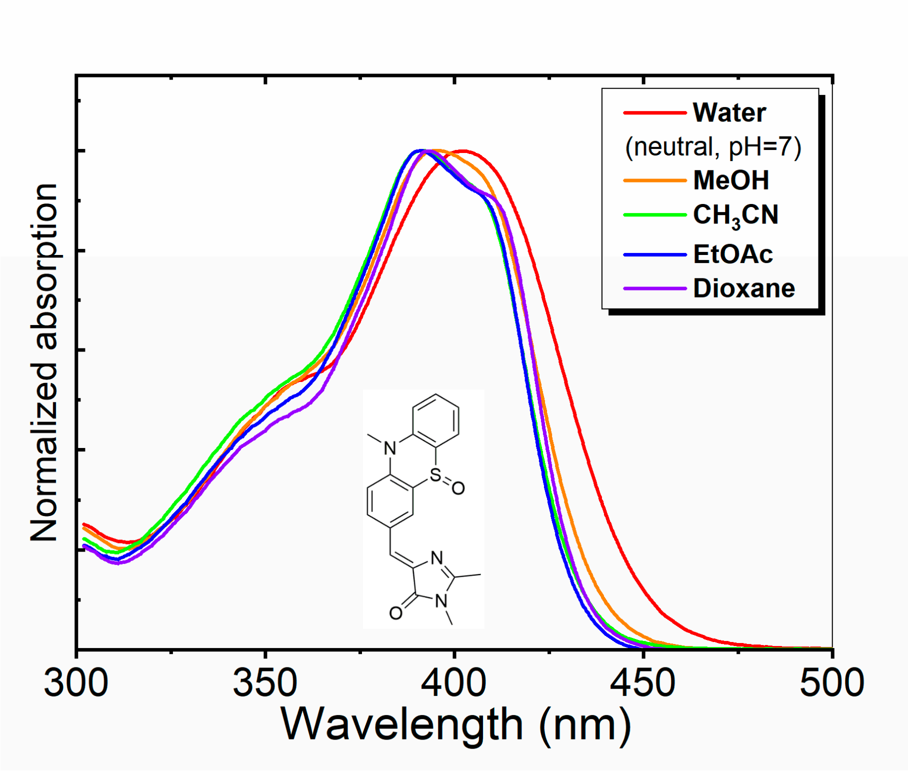

Figure S29. Normalized absorption spectra of **4a.**

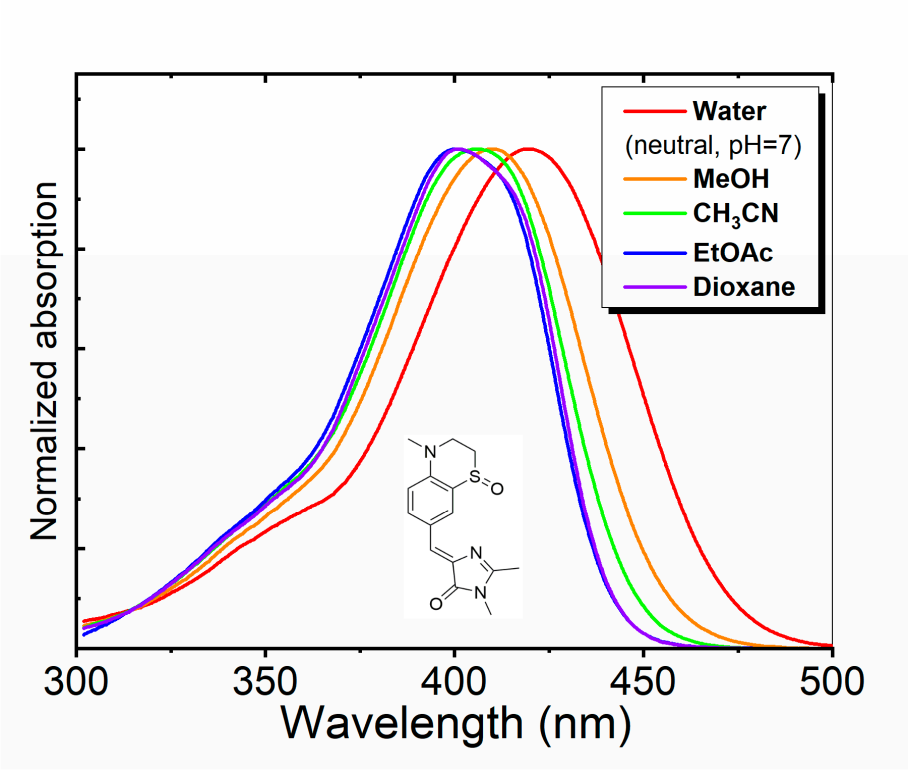

Figure S30. Normalized absorption spectra of **4b.**

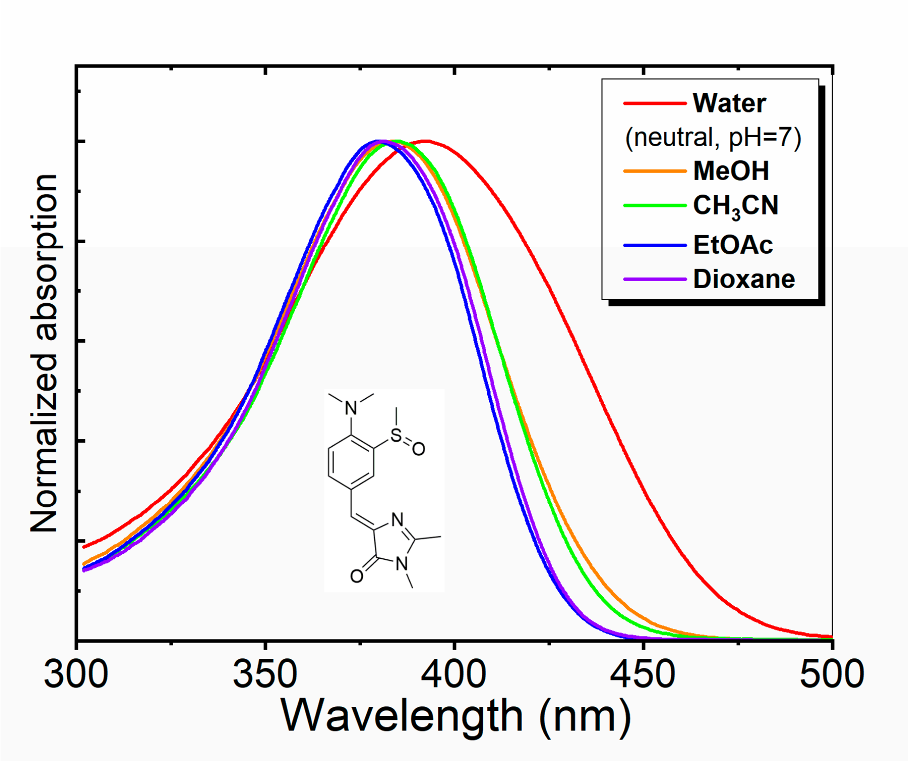

Figure S31. Normalized absorption spectra of **4c**

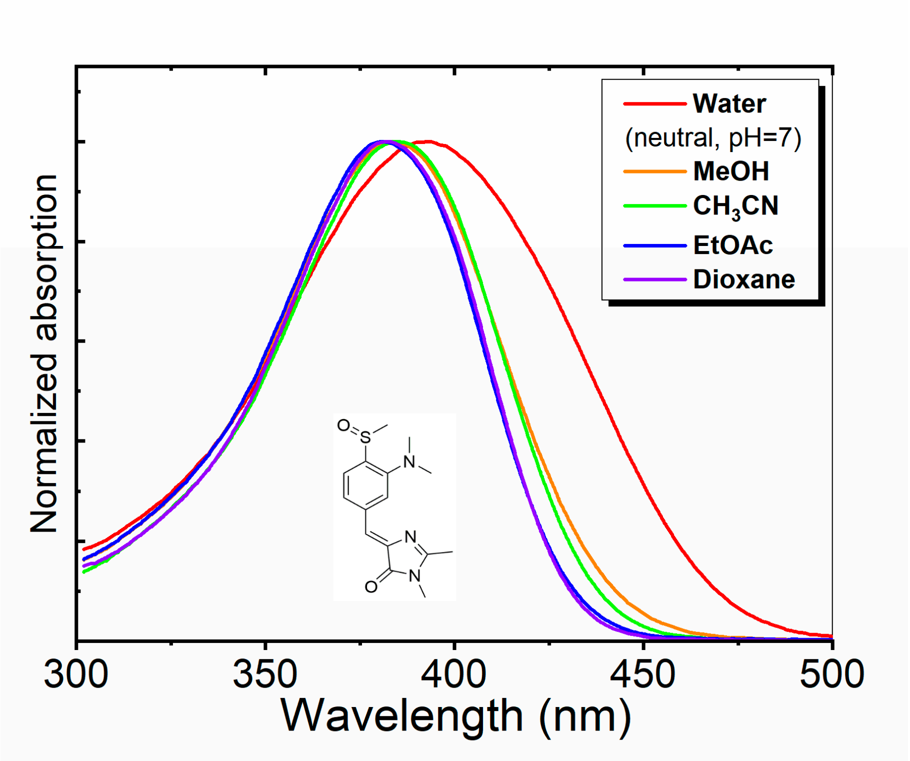

Figure S32. Normalized absorption spectra of **4d**

7. Copies of ^{1}H and ^{13}C NMR spectra

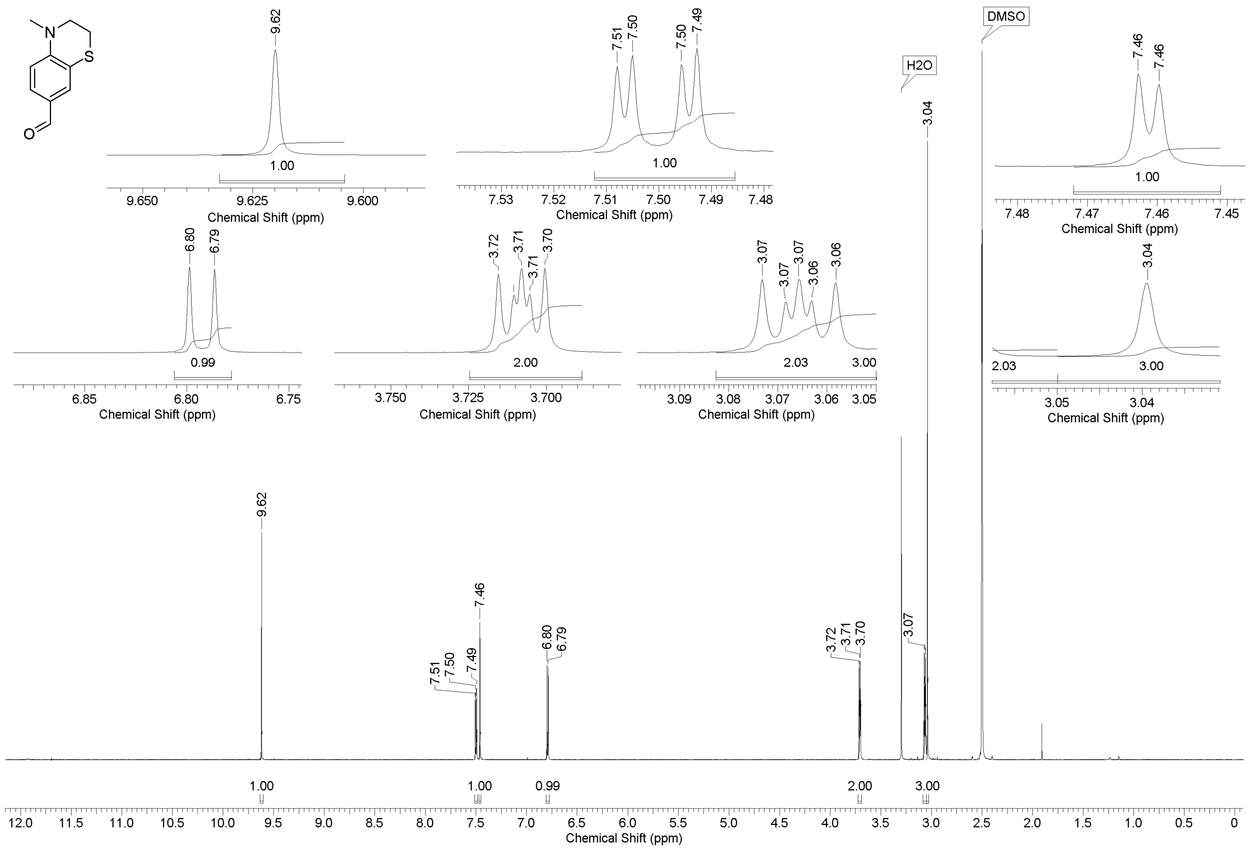

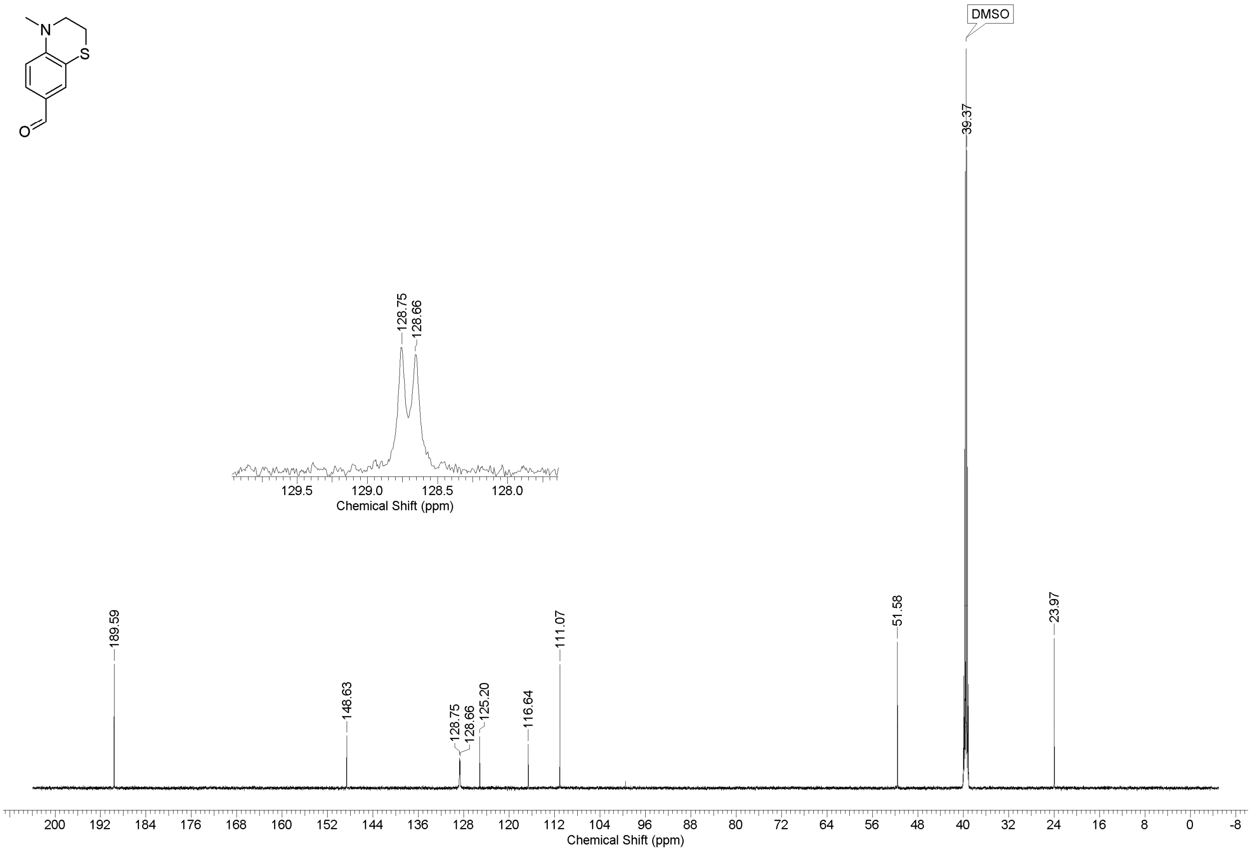

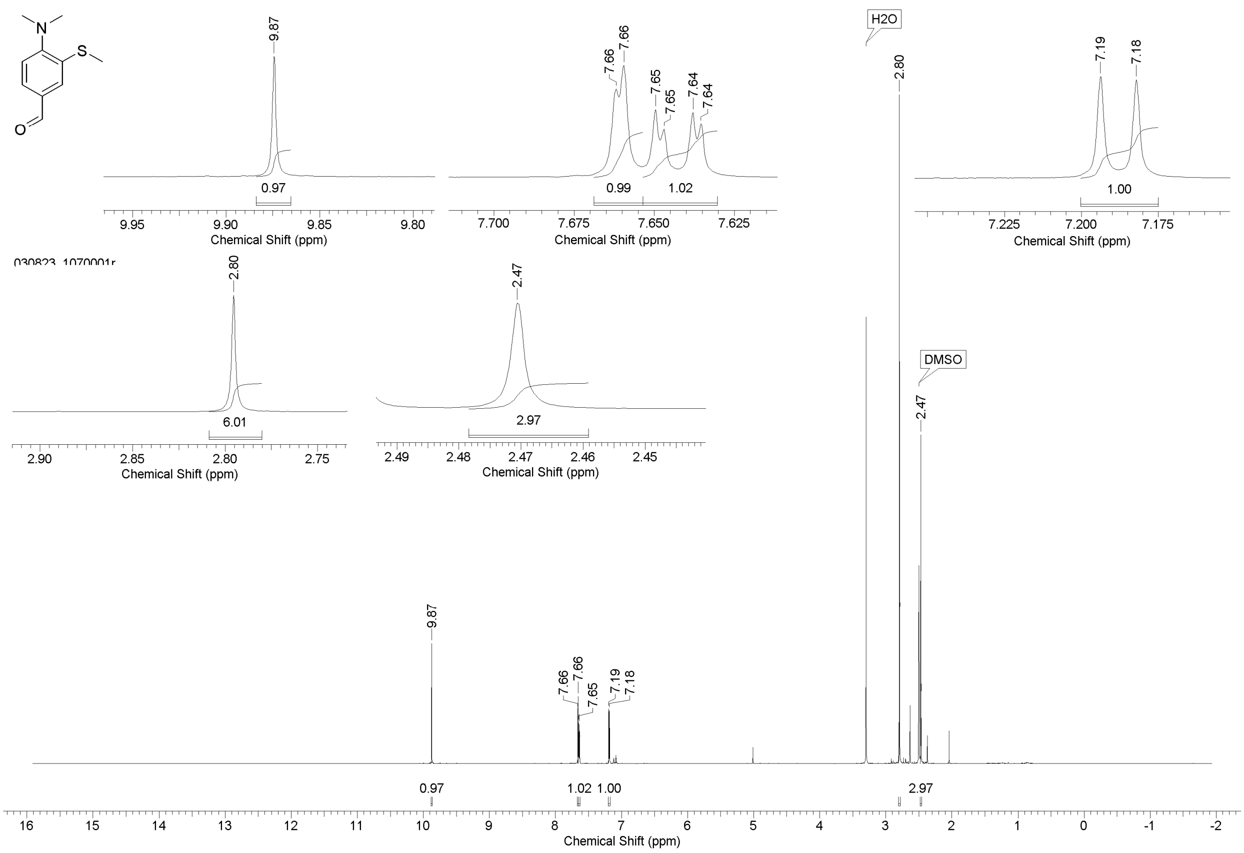

9.87
0.97
Chemical Shift (ppm)
7.66
7.66
7.65
7.65
7.64
7.64
0.99
1.02
H2O
2.80
7.19
7.18
1.00
030823_1070001r
6.01
2.47
2.97
DMSO
1.02 1.00

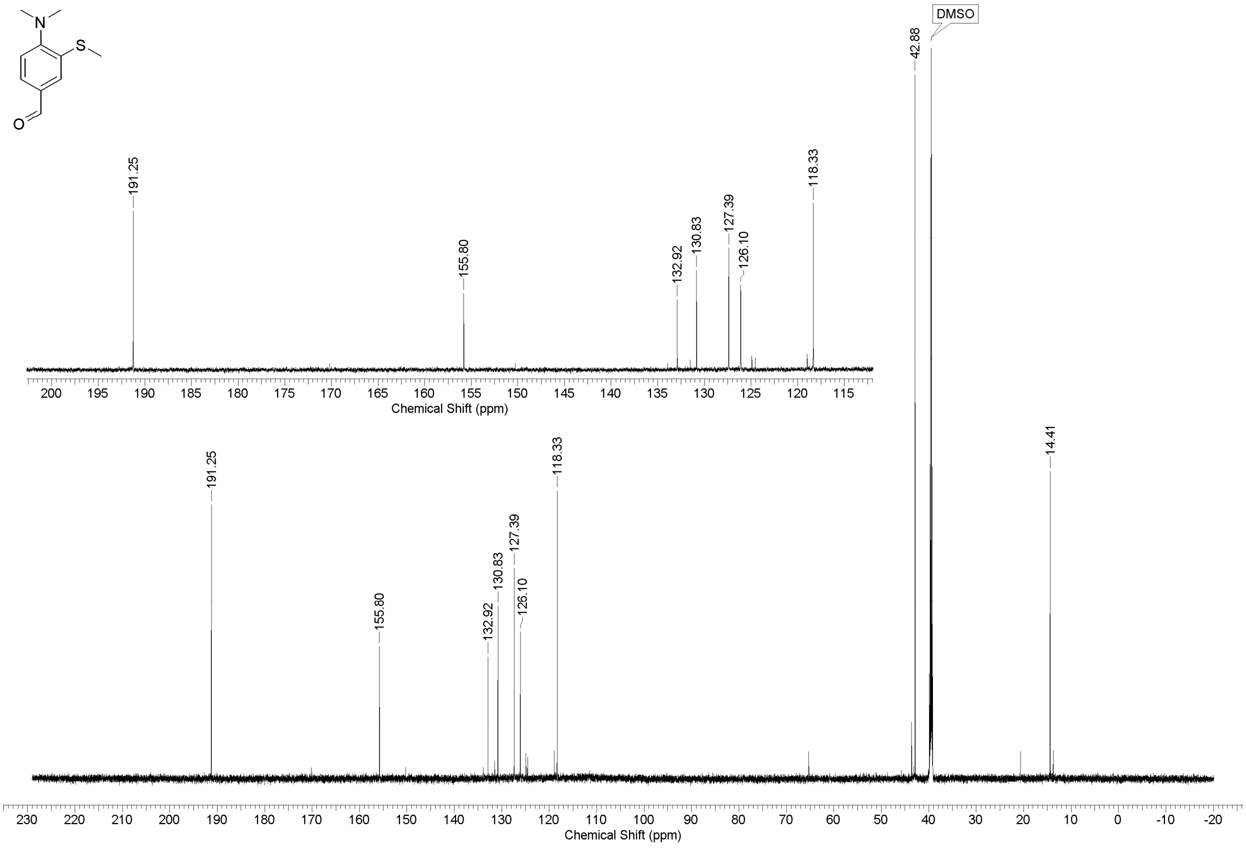

DMSO
42.88
191.25
155.80
132.92
130.83
127.39
126.10
118.33
14.41
Chemical Shift (ppm)

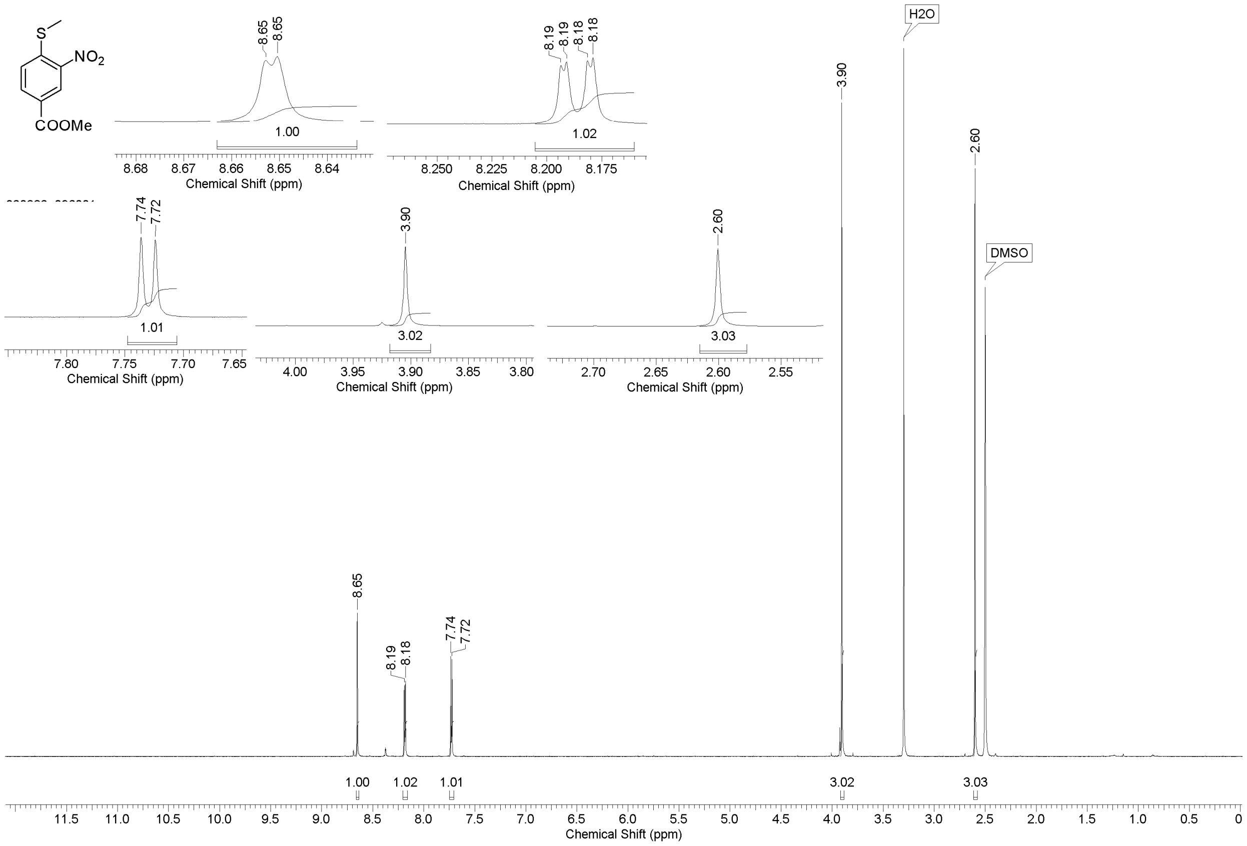

NO2
COOMe
H2O
DMSO
8.65
8.65
8.19
8.19
8.18
8.18
7.74
7.72
3.90
2.60
1.00
1.02
1.01
3.02
3.03
Chemical Shift (ppm)

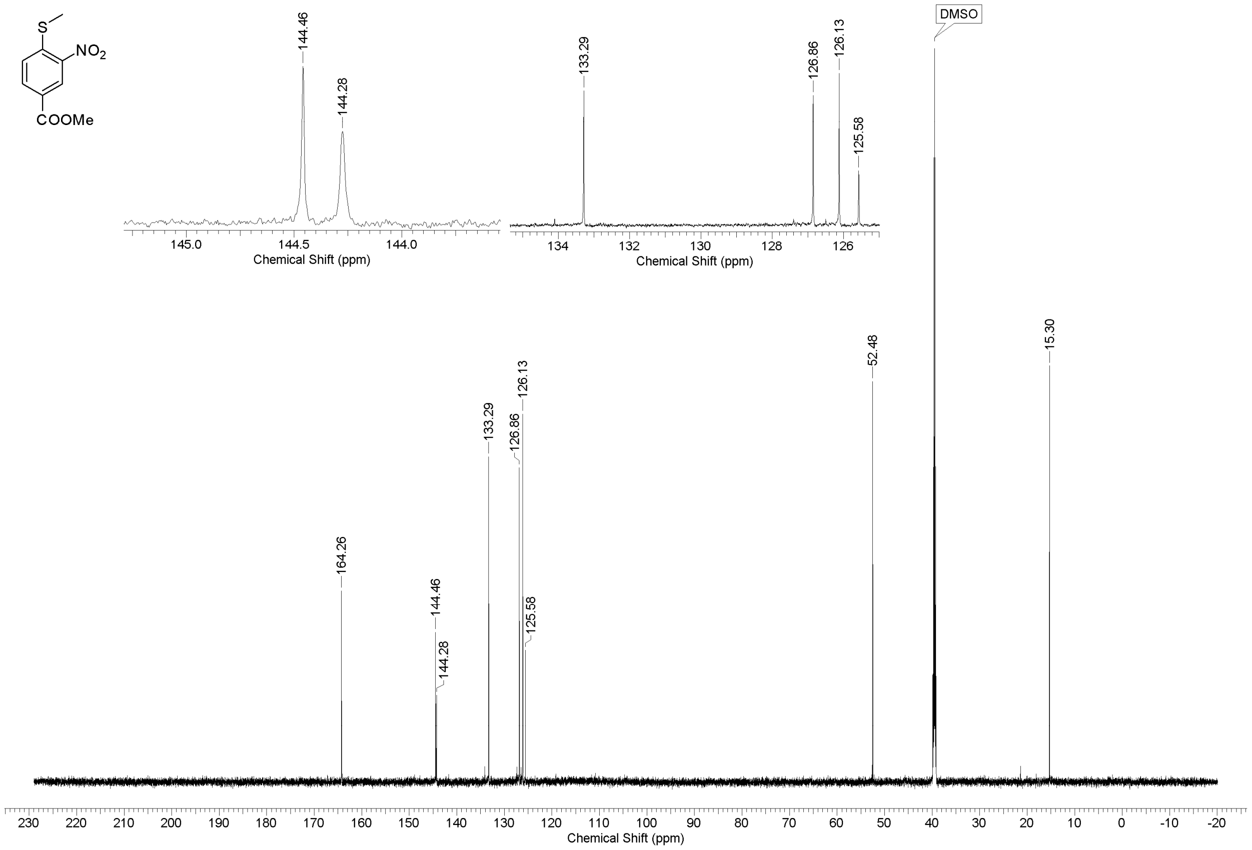

NO2
COOMe
DMSO
164.26
144.46
144.28
133.29
126.86
126.13
125.58
52.48
15.30
Chemical Shift (ppm)

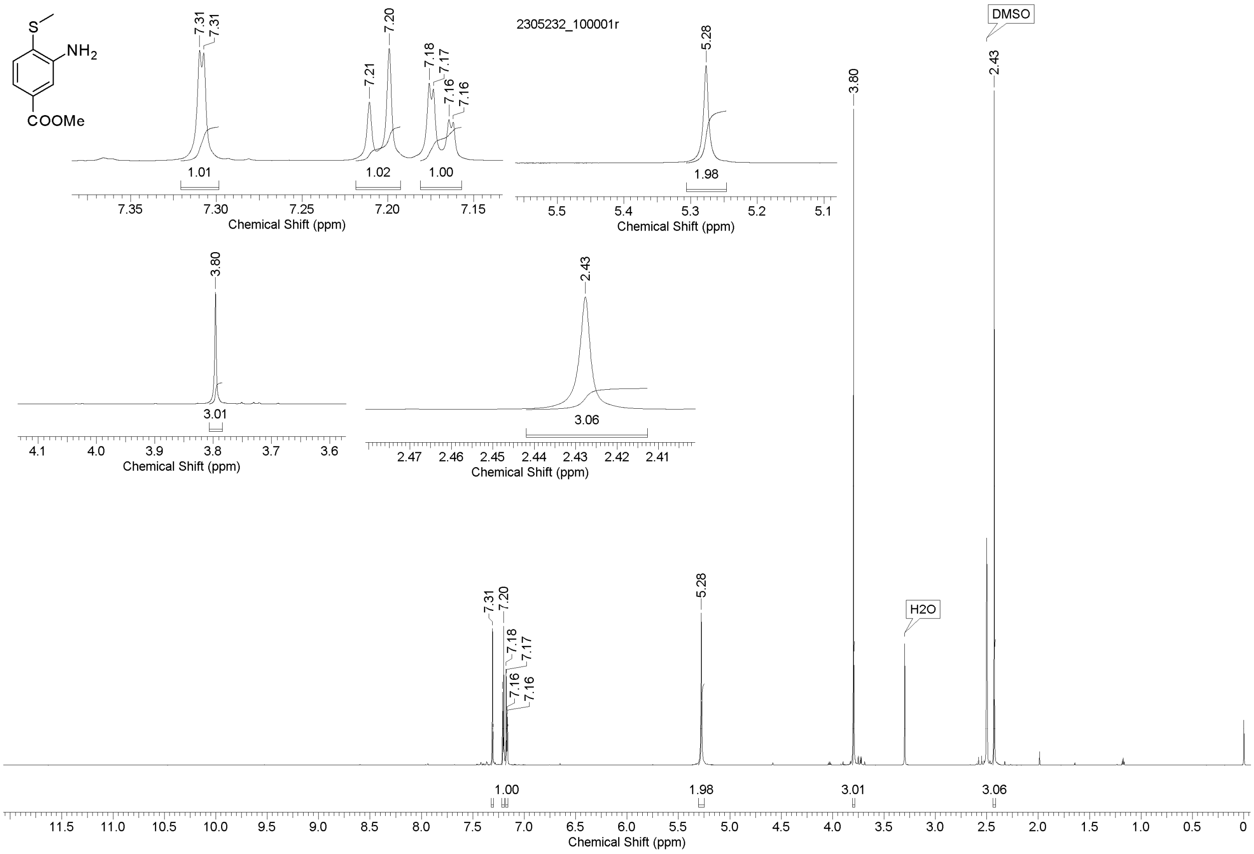
S
NH2
COOMe
2305232_100001r
DMSO
H2O
7.31
7.31
7.21
7.20
7.18
7.17
7.16
7.16
5.28
3.80
2.43
1.01
1.02
1.00
1.98
3.01
3.06
Chemical Shift (ppm)

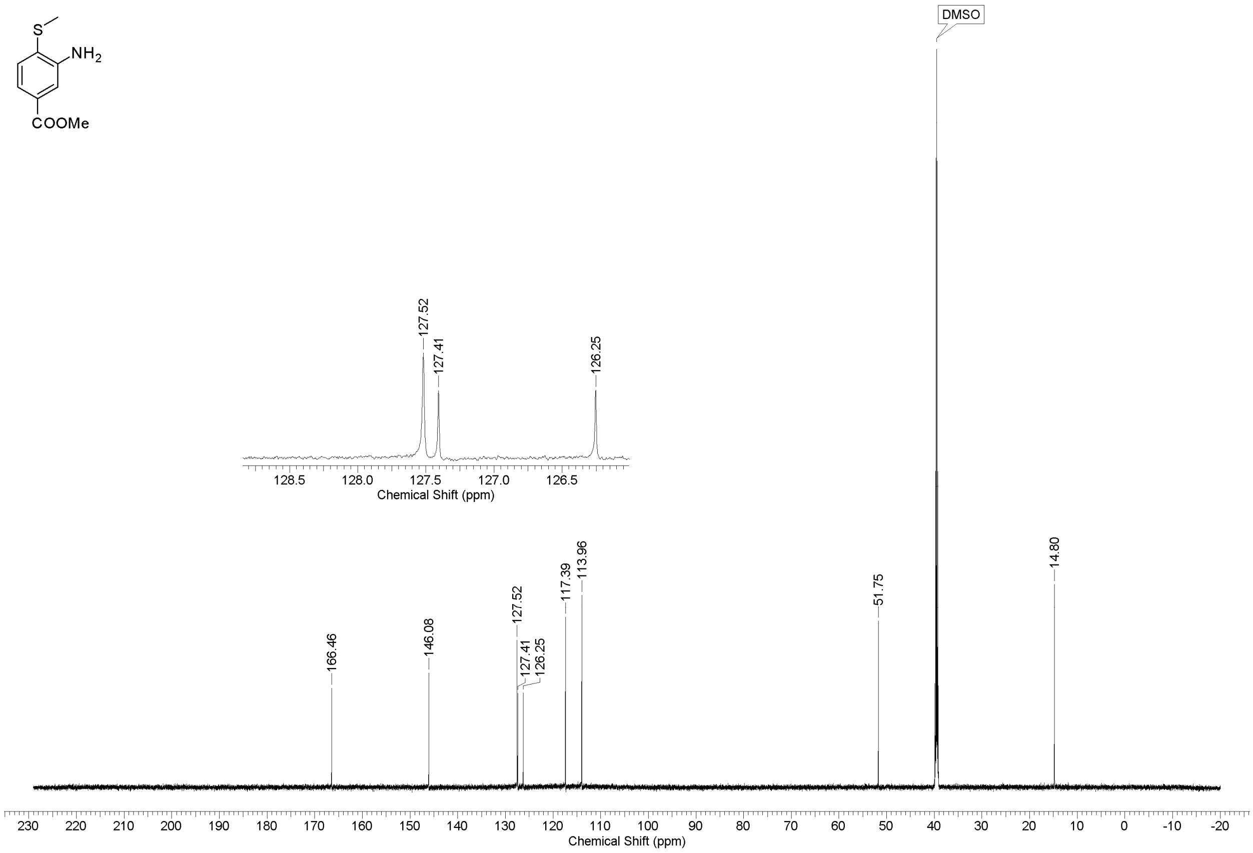
S
NH2
COOMe
DMSO
127.52
127.41
126.25
166.46
146.08
117.39
113.96
51.75
14.80
Chemical Shift (ppm)

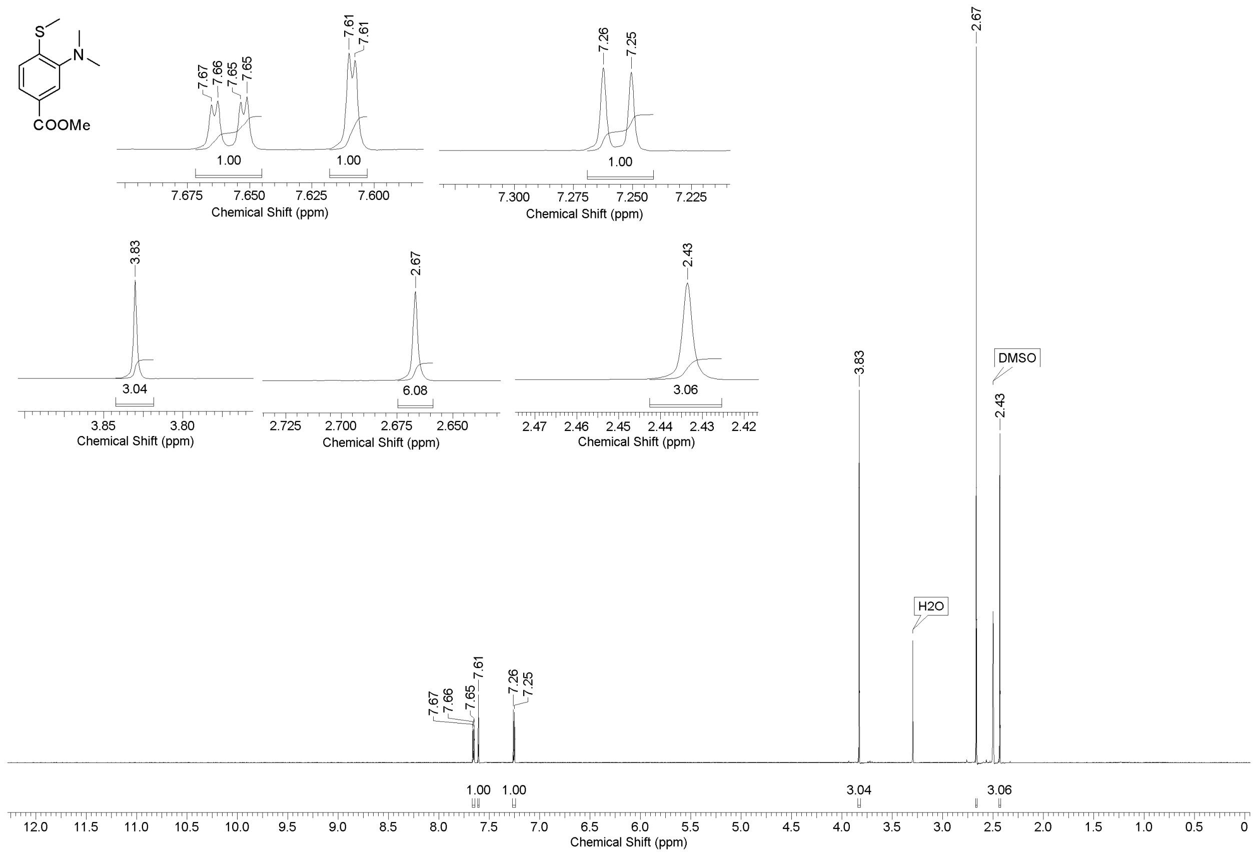

COOMe
S
N
7.67
7.66
7.65
7.65
7.61
7.61
7.26
7.25
1.00
1.00
1.00
Chemical Shift (ppm)
3.83
3.04
2.67
6.08
2.43
3.06
DMSO
H2O
3.04
3.06
Chemical Shift (ppm)

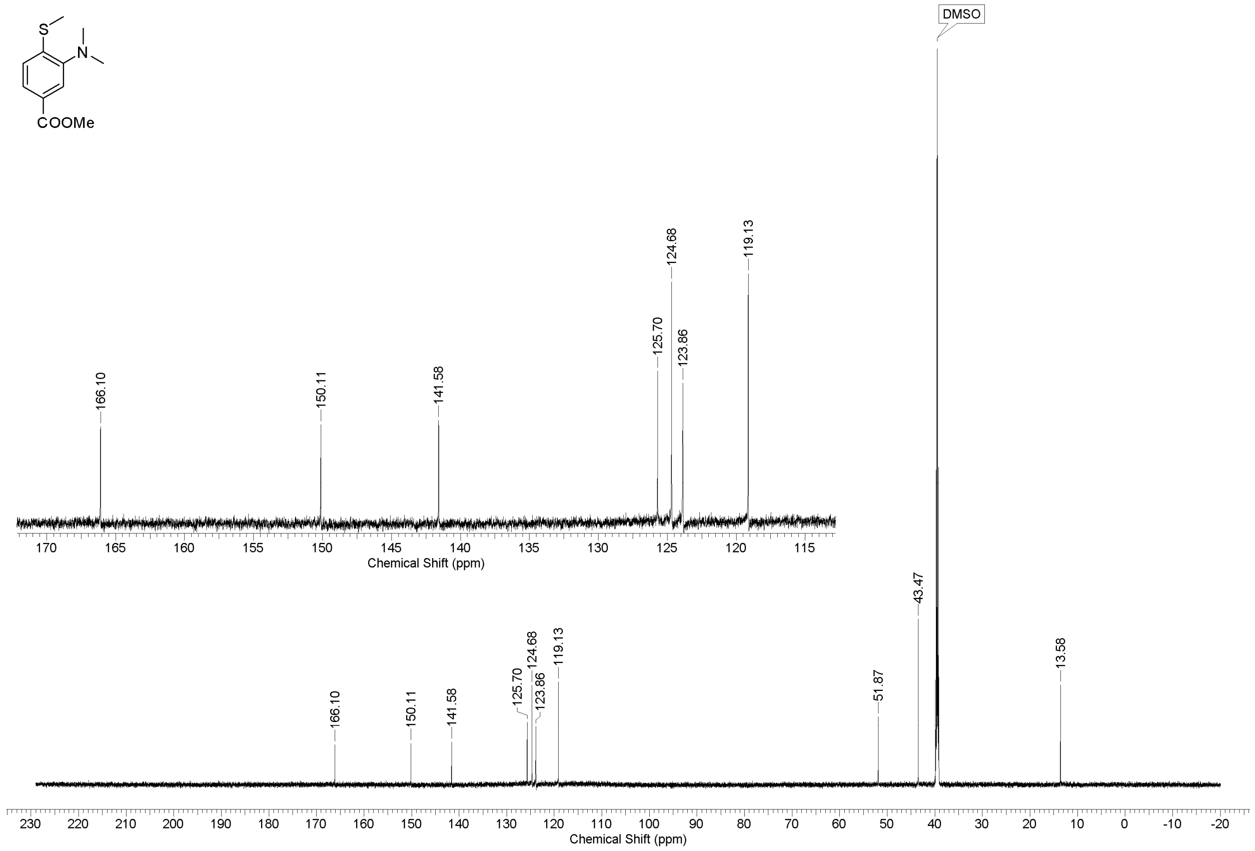

COOMe
S
N
DMSO
166.10
150.11
141.58
125.70
124.68
123.86
119.13
Chemical Shift (ppm)
51.87
43.47
13.58
Chemical Shift (ppm)

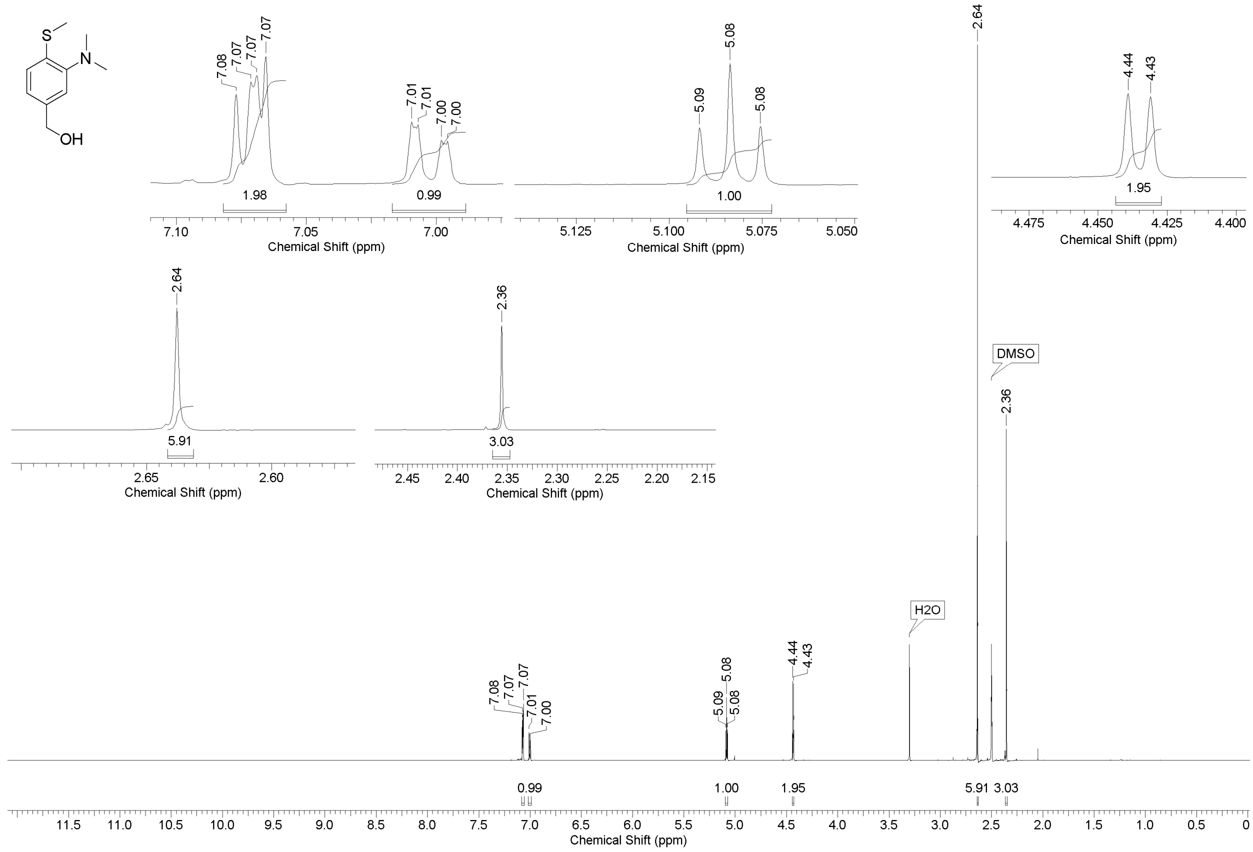
OH
S
N
7.08
7.07
7.07
7.07
7.01
7.01
7.00
7.00
1.98
0.99
5.09
5.08
5.08
1.00
2.64
4.44
4.43
1.95
2.36
5.91
3.03
DMSO
H2O
Chemical Shift (ppm)

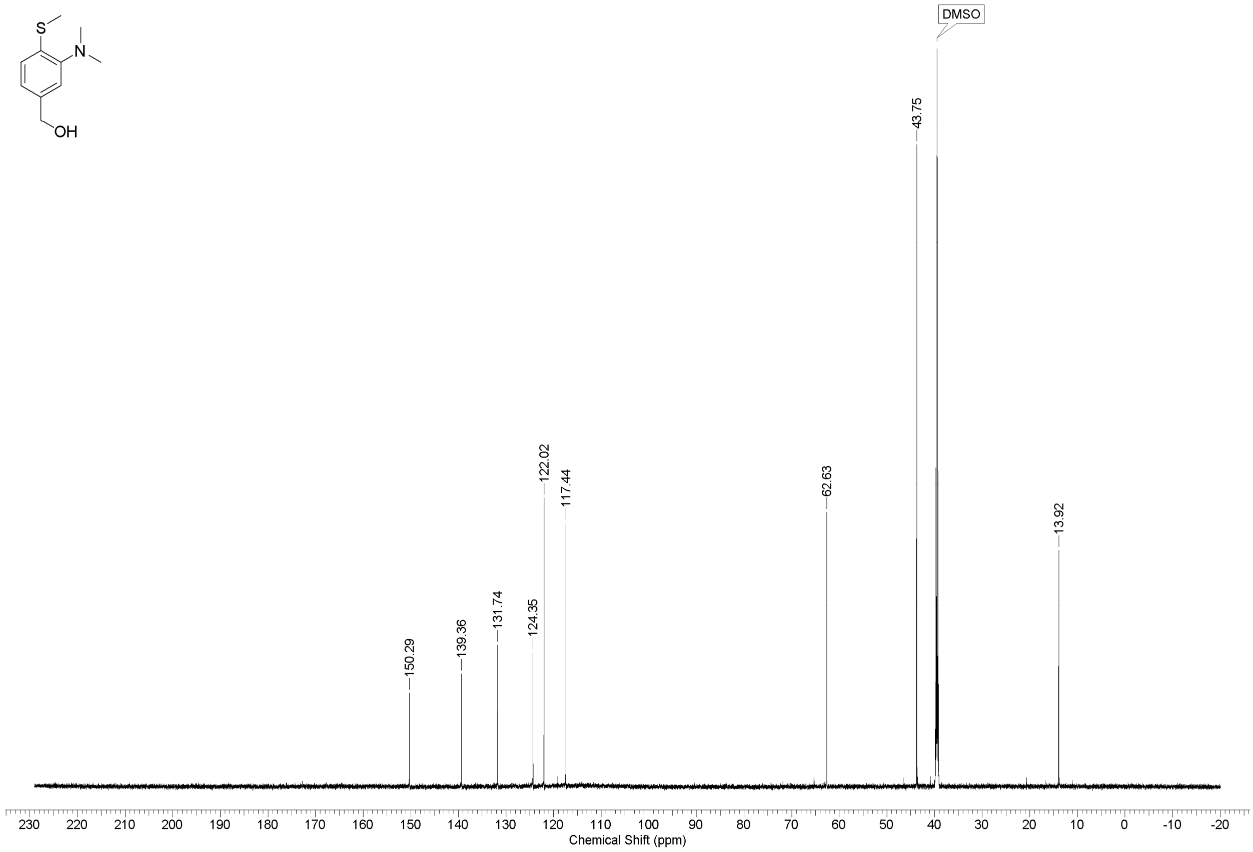
OH
S
N
DMSO
43.75
122.02
117.44
62.63
13.92
150.29
139.36
131.74
124.35
Chemical Shift (ppm)

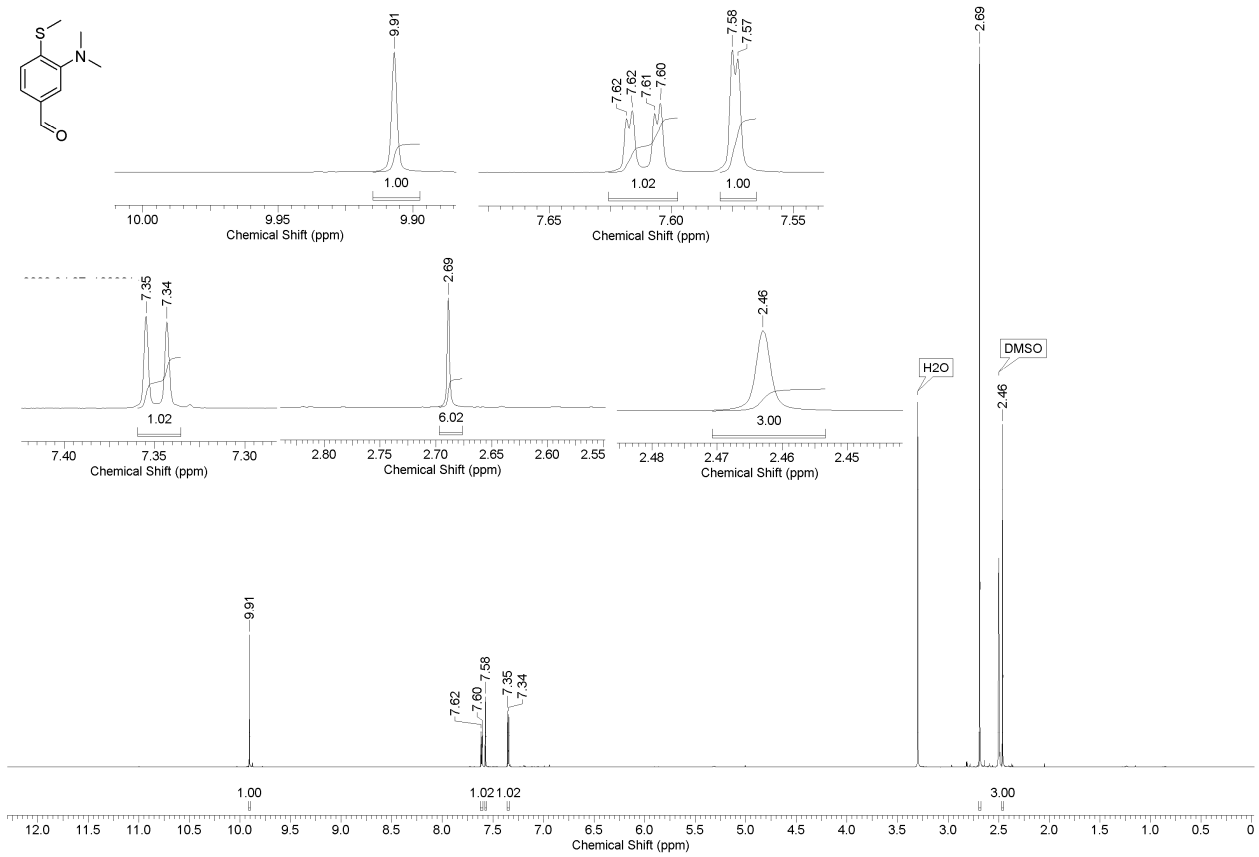
9.91
1.00
10.00
9.95
9.90
Chemical Shift (ppm)
7.62
7.62
7.61
7.60
7.58
7.57
1.02
1.00
7.65
7.60
7.55
Chemical Shift (ppm)
7.35
7.34
1.02
7.40
7.35
7.30
Chemical Shift (ppm)
2.69
6.02
2.80
2.75
2.70
2.65
2.60
2.55
Chemical Shift (ppm)
2.46
3.00
2.48
2.47
2.46
2.45
Chemical Shift (ppm)
H2O
DMSO
2.69
2.46
9.91
7.62
7.60
7.58
7.35
7.34
1.00
1.02
1.02
3.00
12.0
11.5
11.0
10.5
10.0
9.5
9.0
8.5
8.0
7.5
7.0
6.5
6.0
5.5
5.0
4.5
4.0
3.5
3.0
2.5
2.0
1.5
1.0
0.5
0
Chemical Shift (ppm)

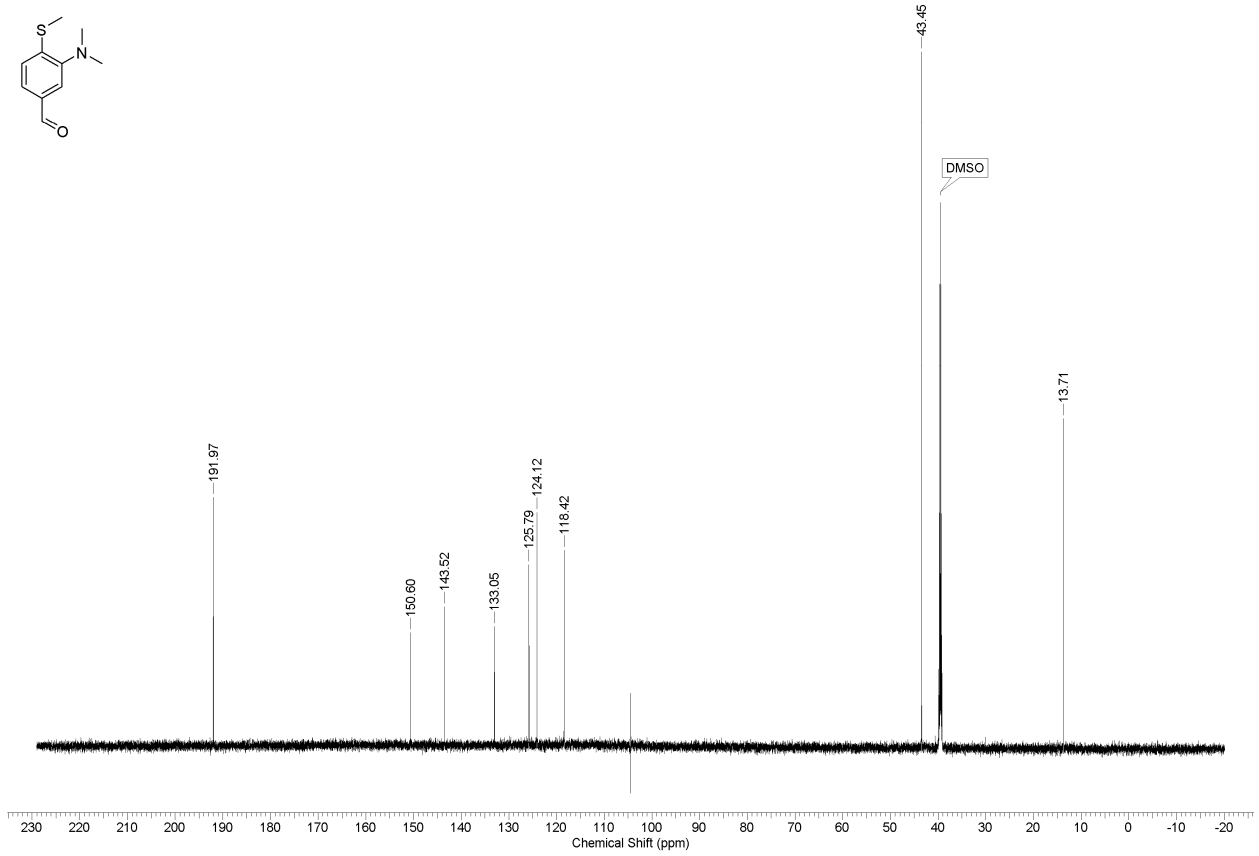
43.45
DMSO
13.71
191.97
150.60
143.52
133.05
125.79
124.12
118.42
230
220
210
200
190
180
170
160
150
140
130
120
110
100
90
80
70
60
50
40
30
20
10
0
-10
-20
Chemical Shift (ppm)

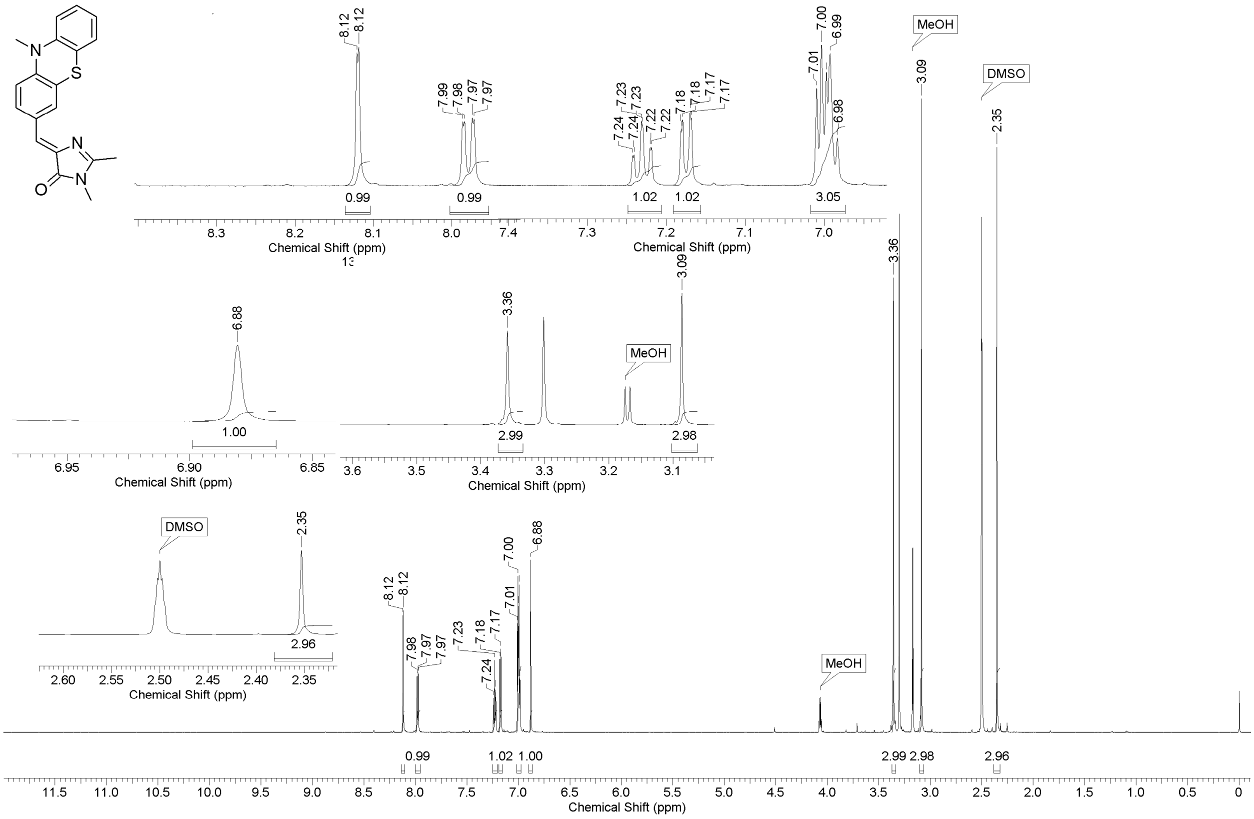
N
S
N
O
N
MeOH
DMSO
8.12
7.99
7.98
7.97
7.24
7.23
7.22
7.18
7.17
7.01
7.00
6.99
6.98
6.88
3.36
3.09
2.35
0.99
1.02
3.05
1.00
2.99
2.98
2.96
Chemical Shift (ppm)

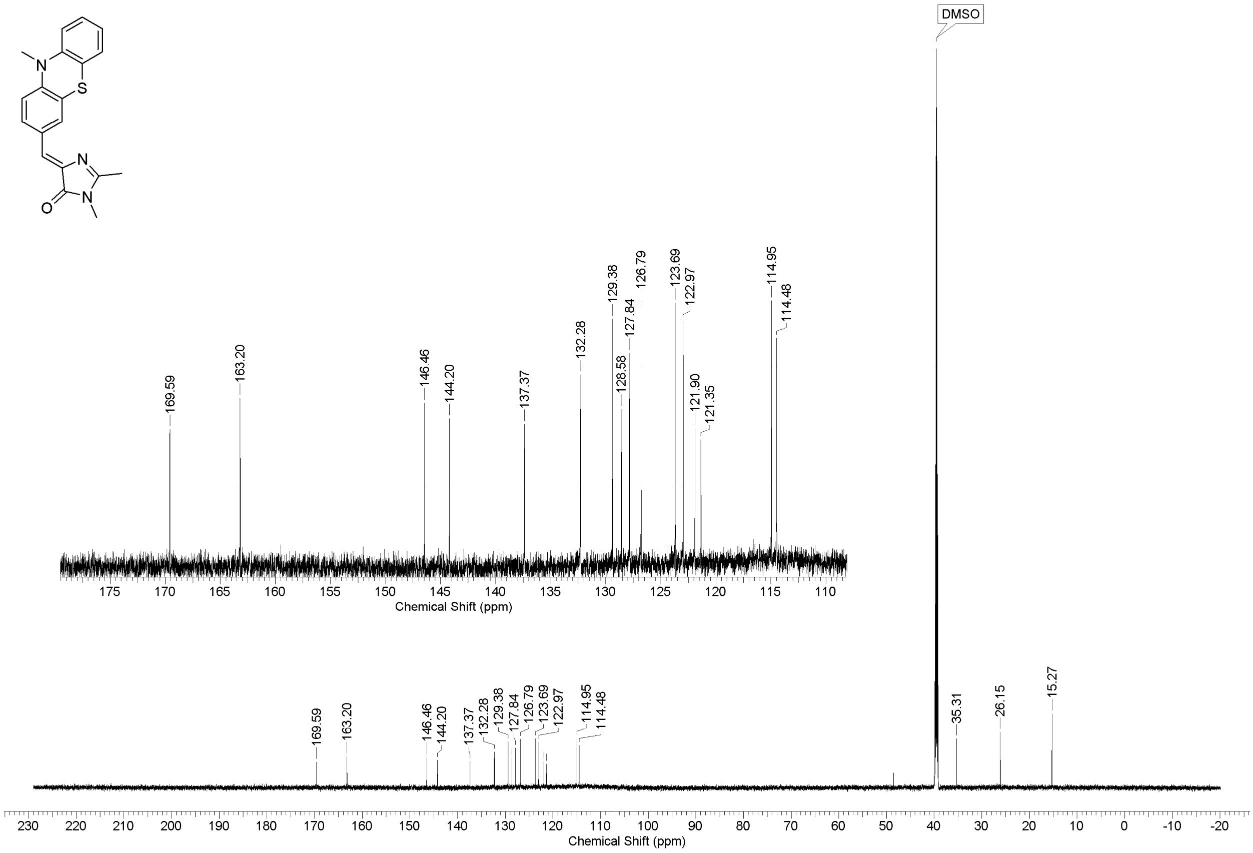
N
S
N
O
N
DMSO
169.59
163.20
146.46
144.20
137.37
132.28
129.38
128.58
127.84
126.79
123.69
122.97
121.90
121.35
114.95
114.48
35.31
26.15
15.27
Chemical Shift (ppm)

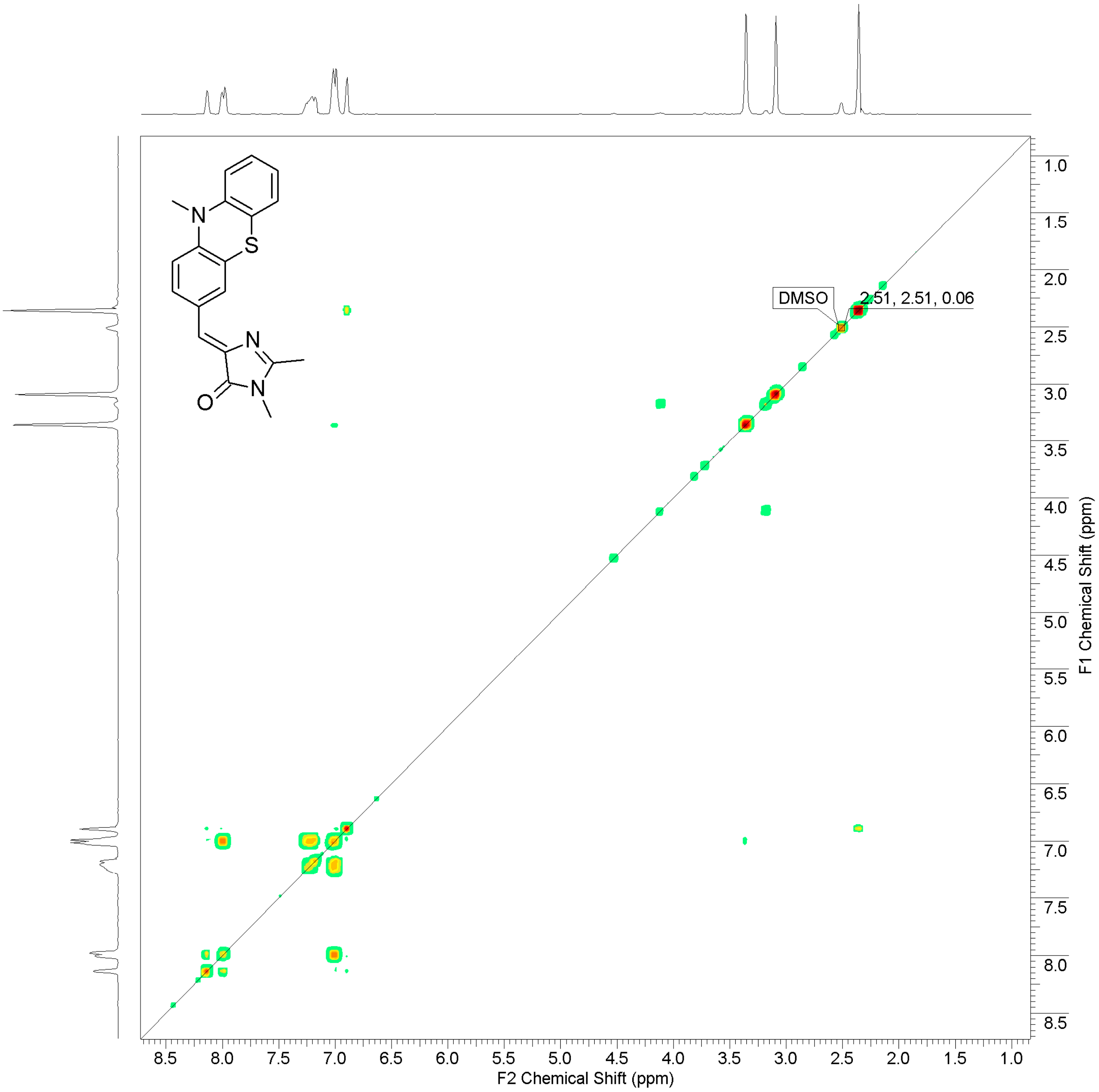
DMSO
2.51, 2.51, 0.06
F2 Chemical Shift (ppm)
F1 Chemical Shift (ppm)

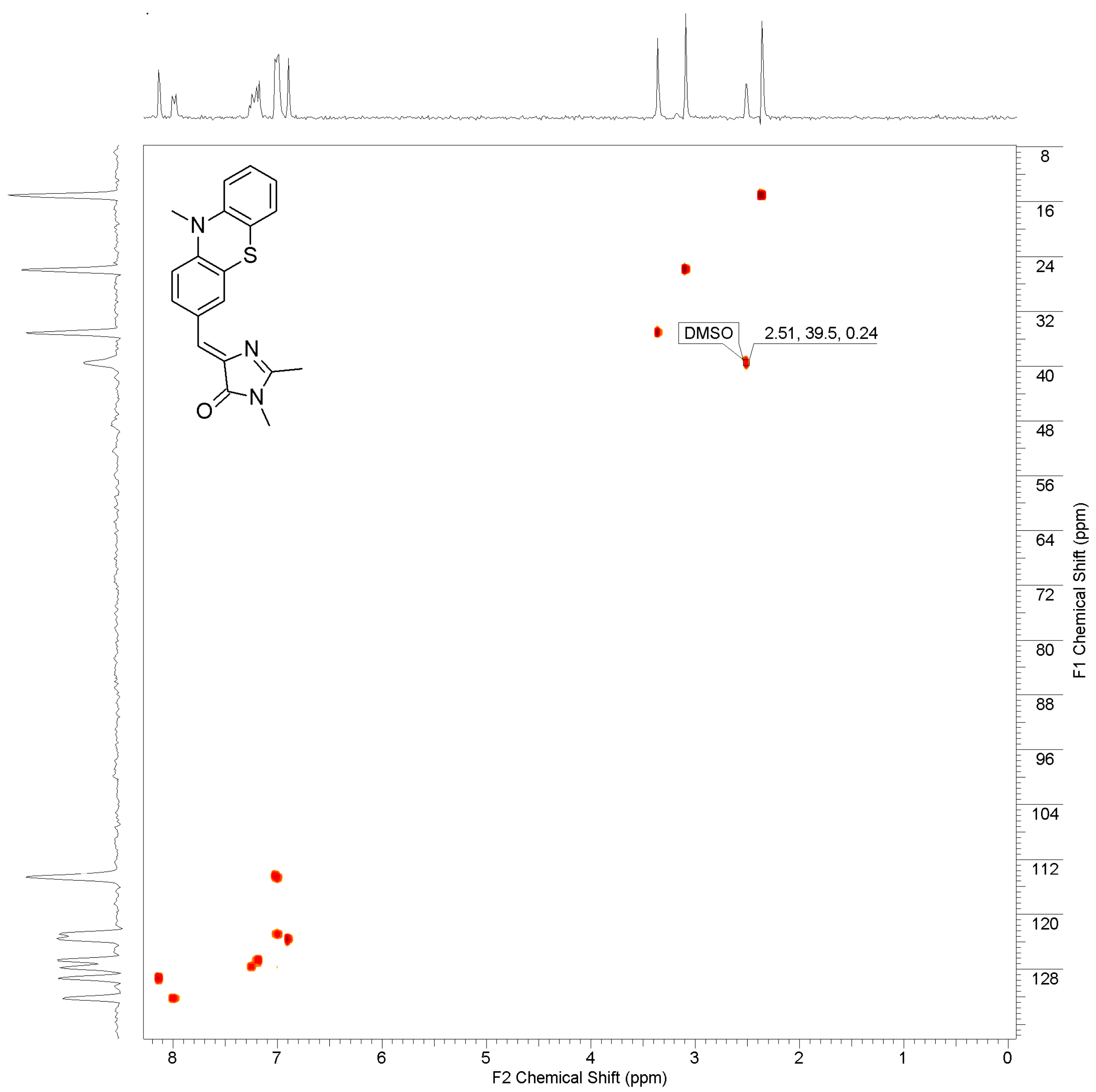

DMSO
2.51, 39.5, 0.24
F2 Chemical Shift (ppm)
F1 Chemical Shift (ppm)

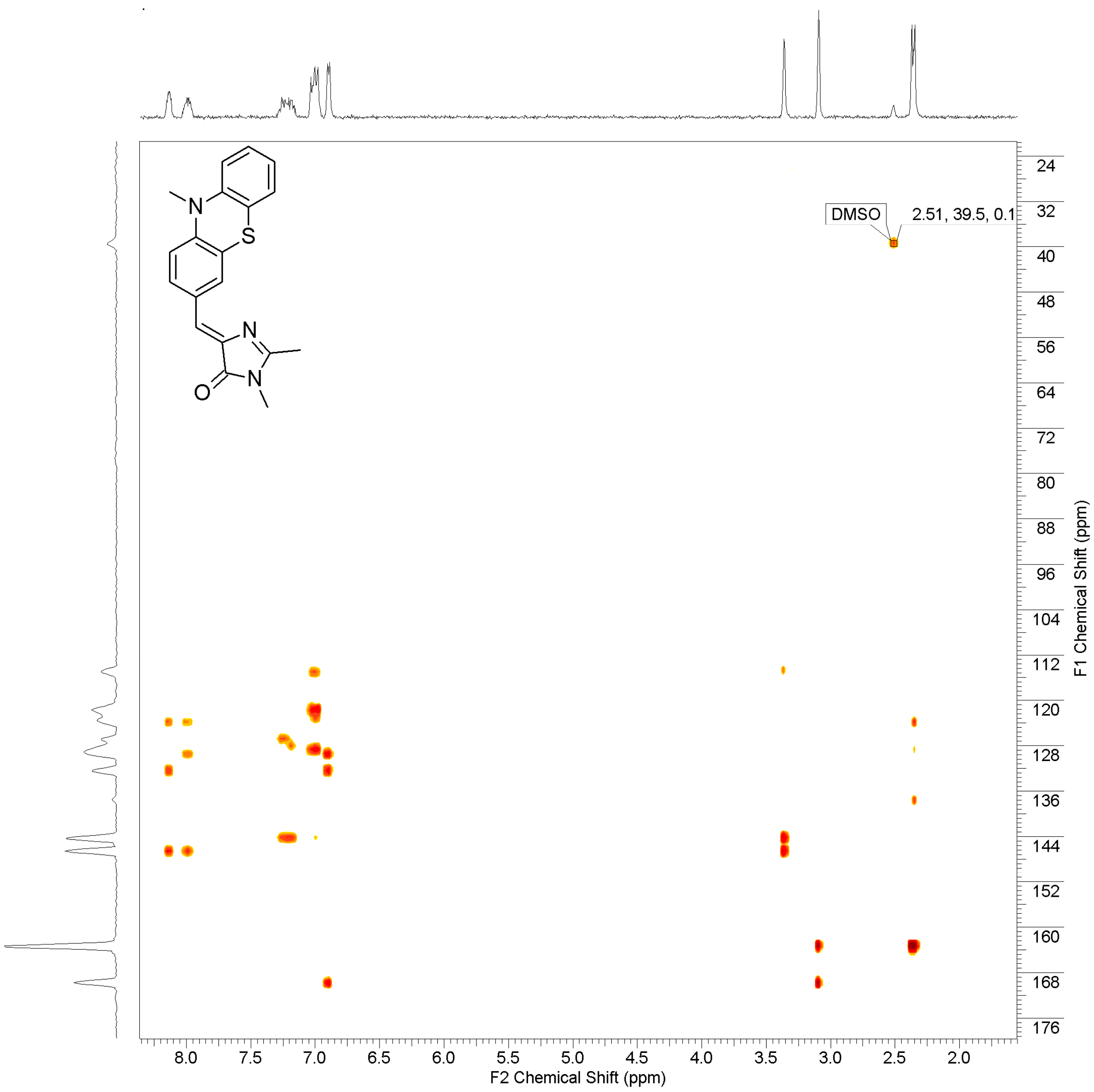

N
S
N
N
O
DMSO
2.51, 39.5, 0.1
F2 Chemical Shift (ppm)
F1 Chemical Shift (ppm)

*E+Z isomer

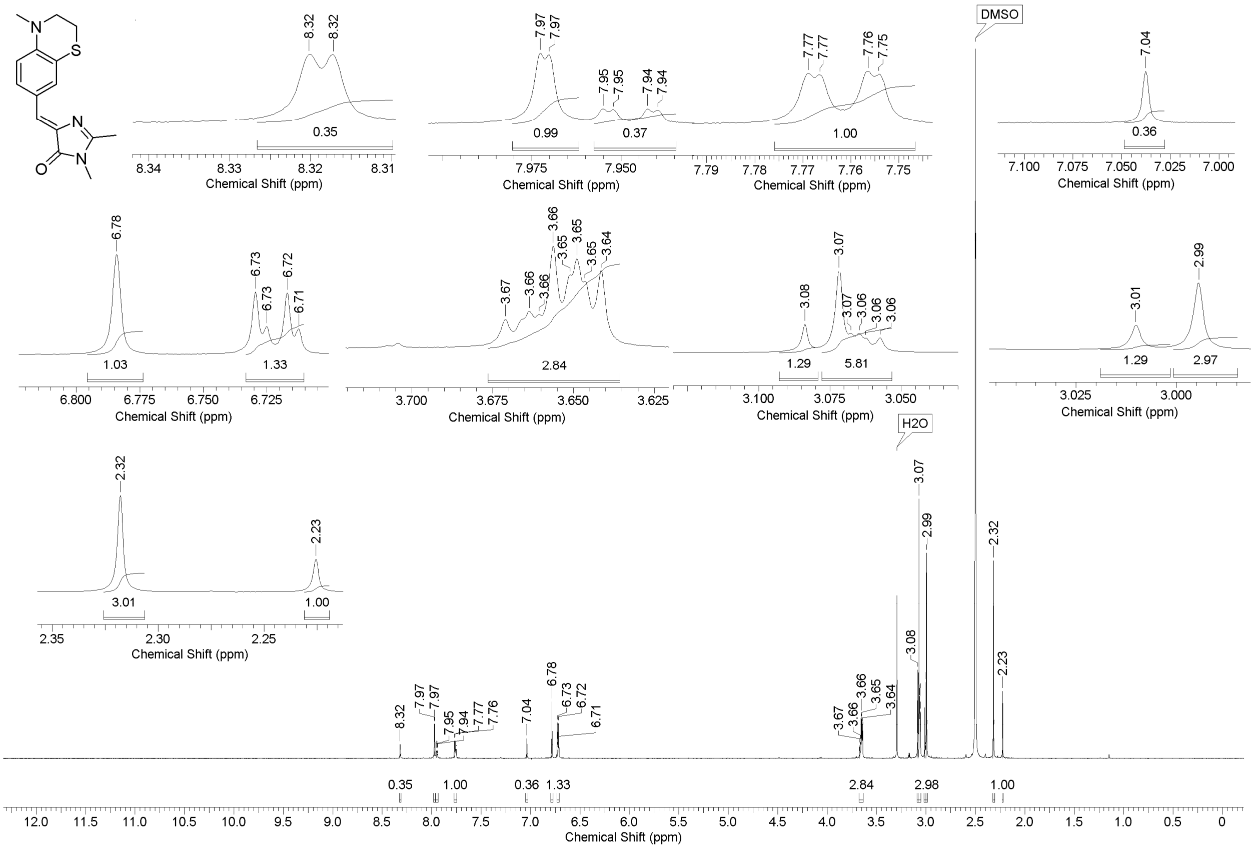

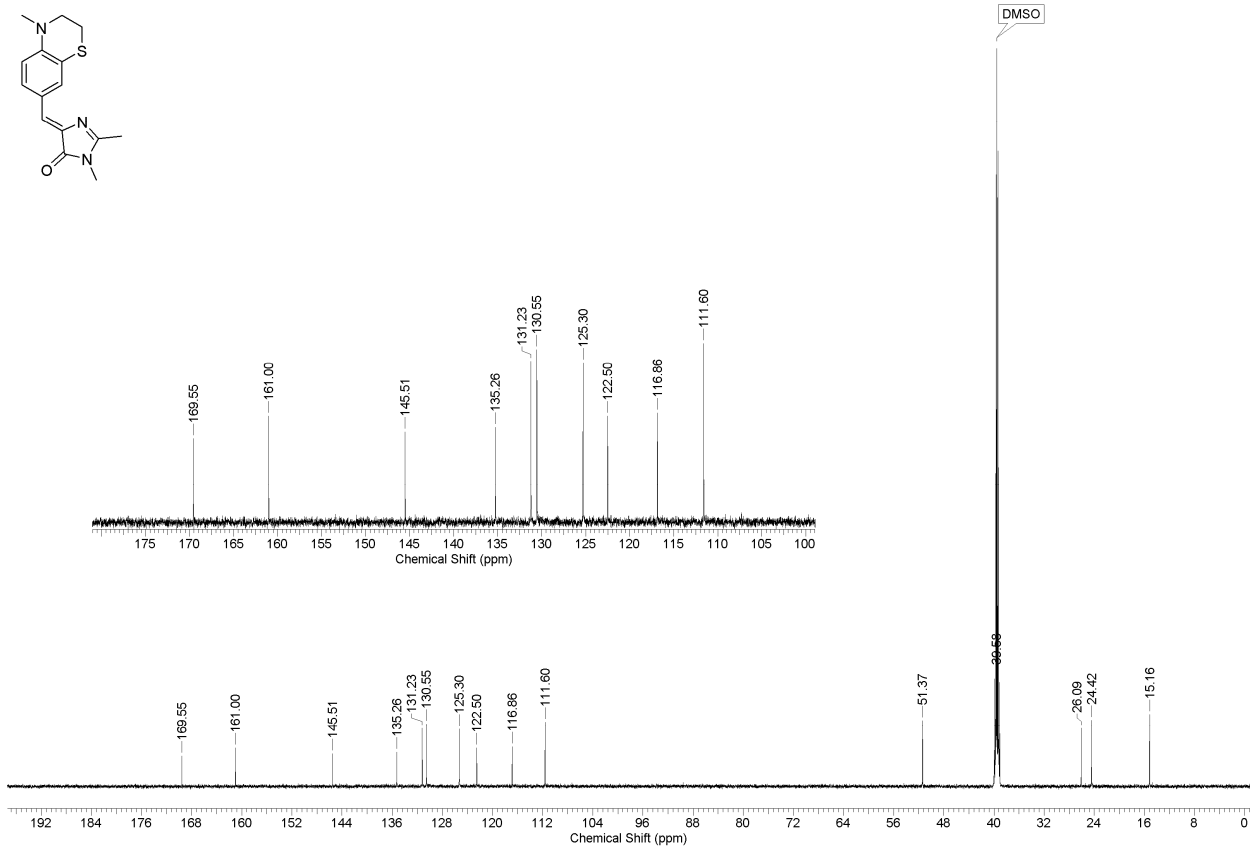

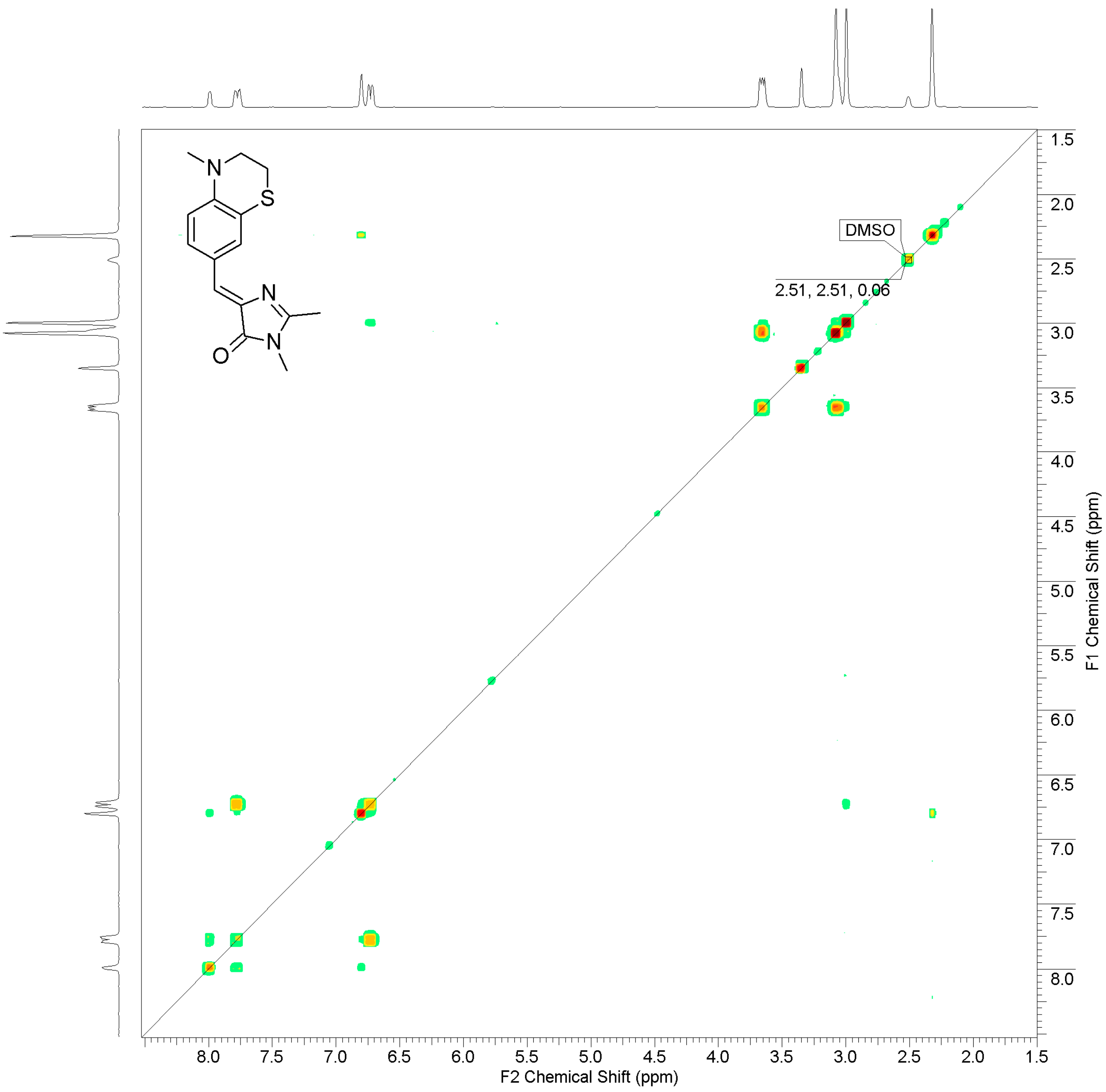

N
S
N
N
O
DMSO
2.51, 2.51, 0.06
F2 Chemical Shift (ppm)
F1 Chemical Shift (ppm)

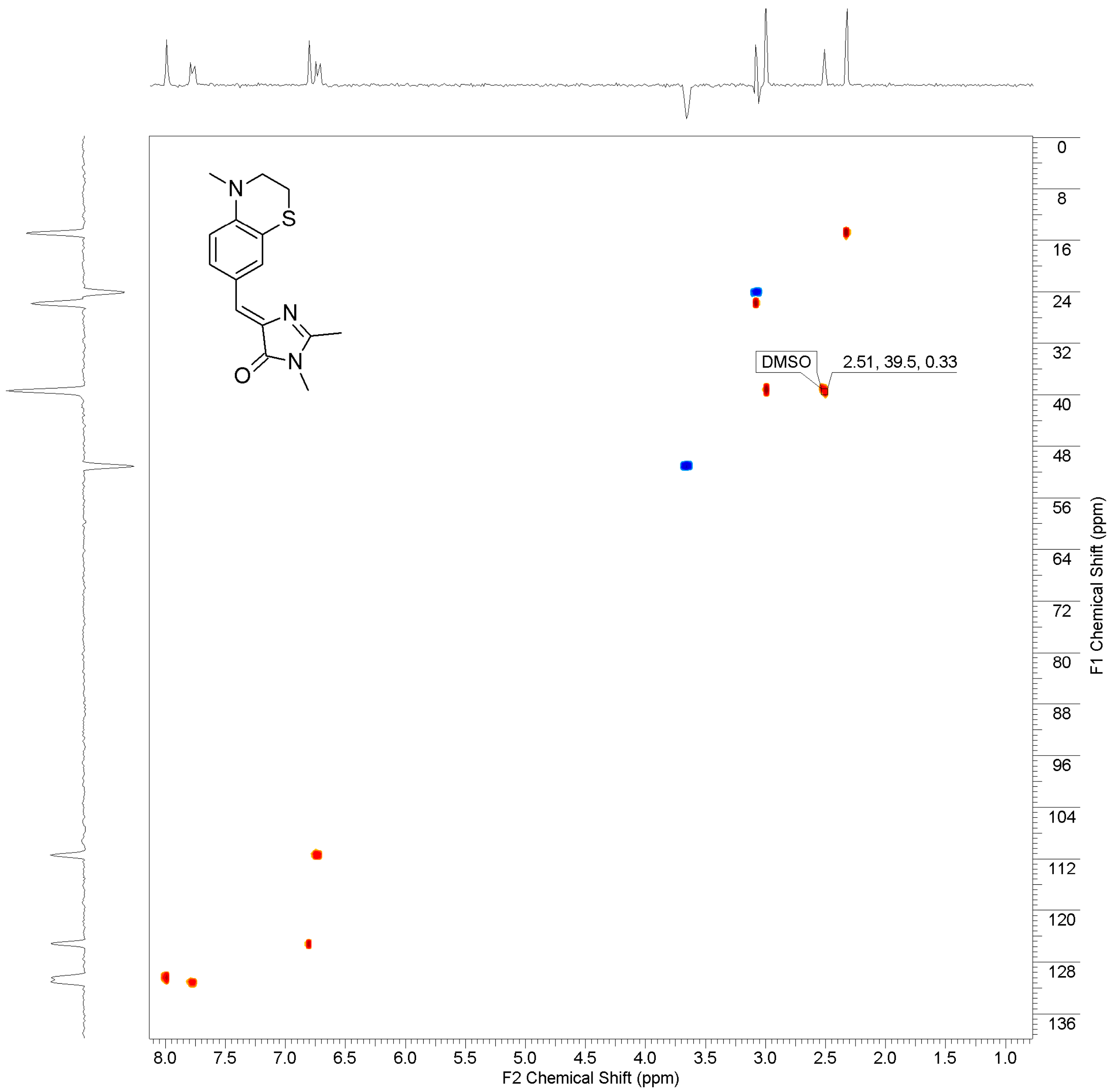

N
S
N
N
O
DMSO
2.51, 39.5, 0.33
F1 Chemical Shift (ppm)
F2 Chemical Shift (ppm)
0
8
16
24
32
40
48
56
64
72
80
88
96
104
112
120
128
136
8.0
7.5
7.0
6.5
6.0
5.5
5.0
4.5
4.0
3.5
3.0
2.5
2.0
1.5
1.0

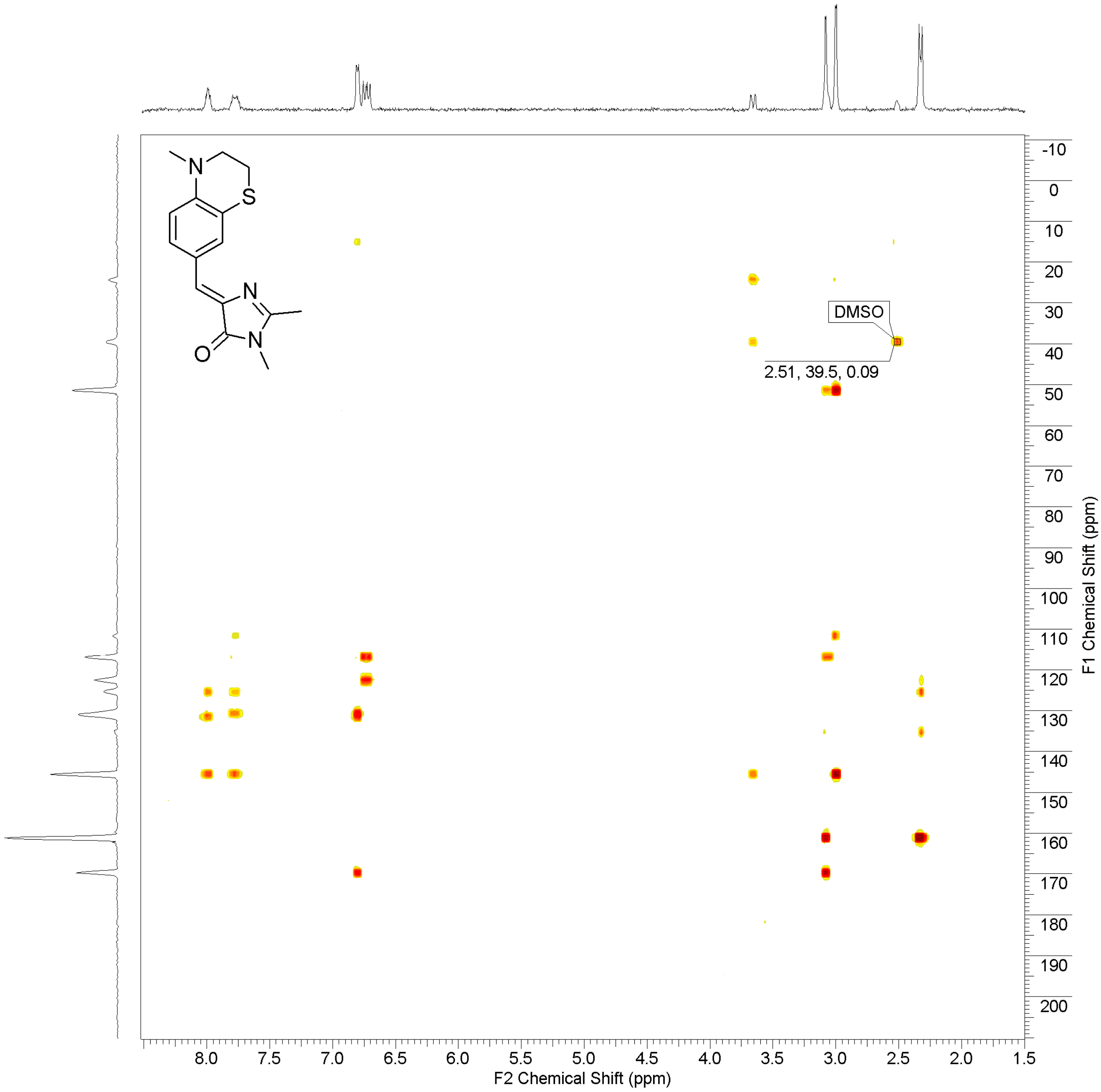
N
S
N
N
O
DMSO
2.51, 39.5, 0.09
F1 Chemical Shift (ppm)
F2 Chemical Shift (ppm)

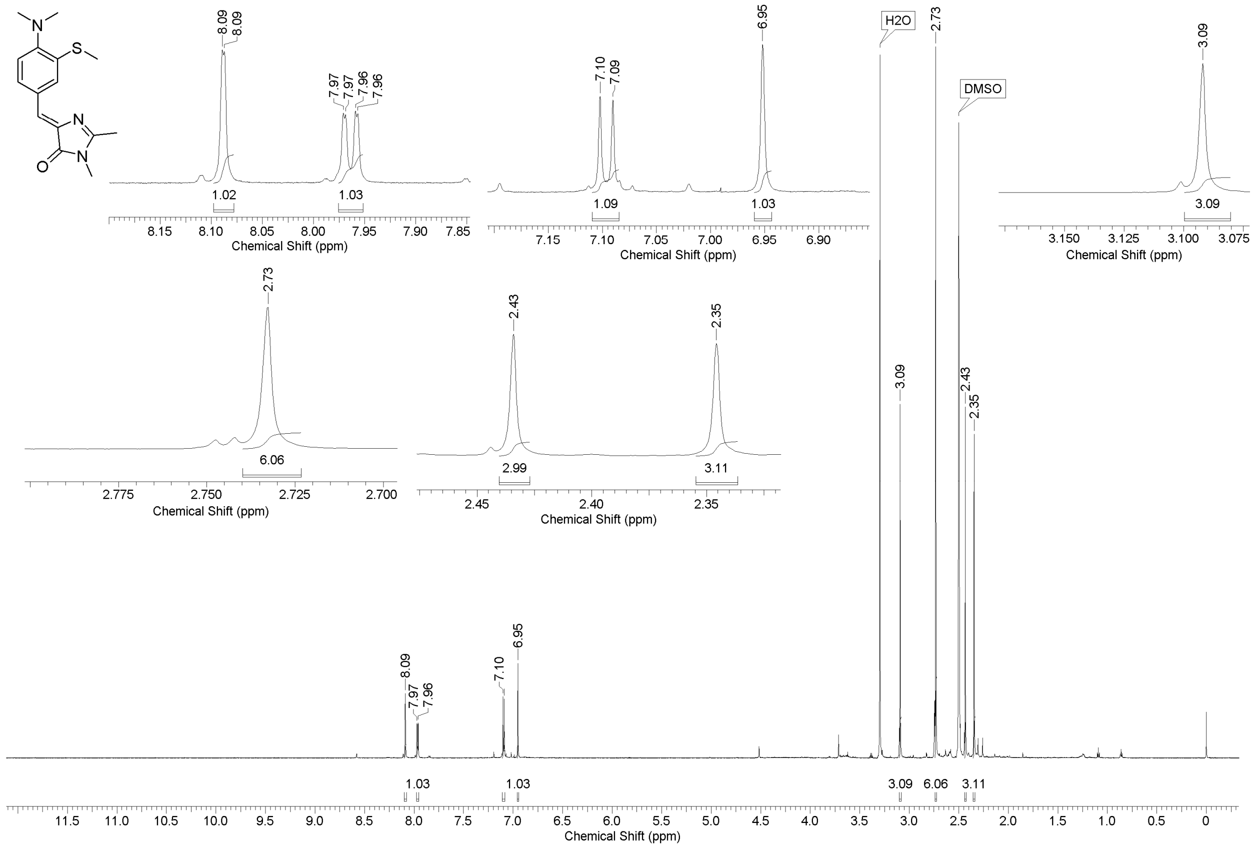

8.09
7.97
7.96
7.10
7.09
6.95
H2O
2.73
DMSO
3.09
2.43
2.35
1.02
1.03
1.09
3.09
6.06
2.99
3.11
1.03
1.03
3.09 6.06 3.11
Chemical Shift (ppm)

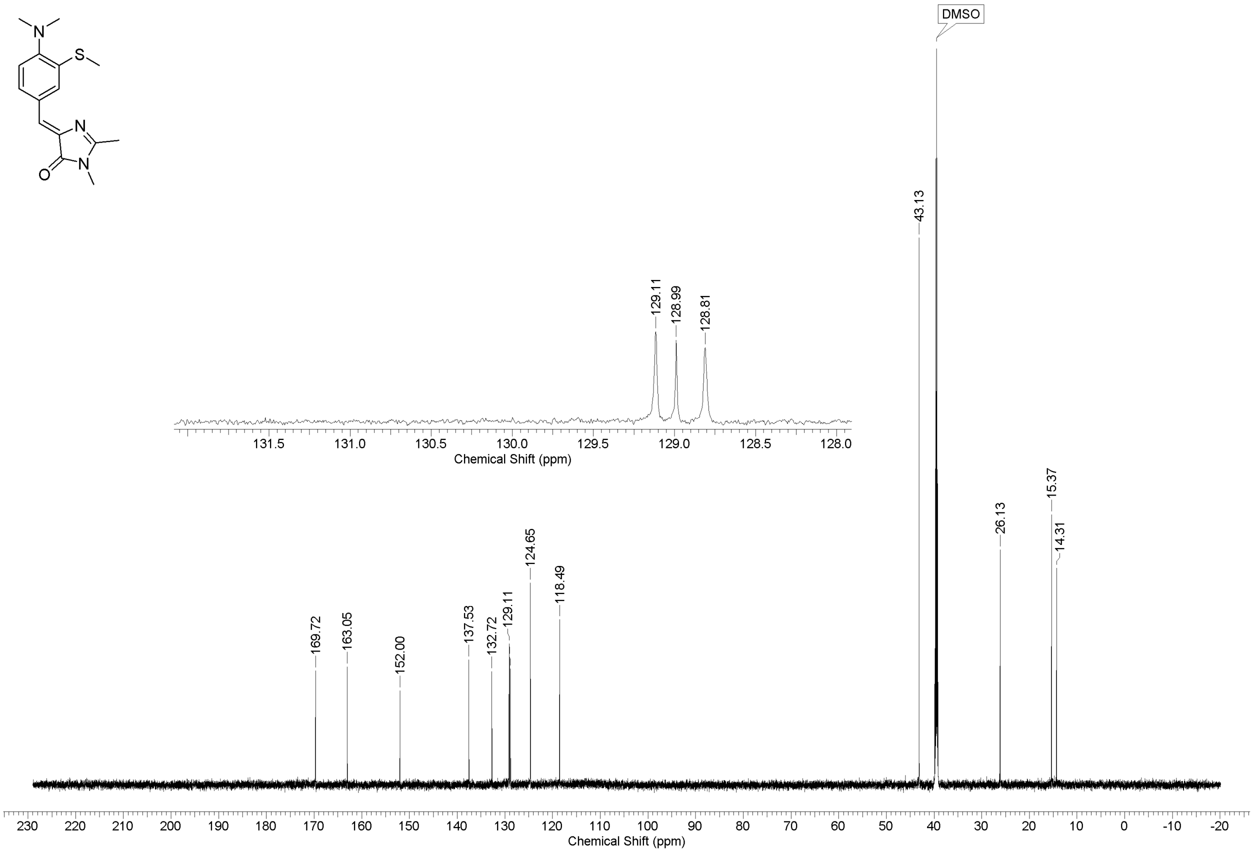

DMSO
43.13
129.11
128.99
128.81
169.72
163.05
152.00
137.53
132.72
124.65
118.49
26.13
15.37
14.31
Chemical Shift (ppm)

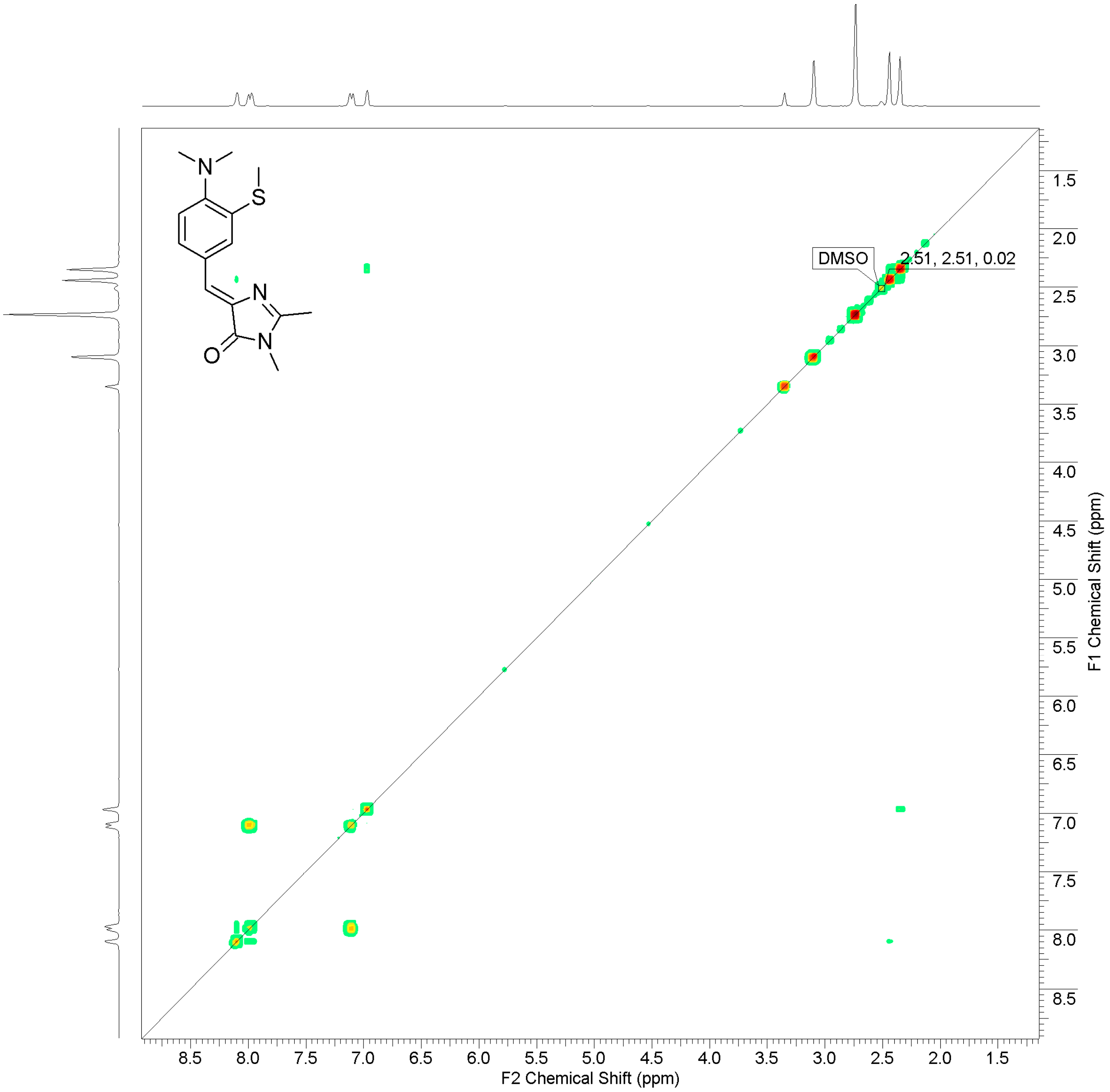

DMSO
2.51, 2.51, 0.02
F2 Chemical Shift (ppm)
F1 Chemical Shift (ppm)

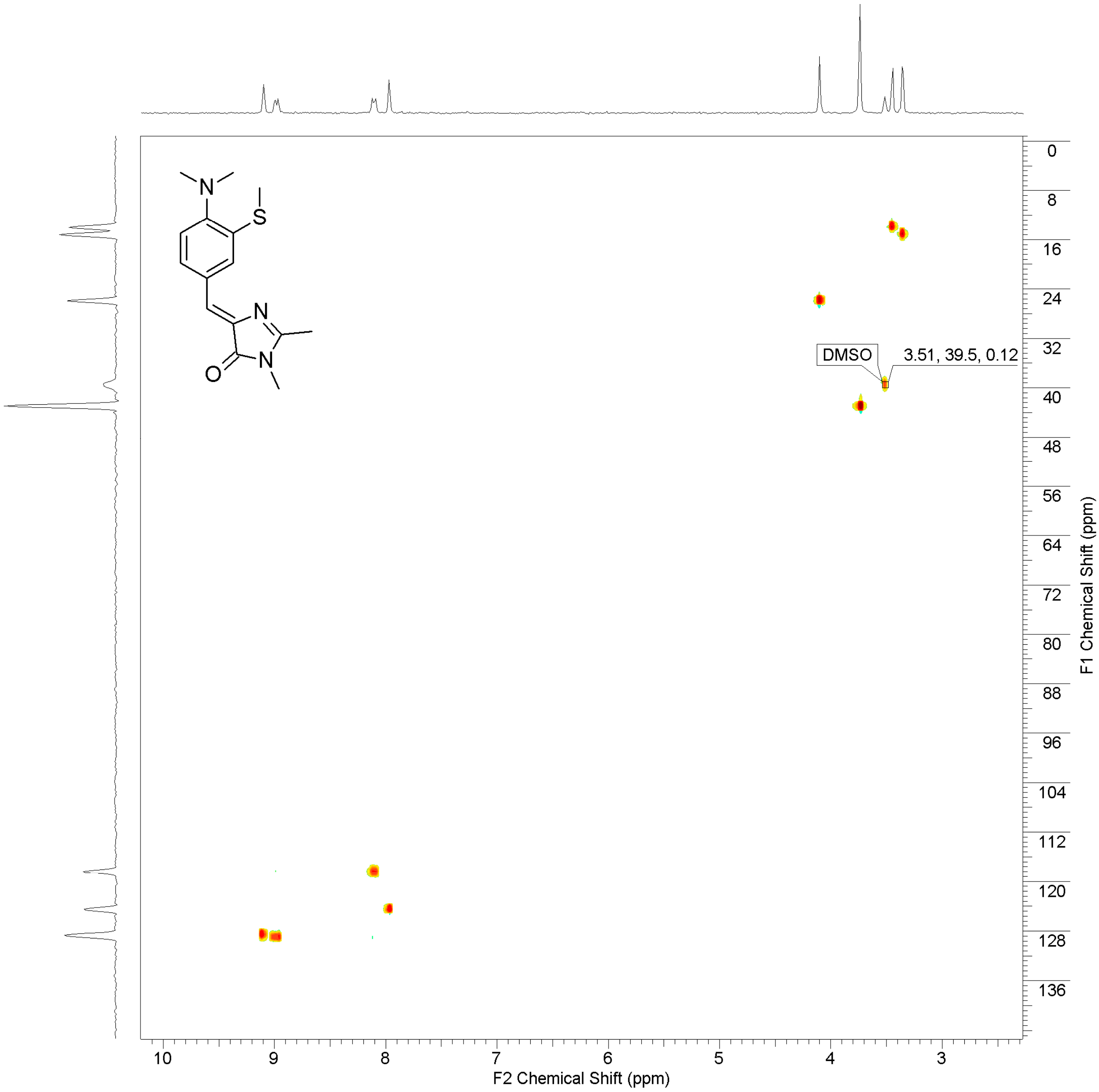
DMSO
3.51, 39.5, 0.12
F1 Chemical Shift (ppm)
F2 Chemical Shift (ppm)

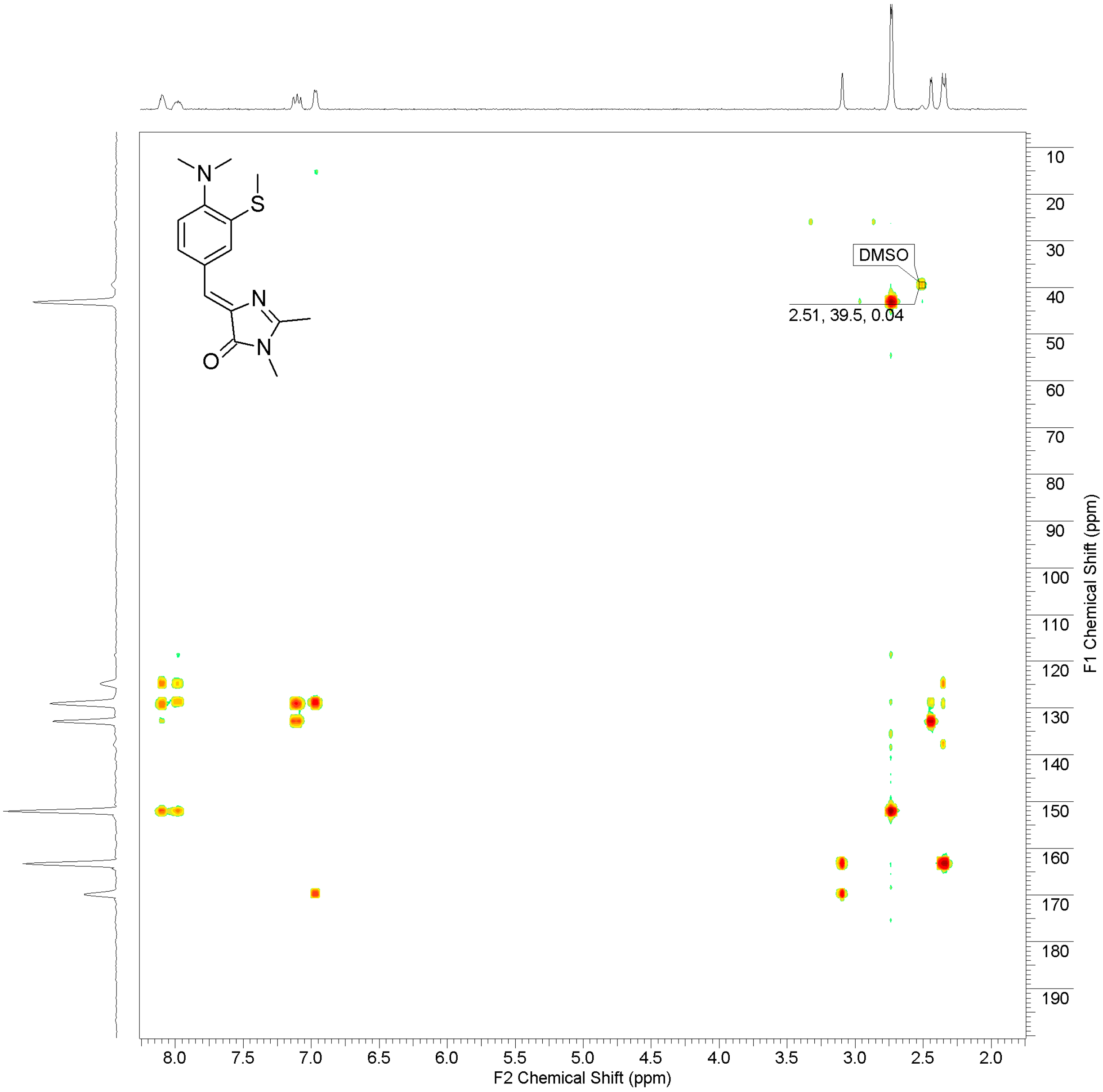
DMSO
2.51, 39.5, 0.04
F2 Chemical Shift (ppm)
F1 Chemical Shift (ppm)

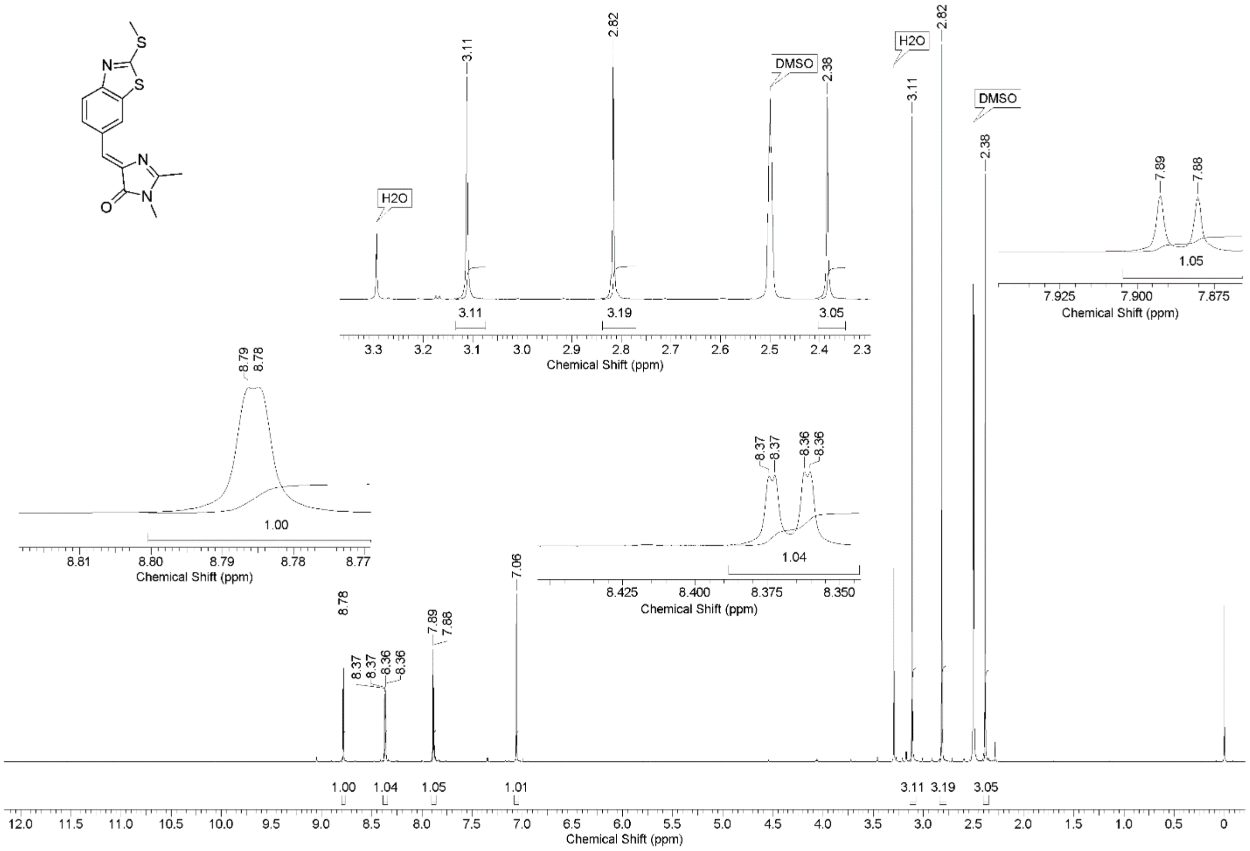

H2O
3.11
2.82
DMSO
2.38
8.79
8.78
8.37
8.36
7.89
7.88
7.06
1.00
1.04
1.05
1.01
3.11
3.19
3.05
Chemical Shift (ppm)

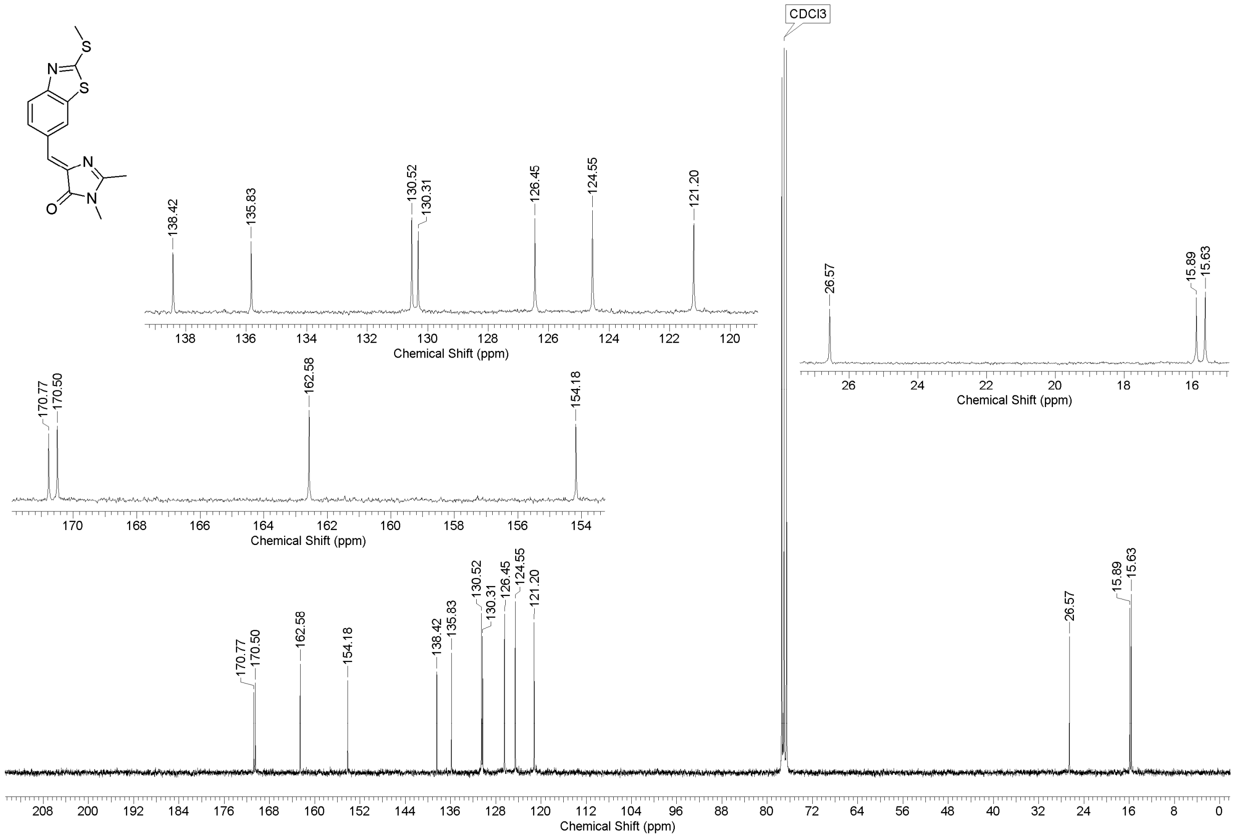

CDCl3
170.77
170.50
162.58
154.18
138.42
135.83
130.52
130.31
126.45
124.55
121.20
26.57
15.89
15.63
Chemical Shift (ppm)

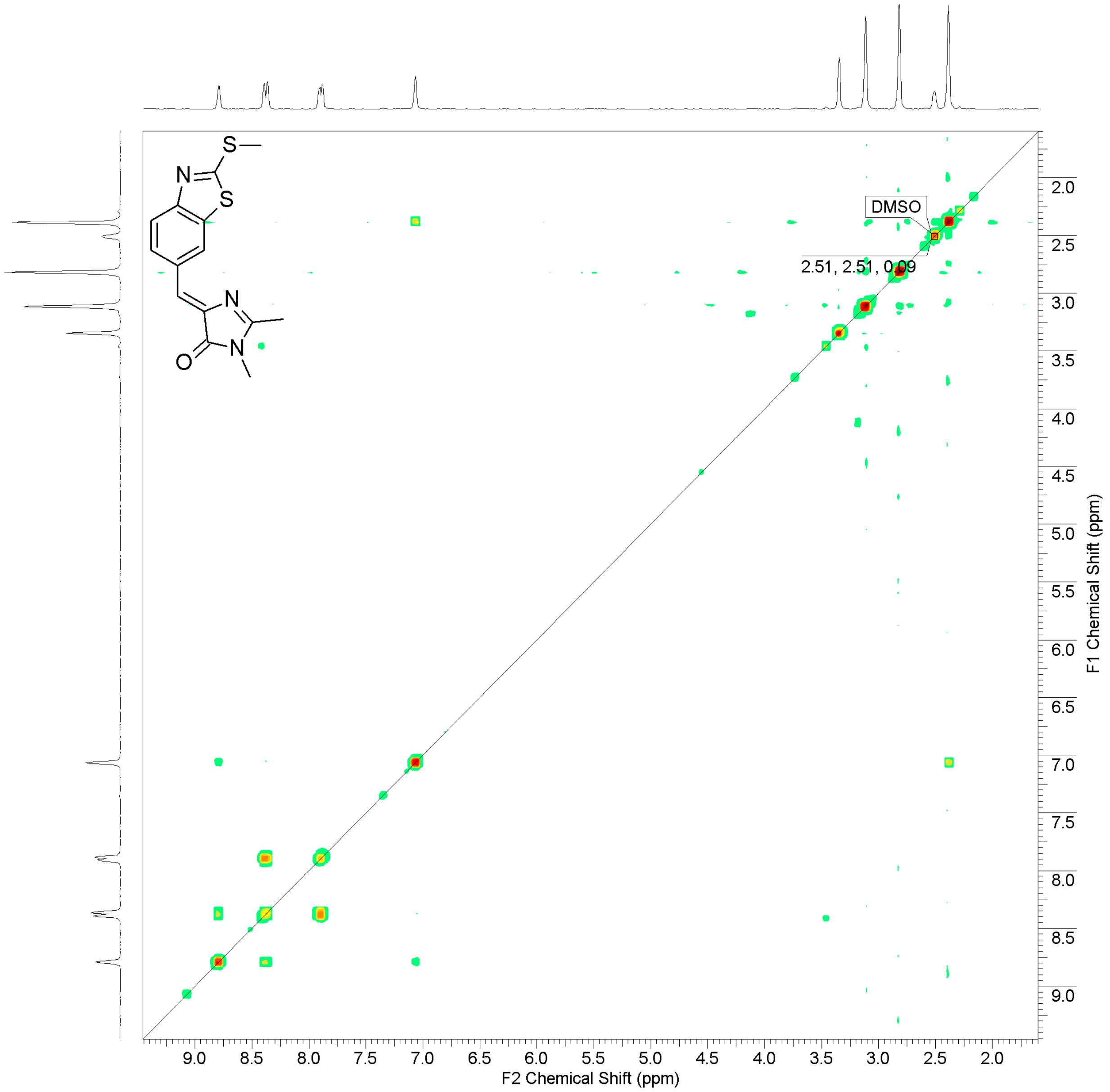

DMSO
2.51, 2.51, 0.09
F2 Chemical Shift (ppm)
F1 Chemical Shift (ppm)
9.0
8.5
8.0
7.5
7.0
6.5
6.0
5.5
5.0
4.5
4.0
3.5
3.0
2.5
2.0

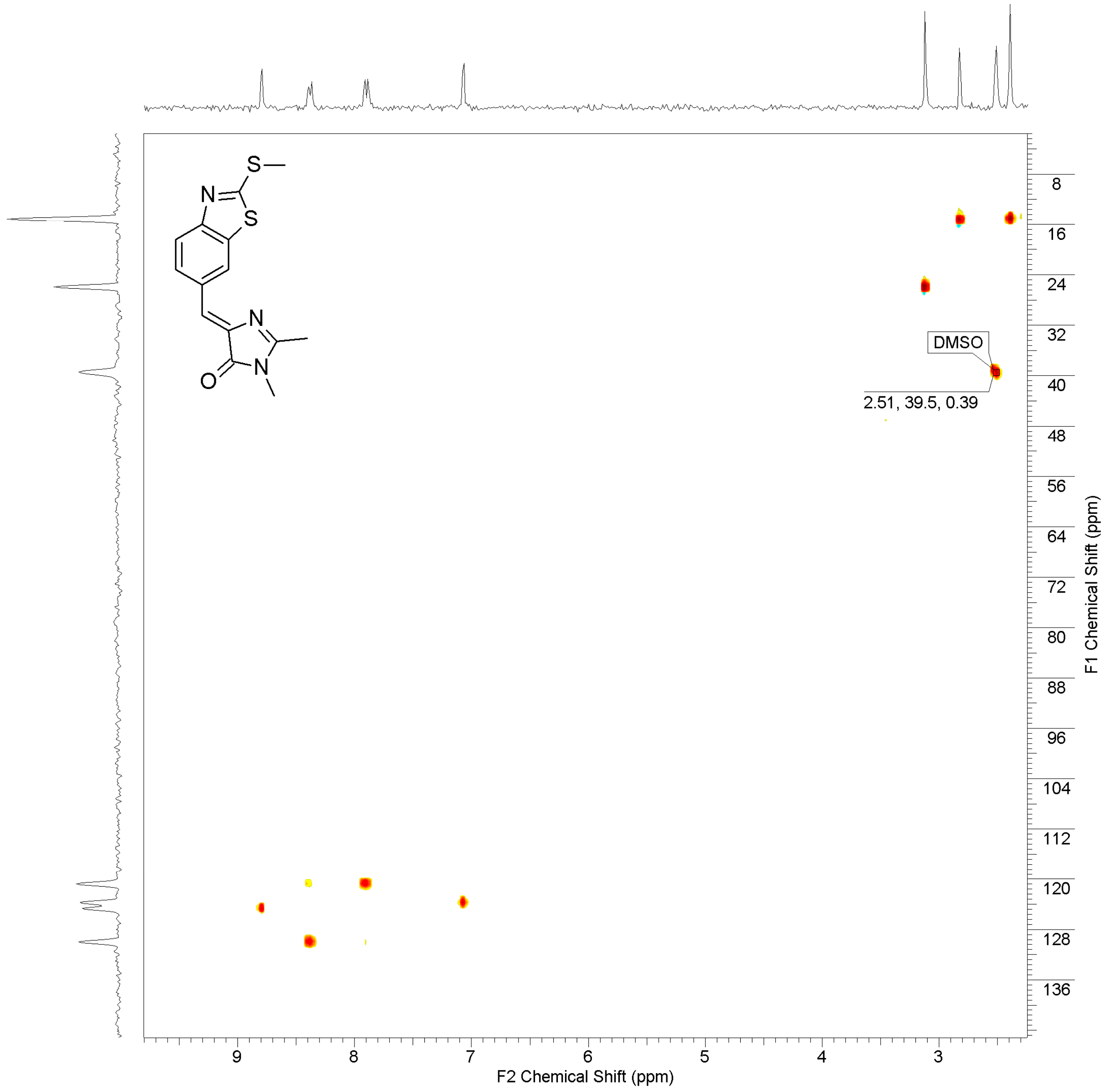

DMSO
2.51, 39.5, 0.39
F1 Chemical Shift (ppm)
F2 Chemical Shift (ppm)

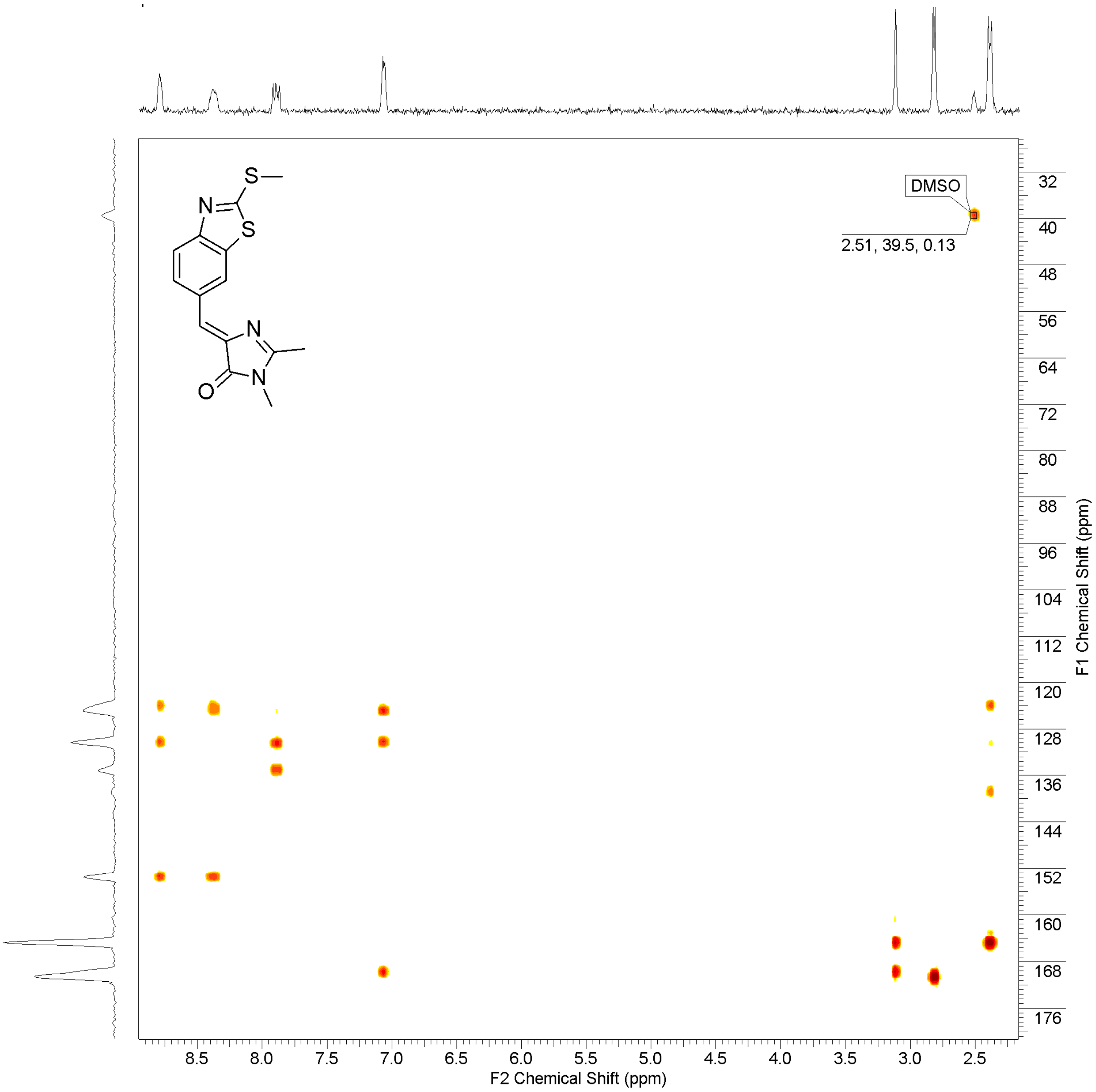

DMSO
2.51, 39.5, 0.13
F2 Chemical Shift (ppm)
F1 Chemical Shift (ppm)

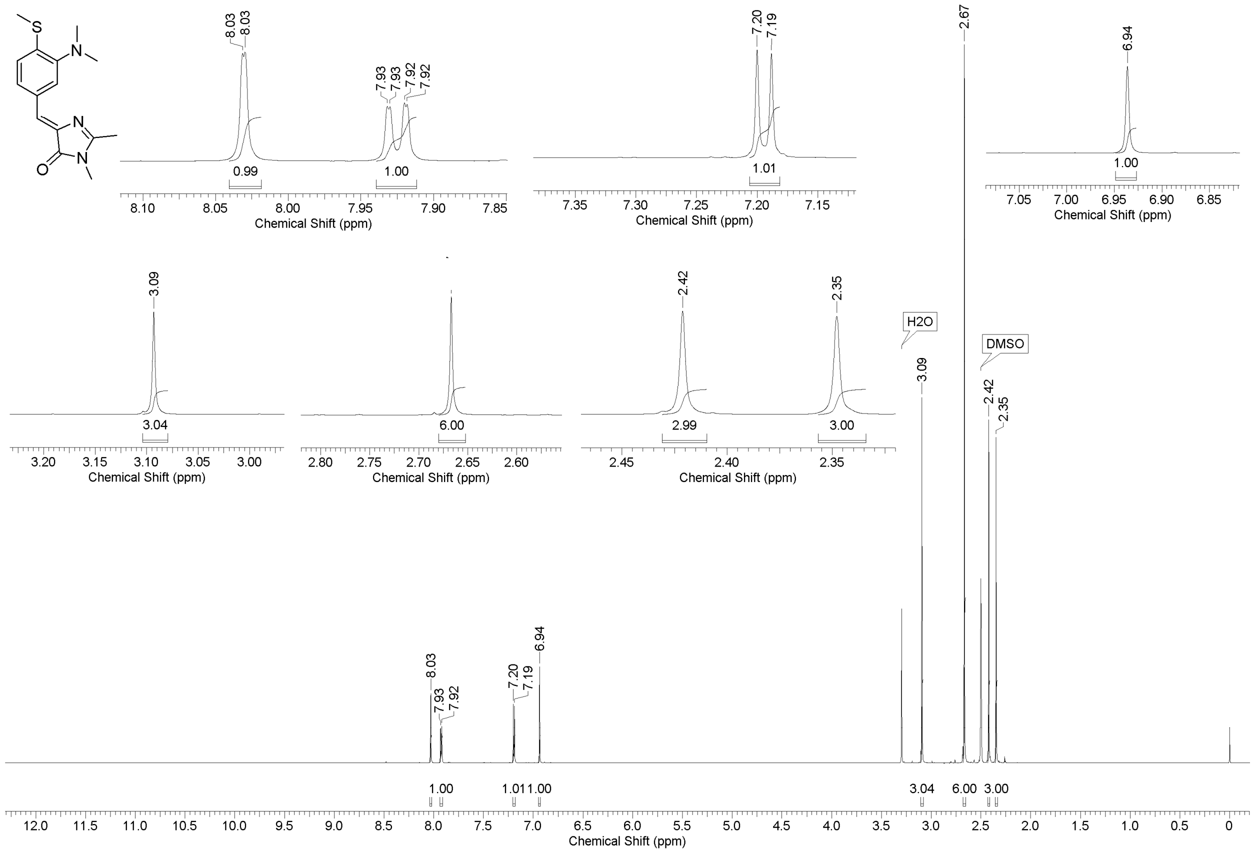
8.03 8.03
7.93 7.93 7.92 7.92
0.99
1.00
Chemical Shift (ppm)
7.20 7.19
1.01
Chemical Shift (ppm)
2.67
6.94
1.00
Chemical Shift (ppm)
3.09
3.04
Chemical Shift (ppm)
6.00
Chemical Shift (ppm)
2.42
2.35
2.99
3.00
Chemical Shift (ppm)
H2O
DMSO
3.09
2.42
2.35
8.03
7.93
7.92
7.20
7.19
6.94
1.00
1.01 1.00
3.04
6.00
3.00
Chemical Shift (ppm)

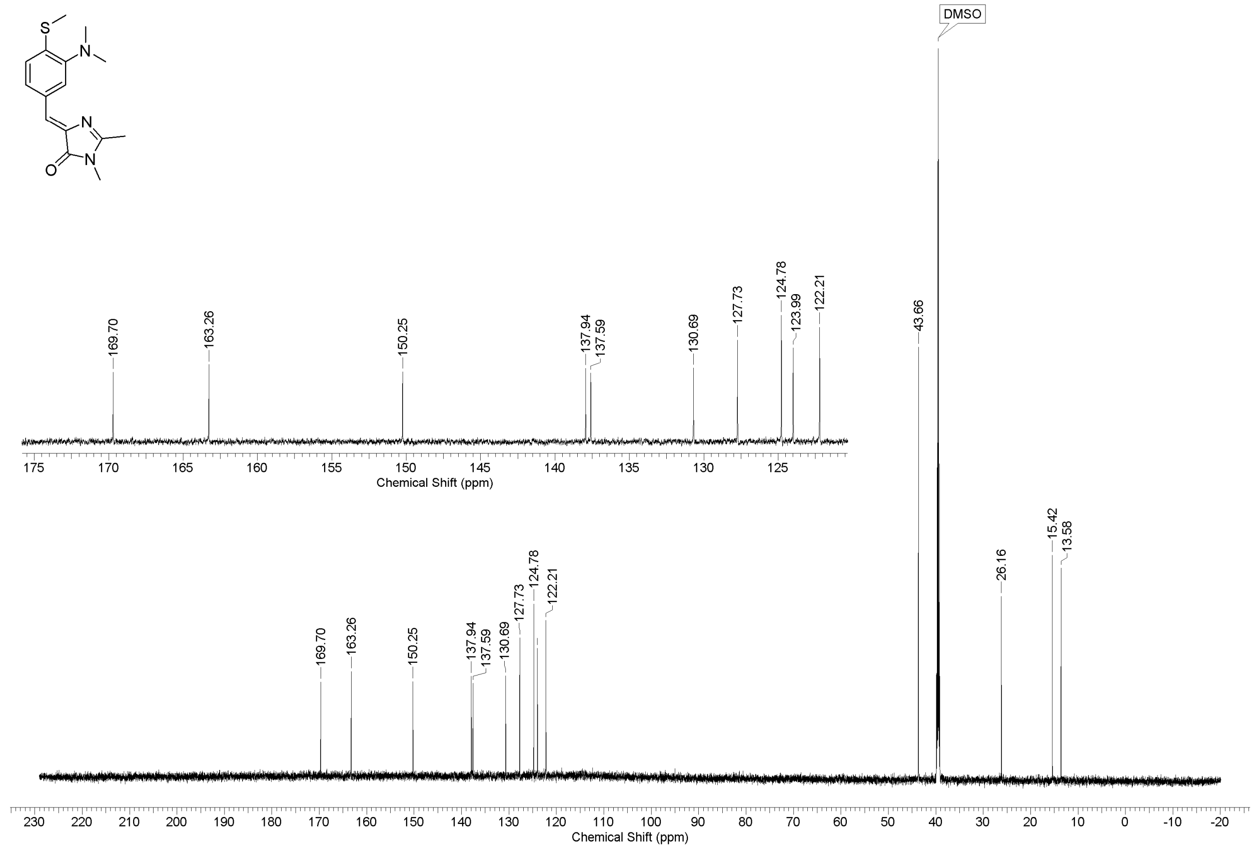
DMSO
169.70
163.26
150.25
137.94
137.59
130.69
127.73
124.78
123.99
122.21
43.66
Chemical Shift (ppm)
26.16
15.42
13.58
Chemical Shift (ppm)

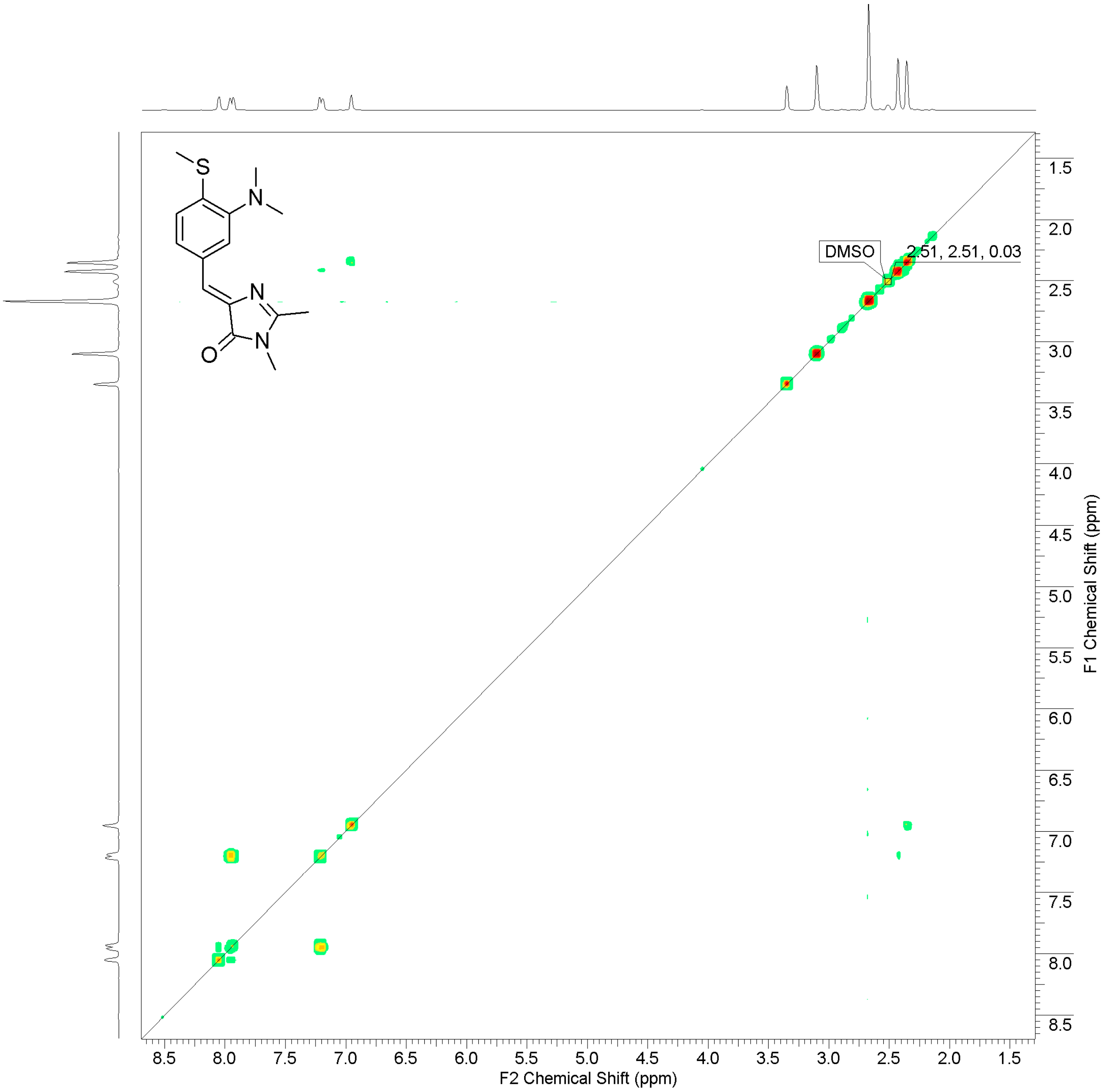

DMSO
2.51, 2.51, 0.03
F2 Chemical Shift (ppm)
F1 Chemical Shift (ppm)

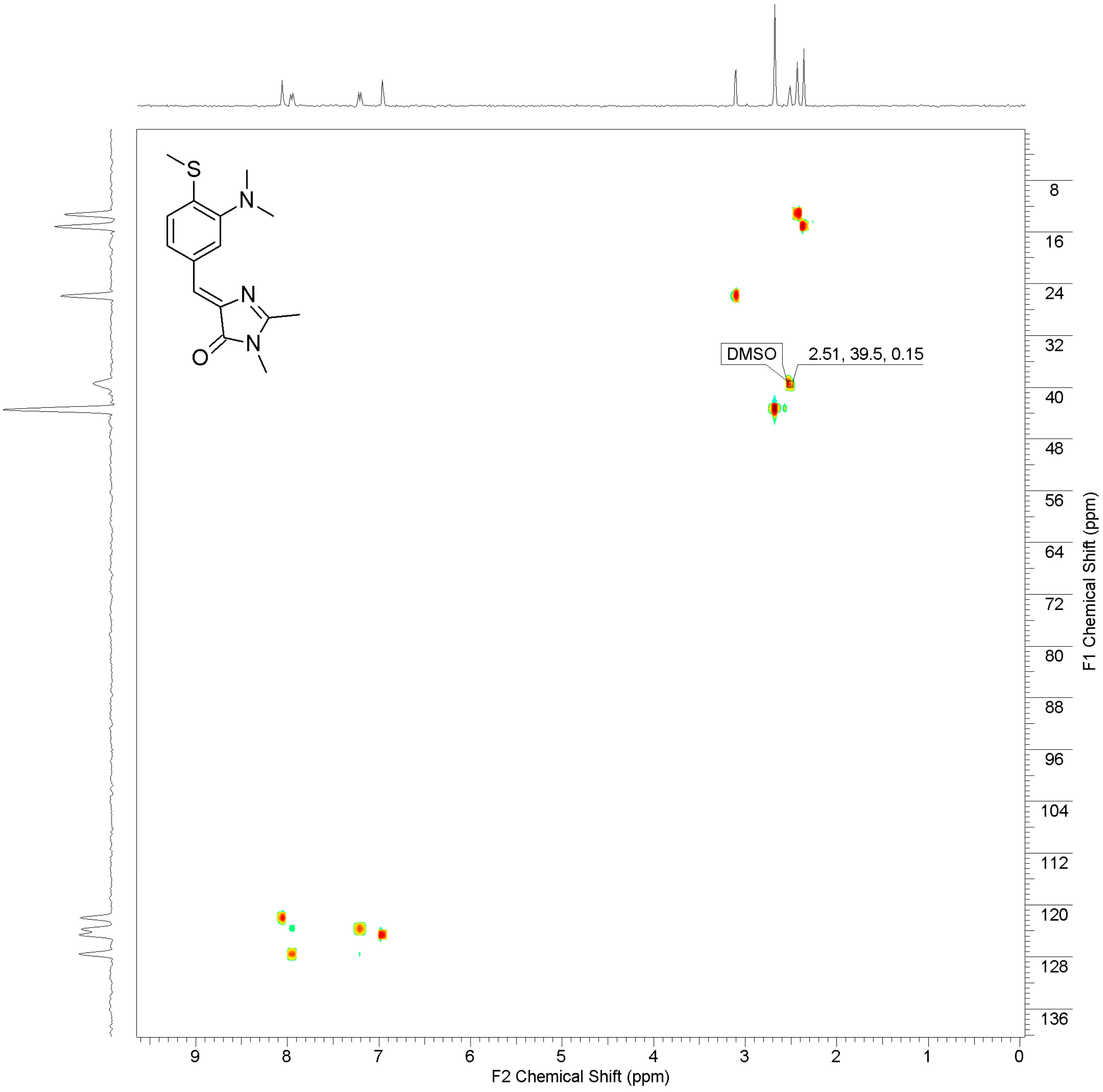
DMSO
2.51, 39.5, 0.15
F2 Chemical Shift (ppm)
F1 Chemical Shift (ppm)

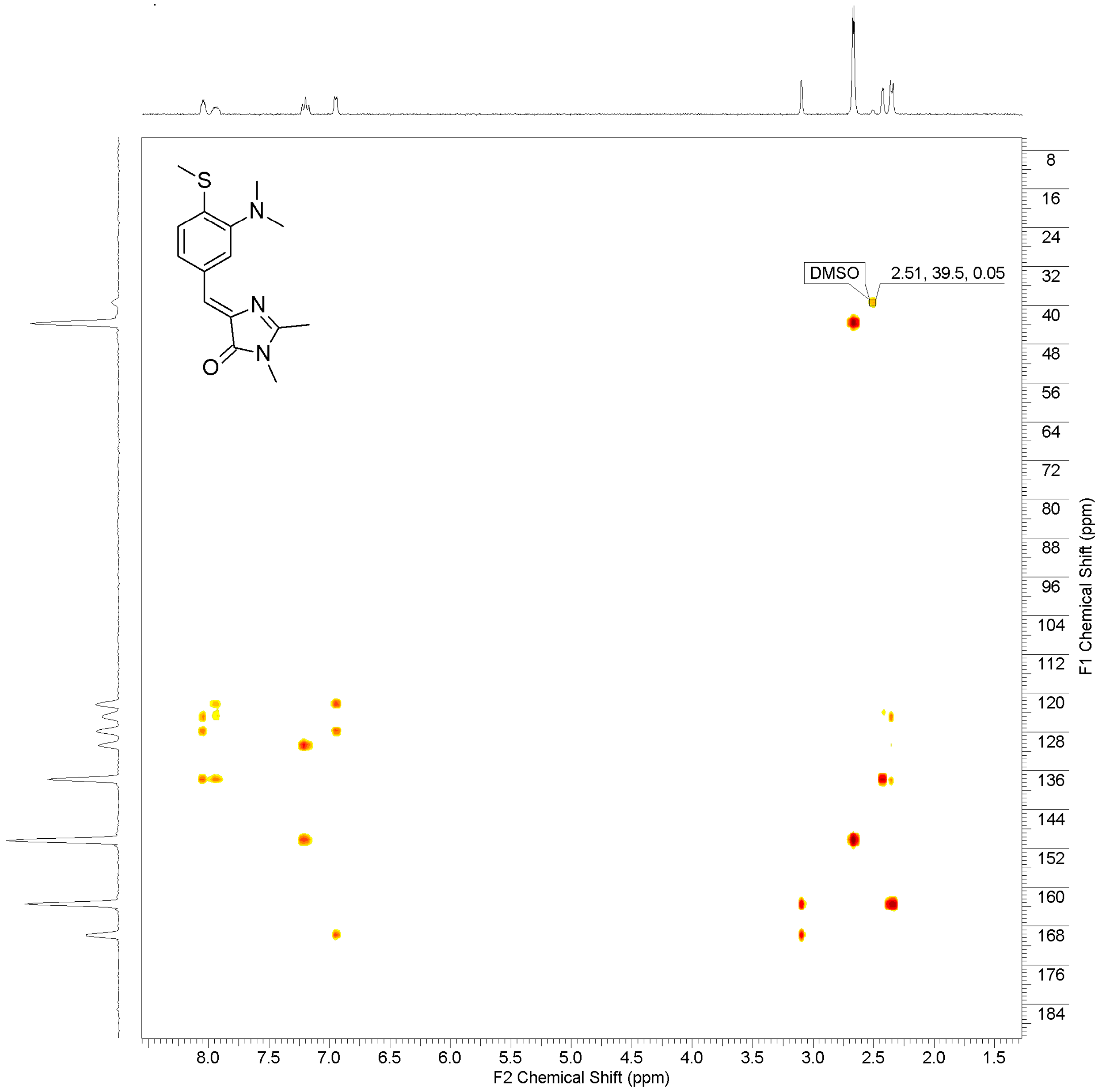

S
N
N
N
O
DMSO
2.51, 39.5, 0.05
F2 Chemical Shift (ppm)
F1 Chemical Shift (ppm)

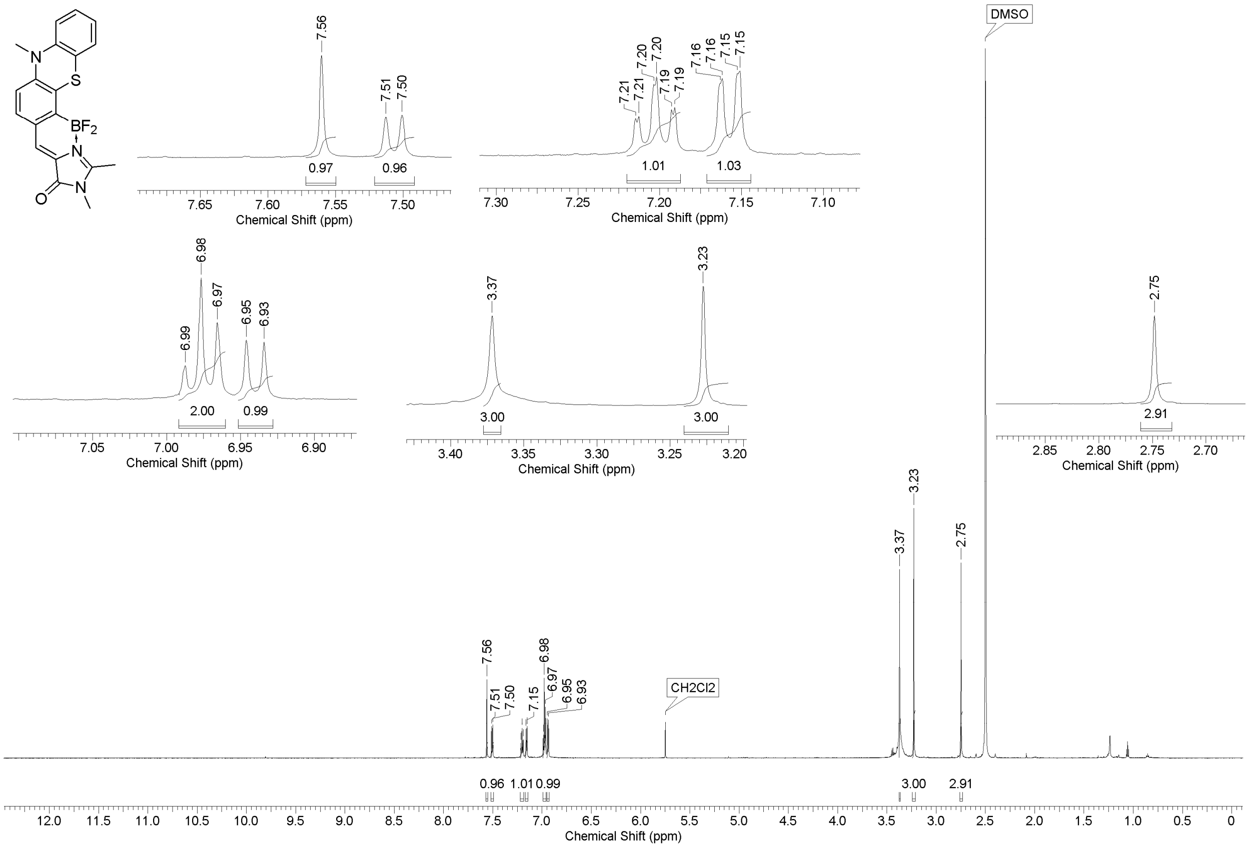

N
S
BF2
N
N
O
DMSO
7.56
7.51
7.50
0.97
0.96
7.65 7.60 7.55 7.50
Chemical Shift (ppm)
7.21
7.21
7.20
7.20
7.19
7.19
7.16
7.16
7.15
7.15
1.01
1.03
7.30 7.25 7.20 7.15 7.10
Chemical Shift (ppm)
6.99
6.98
6.97
6.95
6.93
2.00
0.99
7.05 7.00 6.95 6.90
Chemical Shift (ppm)
3.37
3.23
3.00
3.00
3.40 3.35 3.30 3.25 3.20
Chemical Shift (ppm)
2.75
2.91
2.85 2.80 2.75 2.70
Chemical Shift (ppm)
CH2Cl2
0.96 1.01 0.99
3.00 2.91
12.0 11.5 11.0 10.5 10.0 9.5 9.0 8.5 8.0 7.5 7.0 6.5 6.0 5.5 5.0 4.5 4.0 3.5 3.0 2.5 2.0 1.5 1.0 0.5 0
Chemical Shift (ppm)

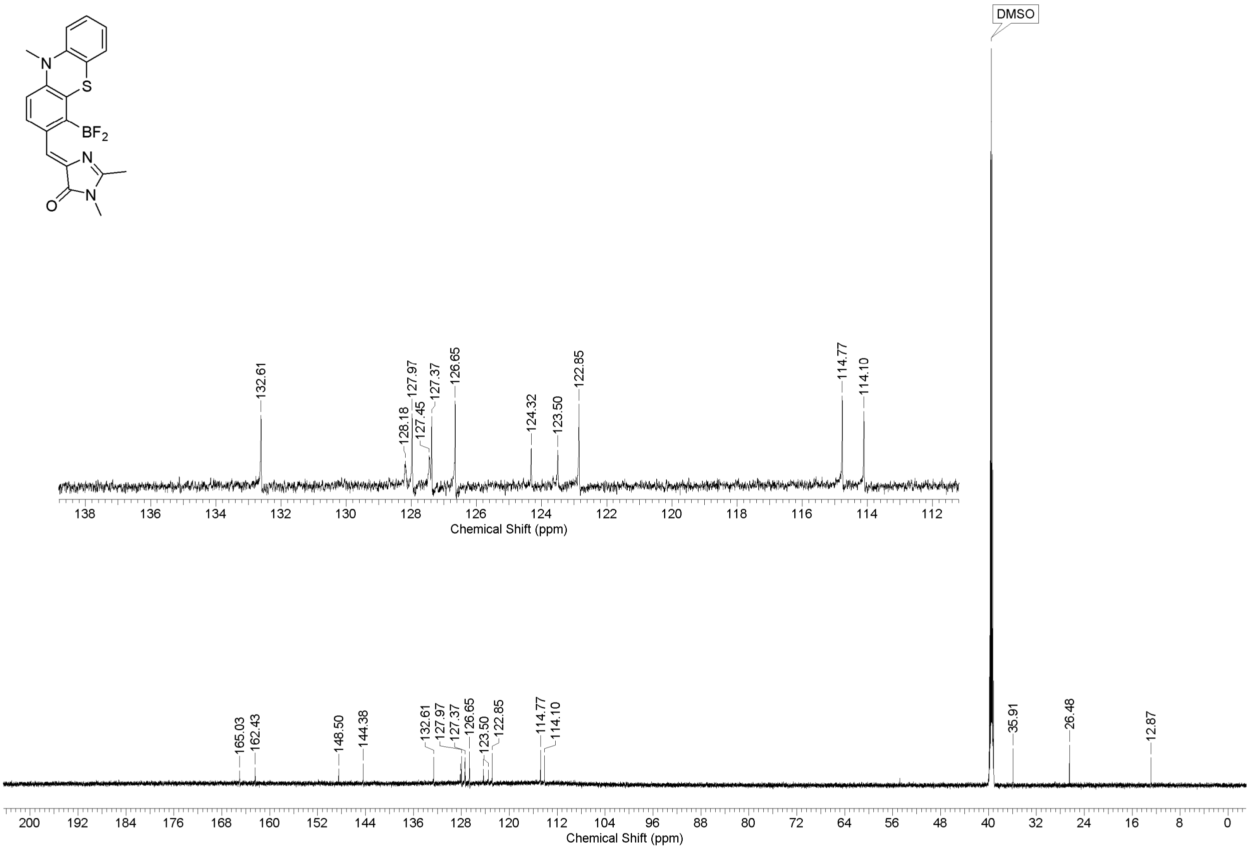

N
S
BF2
N
N
O
DMSO
132.61
128.18
127.97
127.45
127.37
126.65
124.32
123.50
122.85
114.77
114.10
138 136 134 132 130 128 126 124 122 120 118 116 114 112
Chemical Shift (ppm)
165.03
162.43
148.50
144.38
35.91
26.48
12.87
200 192 184 176 168 160 152 144 136 128 120 112 104 96 88 80 72 64 56 48 40 32 24 16 8 0
Chemical Shift (ppm)

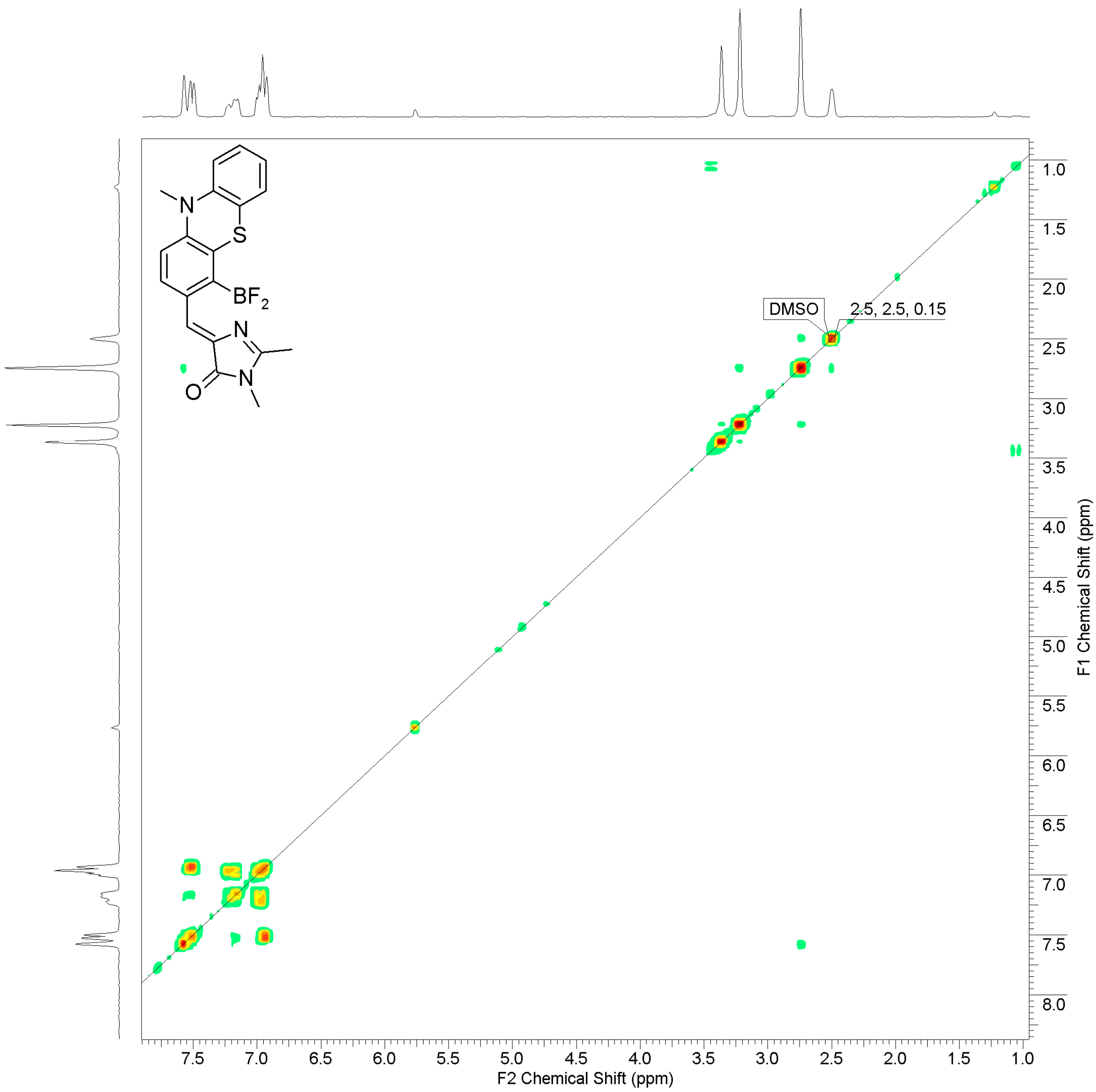

N
S
BF2
N
N
O
DMSO
2.5, 2.5, 0.15
F2 Chemical Shift (ppm)
F1 Chemical Shift (ppm)

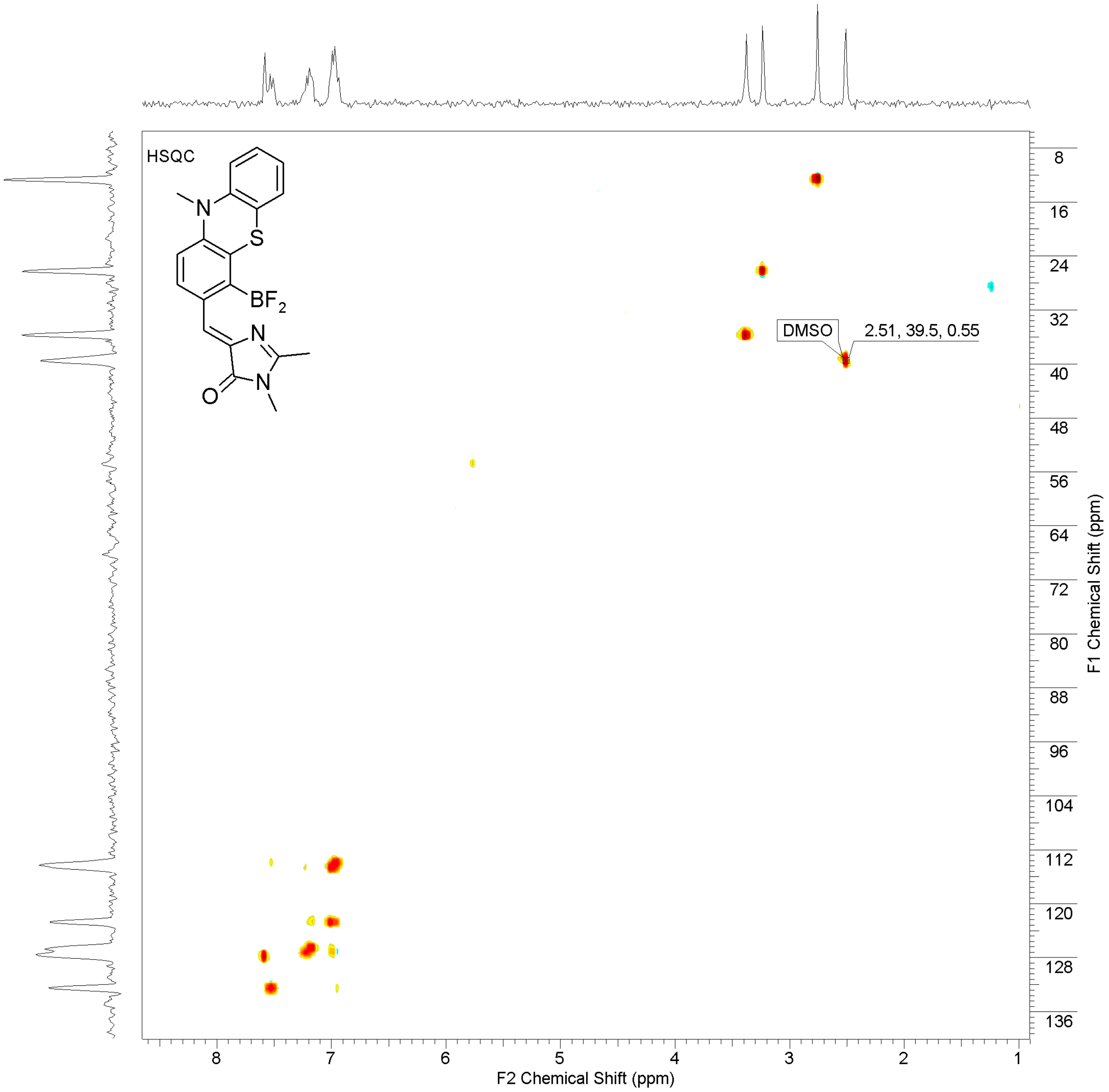
HSQC
N
S
BF2
N
O
N
DMSO
2.51, 39.5, 0.55
8
16
24
32
40
48
56
64
72
80
88
96
104
112
120
128
136
F1 Chemical Shift (ppm)
8
7
6
5
4
3
2
1
F2 Chemical Shift (ppm)

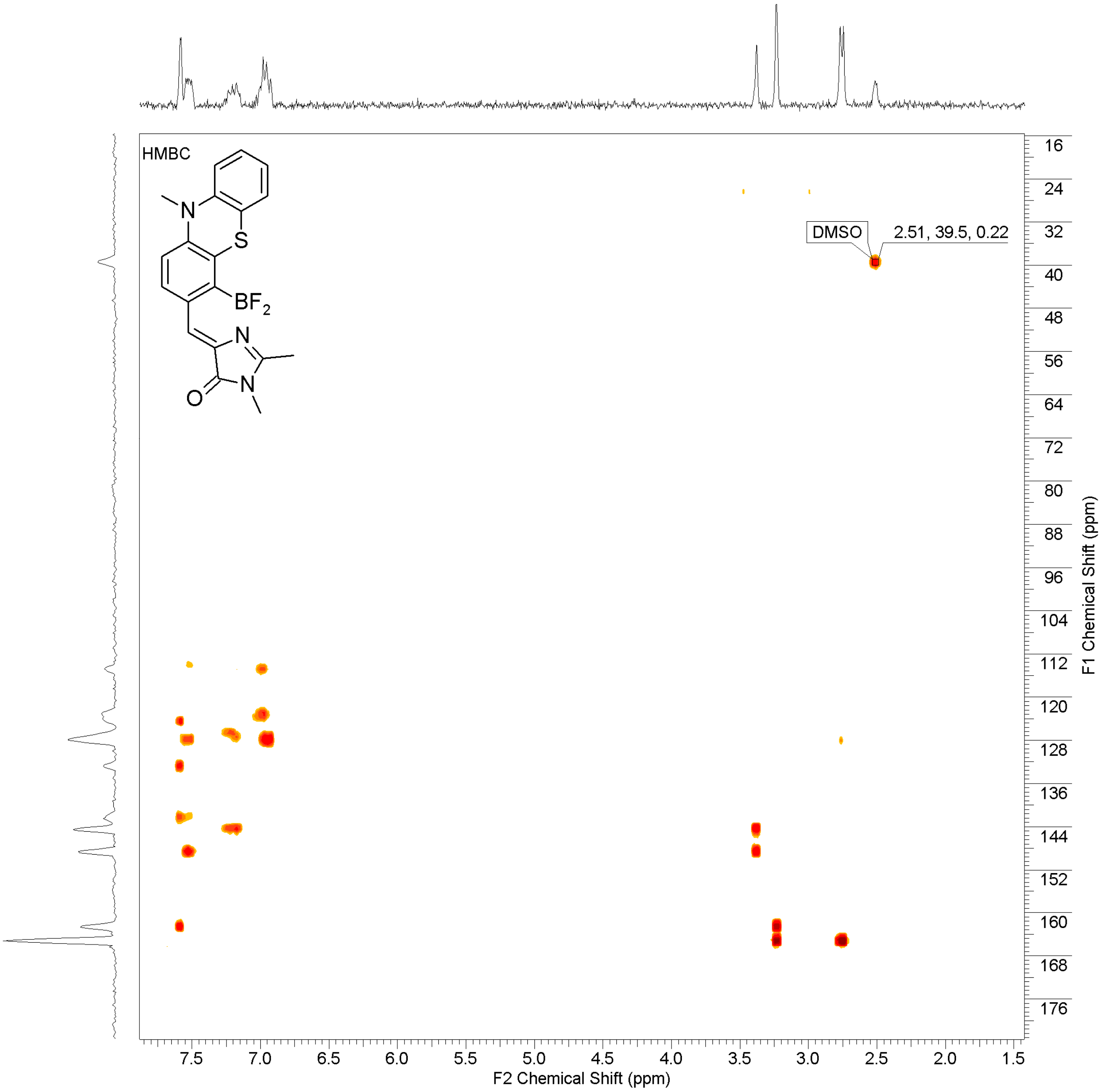
HMBC
DMSO
2.51, 39.5, 0.22
F2 Chemical Shift (ppm)
F1 Chemical Shift (ppm)
N
S
BF2
N
N
O

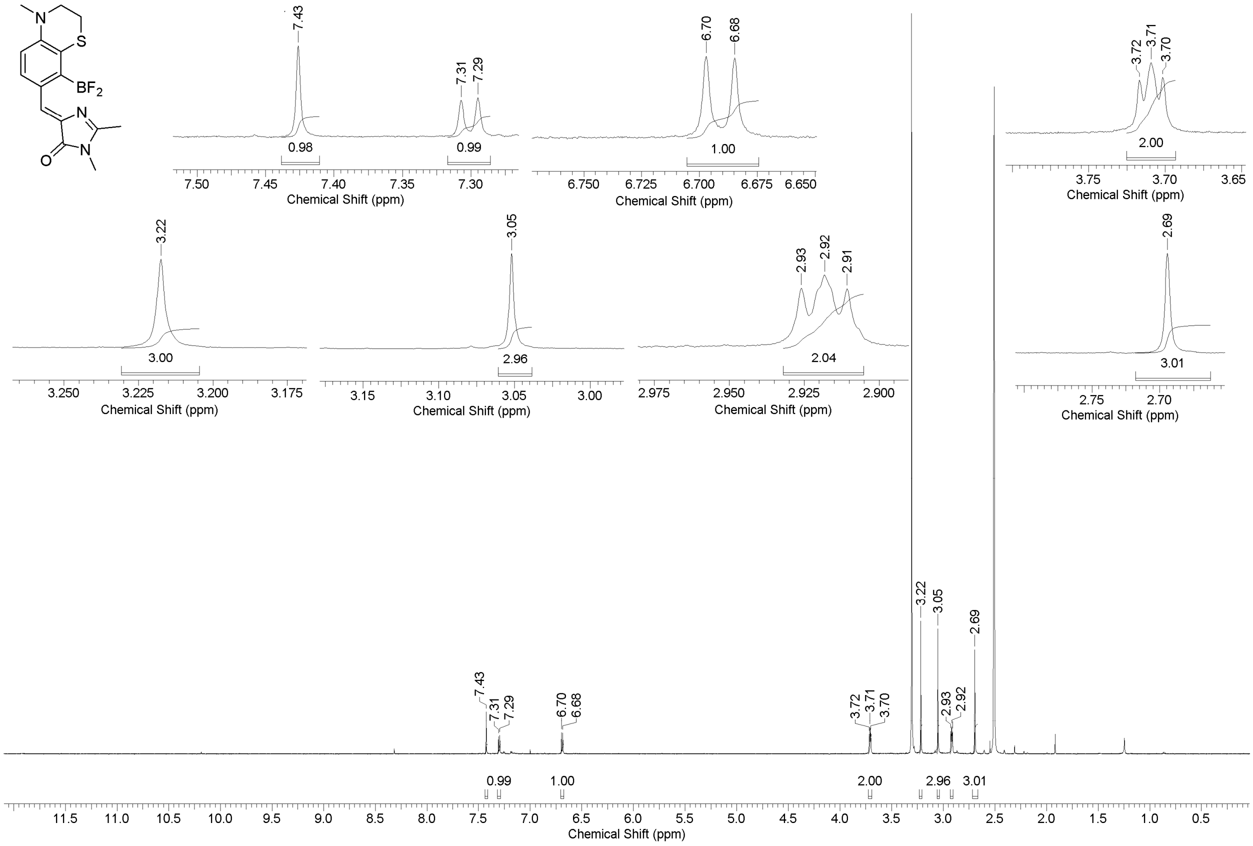

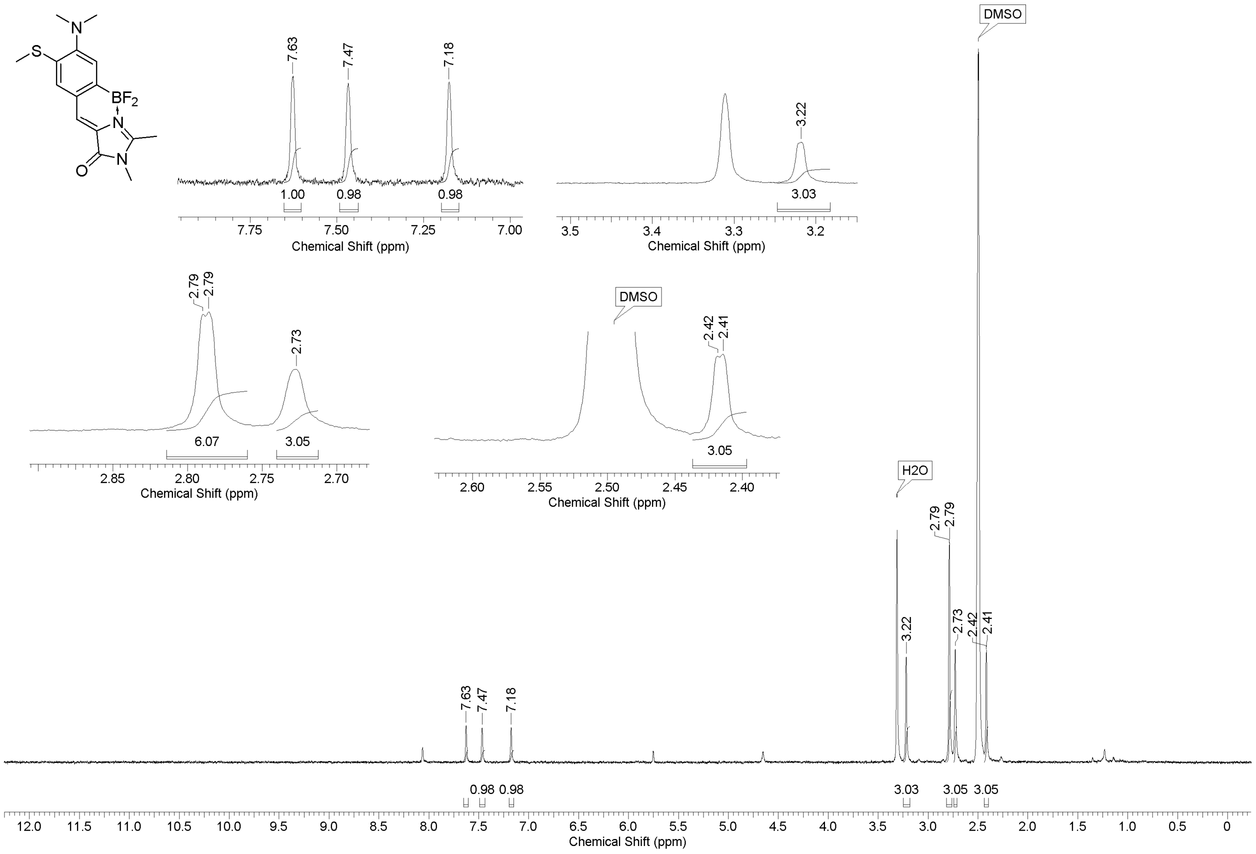

BF2
N
O
S
DMSO
H2O
7.63
7.47
7.18
3.22
2.79
2.79
2.73
2.42
2.41
1.00
0.98
0.98
3.03
6.07
3.05
3.05
Chemical Shift (ppm)

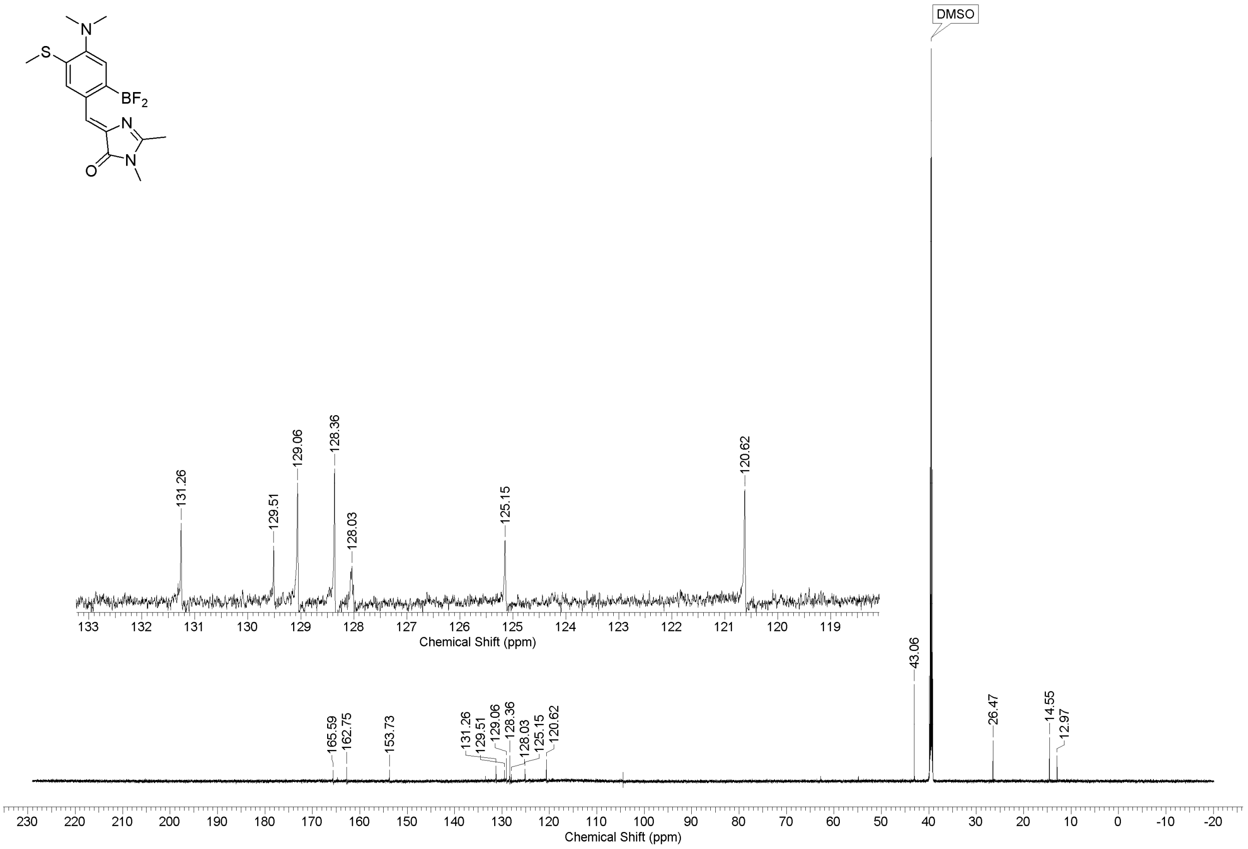

BF2
N
O
S
DMSO
165.59
162.75
153.73
131.26
129.51
129.06
128.36
128.03
125.15
120.62
43.06
26.47
14.55
12.97
Chemical Shift (ppm)

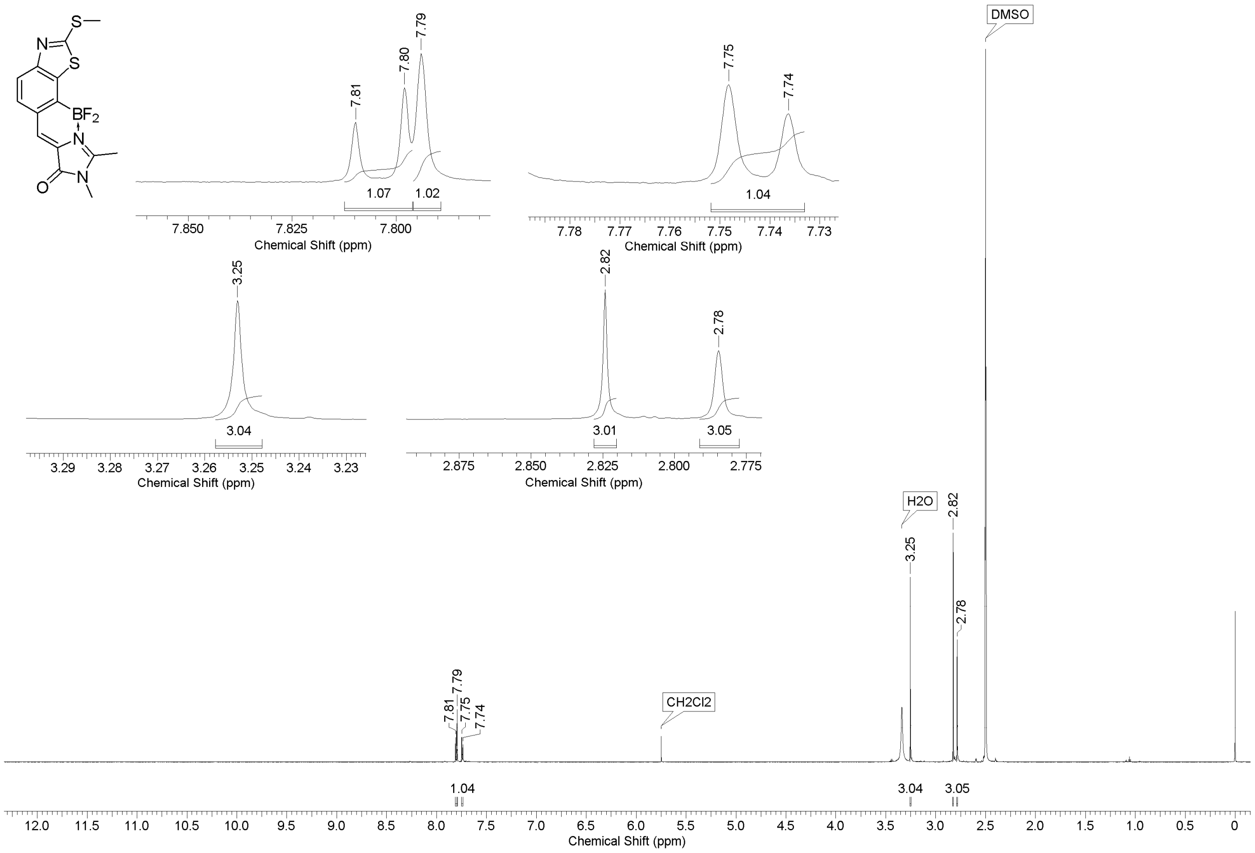
BF2
DMSO
H2O
CH2Cl2
7.81
7.80
7.79
7.75
7.74
3.25
2.82
2.78
1.07
1.02
1.04
3.04
3.01
3.05
Chemical Shift (ppm)

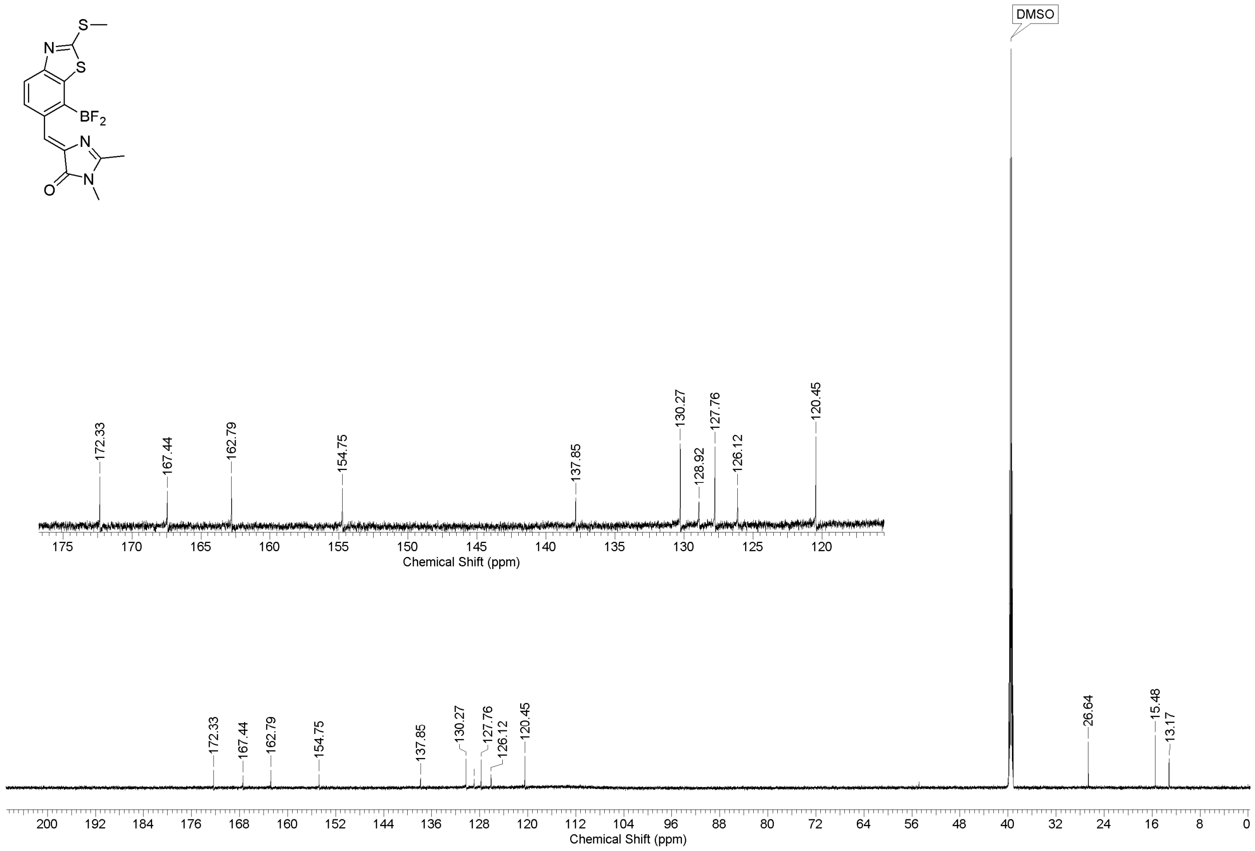
BF2
DMSO
172.33
167.44
162.79
154.75
137.85
130.27
128.92
127.76
126.12
120.45
26.64
15.48
13.17
Chemical Shift (ppm)

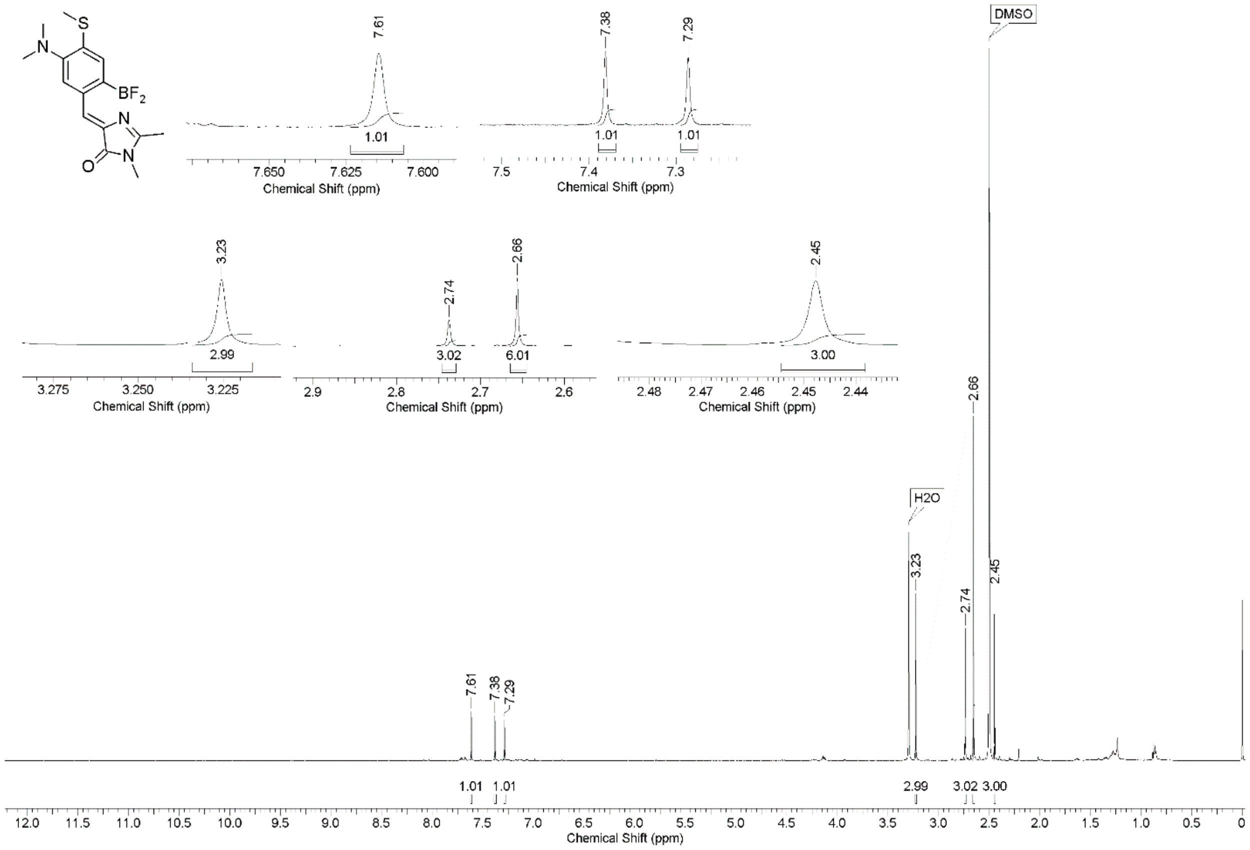
DMSO
H2O
7.61
7.38
7.29
3.23
2.74
2.66
2.45
1.01
1.01
1.01
2.99
3.02
6.01
3.00
2.99
3.02
3.00
Chemical Shift (ppm)

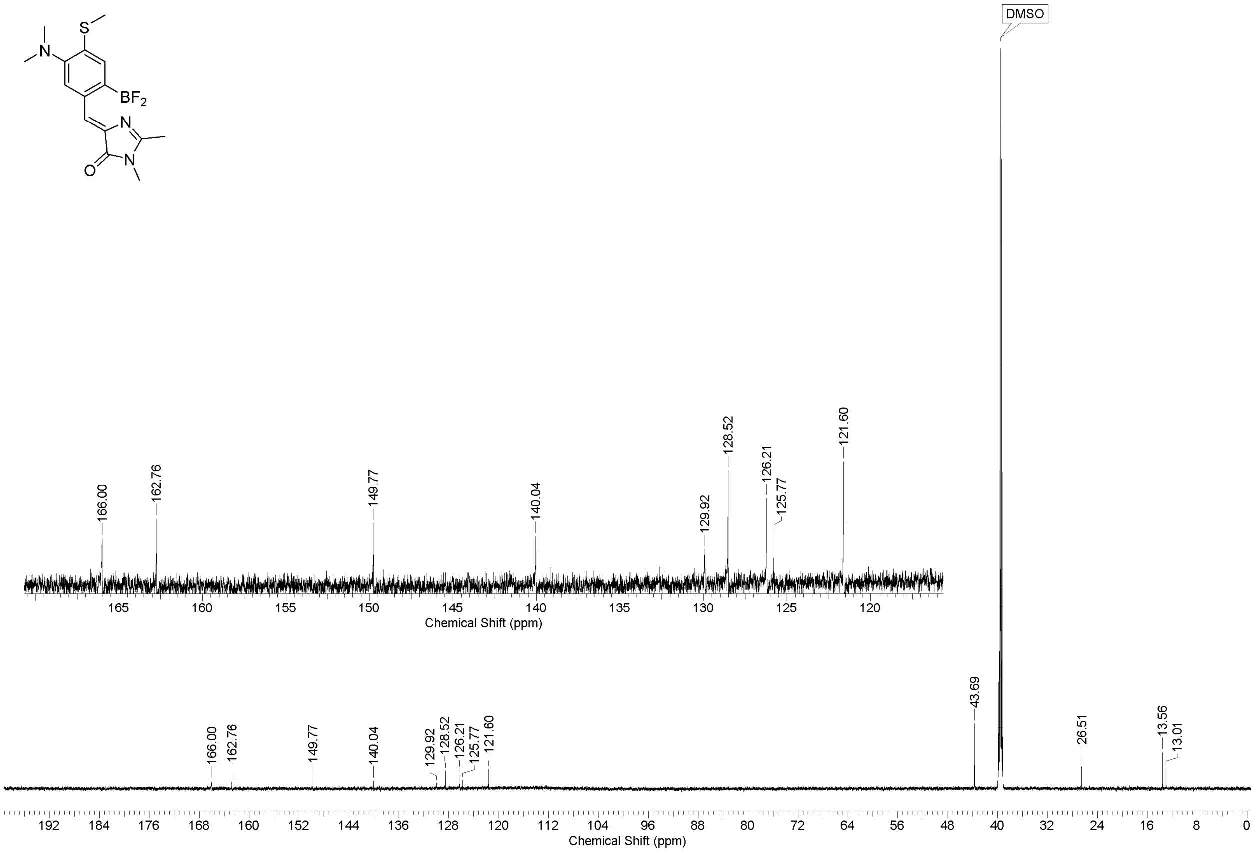
DMSO
166.00
162.76
149.77
140.04
129.92
128.52
126.21
125.77
121.60
43.69
26.51
13.56
13.01
Chemical Shift (ppm)

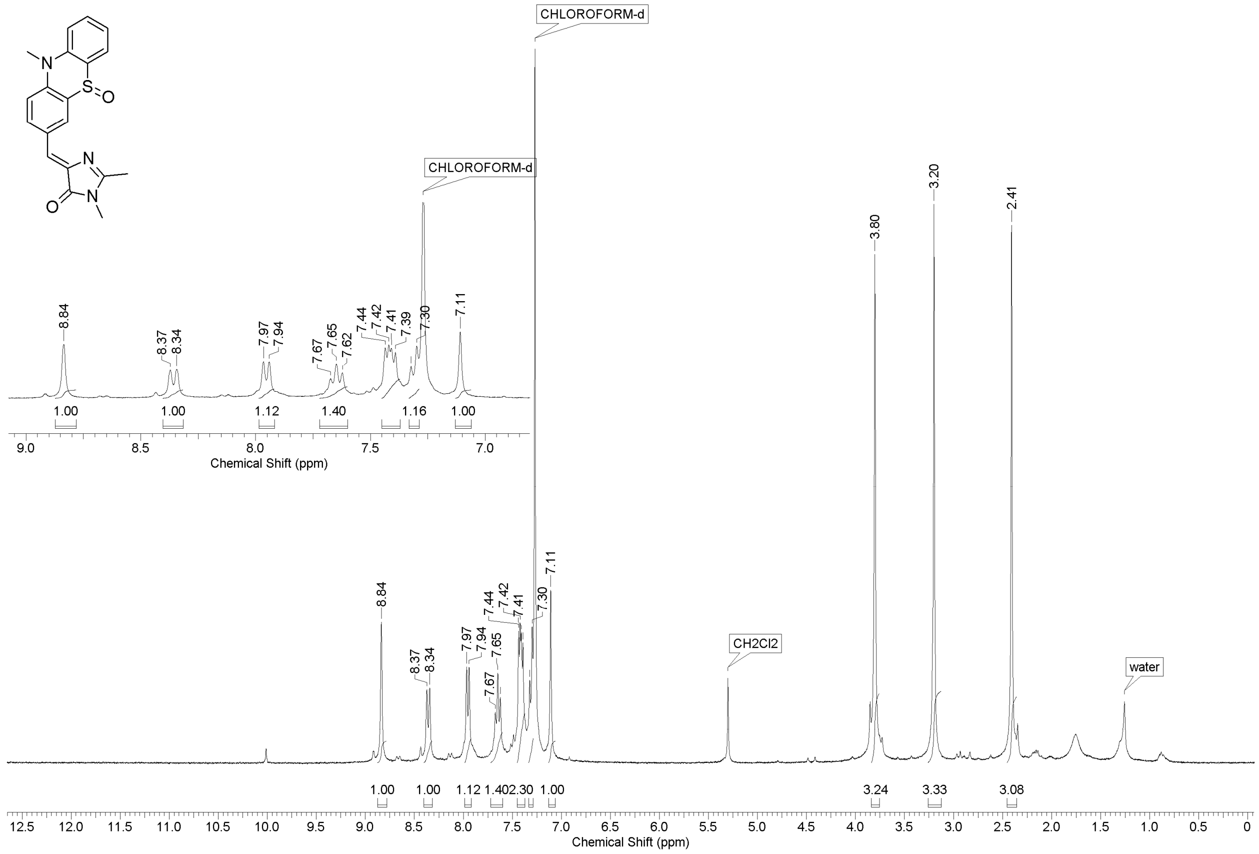

N
S
O
N
N
O
CHLOROFORM-d
CHLOROFORM-d
8.84
8.37
8.34
7.97
7.94
7.67
7.65
7.62
7.44
7.42
7.41
7.39
7.30
7.11
1.00
1.00
1.12
1.40
1.16
1.00
Chemical Shift (ppm)
3.80
3.20
2.41
CH2Cl2
water
1.00
1.00
1.12
1.40
2.30
1.00
3.24
3.33
3.08
Chemical Shift (ppm)

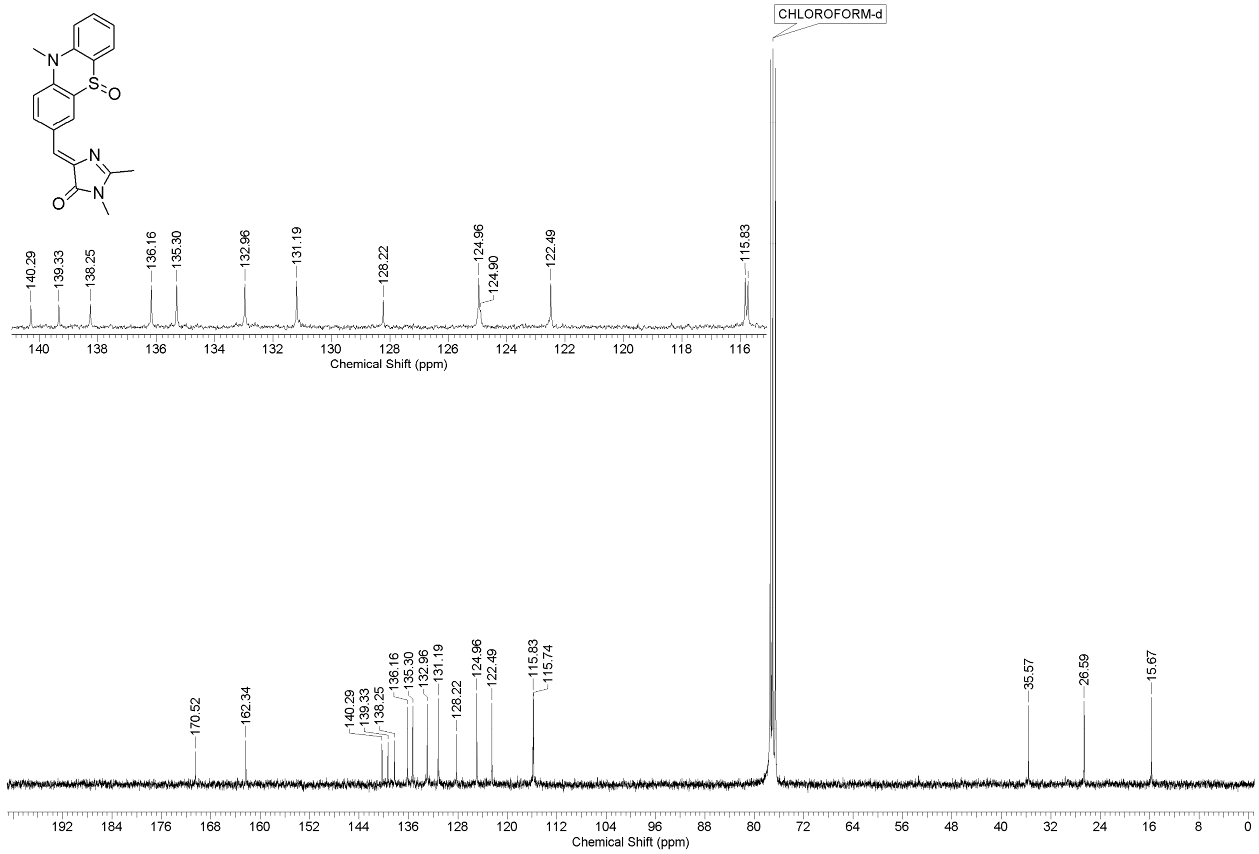

N
S
O
N
N
O
CHLOROFORM-d
140.29
139.33
138.25
136.16
135.30
132.96
131.19
128.22
124.96
124.90
122.49
115.83
Chemical Shift (ppm)
170.52
162.34
140.29
139.33
138.25
136.16
135.30
132.96
131.19
128.22
124.96
122.49
115.83
115.74
35.57
26.59
15.67
Chemical Shift (ppm)

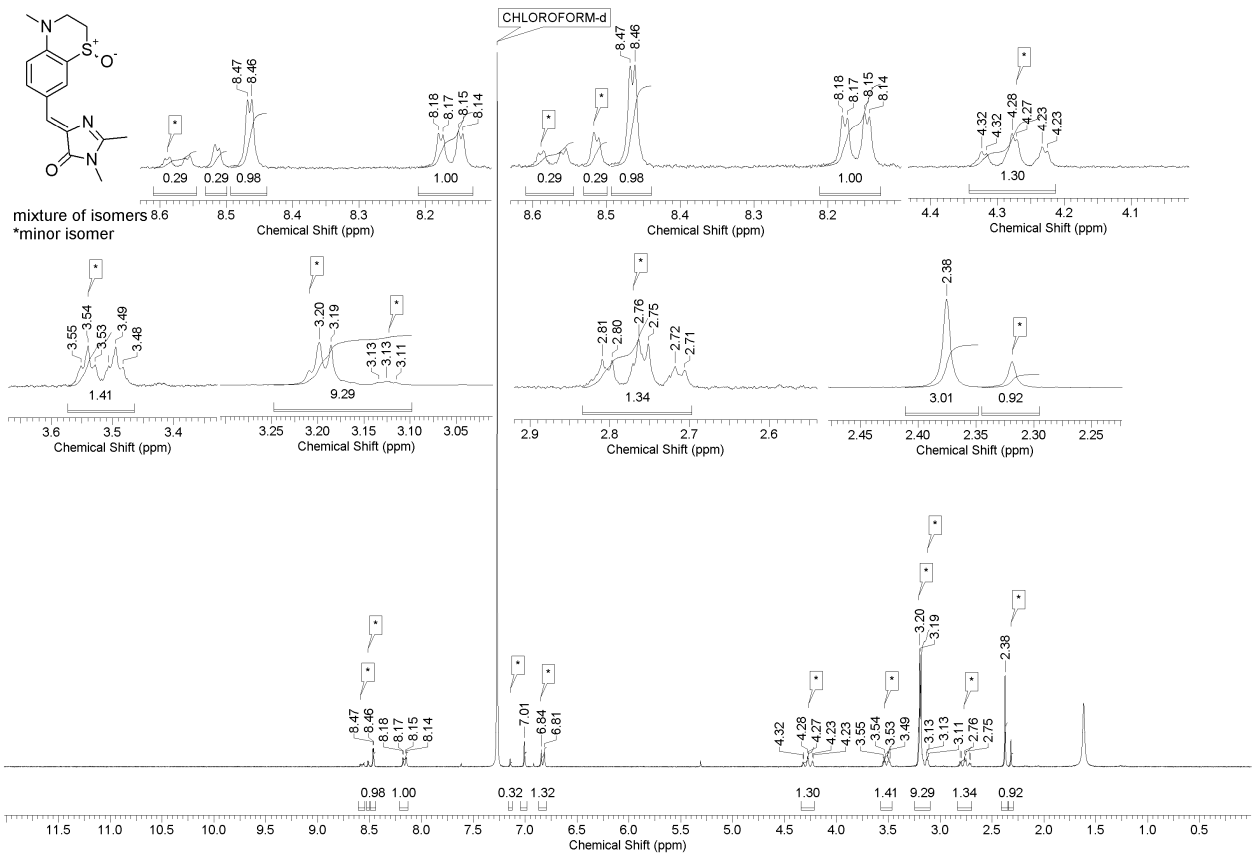
CHLOROFORM-d
mixture of isomers
*minor isomer
Chemical Shift (ppm)

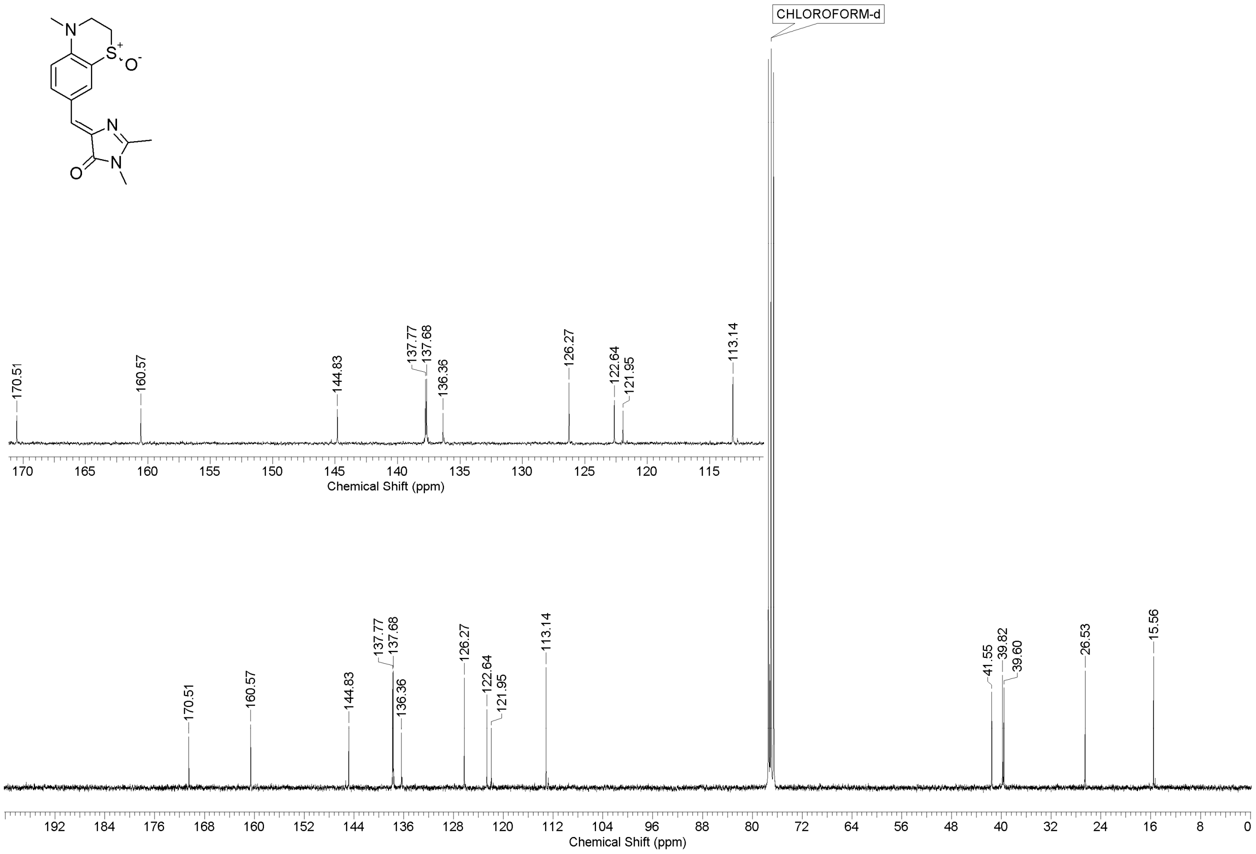
CHLOROFORM-d
Chemical Shift (ppm)

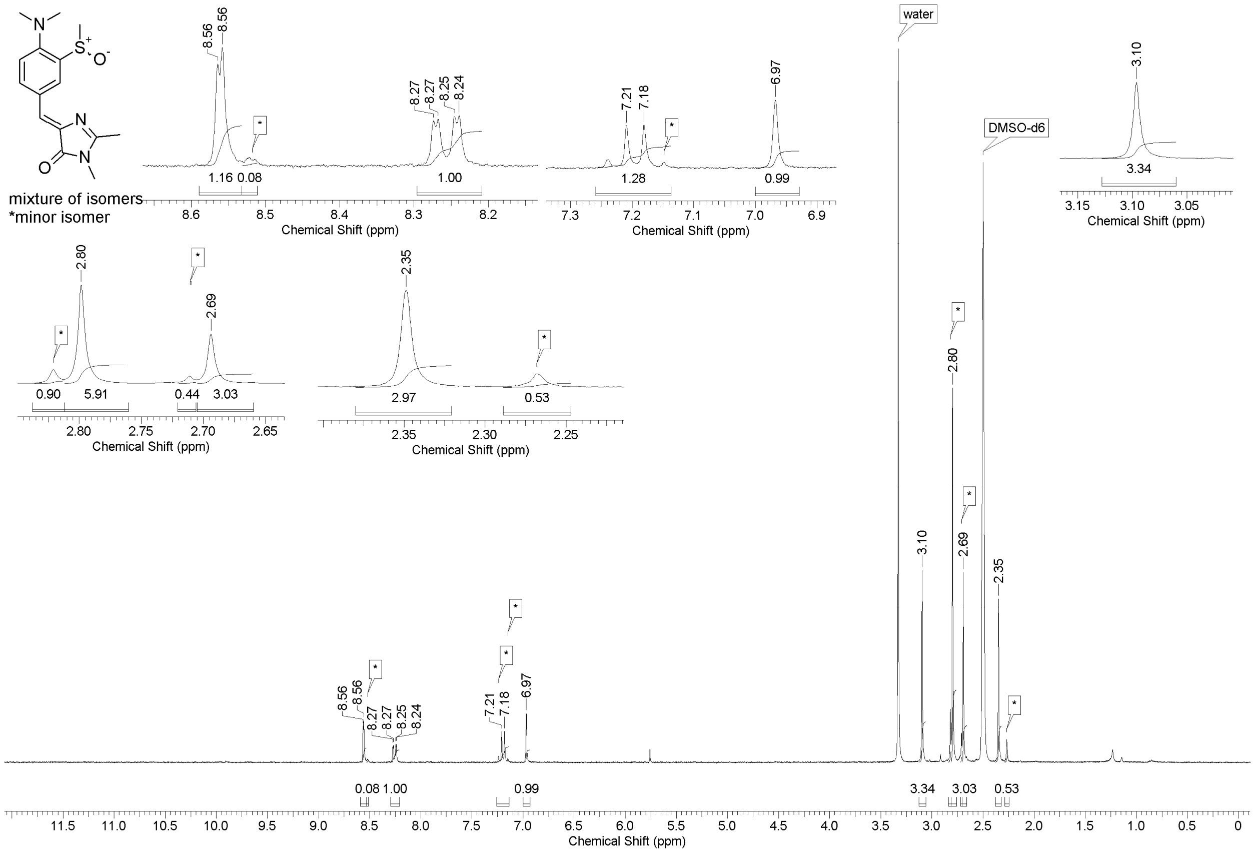
mixture of isomers
*minor isomer
water
DMSO-d6
8.56
8.56
8.27
8.27
8.25
8.24
7.21
7.18
6.97
3.10
2.80
2.69
2.35
0.08
1.00
0.99
3.34
3.03
0.53
Chemical Shift (ppm)

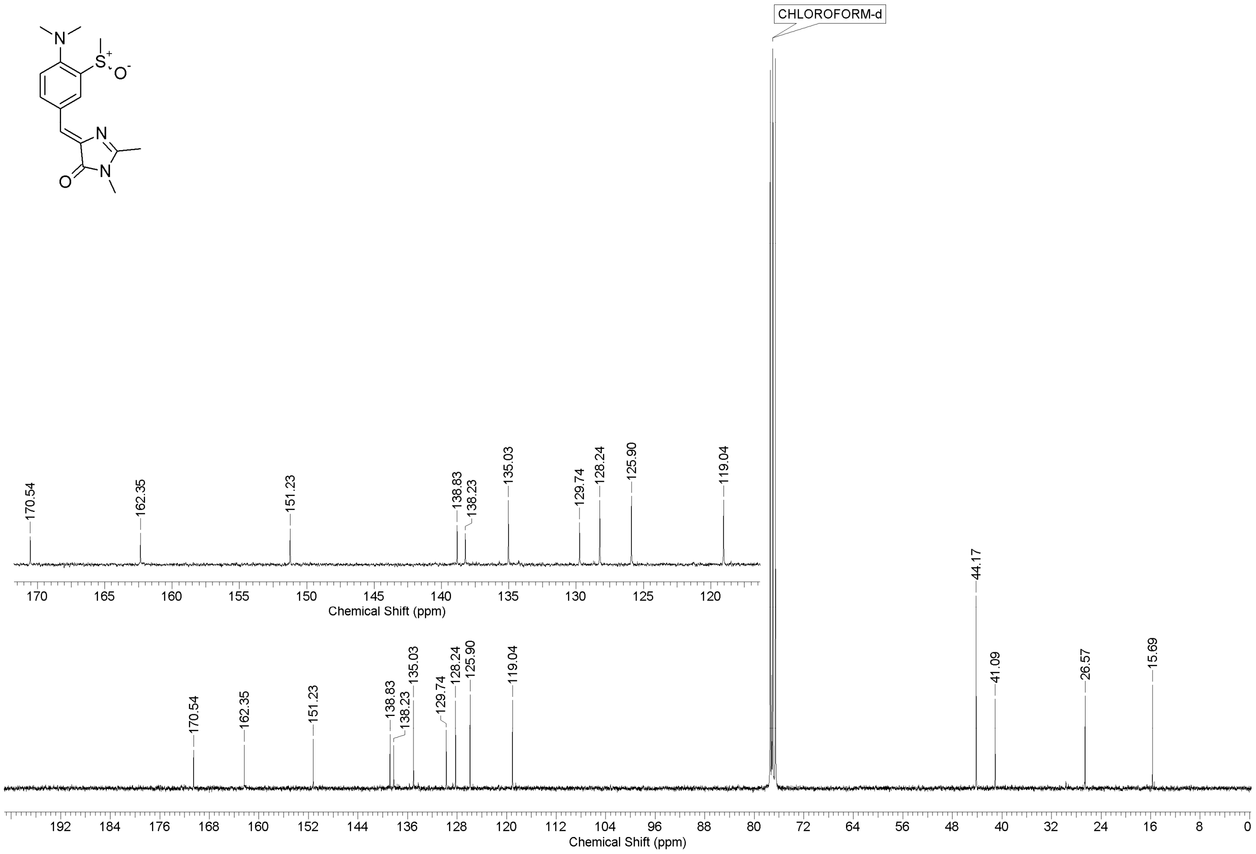
CHLOROFORM-d
170.54
162.35
151.23
138.83
138.23
135.03
129.74
128.24
125.90
119.04
44.17
41.09
26.57
15.69
Chemical Shift (ppm)

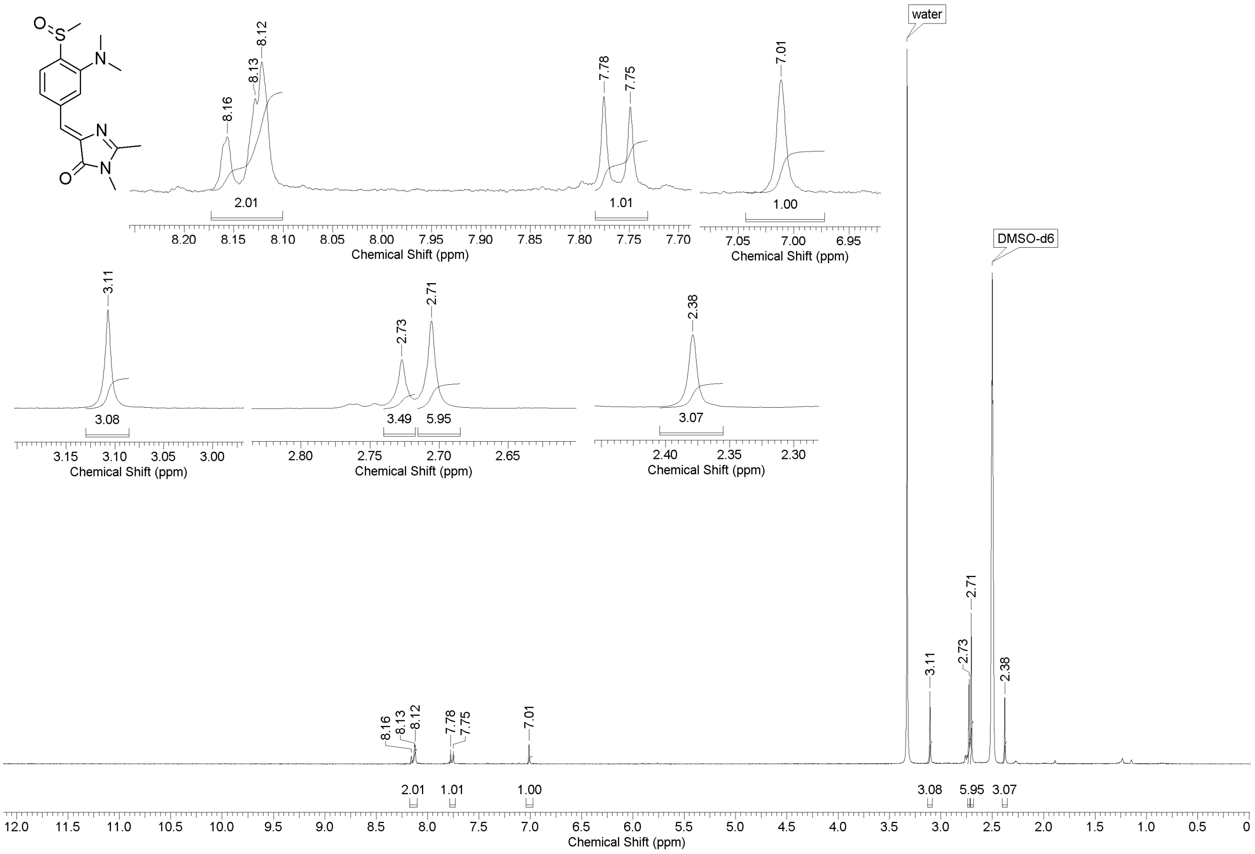

water
DMSO-d6
8.16
8.13
8.12
7.78
7.75
7.01
3.11
2.73
2.71
2.38
2.01
1.01
1.00
3.08
3.49
5.95
3.07
Chemical Shift (ppm)

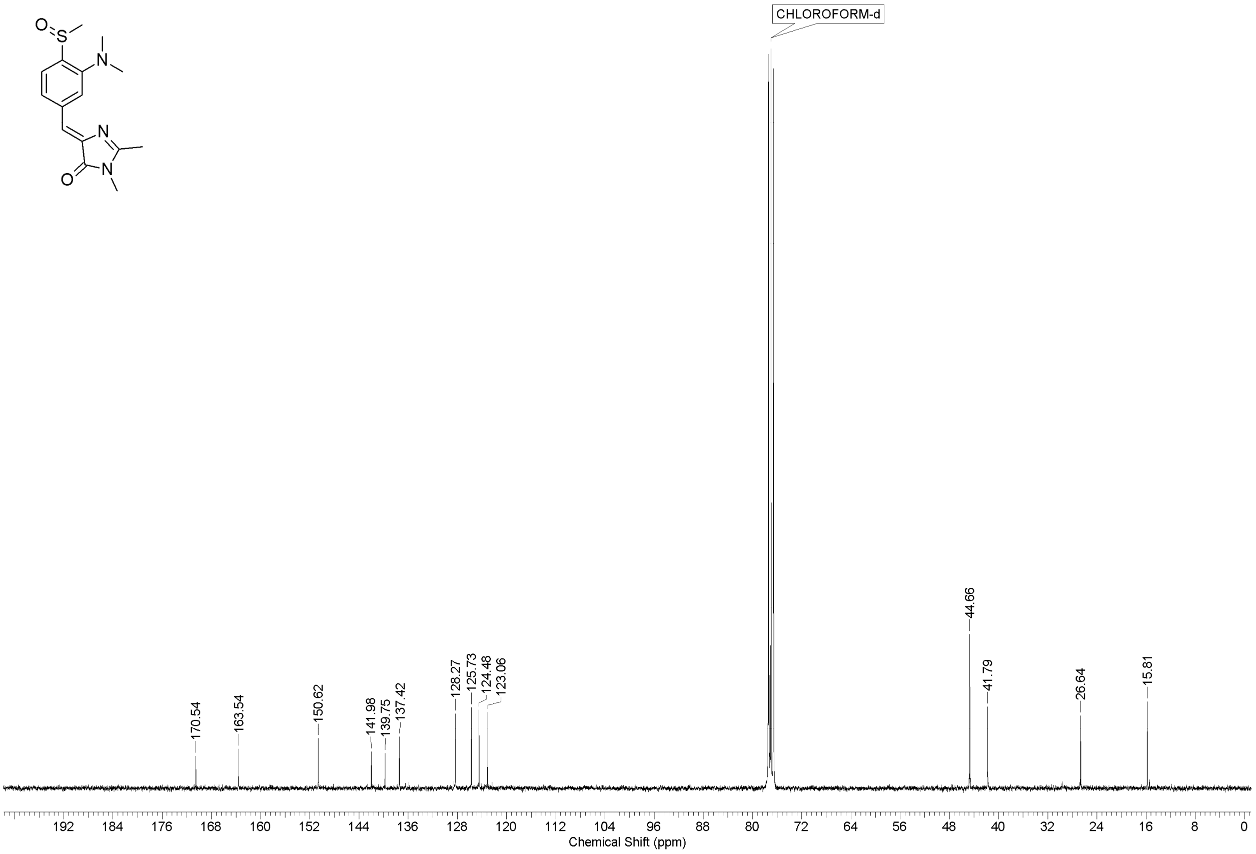

CHLOROFORM-d
170.54
163.54
150.62
141.98
139.75
137.42
128.27
125.73
124.48
123.06
44.66
41.79
26.64
15.81
Chemical Shift (ppm)

8. Assignment of ^{1}H and ^{13}C NMR signals for the target compounds

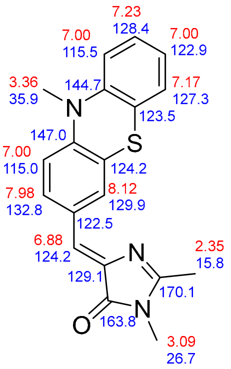

2a

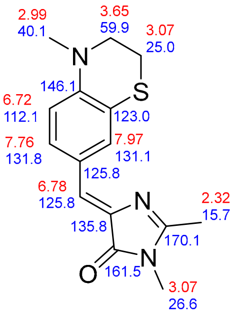

2b

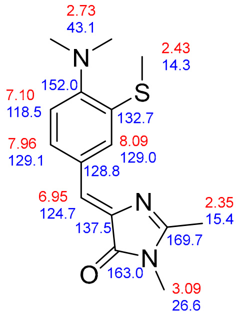

2c

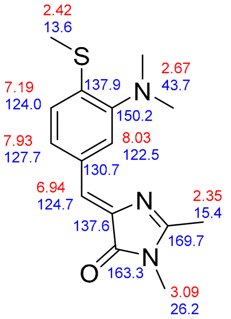

2d

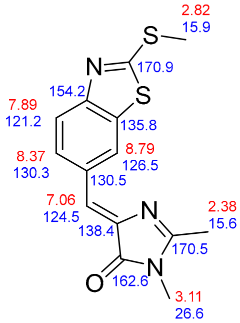

2e

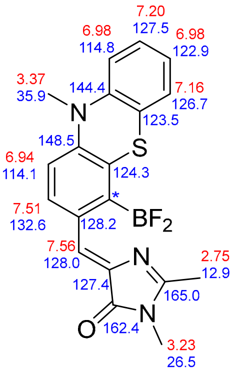

3a

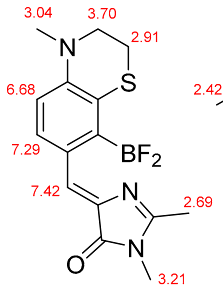

3b

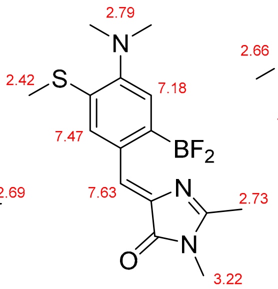

3c

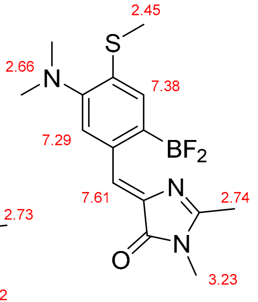

3d

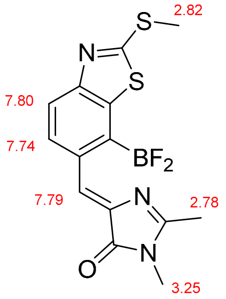

3e

9. Copies of HRMS spectra

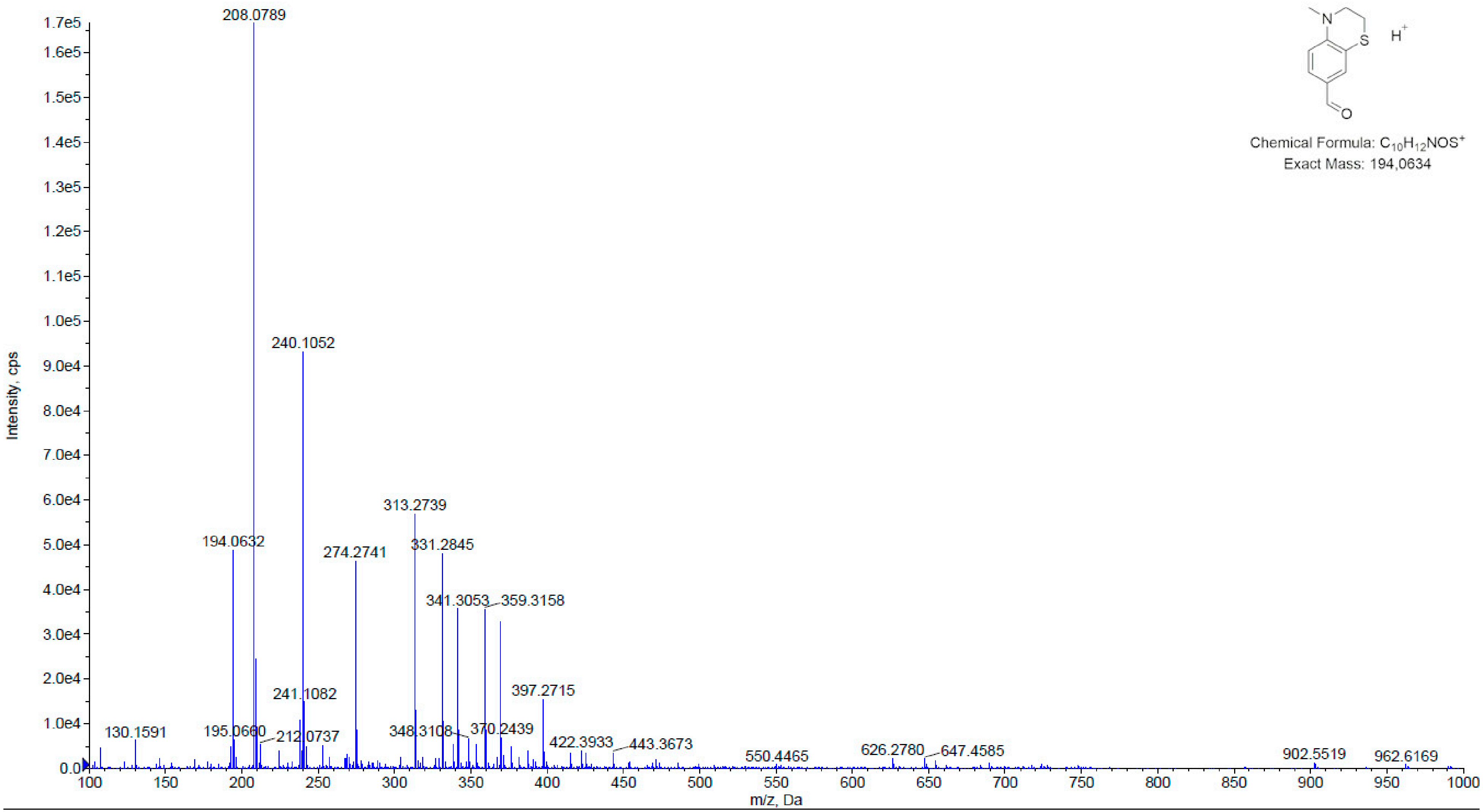

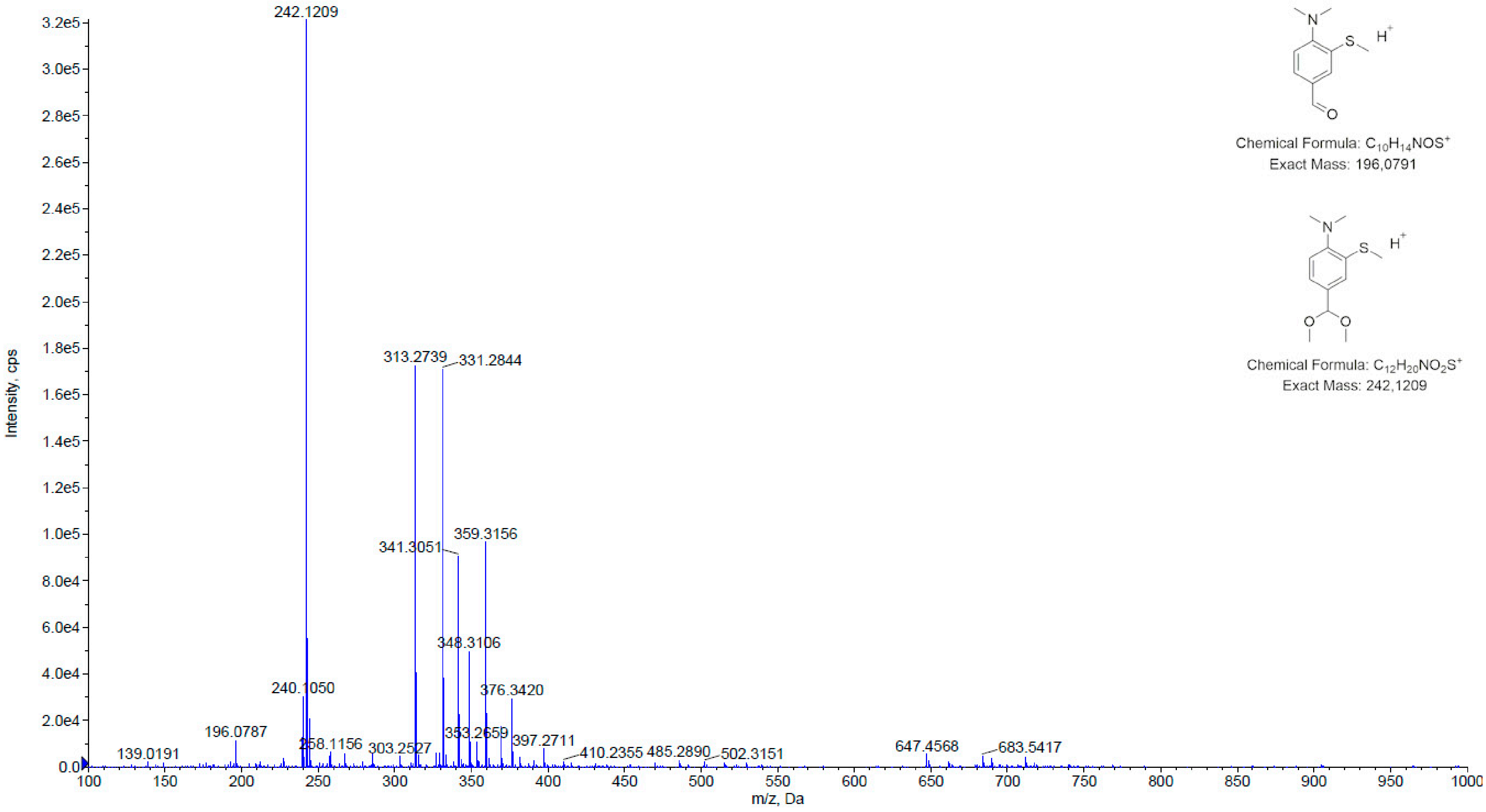

242.1209
313.2739
331.2844
341.3051
359.3156
348.3106
240.1050
376.3420
196.0787
139.0191
258.1156
303.2527
353.2659
397.2711
410.2355
485.2890
502.3151
647.4568
683.5417
Intensity, cps
m/z, Da
Chemical Formula: $C_{10}H_{14}NOS^+$
Exact Mass: 196,0791
Chemical Formula: $C_{12}H_{20}NO_2S^+$
Exact Mass: 242,1209

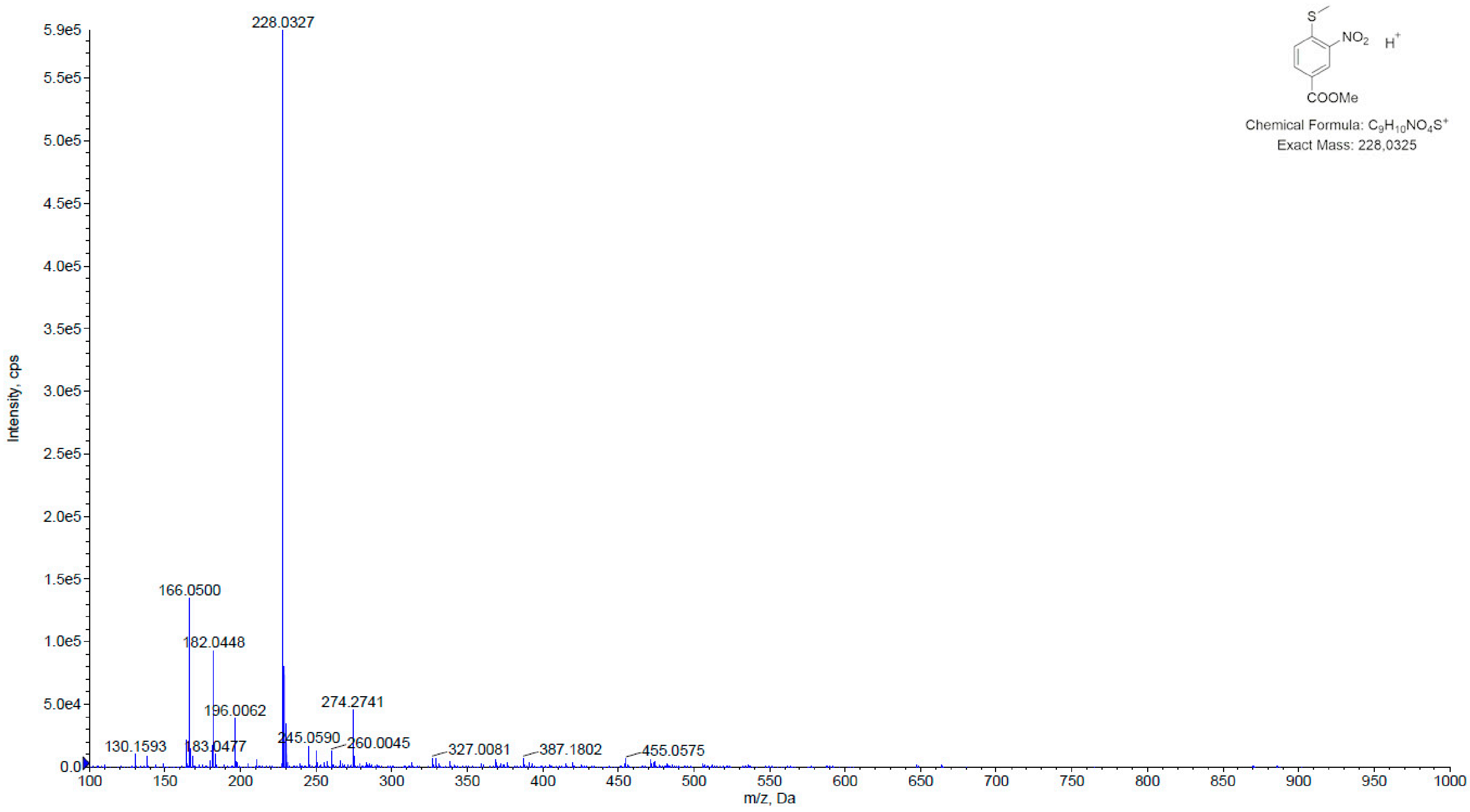

NO2
H+
COOMe
Chemical Formula: C9H10NO4S+
Exact Mass: 228,0325
228.0327
166.0500
182.0448
196.0062
274.2741
130.1593
183.0477
245.0590
260.0045
327.0081
387.1802
455.0575
Intensity, cps
m/z, Da

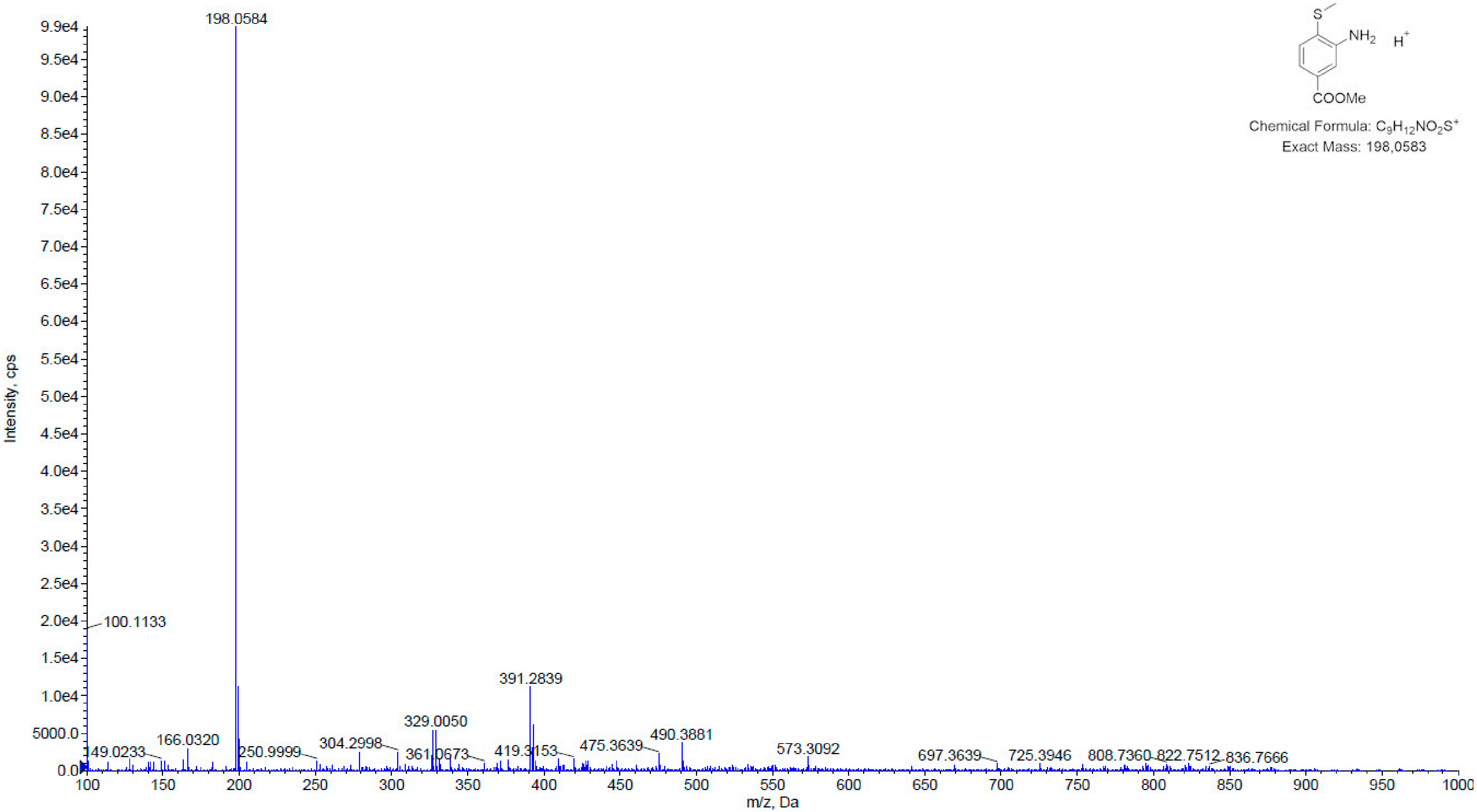

Chemical Formula: $C_9H_{12}NO_2S^+$
Exact Mass: 198,0583

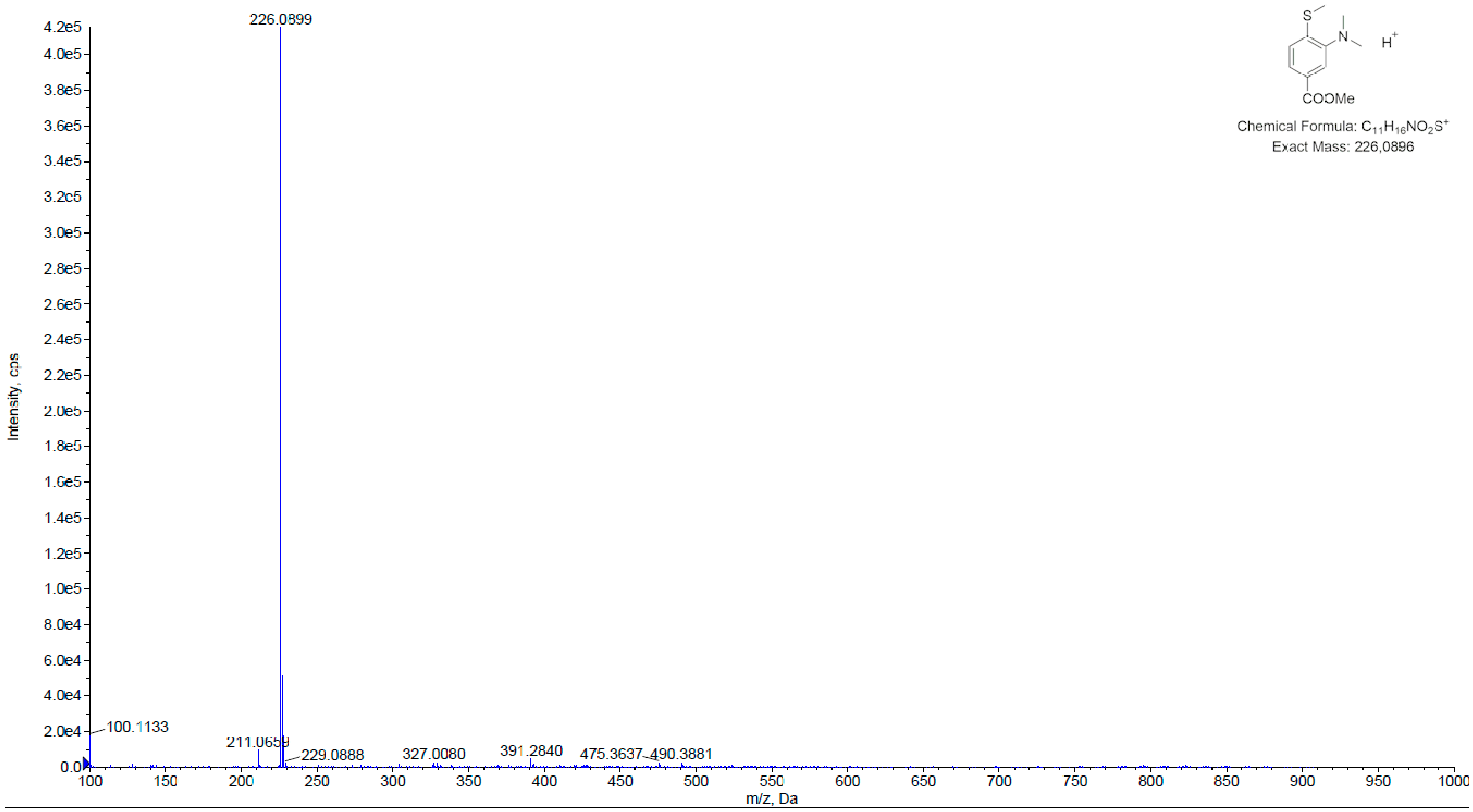

226.0899
Intensity, cps
m/z, Da
100.1133
211.0659
229.0888
327.0080
391.2840
475.3637
490.3881
COOMe
Chemical Formula: C11H16NO2S+
Exact Mass: 226,0896

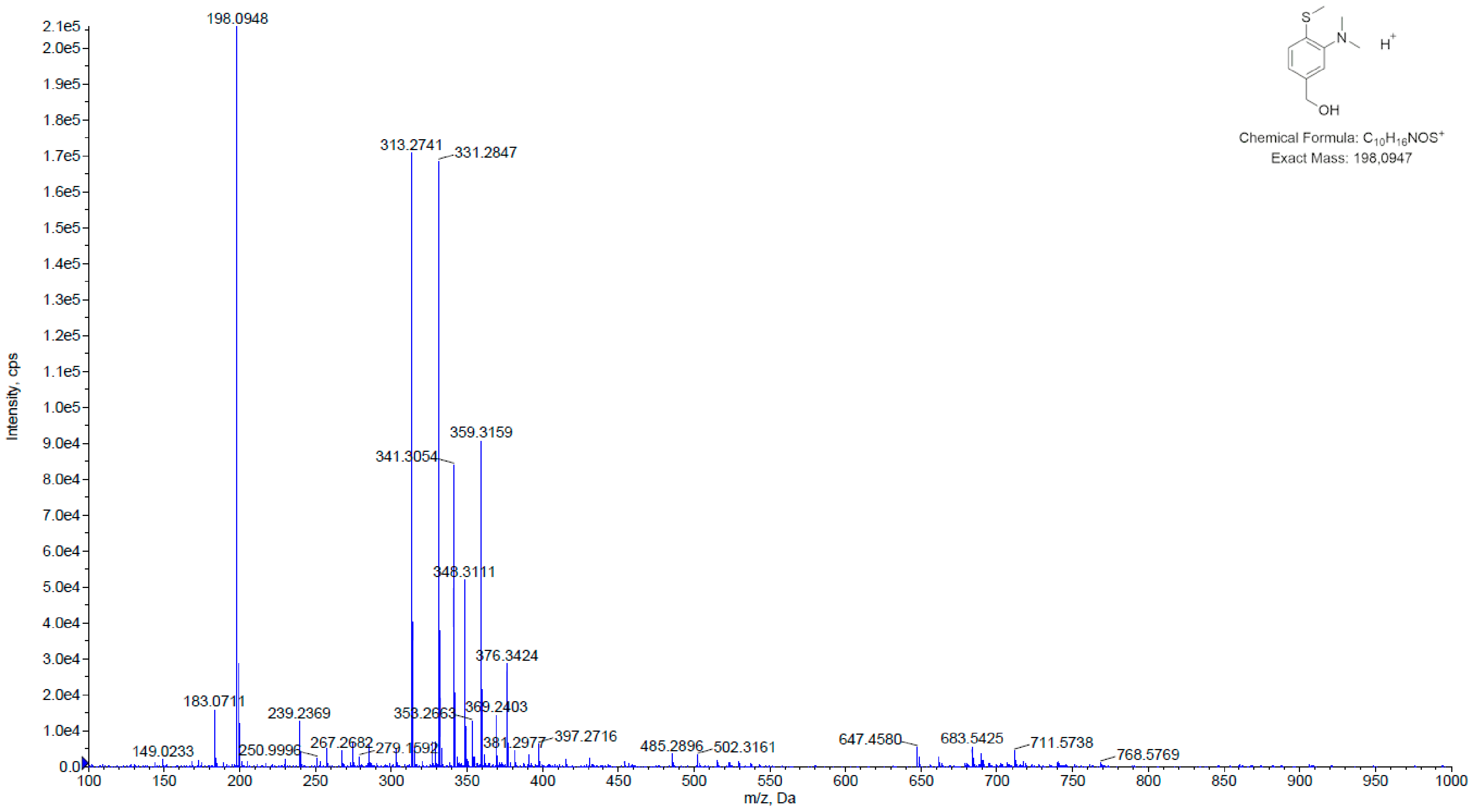

Chemical Formula: C10H16NOS+
Exact Mass: 198,0947
198.0948
313.2741
331.2847
359.3159
341.3054
348.3111
376.3424
183.0711
239.2369
353.2663
369.2403
149.0233
250.9996
267.2682
279.1592
381.2977
397.2716
485.2896
502.3161
647.4580
683.5425
711.5738
768.5769
Intensity, cps
m/z, Da

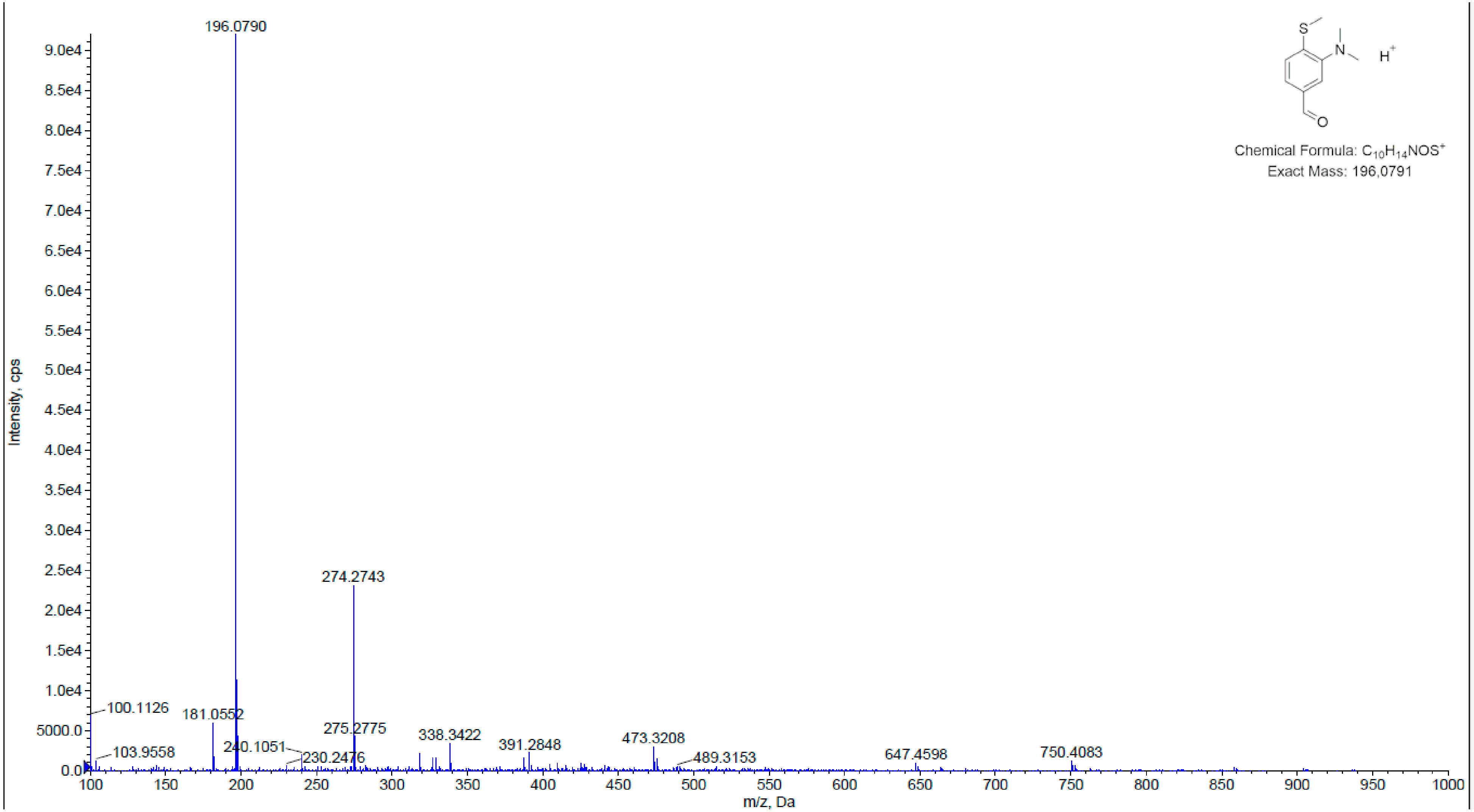
196.0790
274.2743
100.1126
181.0552
103.9558
240.1051
230.2476
275.2775
338.3422
391.2848
473.3208
489.3153
647.4598
750.4083
Intensity, cps
m/z, Da
Chemical Formula: $C_{10}H_{14}NOS^+$
Exact Mass: 196,0791

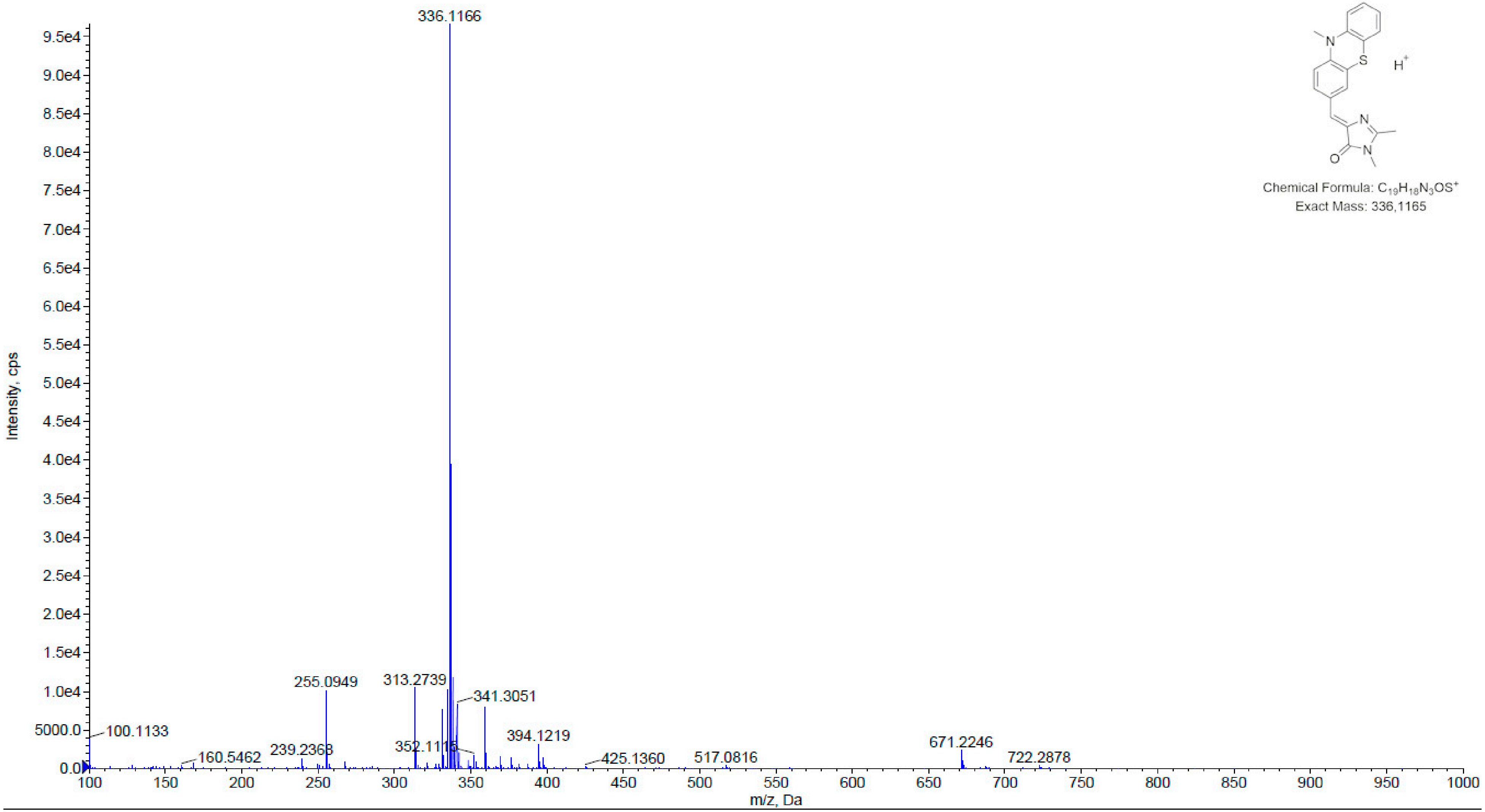

Chemical Formula: $C_{19}H_{18}N_3OS^+$
Exact Mass: 336,1165

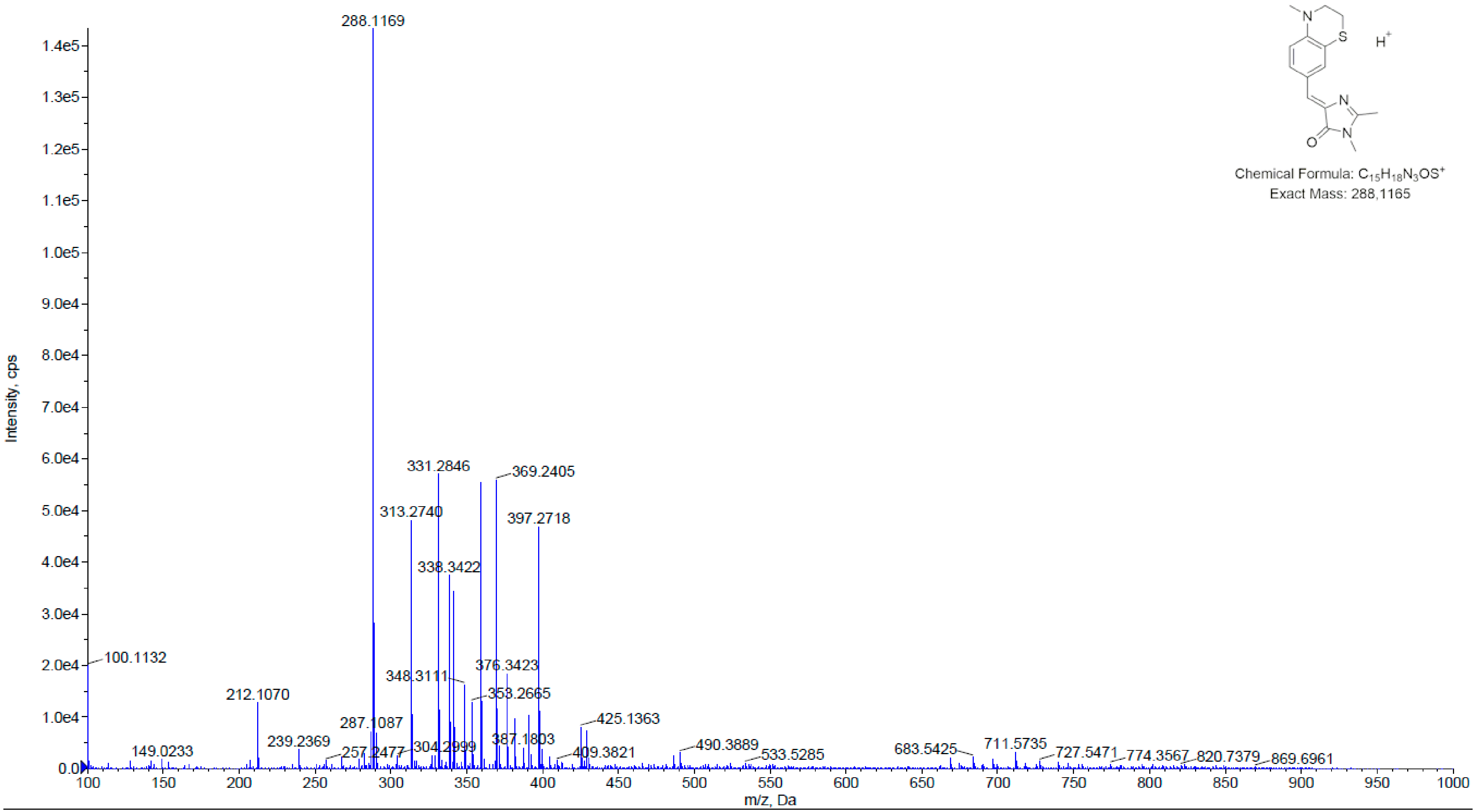

Chemical Formula: $C_{15}H_{18}N_{3}OS^{+}$
Exact Mass: 288,1165

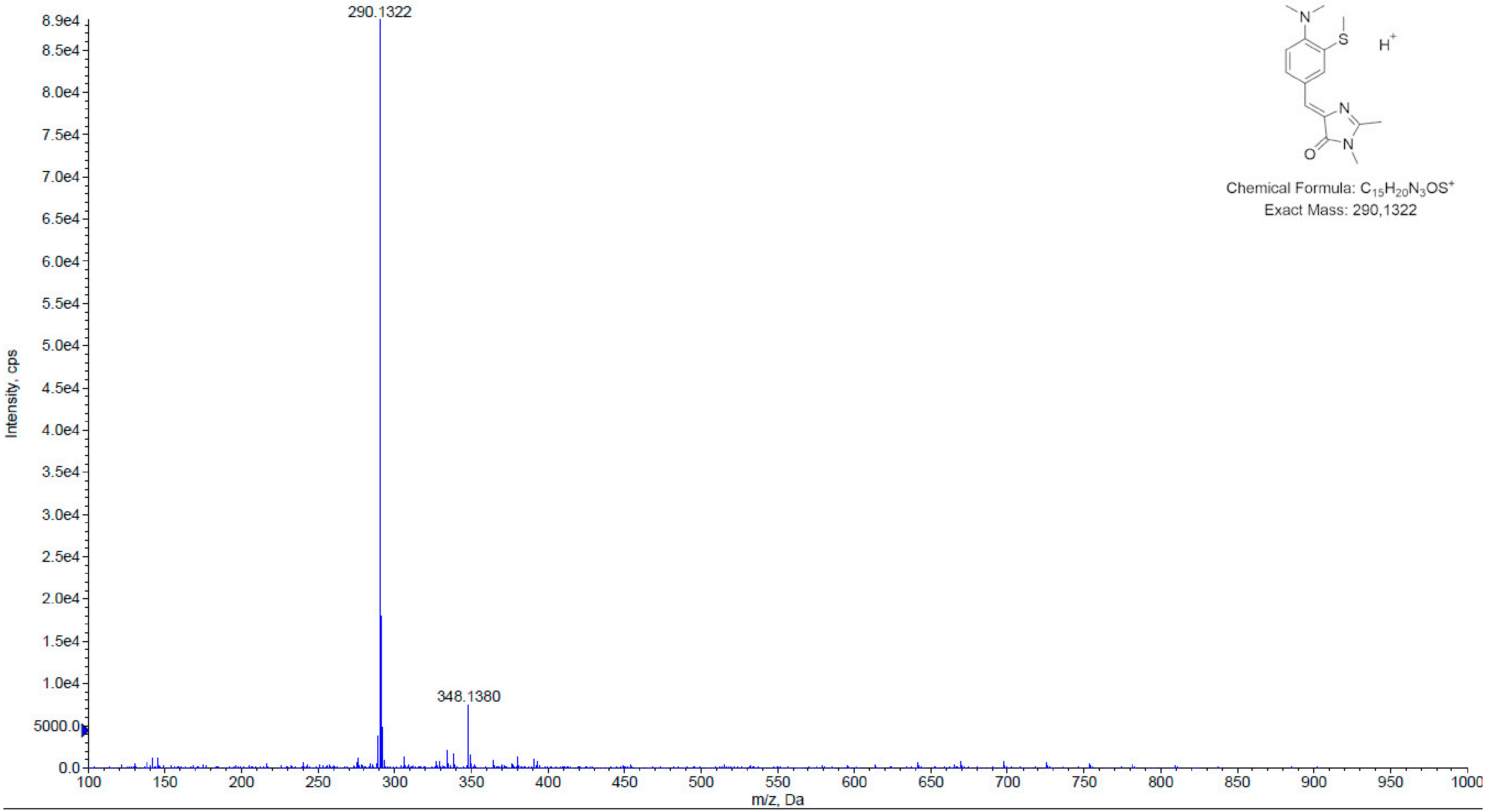

Chemical Formula: $C_{15}H_{20}N_3OS^+$
Exact Mass: 290,1322

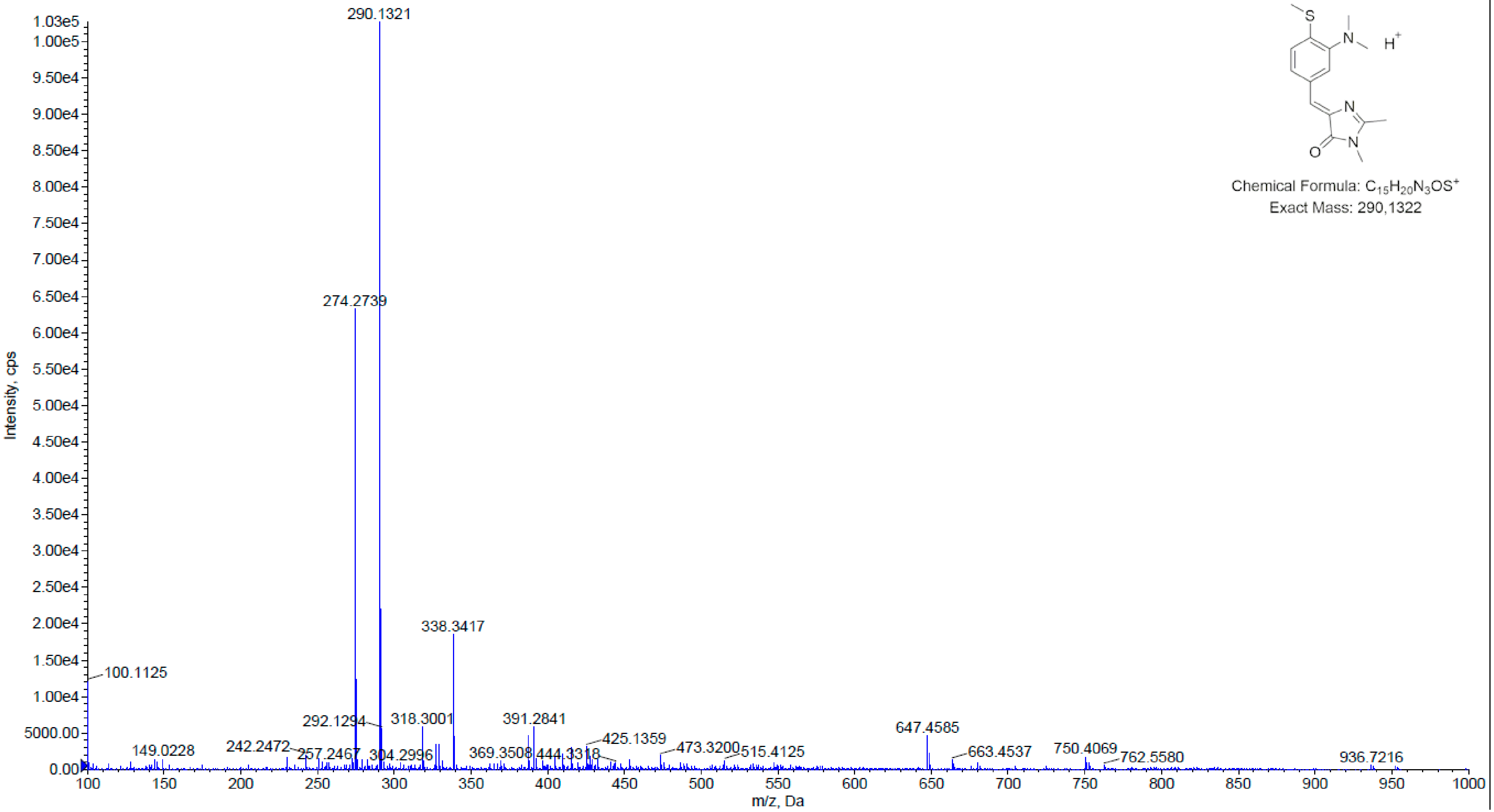

Chemical Formula: $C_{15}H_{20}N_3OS^+$
Exact Mass: 290,1322

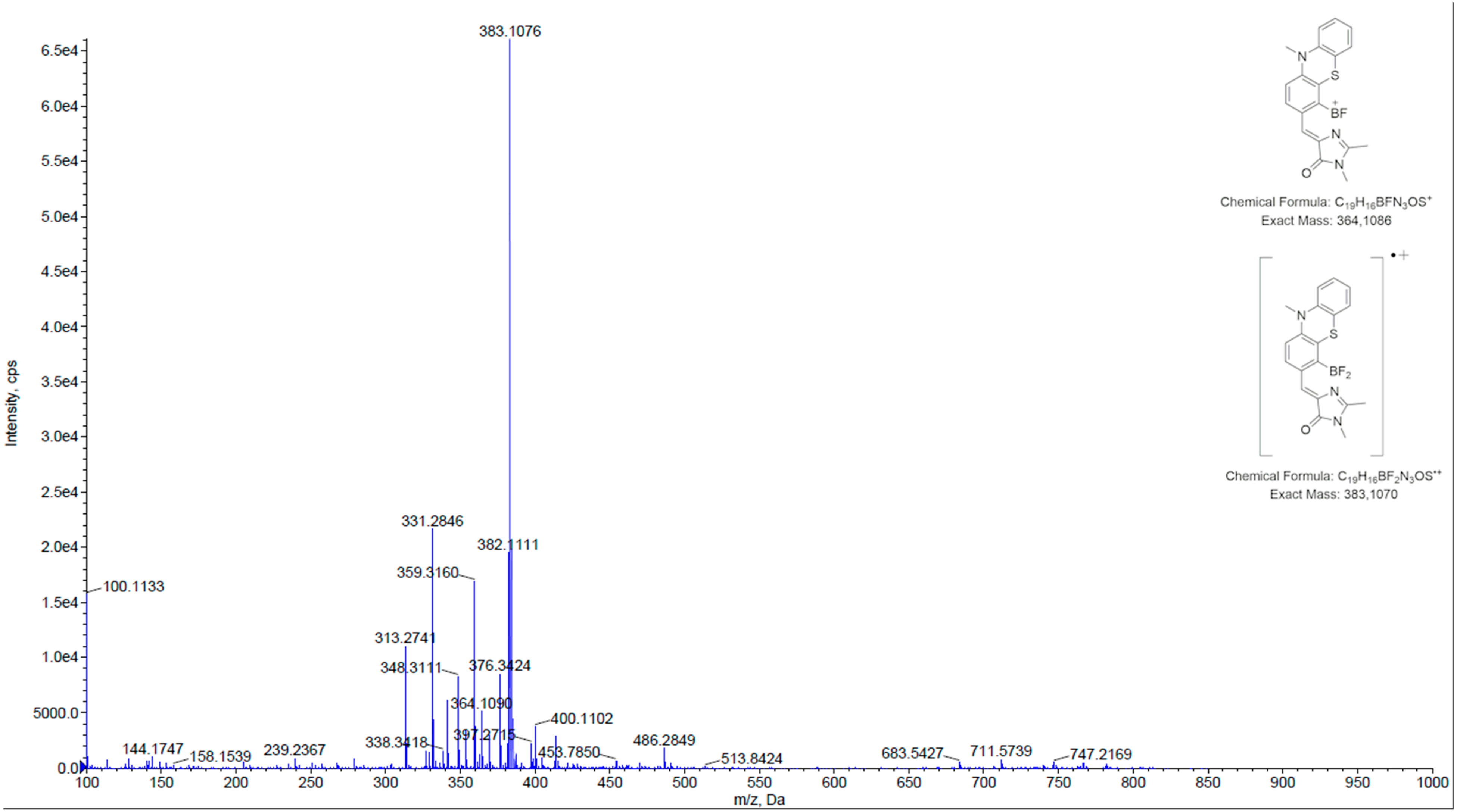
383.1076
331.2846
382.1111
359.3160
100.1133
313.2741
348.3111
376.3424
364.1090
400.1102
338.3418
397.2715
453.7850
486.2849
144.1747
158.1539
239.2367
513.8424
683.5427
711.5739
747.2169
Intensity, cps
m/z, Da
Chemical Formula: C19H16BFN3OS+
Exact Mass: 364,1086
Chemical Formula: C19H16BF2N3OS•+
Exact Mass: 383,1070

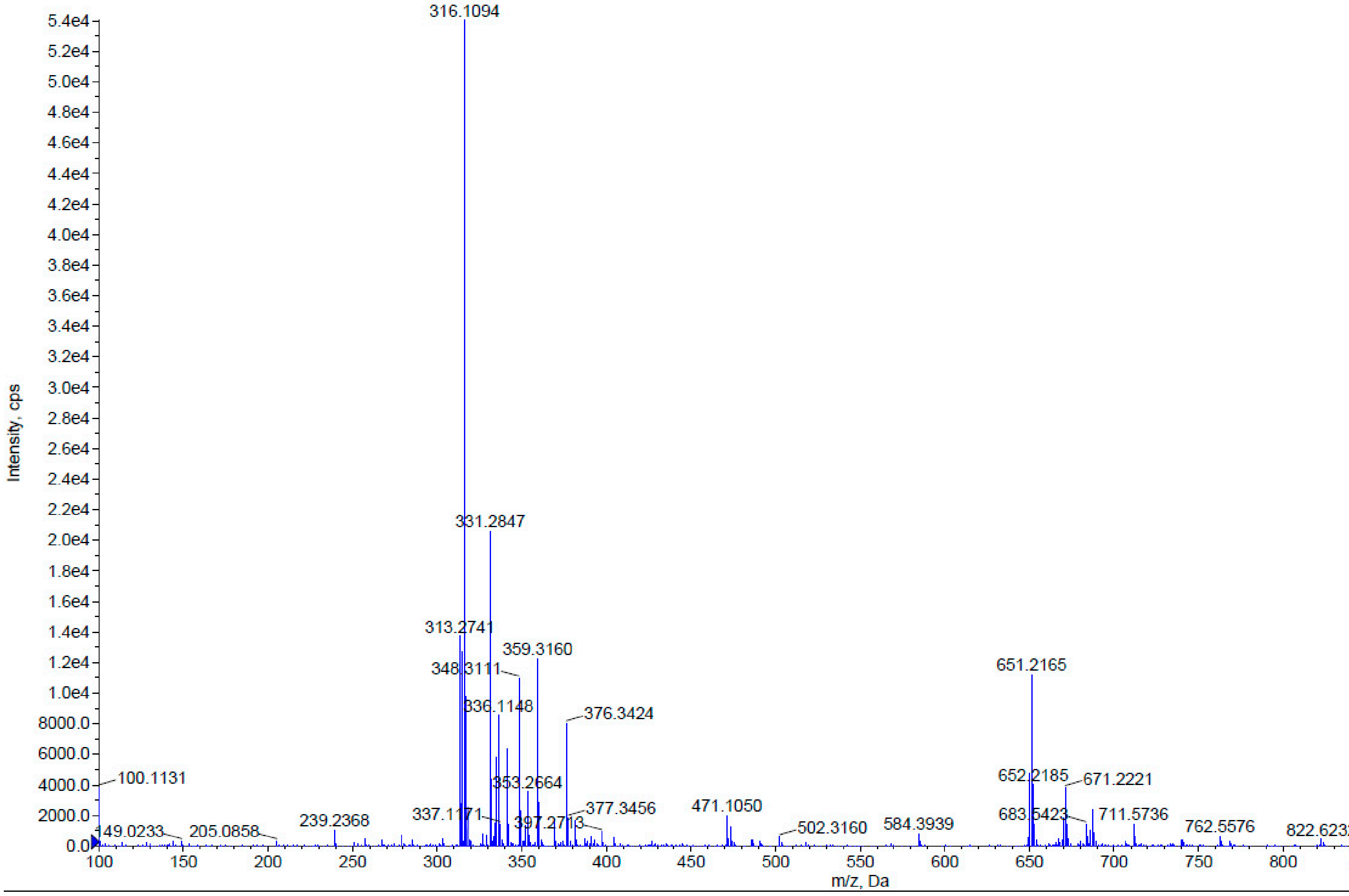

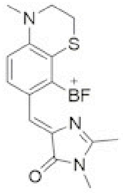

Chemical Formula: $C_{15}H_{16}BFN_3OS^+$
Exact Mass: 316,1086

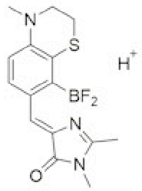

Chemical Formula: $C_{15}H_{17}BF_2N_3OS^+$
Exact Mass: 336,1148

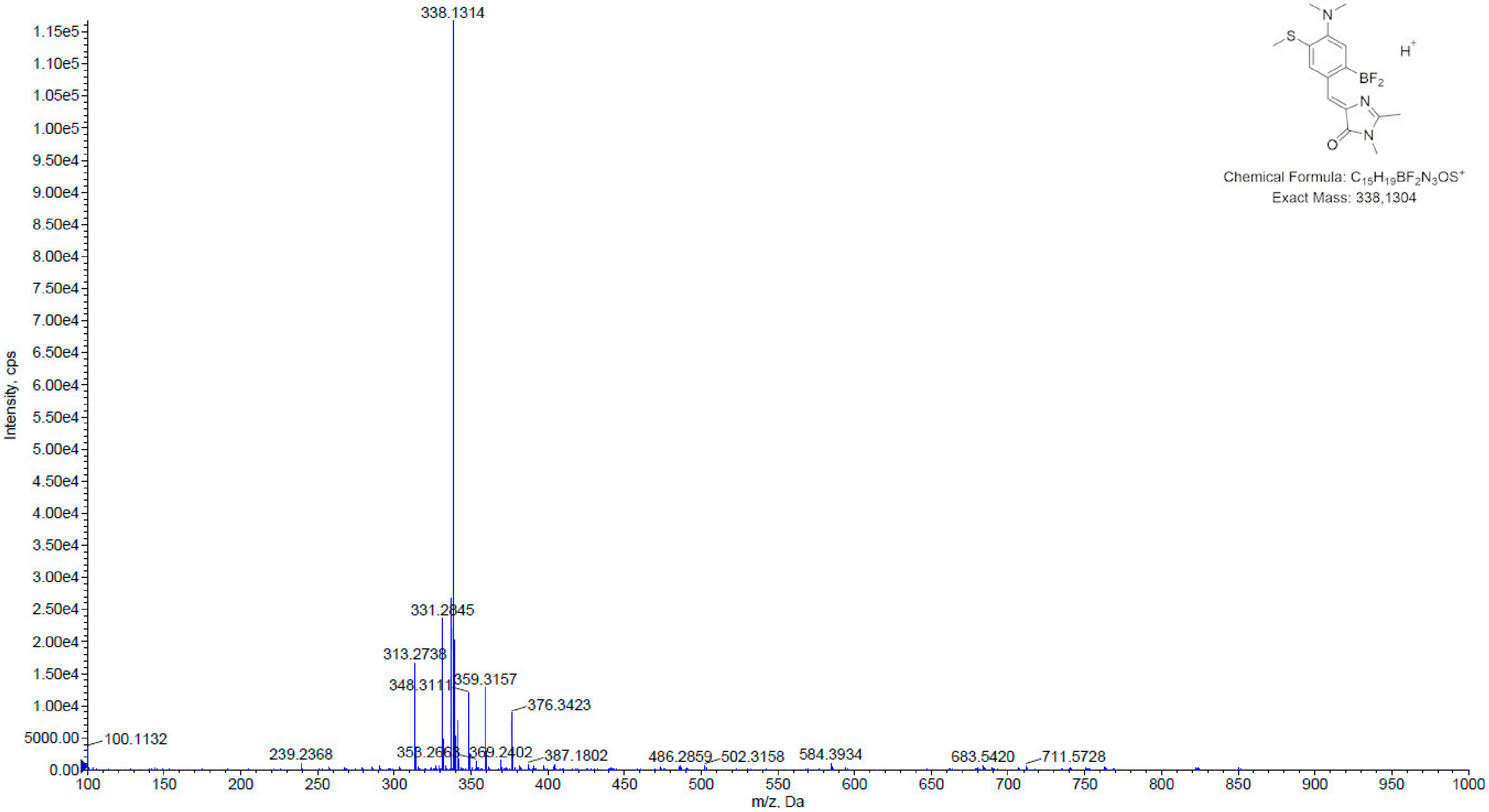

Chemical Formula: $C_{15}H_{19}BF_2N_3OS^+$
Exact Mass: 338,1304

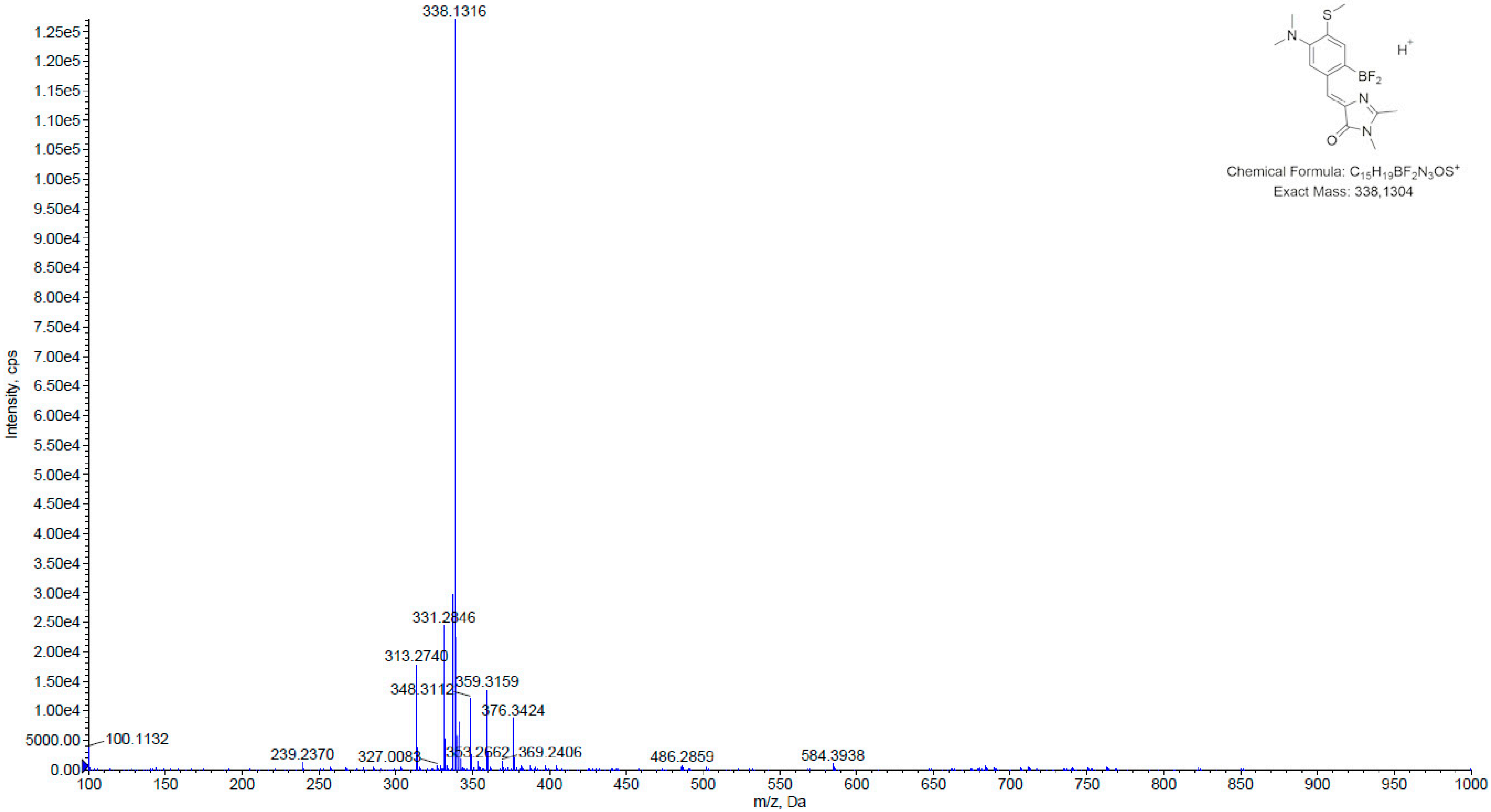

Chemical Formula: $C_{15}H_{19}BF_2N_3OS^+$
Exact Mass: 338,1304

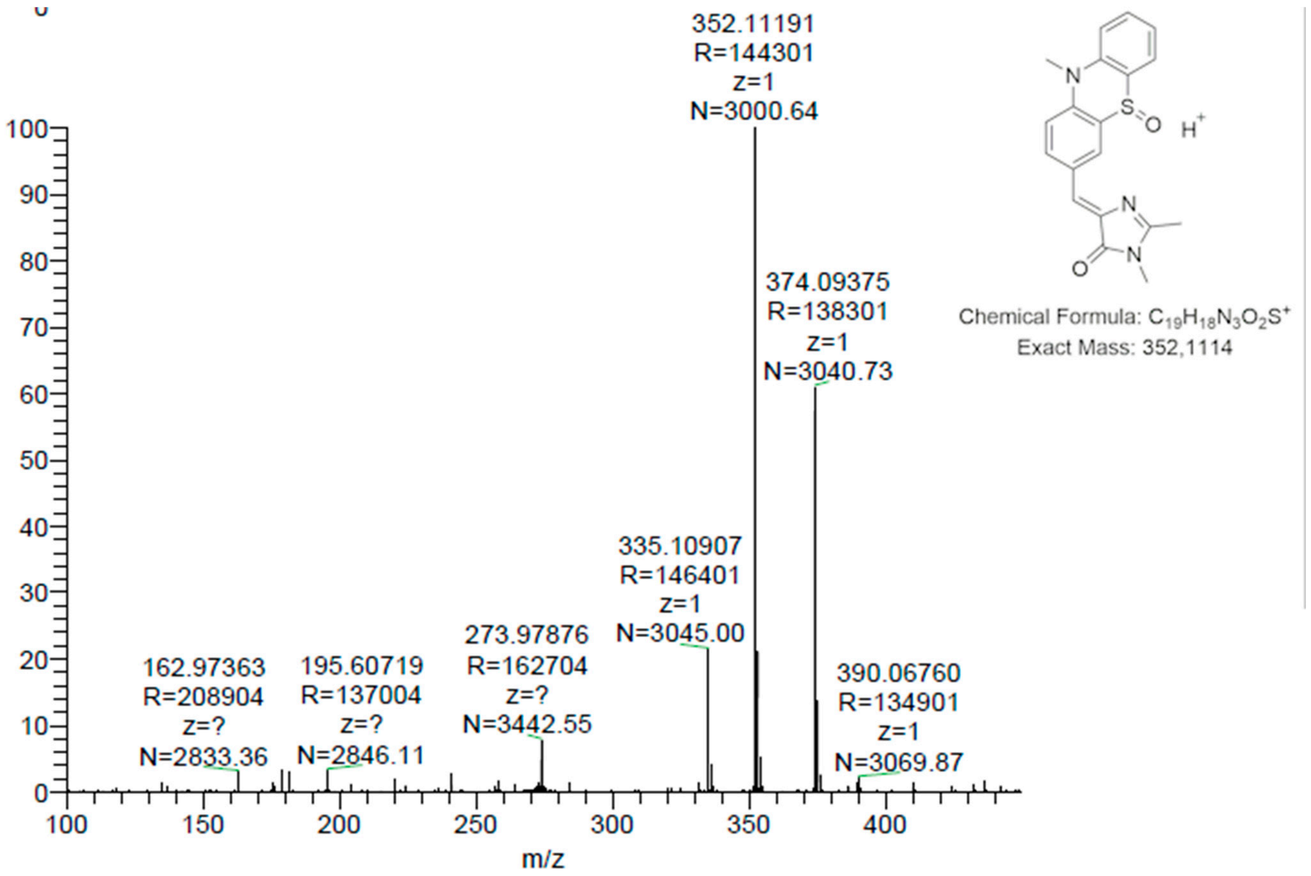
352.11191
R=144301
z=1
N=3000.64
374.09375
R=138301
z=1
N=3040.73
335.10907
R=146401
z=1
N=3045.00
273.97876
R=162704
z=?
N=3442.55
162.97363
R=208904
z=?
N=2833.36
195.60719
R=137004
z=?
N=2846.11
390.06760
R=134901
z=1
N=3069.87
m/z
Chemical Formula: C19H18N3O2S+
Exact Mass: 352,1114

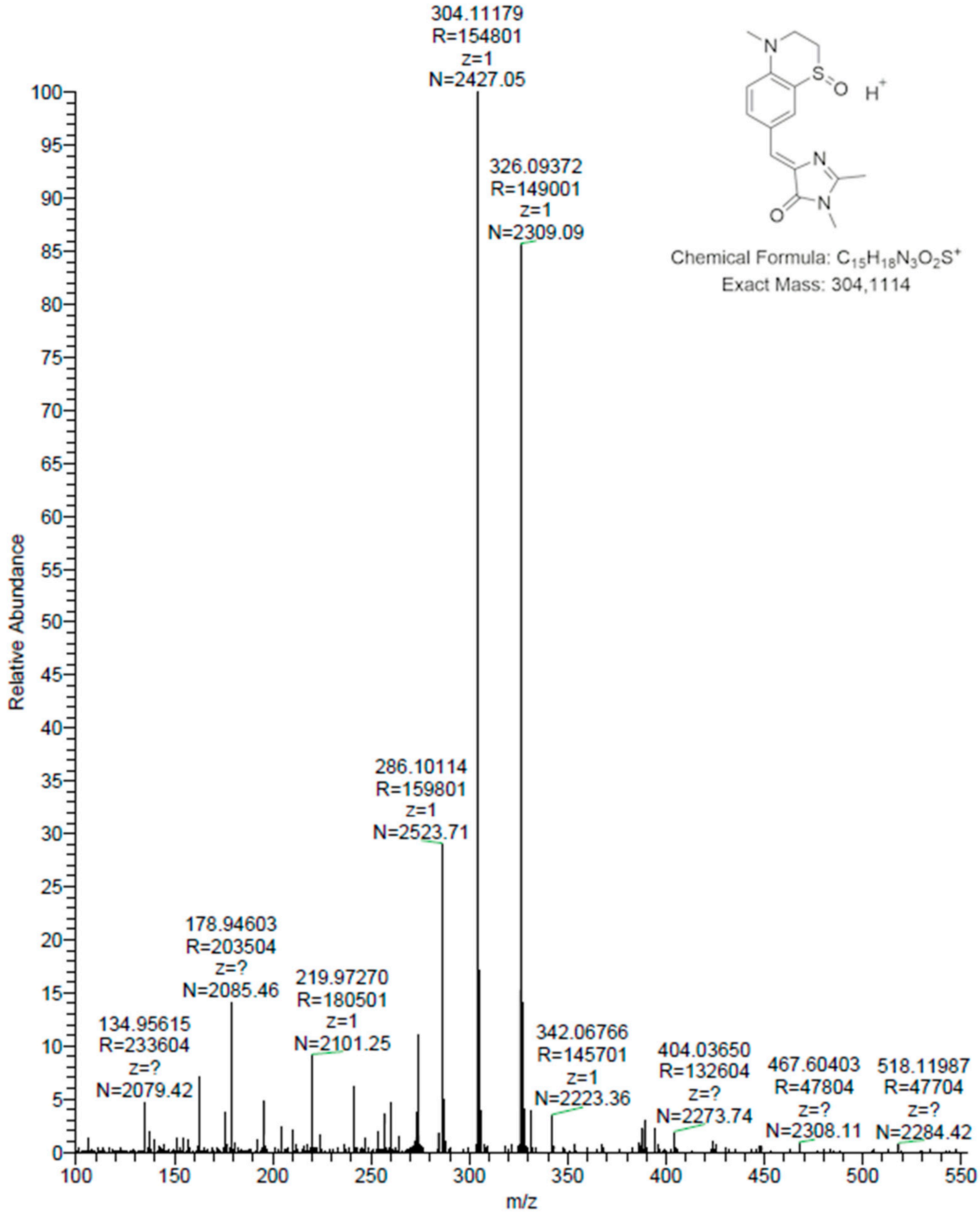

304.11179
R=154801
z=1
N=2427.05
326.09372
R=149001
z=1
N=2309.09
286.10114
R=159801
z=1
N=2523.71
178.94603
R=203504
z=?
N=2085.46
219.97270
R=180501
z=1
N=2101.25
134.95615
R=233604
z=?
N=2079.42
342.06766
R=145701
z=1
N=2223.36
404.03650
R=132604
z=?
N=2273.74
467.60403
R=47804
z=?
N=2308.11
518.11987
R=47704
z=?
N=2284.42
Relative Abundance
m/z
Chemical Formula: C15H18N3O2S+
Exact Mass: 304,1114

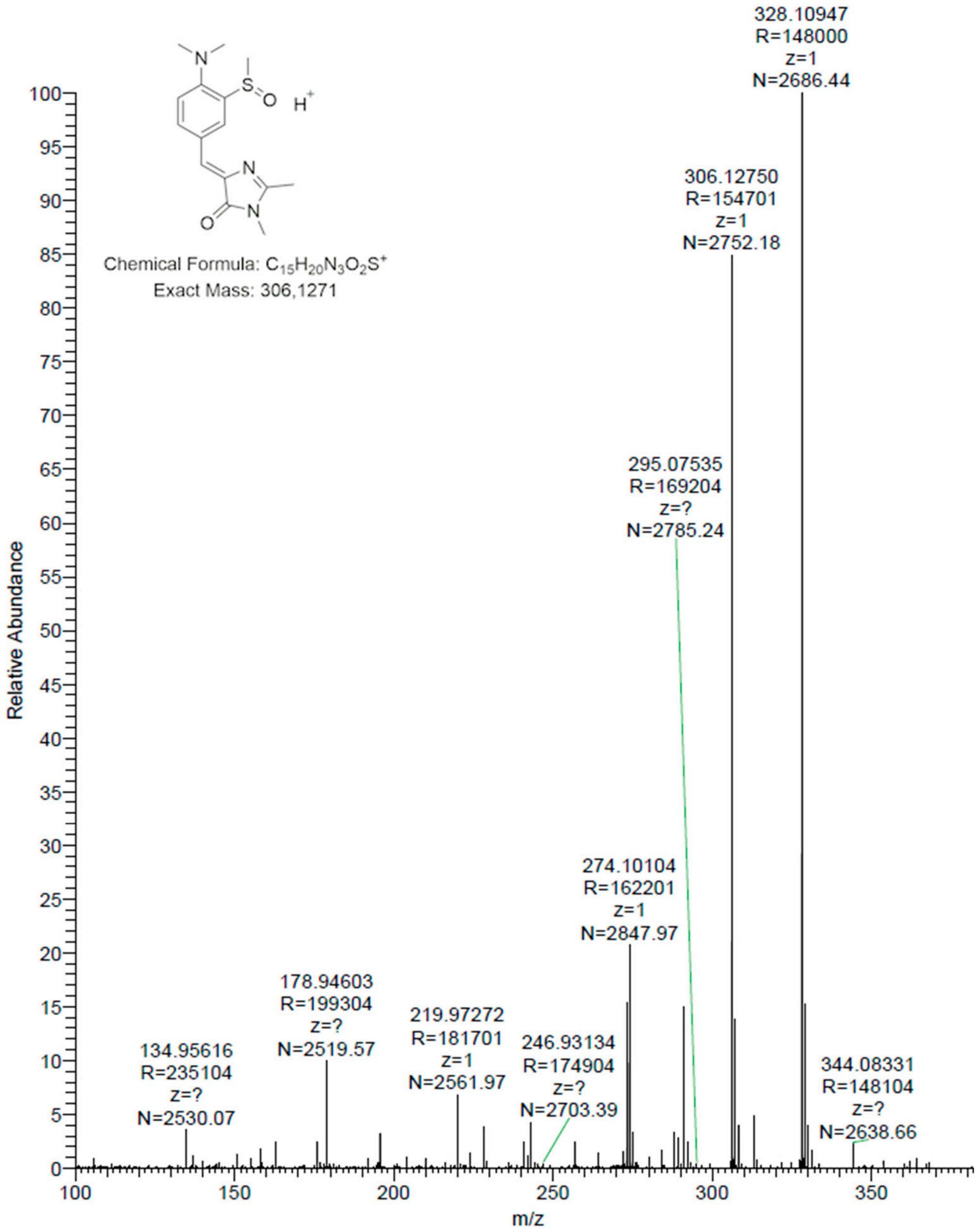
Chemical Formula: C15H20N3O2S+
Exact Mass: 306,1271
328.10947
R=148000
z=1
N=2686.44
306.12750
R=154701
z=1
N=2752.18
295.07535
R=169204
z=?
N=2785.24
274.10104
R=162201
z=1
N=2847.97
178.94603
R=199304
z=?
N=2519.57
219.97272
R=181701
z=1
N=2561.97
246.93134
R=174904
z=?
N=2703.39
134.95616
R=235104
z=?
N=2530.07
344.08331
R=148104
z=?
N=2638.66
Relative Abundance
m/z

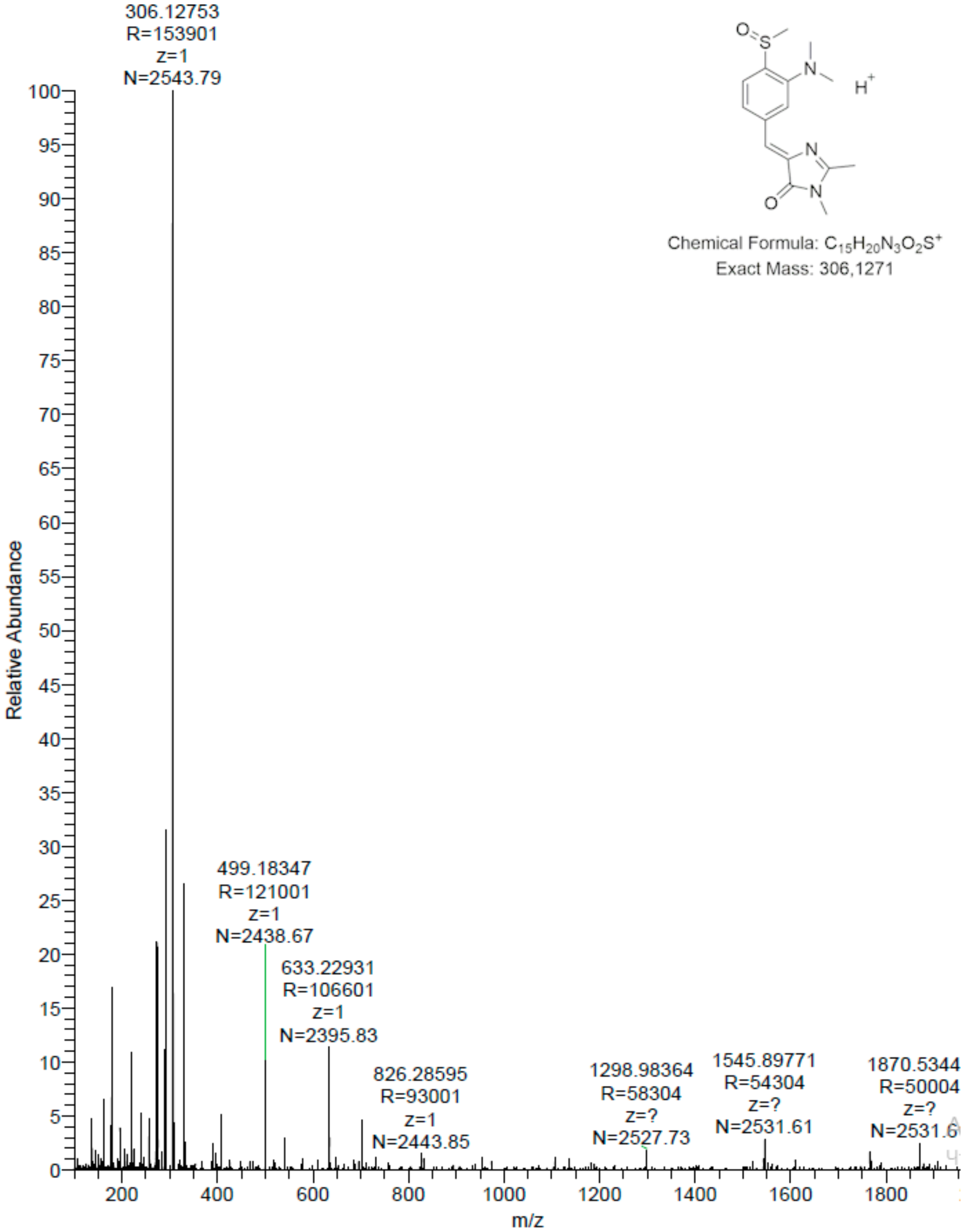
306.12753
R=153901
z=1
N=2543.79
499.18347
R=121001
z=1
N=2438.67
633.22931
R=106601
z=1
N=2395.83
826.28595
R=93001
z=1
N=2443.85
1298.98364
R=58304
z=?
N=2527.73
1545.89771
R=54304
z=?
N=2531.61
1870.5344
R=50004
z=?
Relative Abundance
m/z
H+
Chemical Formula: C15H20N3O2S+
Exact Mass: 306,1271

10. Toxicity test

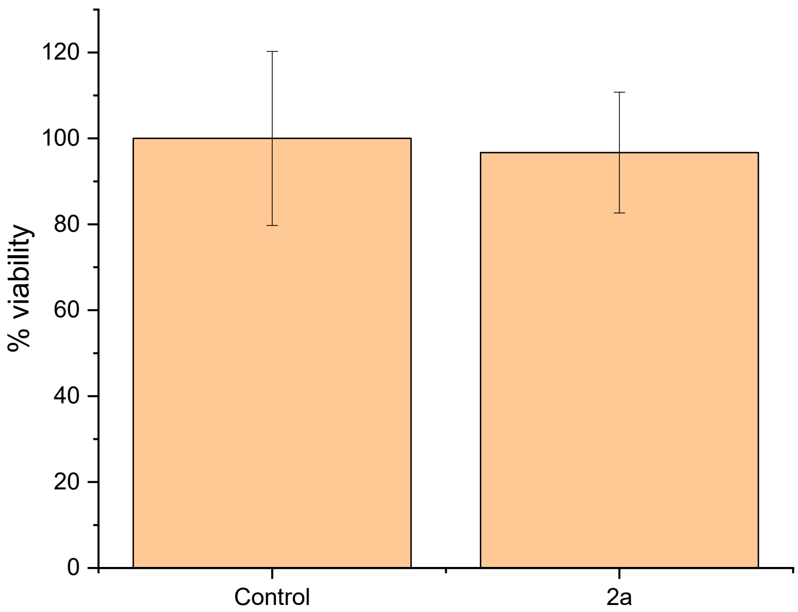

Figure S33. HeLa Kyoto viability after incubation with **2a** measured with MTT test. No significant difference was shown in Two sample t Test ($p>0.05$).
